# Supplementary material for: Modulation of metal species as control point for Ni-catalyzed stereodivergent semihydrogenation of alkynes with water
Source: Nat Commun. 2023 Mar 24;14:1655. doi: 10.1038/s41467-023-37022-w (PMC10039052; doi:10.1038/s41467-023-37022-w)
Supplement: Supplementary file 1 — Supplementary Information [file 41467_2023_37022_MOESM1_ESM.pdf]

Supplementary Information

Modulation of metal species as control point for Ni-catalyzed stereodivergent semihydrogenation of alkynes with water

Yuanqi Wu[1], Yuhui Ao[1], Zhiming Li[2]*, Chunhui Liu[3], Jinbo Zhao[1], Wenyu Gao[1], Xuemeng Li[1], Hui Wang[1],

Yongsheng Liu[1] & Yu Liu[1]*

[1]Jilin Provincial Key Laboratory of Carbon Fiber Development and Application, College of Chemistry and Life Science, Advanced Institute of Materials Science, Changchun University of Technology, 130012 Changchun, PR China. [2]Department of Chemistry, Fudan University, 200438 Shanghai, PR China. [3]College of Chemical and Materials Engineering, Xuchang University, 461000 Xuchang, PR China. ✉email: zmli@fudan.edu.cn; yuliu@ccut.edu.cn

Table of Contents

1. Supplementary Tables.. 4

Supplementary Table 1. Screening of Solvent and Catalyst for Transfer Semihydrogenation of Alkynes... 4

Supplementary Table 2. Screening of the Amount of Nickel Catalyst, Ligand, Base, Boron and Water for Transfer Semihydrogenation of Alkynes...................................... 4

Supplementary Table 3. Screening of Base for Transfer Semihydrogenation of Alkynes 5

2. Supplementary Methods.. 5

2.1 General Information 5

2.2 Deuterium-labeled Experiments 6

2.3 Control Experiments on Hydrolysis of Vinylboron Derivatives 4, 5, 4', 5' 8

2.4 The Kinetic Behavior of the Catalytic Systems.. 12

2.4.1 Kinetic Profile of Transfer Semihydrogenation of 1a 12

2.4.2 Kinetic Isotopic Effect of Transfer Semihydrogenation of 1a 13

2.5 Control Experiments on *Z*-*E* Isomerization.. 14

2.6 The Color Change of the Two Hydrogenation Systems... 15

2.7 Control Experiments on Base Effect... 16

2.8. Mercury Poison Test 16

2.9 EPR Experiments. 17

2.10 Control Experiments on Ni(I) Species 18

2.11 Investigations on Adding Reductants in Condition A... 18

2.12 Control Experiments with Reductants Instead of B_2Pin_2 in Condition A........... 19

2.13 In Situ ^{1}H NMR Analysis of the Reaction System 19

2.14 Density functional theory (DFT) calculations.. 20

2.14.1 Computations for *Z*-selective Hydrogenation 21

2.14.2 Computations for Isomerization 21

2.15 Synthesis of Alkynes 1 22

2.16 General Procedure for *Z*-Selective Transfer Semihydrogenation of Alkynes 1... 23

2.17 General Procedure for *E*-Selective Transfer Semihydrogenation of Alkynes 1 .. 34

2.18 Procedure for Transfer Semihydrogenation of Alkyne 1mm 43

2.19 Procedure for Transfer Semihydrogenation of Alkyne 1nn 44

2.20 Procedure for Transfer Semihydrogenation of Alkyne 1oo 44

2.21 Procedure for Transfer Semihydrogenation of Alkyne 1pp 44

2.22 Procedure for Transfer Semihydrogenation of Alkyne 1qq 45

3. Supplementary References 45

1. Supplementary Tables

Supplementary Table 1. Screening of Solvent and Catalyst for Transfer Semihydrogenation of Alkynes.

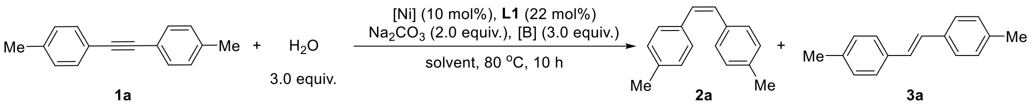

Entry	[Ni]	[B]	solvent	conv. /%[b]	yield/%[b]	**2a/3a**[b]
1	$NiBr_2$	B_2Pin_2	THF	83	46	14/86
2	$NiBr_2$	B_2Pin_2	DME	100	75	20/80
3	$NiBr_2$	B_2Pin_2	DCM	0	0	-
4	$NiBr_2$	B_2Pin_2	MeOH	100	25	56/44
5	$NiBr_2$	B_2Pin_2	^{t}BuOH	71	47	30/70
5	$NiBr_2$	B_2Pin_2	toluene	0	0	-
6	$NiBr_2$	B_2Pin_2	MeCN	100	70	17/83
7	$NiBr_2$	B_2Pin_2	DMF	100	72	11/89
8	$NiBr_2$	B_2Pin_2	DMSO	100	63	15/85
9	$NiBr_2$	B_2Pin_2	NMP	100	61	16/84
10	$NiBr_2$	B_2Pin_2	DMA	90	64	16/84
11	$NiBr_2$	B_2Pin_2	THF/DMF: 1/1	100	72	12/88
12	$NiBr_2$	B_2Pin_2	dioxane/DMF: 1/1	100	72	11/89
13	$Ni(acac)_2$	B_2Pin_2	DMF	20	14	40/60
14	NiF_2	B_2Pin_2	DMF	0	0	-
15	$NiBr_2{\cdot}dme$	B_2Pin_2	DMF	100	67	13/87
16	$NiCl_2$	B_2Pin_2	DMF	100	70	11/89
17	$NiBr_2$	$B_2(OH)_4$	DMF	100	76	21/79
18	$NiBr_2$	B_2cat_2	DMF	100	33	9/91
19	$NiBr_2$	B_2neop_2	DMF	100	75	15/85

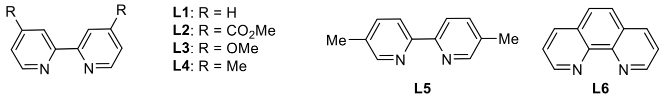

[a]Reactions were performed with **1a** (0.15 mmol), solvent (2 mL), 80 °C, 10 h; [b]Determined by crude ^{1}H NMR.

Supplementary Table 2. Screening of the Amount of Nickel Catalyst, Ligand, Base, Boron and Water for Transfer Semihydrogenation of Alkynes

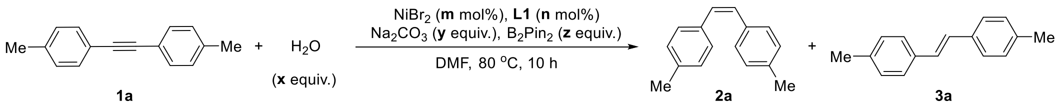

Entry	**m**	**n**	**x**	**y**	**z**	conv. /%[b]	yield/%[b]	**2a/3a**[b]
1	5	11	3	2	3	100	75	11/89
2	7.5	16.5	3	2	3	100	57	17/83
3	5	22	3	2	3	100	72	12/88
4	2.5	7	3	2	3	20	17	11/89
5	5	11	3	1	3	100	69	14/86
6	5	11	2	2	3	100	67	13/87
7	5	11	3	2	2	100	63	12/88

[a]Reactions were performed with **1a** (0.15 mmol), solvent (2 mL), 80 °C, 10 h; [b]Determined by crude ^{1}H NMR.

Supplementary Table 3. Screening of Base for Transfer Semihydrogenation of Alkynes

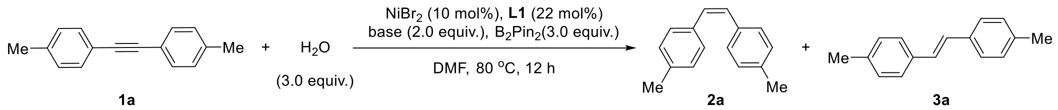

Entry	base	conv. /%[b]	yield/%[b]	**2a/3a**[b]
1	Na_2CO_3	100	72[c]	11/89
2	K_2CO_3	100	42	22/78
3	Cs_2CO_3	100	27	25/75
4	$NaHCO_3$	100	62	14/86
5	K_3PO_4	81	39	16/84
6	KH_2PO_4	29	28	43/57
7	NaOH	100	30	33/67
8	KOH	85	27	20/80
9	CsF	0	0	-
10	DBU	100	80	24/76
11	DABCO	100	74	12/88
12	Et_2NH	100	53	19/81
13	Et_3N	90	62	11/89
14	DIPEA	100	70	11/89
15	TMEDA	100	65	10/90
16	Pyridine	25	0	-
17	CF_3CO_2Na	100	84[c]	6/94
18	HCO_2K	100	77	67/33
19	CH_3CH_2ONa	100	40	60/40
20	CH_3CO_2K	100	87	70/30
21	CH_3CO_2Cs	100	97	72/28
22	HCO_2Na	100	85	56/44
23	$PhCO_2Na$	100	93	80/20
24	-	0	0	-
25[d]	CF_3CO_2Na	0	0	-

[a]Reactions were performed with **1a** (0.15 mmol), $NiBr_2$ (10 mol%), **L1** (22 mol%), base (2.0 equiv.), B_2Pin_2 (3.0 equiv.), H_2O (3.0 equiv.), DMF (2 mL), 80 °C, 12 h; [b]Determined by crude ^{1}H NMR; [c]Isolated yield; [d]Reactions were performed at rt.

2. Supplementary Methods

2.1 General Information

Commercially available materials were used without further purification. All reactions were carried out under an atmosphere of argon in flame-dried glassware with magnetic stirring. ^{1}H NMR spectra, ^{19}F NMR spectra, ^{13}C NMR spectra were recorded on Bruker 300 and 400 MHz spectrometer in $CDCl_3$. All signals are reported in ppm with the internal TMS signal at 0 ppm as a standard. Data for ^{1}H NMR spectra are reported as follows: chemical shift (ppm, referenced to TMS; s = singlet, d = doublet, t = triplet, q = quarte, dd = doublet of doublets, dt = doublet of triplets, m = multiplet), coupling constant (Hz), and intergration. Data for ^{13}C NMR are reported in terms of chemical shift (ppm) relative to residual solvent peak ($CDCl_3$: 77.0 ppm). EPR spectra were recorded on a Bruker E-500 spectrometer. Reactions were monitored by thin layer chromatography (TLC) using silica gel plates. Flash column chromatography was performed over silica gel (300-

400 mesh).

2.2 Deuterium-labeled Experiments

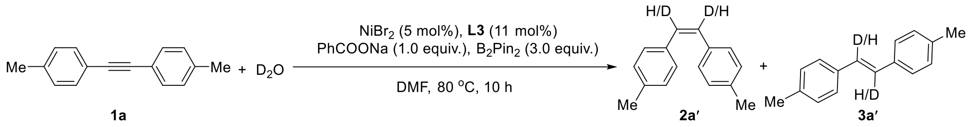

To a dry sealed tube were added alkyne **1a** (62 mg, 0.3 mmol), $NiBr_2$ (3.3 mg, 0.015 mmol, 5 mol%), **L3** (7.1 mg, 0.033 mmol, 11 mol%), $PhCO_2Na$ (43.2 mg, 0.3 mmol, 1.0 equiv.) and B_2Pin_2 (228.5 mg, 0.9 mmol, 3.0 equiv.). The flask was evacuated and refilled with argon, followed by the addition of D_2O (16.3 μL, 0.9 mmol, 3.0 equiv.) and DMF (4 mL). The mixture was stirred at 80 °C for 10 h until the reaction was completed as monitored by TLC. The resultant solution was diluted with ethyl acetate, washed with HCl aqueous solution (1 M) and concentrated in vacuum. The residue was detected by ^1H NMR analysis with CH_2Br_2 as an internal standard to report *Z*/*E* ratios, and purified by chromatography on silica gel, eluting with petroleum ether to give alkene product **2a'** as a white solid (57.5 mg, 92%, 92/8 *Z/E*, D/H = 84/16). ^1H NMR (400 MHz, $CDCl_3$) δ 7.26 (d, *J* = 7.8 Hz, 4 H), 7.12 (d, *J* = 7.7 Hz, 4 H), 6.60 (s, 0.32 H), 2.40 (s, 6 H). ^{13}C NMR (101 MHz, $CDCl_3$) δ 136.68, 134.41, 129.36, 128.85, 128.72, 21.20.

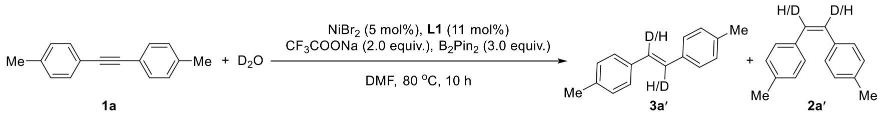

To a dry sealed tube were added alkyne **1a** (62 mg, 0.3 mmol), $NiBr_2$ (3.3 mg, 0.015 mmol, 5 mol%), **L1** (5.2 mg, 0.033 mmol, 11 mol%), CF_3COONa (81.6 mg, 0.6 mmol, 2.0 equiv.) and B_2Pin_2 (228.5 mg, 0.9 mmol, 3.0 equiv.). The flask was evacuated and refilled with argon, followed by the addition of D_2O (16.3 μL, 0.9 mmol, 3.0 equiv.) and DMF (4 mL). The mixture was stirred at 80 °C for 10 h until the reaction was completed as monitored by TLC. The resultant solution was diluted with ethyl acetate, washed with HCl aqueous solution (1 M) and concentrated in vacuum. The residue was detected by ^1H NMR analysis with CH_2Br_2 as an internal standard to report *Z*/*E* ratios, and purified by chromatography on silica gel, eluting with petroleum ether to give alkene product **3a'** as a white solid (56.7 mg, 91%, 96/4 *E/Z*, D/H = 71/29). ^1H NMR (400 MHz, $CDCl_3$) δ 7.43 (d, *J* = 7.6 Hz, 4 H), 7.18 (d, *J* = 7.6 Hz, 4 H), 7.06 (s, 0.59 H), 2.38 (s, 6H). ^{13}C NMR (101 MHz, $CDCl_3$) δ 137.22, 134.69, 129.35, 127.54, 126.28, 21.21.

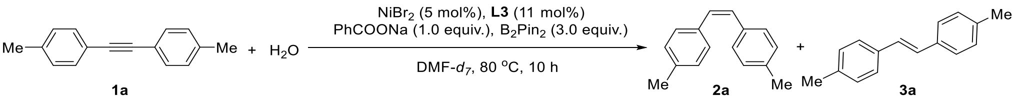

To a dry sealed tube were added alkyne **1a** (62 mg, 0.3 mmol), $NiBr_2$ (3.3 mg, 0.015 mmol, 5 mol%), **L3** (7.1 mg, 0.033 mmol, 11 mol%), $PhCO_2Na$ (43.2 mg, 0.3 mmol, 1.0 equiv.) and B_2Pin_2 (228.5 mg, 0.9 mmol, 3.0 equiv.). The flask was evacuated and refilled with argon, followed by the addition of H_2O (16.2 μL, 0.9 mmol, 3.0 equiv.) and DMF-d_7 (4 mL). The mixture was stirred at 80 °C for 10 h until the reaction was completed as monitored by TLC. The resultant solution was diluted with ethyl acetate, washed with HCl aqueous solution (1 M) and concentrated in vacuum. The residue was detected by ^1H NMR analysis with CH_2Br_2 as an internal standard to report *Z*/*E* ratios, and purified by chromatography on silica gel, eluting with petroleum ether to give alkene product **2a** as a white solid (49.7 mg, 82%, 95/5 *Z/E*). ^1H NMR (400 MHz, $CDCl_3$) δ 7.21 (d, *J* = 8.0 Hz, 4 H), 7.08 (d, *J* = 7.9 Hz, 4 H), 6.56 (s, 2 H), 2.36 (s, 6 H). ^{13}C NMR (101 MHz, $CDCl_3$) δ

136.68, 134.49, 129.50, 128.86, 128.73, 21.20.

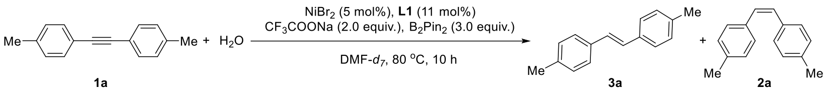

To a dry sealed tube were added alkyne **1a** (62 mg, 0.3 mmol), $NiBr_2$ (3.3 mg, 0.015 mmol, 5 mol%), **L1** (5.2 mg, 0.033 mmol, 11 mol%), CF_3COONa (81.6 mg, 0.6 mmol, 2.0 equiv.) and B_2Pin_2 (228.5 mg, 0.9 mmol, 3.0 equiv.). The flask was evacuated and refilled with argon, followed by the addition of H_2O (16.2 μL, 0.9 mmol, 3.0 equiv.) and DMF-d_7 (4 mL). The mixture was stirred at 80 °C for 10 h until the reaction was completed as monitored by TLC. The resultant solution was diluted with ethyl acetate, washed with HCl aqueous solution (1 M) and concentrated in vacuum. The residue was detected by ^{1}H NMR analysis with CH_2Br_2 as an internal standard to report *Z*/*E* ratios, and purified by chromatography on silica gel, eluting with petroleum ether to give alkene product **3a** as a white solid (52.4 mg, 84%, 94/6 *E/Z*). ^{1}H NMR (400 MHz, $CDCl_3$) δ 7.38 (d, J = 8.0 Hz, 4 H), 7.14 (d, J = 7.9 Hz, 4 H), 7.02 (s, 2 H), 2.34 (s, 6 H). ^{13}C NMR (101 MHz, $CDCl_3$) δ 137.22, 134.74, 129.34, 127.64, 126.29, 21.19.

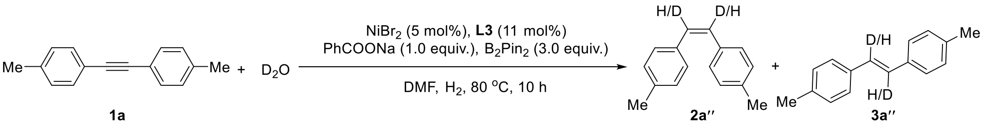

To a dry sealed tube were added alkyne **1a** (62 mg, 0.3 mmol), $NiBr_2$ (3.3 mg, 0.015 mmol, 5 mol%), **L3** (7.1 mg, 0.033 mmol, 11 mol%), $PhCO_2Na$ (43.2 mg, 0.3 mmol, 1.0 equiv.) and B_2Pin_2 (228.5 mg, 0.9 mmol, 3.0 equiv.). The flask was evacuated and refilled with hydrogen, followed by the addition of D_2O (16.3 μL, 0.9 mmol, 3.0 equiv.) and DMF (4 mL). The mixture was stirred at 80 °C for 10 h until the reaction was completed as monitored by TLC. The resultant solution was diluted with ethyl acetate, washed with HCl aqueous solution (1 M) and concentrated in vacuum. The residue was detected by ^{1}H NMR analysis with CH_2Br_2 as an internal standard to report *Z*/*E* ratios, and purified by chromatography on silica gel, eluting with petroleum ether to give alkene product **2a''** as a white solid (57.3 mg, 91%, 95/5 *Z/E*, D/H = 78/22). ^{1}H NMR (400 MHz, $CDCl_3$) δ 7.16 (d, J = 7.9 Hz, 4 H), 7.02 (d, J = 7.7 Hz, 4 H), 6.50 (s, 0.4 H), 2.30 (s, 6 H). ^{13}C NMR (101 MHz, $CDCl_3$) δ 136.68, 134.41, 129.36, 128.85, 128.72, 21.19.

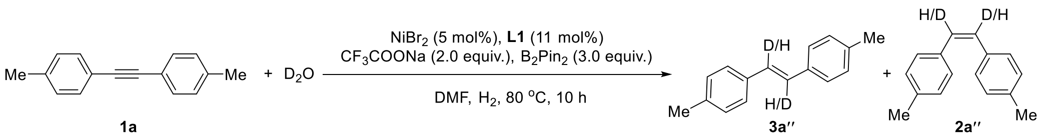

To a dry sealed tube were added alkyne **1a** (62 mg, 0.3 mmol), $NiBr_2$ (3.3 mg, 0.015 mmol, 5 mol%), **L1** (5.2 mg, 0.033 mmol, 11 mol%), CF_3COONa (81.6 mg, 0.6 mmol, 2.0 equiv.) and B_2Pin_2 (228.5 mg, 0.9 mmol, 3.0 equiv.). The flask was evacuated and refilled with hydrogen, followed by the addition of D_2O (16.3 μL, 0.9 mmol, 3.0 equiv.) and DMF (4 mL). The mixture was stirred at 80 °C for 10 h until the reaction was completed as monitored by TLC. The resultant solution was diluted with ethyl acetate, washed with HCl aqueous solution (1 M) and concentrated in vacuum. The residue was detected by ^{1}H NMR analysis with CH_2Br_2 as an internal standard to report *Z*/*E* ratios, and purified by chromatography on silica gel, eluting with petroleum ether to give alkene product **3a''** as a white solid (56.7 mg, 90%, 91/9 *E/Z*, D/H = 76/24). ^{1}H NMR (400 MHz, $CDCl_3$) δ 7.43 (d, J = 7.7 Hz, 4 H), 7.18 (d, J = 7.7 Hz, 4 H), 7.06 (s, 0.48 H), 2.38 (s, 6 H). ^{13}C NMR (101

MHz, $CDCl_3$) δ 137.23, 134.65, 129.35, 127.51, 126.27, 21.21.

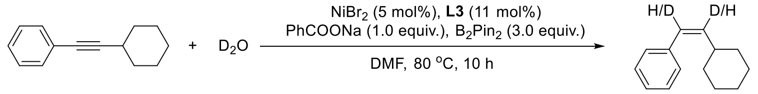

To a dry sealed tube were added alkyne **1bb** (55.3 mg, 0.3 mmol), $NiBr_2$ (3.3 mg, 0.015 mmol, 5 mol%), **L3** (7.1 mg, 0.033 mmol, 11 mol%), $PhCO_2Na$ (43.2 mg, 0.3 mmol, 1.0 equiv.) and B_2Pin_2 (228.5 mg, 0.9 mmol, 3.0 equiv.). The flask was evacuated and refilled with argon, followed by the addition of D_2O (16.3 μL, 0.9 mmol, 3.0 equiv.) and DMF (4 mL). The mixture was stirred at 80 °C for 10 h until the reaction was completed as monitored by TLC. The resultant solution was diluted with ethyl acetate, washed with HCl aqueous solution (1 M) and concentrated in vacuum. The residue was detected by ^{1}H NMR analysis with CH_2Br_2 as an internal standard to report *Z*/*E* ratios, and purified by chromatography on silica gel, eluting with petroleum ether to give alkene product **2bb'** as a colorless oil (58.7 mg, 97%, >99/1 *Z/E*, D/H = 93/7, D/H = 78/22). ^{1}H NMR (400 MHz, $CDCl_3$) δ 7.38 (t, J = 7.5 Hz, 2 H), 7.32 (d, J = 7.2 Hz, 2 H), 7.27 (t, J = 7.1 Hz, 7 H), 6.36 (s, 0.7 H), 5.54 (d, J = 10.1 Hz, 0.22 H), 2.64 (t, J = 10.2 Hz, 1 H), 1.86-1.76 (m, 4 H), 1.42-1.14 (m, 6 H). ^{13}C NMR (101 MHz, $CDCl_3$) δ 138.84, 137.90, 128.58, 128.16, 126.39, 36.77, 33.23, 26.03, 25.67.

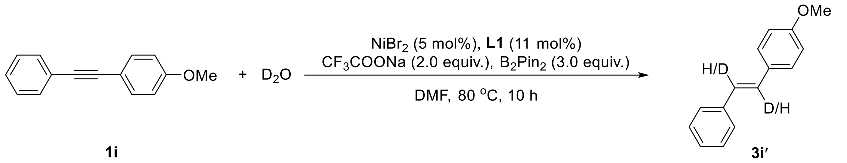

To a dry sealed tube were added alkyne **1i** (62.5 mg, 0.3 mmol), $NiBr_2$ (3.3 mg, 0.015 mmol, 5 mol%), **L1** (5.2 mg, 0.033 mmol, 11 mol%), CF_3COONa (81.6 mg, 0.6 mmol, 2.0 equiv.) and B_2Pin_2 (228.5 mg, 0.9 mmol, 3.0 equiv.). The flask was evacuated and refilled with argon, followed by the addition of D_2O (16.3 μL, 0.9 mmol, 3.0 equiv.) and DMF (4 mL). The mixture was stirred at 80 °C for 10 h until the reaction was completed as monitored by TLC. The resultant solution was diluted with ethyl acetate, washed with HCl aqueous solution (1 M) and concentrated in vacuum. The residue was detected by ^{1}H NMR analysis with CH_2Br_2 as an internal standard to report *Z*/*E* ratios, and purified by chromatography on silica gel, eluting with petroleum ether to give alkene product **3i'** as a white solid (53.2 mg, 82%, 94/6 *E/Z*, D/H = 71/29, D/H = 74/26). ^{1}H NMR (400 MHz, $CDCl_3$) δ 7.47 (dd, J = 13.5, 8.1 Hz, 4 H), 7.34 (t, J = 7.5 Hz, 2 H), 7.28-7.18 (m, 1 H), 7.07 (d, J = 12.8 Hz, 0.29 H), 6.98 (d, J = 8.9 Hz, 0.26 H), 6.90 (d, J = 8.6 Hz, 2 H), 3.83 (s, 3 H). ^{13}C NMR (101 MHz, $CDCl_3$) δ 159.32, 137.60, 130.11, 128.63, 128.12, 127.69, 127.18, 126.53, 126.24, 126.22, 114.15, 55.32.

2.3 Control Experiments on Hydrolysis of Vinylboron Derivatives 4, 5, 4', 5'

Vinylboron reagent **4** was synthesized according to published procedures[1]. A flame-dried Schlenk tube was charged with $Co(acac)_2$ (3.08 mg, 0.012 mmol, 3 mol%) and xantphos (8.8 mg, 0.016 mmol, 4 mol%). The flask was evacuated and refilled with argon for three times. Then, 2 ml of THF was added and the mixture was stirred for 5 min at room temperature. After, the pinacolborane (87 µl, 0.6 mmol, 1.5 equiv.) is added and the mixture was stirred for 20 min more. Finally, diphenylacetylene (71 mg, 0.4 mmol, 1.0 equiv.) was added, and the mixture was stirred at room temperature for 24 h. The resulting residue was purified by flash chromatography (petroleum ether:ethyl acetate = 30:1) to afford (*Z*)-2-(1,2-diphenylvinyl)-4,4,5,5-tetramethyl-1,3,2-dioxaborolane **4** as a white soild.

Vinylboron derivative **5** was synthesized according to published procedures[2,3]. (2,2-

dibromovinyl)benzene (262 mg, 1.0 mmol, 1.0 equiv.) was added to a 50 ml flame-dried schlenk tube equipped with a magnetic stir bar and dissolved in diethyl ether (4 mL) and tetrahydrofuran (7 mL). The reaction mixture was cooled to -80 °C. The solution was allowed to stir vigorously for 10 min before the dropwise addition of *n*-Butyl-lithium (0.42 mL, 2.5 M in hexanes, 1.05 mmol, 1.05 equiv.). The reaction was allowed to stir for an additional 30 min before the dropwise addition of a solution of B_2pin_2 (267 mg, 1.05 mmol, 1.05 equiv) in tetrahydrofuran (3 mL). The reaction was then allowed to gradually warm to room temperature and stirred for 20 hours at 23 °C after which the reaction was quenched with a saturated solution of ammonium chloride. The organics were extracted with diethyl ether for 3 times, and the combined organics were washed with brine, dried over magnesium sulfate, filtered, and concentrated in vacuo. The crude product was purified via silica gel flash chromatography (petroleum ether:ethyl acetate = 20:1) to afford the desired 2,2'-(2-phenylethene-1,1-diyl)bis(4,4,5,5-tetramethyl-1,3,2-dioxaborolane) as a white solid.

A flame-dried schlenk tube was charged with $Pd(OAc)_2$ (1.3 mg, 6.0 μmol, 3 mol%), ^{t}Bu-JohnPhos (2.0 mg, 6.6 μmol, 3.3 mol%), 2,2'-(2-phenylethene-1,1-diyl)bis(4,4,5,5-tetramethyl-1,3,2-dioxaborolane) (71 mg, 0.2 mmol, 1.0 equiv.), iodobenzene (41 mg, 0.2 mmol, 1.0 equiv.), KOH (22.4 mg, 0.4 mmol, 2.0 equiv.) and DMF (1.0 mL). The reaction was stirred for 30 °C. After stirring for 12 h, the reaction mixture was cooled to room temperature. Then $H_2O$was added to the mixture and the aqueous phase was extracted with diethyl ether for three times. The combined organic extracts were washed with water and brine, dried with $MgSO_4$ and concentrated in vacuo, and purified by flash chromatography on silica (petroleum ether:ethyl acetate = 50:1 to 10:1) to afford (*E*)-2-(1,2-diphenylvinyl)-4,4,5,5-tetramethyl-1,3,2-dioxaborolane **5**.

Vinylboron derivative **4'** was synthesized according to published procedures[4]. In a glovebox, B_2pin_2 (254.0 mg, 1.0 mmol, 2 equiv.), NaOH (5.0 mg, 0.125 mmol, 0.25 equiv.), THF (1.0 mL), diphenylacetylene (89.1 mg, 0.5 mmol, 1.0 equiv.) and MeOH (100 μL, 5 equiv.) were added to a flame-dried schlenk tube. The reaction was allowed to stir at 80 °C for 24 h. The reaction mixture was diluted with ethyl acetate, filtered through a silica gel plug, washed with ethyl acetate, and concentrated in vacuo. The residue was purified on silica gel column chromatography (petroleum ether:ethyl acetate = 30:1) to afford (*Z*)-1,2-diphenyl-1,2-bis(4,4,5,5-tetramethyl-1,3,2-dioxaborolan-2-yl)ethene **4'** as a white solid.

Vinylboron derivative **5'** was synthesized according to published procedures[5,6]. To a sealed tube were added $Cu(OAc)_2$ (3.6 mg, 0.02 mmol, 10 mol %), B_2pin_2 (152.4 mg, 0.6 mmol, 3 equiv.) and toluene (1 mL). Then, phenylacetylene (20 mg, 0.2 mmol, 1.0 equiv.), acrylonitrile (11 mg, 0.2 mmol, 1.0 equiv.) and P^nBu_3 (10 μL, 0.04 mmol, 20 mol%) were added in that order. The reaction was heated at 80 °C under argon for12 h. The reaction mixture was then diluted with Et_2O and filtered through a plug of celite. The solvents were removed in vacuo, and the residue was purified by column chromatography on silica gel (petroleum ether:ethyl acetate = 30:1) to afford 2,2',2''-(2-phenylethene-1,1,2-triyl)tris(4,4,5,5-tetramethyl-1,3,2-dioxaborolane) as a brown solid.

To a sealed tube were added $Pd(PPh_3)_4$ (35 mg, 0.03 mol, 10 mol%), 2,2',2''-(2-phenylethene-1,1,2-triyl)tris(4,4,5,5-tetramethyl-1,3,2-dioxaborolane) (144 mg, 0.3 mmol, 1.0 equiv.), and iodobenzene (61.2 mg, 0.3 mmol, 1.0 equiv.). The flask was evacuated and refilled with argon. THF (3 mL) and degassed aqueous K_3PO_4 (1.5 M, 0.6 mL) were added via syringes. The reaction mixture was stirred at 70 °C for 24 h. After cooling to the room temperature, the mixture was filtered through a pad of Celite and washed with Et_2O. The filtrate was concentrated under vacuum. The residue was purified by flash column chromatography (petroleum ether:ethyl acetate = 30:1) to afford (*E*)-1,2-

diphenyl-1,2-bis(4,4,5,5-tetramethyl-1,3,2-dioxaborolan-2-yl)ethene **5'** as a white solid.

To a sealed tube were added vinylboron derivative **4** (48 mg, 0.15 mmol), $NiBr_2$ (1.7 mg, 0.0075 mmol, 5 mol%), **L3** (3.6 mg, 0.0165 mmol, 11 mol%), $PhCO_2Na$ (21.6 mg, 0.15 mmol, 1.0 equiv.) and B_2Pin_2 (114.3 mg, 0.45 mmol, 3.0 equiv.). The flask was evacuated and refilled with argon, followed by the addition of H_2O (8.1 μL, 0.45 mmol, 3.0 equiv.) and DMF (2 mL), and stirred at 80 °C for 10 h. The resultant solution was diluted with ethyl acetate, washed with HCl aqueous solution (1 M) and concentrated in vacuum. The residue was detected by ^1H NMR analysis with CH_2Br_2 as the internal standard.

To a sealed tube were added vinylboron derivative **4** (24.7 mg, 0.081 mmol), $NiBr_2$ (0.9 mg, 0.00405 mmol, 5 mol%), **L3** (1.9 mg, 0.00891 mmol, 11 mol%), $PhCO_2Na$ (11.7 mg, 0.081 mmol, 1.0 equiv.) and B_2Pin_2 (41.1 mg, 0.162 mmol, 2.0 equiv.). The flask was evacuated and refilled with argon, followed by the addition of H_2O (4.4 μL, 0.243 mmol, 3.0 equiv.) and DMF (1.1 mL), and stirred at 80 °C for 12 h. The resultant solution was diluted with ethyl acetate, washed with HCl aqueous solution (1 M) and concentrated in vacuum. The residue was detected by ^1H NMR analysis with CH_2Br_2 as the internal standard.

To a sealed tube were added vinylboron derivative **4** (24.7 mg, 0.081 mmol), $NiBr_2$ (0.9 mg, 0.00405 mmol, 5 mol%), **L3** (1.9 mg, 0.00891 mmol, 11 mol%), $PhCO_2Na$ (11.7 mg, 0.081 mmol, 1.0 equiv.) and B_2Pin_2 (20.6 mg, 0.081 mmol, 1.0 equiv.). The flask was evacuated and refilled with argon, followed by the addition of H_2O (4.4 μL, 0.243 mmol, 3.0 equiv.) and DMF (1.1 mL), and stirred at 80 °C for 12 h. The resultant solution was diluted with ethyl acetate, washed with HCl aqueous solution (1 M) and concentrated in vacuum. The residue was detected by ^1H NMR analysis with CH_2Br_2 as the internal standard.

To a sealed tube were added vinylboron derivative **4** (24.7 mg, 0.081 mmol), $NiBr_2$ (0.9 mg, 0.00405 mmol, 5 mol%), **L3** (1.9 mg, 0.00891 mmol, 11 mol%) and $PhCO_2Na$ (11.7 mg, 0.081 mmol, 1.0 equiv.). The flask was evacuated and refilled with argon, followed by the addition of H_2O (4.4 μL, 0.243 mmol, 3.0 equiv.) and DMF (1.1 mL), and stirred at 80 °C for 12 h. The resultant solution was diluted with ethyl acetate, washed with HCl aqueous solution (1 M) and concentrated in vacuum. The residue was detected by ^1H NMR analysis with CH_2Br_2 as the internal standard.

To a sealed tube were added vinylboron derivative **5** (48 mg, 0.15 mmol), $NiBr_2$ (1.7 mg, 0.0075 mmol, 5 mol%), **L1** (2.6 mg, 0.0165 mmol, 11 mol%), CF_3CO_2Na (40.8 mg, 0.3 mmol, 2.0 equiv.) and B_2Pin_2 (114.3 mg, 0.45 mmol, 3.0 equiv.). The flask was evacuated and refilled with argon, followed by the addition of H_2O (8.1 μL, 0.45 mmol, 3.0 equiv.) and DMF (2 mL), and stirred at 80°C for 10 h. The resultant solution was diluted with ethyl acetate, washed with HCl aqueous solution (1 M) and concentrated in vacuum. The residue was detected by ^1H NMR analysis at given time with CH_2Br_2 as the internal standard.

To a sealed tube were added vinylboron derivative **5** (17.6 mg, 0.057 mmol), $NiBr_2$ (0.6 mg, 0.00285 mmol, 5 mol%), **L1** (1 mg, 0.00627 mmol, 11 mol%), CF_3CO_2Na (15.5 mg, 0.114 mmol, 2.0 equiv.) and B_2Pin_2 (29 mg, 0.114 mmol, 2.0 equiv.). The flask was evacuated and refilled with argon, followed by the addition of H_2O (3.1 μL, 0.171 mmol, 3.0 equiv.) and DMF (0.8 mL), and stirred at 80°C for 12 h. The resultant solution was diluted with ethyl acetate, washed with HCl aqueous solution (1 M) and concentrated in vacuum. The residue was detected by ^1H NMR analysis at given time with CH_2Br_2 as the internal standard.

To a sealed tube were added vinylboron derivative **5** (17.6 mg, 0.057 mmol), $NiBr_2$ (0.6 mg, 0.00285 mmol, 5 mol%), **L1** (1 mg, 0.00627 mmol, 11 mol%), CF_3CO_2Na (15.5 mg, 0.114 mmol,

2.0 equiv.) and B_2Pin_2 (14.5 mg, 0.057 mmol, 1.0 equiv.). The flask was evacuated and refilled with argon, followed by the addition of H_2O (3.1 μL, 0.171 mmol, 3.0 equiv.) and DMF (0.8 mL), and stirred at 80°C for 12 h. The resultant solution was diluted with ethyl acetate, washed with HCl aqueous solution (1 M) and concentrated in vacuum. The residue was detected by ^1H NMR analysis at given time with CH_2Br_2 as the internal standard.

To a sealed tube were added vinylboron derivative **5** (17.6 mg, 0.057 mmol), $NiBr_2$ (0.6 mg, 0.00285 mmol, 5 mol%), **L1** (1 mg, 0.00627 mmol, 11 mol%) and CF_3CO_2Na (15.5 mg, 0.114 mmol, 2.0 equiv.). The flask was evacuated and refilled with argon, followed by the addition of H_2O (3.1 μL, 0.171 mmol, 3.0 equiv.) and DMF (0.8 mL), and stirred at 80°C for 12 h. The resultant solution was diluted with ethyl acetate, washed with HCl aqueous solution (1 M) and concentrated in vacuum. The residue was detected by ^1H NMR analysis at given time with CH_2Br_2 as the internal standard.

To a sealed tube were added vinylboron compound **4'** (51.4 mg, 0.12 mmol), $NiBr_2$ (2.6 mg, 0.012 mmol, 10 mol%), **L3** (5.7 mg, 0.0264 mmol, 22 mol%), $PhCO_2Na$ (17.3 mg, 0.12 mmol, 1.0 equiv.) and B_2Pin_2 (91.4 mg, 0.36 mmol, 3.0 equiv.). The flask was evacuated and refilled with argon, followed by the addition of H_2O (6.5 μL, 0.45 mmol, 3.0 equiv.) and DMF (2 mL), and stirred at 80 °C for 12 h. The resultant solution was diluted with ethyl acetate, washed with aqueous HCl solution (1 M) and concentrated in vacuum. The residue was detected by ^1H NMR analysis with CH_2Br_2 as the internal standard.

To a sealed tube were added vinylboron compound **4'** (64.8 mg, 0.15 mmol), $NiBr_2$ (1.7 mg, 0.0075 mmol, 5 mol%), **L3** (3.6 mg, 0.0165 mmol, 11 mol%), $PhCO_2Na$ (21.6 mg, 0.15 mmol, 1.0 equiv.) and B_2Pin_2 (76.2 mg, 0.3 mmol, 2.0 equiv.). The flask was evacuated and refilled with argon, followed by the addition of H_2O (8.1 μL, 0.45 mmol, 3.0 equiv.) and DMF (2 mL), and stirred at 80 °C for 12 h. The resultant solution was diluted with ethyl acetate, washed with aqueous HCl solution (1 M) and concentrated in vacuum. The residue was detected by ^1H NMR analysis with CH_2Br_2 as the internal standard.

To a sealed tube were added vinylboron compound **4'** (64.8 mg, 0.15 mmol), $NiBr_2$ (1.7 mg, 0.0075 mmol, 5 mol%), **L3** (3.6 mg, 0.0165 mmol, 11 mol%), $PhCO_2Na$ (21.6 mg, 0.15 mmol, 1.0 equiv.) and B_2Pin_2 (38.1 mg, 0.15 mmol, 1.0 equiv.). The flask was evacuated and refilled with argon, followed by the addition of H_2O (8.1 μL, 0.45 mmol, 3.0 equiv.) and DMF (2 mL), and stirred at 80 °C for 12 h. The resultant solution was diluted with ethyl acetate, washed with aqueous HCl solution (1 M) and concentrated in vacuum. The residue was detected by ^1H NMR analysis with CH_2Br_2 as the internal standard.

To a sealed tube were added vinylboron compound **4'** (64.8 mg, 0.15 mmol), $NiBr_2$ (1.7 mg, 0.0075 mmol, 5 mol%), **L3** (3.6 mg, 0.0165 mmol, 11 mol%) and $PhCO_2Na$ (21.6 mg, 0.15 mmol, 1.0 equiv.). The flask was evacuated and refilled with argon, followed by the addition of H_2O (8.1 μL, 0.45 mmol, 3.0 equiv.) and DMF (2 mL), and stirred at 80 °C for 12 h. The resultant solution was diluted with ethyl acetate, washed with aqueous HCl solution (1 M) and concentrated in vacuum. The residue was detected by ^1H NMR analysis with CH_2Br_2 as the internal standard.

To a sealed tube were added vinylboron compound **5'** (42.5 mg, 0.1 mmol), $NiBr_2$ (2.2 mg, 0.01 mmol, 10 mol%), **L1** (3.4 mg, 0.022 mmol, 22 mol%), CF_3CO_2Na (27.4 mg, 0.2 mmol, 2.0 equiv.) and B_2Pin_2 (76.2 mg, 0.3 mmol, 3.0 equiv.). The flask was evacuated and refilled with argon, followed by the addition of H_2O (5.4 μL, 0.3 mmol, 3.0 equiv.) and DMF (1.5 mL), and stirred at 80°C for 12 h. The resultant solution was diluted with ethyl acetate, washed with aqueous HCl solution (1 M) and concentrated in vacuum. The residue was detected by ^1H NMR analysis at given

time with CH_2Br_2 as the internal standard.

To a sealed tube were added vinylboron compound **5'** (37.2 mg, 0.086 mmol), $NiBr_2$ (0.9 mg, 0.0043 mmol, 5 mol%), **L1** (1.5 mg, 0.00946 mmol, 11 mol%), CF_3CO_2Na (23.4 mg, 0.172 mmol, 2.0 equiv.) and B_2Pin_2 (43.7 mg, 0.172 mmol, 2.0 equiv.). The flask was evacuated and refilled with argon, followed by the addition of H_2O (4.6 µL, 0.258 mmol, 3.0 equiv.) and DMF (1.2 mL), and stirred at 80°C for 12 h. The resultant solution was diluted with ethyl acetate, washed with aqueous HCl solution (1 M) and concentrated in vacuum. The residue was detected by ^1H NMR analysis at given time with CH_2Br_2 as the internal standard.

To a sealed tube were added vinylboron compound **5'** (37.2 mg, 0.086 mmol), $NiBr_2$ (0.9 mg, 0.0043 mmol, 5 mol%), **L1** (1.5 mg, 0.00946 mmol, 11 mol%), CF_3CO_2Na (23.4 mg, 0.172 mmol, 2.0 equiv.) and B_2Pin_2 (21.8 mg, 0.086 mmol, 1.0 equiv.). The flask was evacuated and refilled with argon, followed by the addition of H_2O (4.6 µL, 0.258 mmol, 3.0 equiv.) and DMF (1.2 mL), and stirred at 80°C for 12 h. The resultant solution was diluted with ethyl acetate, washed with aqueous HCl solution (1 M) and concentrated in vacuum. The residue was detected by ^1H NMR analysis at given time with CH_2Br_2 as the internal standard.

To a sealed tube were added vinylboron compound **5'** (37.2 mg, 0.086 mmol), $NiBr_2$ (0.9 mg, 0.0043 mmol, 5 mol%), **L1** (1.5 mg, 0.00946 mmol, 11 mol%) and CF_3CO_2Na (23.4 mg, 0.172 mmol, 2.0 equiv.). The flask was evacuated and refilled with argon, followed by the addition of H_2O (4.6 µL, 0.258 mmol, 3.0 equiv.) and DMF (1.2 mL), and stirred at 80°C for 12 h. The resultant solution was diluted with ethyl acetate, washed with aqueous HCl solution (1 M) and concentrated in vacuum. The residue was detected by ^1H NMR analysis at given time with CH_2Br_2 as the internal standard.

2.4 The Kinetic Behavior of the Catalytic Systems

2.4.1 Kinetic Profile of Transfer Semihydrogenation of 1a

To a sealed tube were added alkyne **1a** (30.9 mg, 0.15 mmol), $NiBr_2$ (1.7 mg, 0.0075 mmol, 5 mol%), **L3** (3.6 mg, 0.0165 mmol, 11 mol%), $PhCO_2Na$ (21.6 mg, 0.15 mmol, 1.0 equiv.) and B_2Pin_2 (114.3 mg, 0.45 mmol, 3.0 equiv.). The flask was evacuated and refilled with argon, followed by the addition of H_2O (8.1 µL, 0.45 mmol, 3.0 equiv.) and DMF (2 mL), and stirred at 80 °C for given time. The resultant solution was diluted with ethyl acetate, washed with HCl aqueous solution (1 M) and concentrated in vacuum. The residue was detected by ^1H NMR analysis with CH_2Br_2 as the internal standard.

To a sealed tube were added alkyne **1a** (30.9 mg, 0.15 mmol), $NiBr_2$ (1.7 mg, 0.0075 mmol, 5 mol%), **L1** (2.6 mg, 0.0165 mmol, 11 mol%), CF_3CO_2Na (30.8 mg, 0.3 mmol, 1.0 equiv.) and B_2Pin_2 (114.3 mg, 0.45 mmol, 3.0 equiv.). The flask was evacuated and refilled with argon, followed by the addition of H_2O (8.1 µL, 0.45 mmol, 3.0 equiv.) and DMF (2 mL), and stirred at 80°C for given time. The resultant solution was diluted with ethyl acetate, washed with HCl aqueous solution (1 M) and concentrated in vacuum. The residue was detected by ^1H NMR analysis at given time with CH_2Br_2 as the internal standard.

2.4.2 Kinetic Isotopic Effect of Transfer Semihydrogenation of 1a

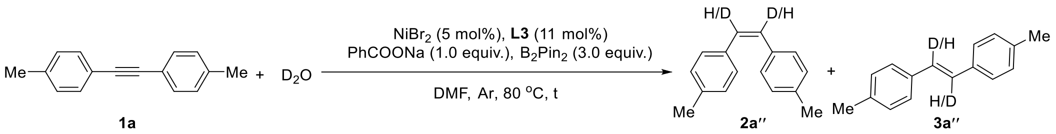

To a sealed tube were added alkyne **1a** (30.9 mg, 0.15 mmol), $NiBr_2$ (1.7 mg, 0.0075 mmol, 5 mol%), **L3** (3.6 mg, 0.0165 mmol, 11 mol%), $PhCO_2Na$ (21.6 mg, 0.15 mmol, 1.0 equiv.) and B_2Pin_2 (114.3 mg, 0.45 mmol, 3.0 equiv.). The flask was evacuated and refilled with argon, followed by the addition of D_2O (9.0 μL, 0.45 mmol, 3.0 equiv.) and DMF (2 mL), and stirred at 80 °C for given time. The resultant solution was diluted with ethyl acetate, washed with HCl aqueous solution (1 M) and concentrated in vacuum. The residue was detected by ^1H NMR analysis with CH_2Br_2 as the internal standard.

The low deuterium isotopic content in the deuterium labeling reactions (see part 2.2) might result from tiny amounts of water in the reactant system. The KIE results were calibrated by D/H ratios, as follows:

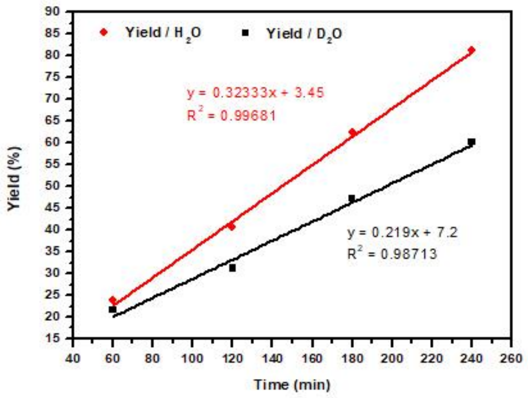

Supplementary Figure 1. The Kinetic Isotopic Effect of *Z*-selective Hydrogenation of **1a**. a H_2O was replaced by D_2O in the *Z*-selective reactant stream.

Unadjusted result of kinetic isotopic effect:

$$KIE' = k_H'/k_D' = 0.32333/0.219 = 1.48$$

Calibrated result of kinetic isotopic effect:

The D/H ratios of deuterium-labeled products all reached 80/20 in *Z*-selective reactions.

$$(100\%\ k_H)/(80\%\ k_D + 20\%\ k_H) = 0.32333/0.219$$

$$KIE = k_H/k_D = 1.68$$

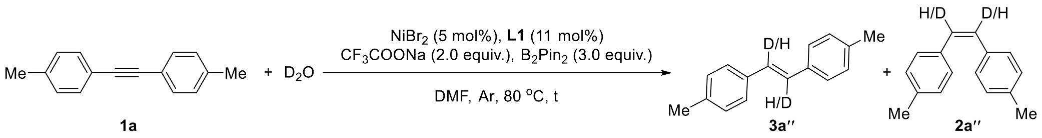

To a sealed tube were added alkyne **1a** (30.9 mg, 0.15 mmol), $NiBr_2$ (1.7 mg, 0.0075 mmol, 5 mol%), **L1** (2.6 mg, 0.0165 mmol, 11 mol%), CF_3CO_2Na (30.8 mg, 0.3 mmol, 1.0 equiv.) and B_2Pin_2 (114.3 mg, 0.45 mmol, 3.0 equiv.). The flask was evacuated and refilled with argon, followed by the addition of D_2O (9.0 μL, 0.45 mmol, 3.0 equiv.) and DMF (2 mL), and stirred at 80°C for given time. The resultant solution was diluted with ethyl acetate, washed with HCl aqueous solution (1 M) and concentrated in vacuum. The residue was detected by ^1H NMR analysis at given time with

CH_2Br_2 as the internal standard.

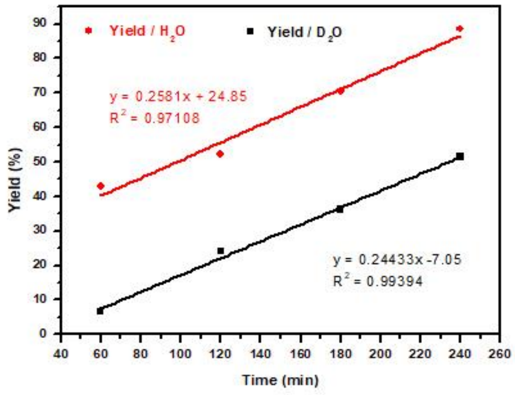

Supplementary Figure 2. The Kinetic Isotopic Effect of *E*-selective Hydrogenation of **1a**. a H_2O was replaced by D_2O in the *E*-selective reaction.

Unadjusted result of kinetic isotopic effect:

$$KIE' = k_H'/k_D' = 0.2581/0.24433 = 1.06$$

Calibrated result of kinetic isotopic effect:

The D/H ratios of deuterium-labeled alkenes were observed to be 70/30 in *E*-selective reactions.

$$(100\%\ k_H)/(70\%\ k_D+30\%\ k_H) = 0.2581/0.24433$$

$$KIE = k_H/k_D = 1.08$$

Calibrated result of kinetic isotopic effect in *Z*-selective reactant stream was calculated to be 1.68. The actual KIE value is estimated to be between 1.48 and 1.68 due to the ambiguous effects of water on deuterium labeling experiments. And it is obvious that no significant KIE is observed in either conditions.

2.5 Control Experiments on *Z-E* Isomerization

To a sealed tube were added **2a** (31.2 mg, 0.15 mmol), $NiBr_2$ (1.7 mg, 0.0075 mmol, 5 mol%), **L1** (2.6 mg, 0.0165 mmol, 11 mol%), CF_3CO_2Na (40.8 mg, 0.3 mmol, 2.0 equiv.) and B_2Pin_2 (114.3 mg, 0.45 mmol, 3.0 equiv.). The flask was evacuated and refilled with argon, followed by the addition of H_2O (8.1 μL, 0.45 mmol, 3.0 equiv.) and DMF (2 mL), and stirred at 80°C for 12 h. The resultant solution was diluted with ethyl acetate, washed with HCl aqueous solution (1 M) and concentrated in vacuum. The residue was detected by ^1H NMR analysis at given time with CH_2Br_2 as the internal standard (3% yield of **3a**).

To a sealed tube were added alkene **2a** (31.2 mg, 0.15 mmol), $NiBr_2$ (1.7 mg, 0.0075 mmol, 5 mol%), **L3** (3.6 mg, 0.0165 mmol, 11 mol%), $PhCO_2Na$ (21.6 mg, 0.15 mmol, 1.0 equiv.) and B_2Pin_2 (114.3 mg, 0.45 mmol, 3.0 equiv.). The flask was evacuated and refilled with argon, followed by the addition of H_2O (8.1 μL, 0.45 mmol, 3.0 equiv.) and DMF (2 mL), and stirred at 80 °C for 12 h. The resultant solution was diluted with ethyl acetate, washed with HCl aqueous solution (1 M) and concentrated in vacuum. The residue was detected by ^1H NMR analysis with CH_2Br_2 as the internal standard (4% yield of **3a**).

To a sealed tube were added alkene **2a** (31.2 mg, 0.15 mmol), $NiBr_2$ (1.7 mg, 0.0075 mmol, 5 mol%), **L3** (3.6 mg, 0.0165 mmol, 11 mol%), $PhCO_2Na$ (21.6 mg, 0.15 mmol, 1.0 equiv.) and B_2Pin_2 (114.3 mg, 0.45 mmol, 3.0 equiv.). The flask was evacuated and refilled with argon, followed by the addition of H_2O (8.1 μL, 0.45 mmol, 3.0 equiv.) and DMF (2 mL), and stirred at 120 °C for 12 h. The resultant solution was diluted with ethyl acetate, washed with HCl aqueous solution (1 M)

and concentrated in vacuum. The residue was detected by ^{1}H NMR analysis with CH_2Br_2 as the internal standard (13% yield of **3a**).

To a sealed tube were added alkyne **1a** (30.9 mg, 0.15 mmol), $NiBr_2$ (1.7 mg, 0.0075 mmol, 5 mol%), **L3** (3.6 mg, 0.0165 mmol, 11 mol%), $PhCO_2Na$ (21.6 mg, 0.15 mmol, 1.0 equiv.) and B_2Pin_2 (114.3 mg, 0.45 mmol, 3.0 equiv.). The flask was evacuated and refilled with argon, followed by the addition of H_2O (8.1 µL, 0.45 mmol, 3.0 equiv.) and DMF (2 mL), and stirred at 120 °C for 12 h. The resultant solution was diluted with ethyl acetate, washed with HCl aqueous solution (1 M) and concentrated in vacuum. The residue was detected by ^{1}H NMR analysis with CH_2Br_2 as the internal standard (87% yield, 86/14 *Z*/*E*).

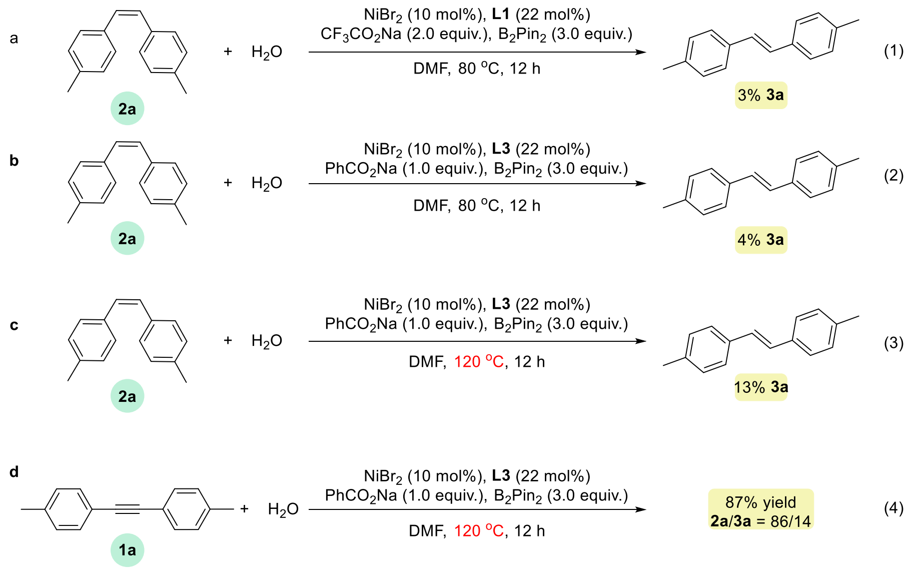

Supplementary Figure 3. Control Experiments on *Z-E* Isomerization. a and b *Z*-alkene **2a** was put in both standard conditions. c *Z*-alkene **2a** was put in *Z*-selective conditions of 120 °C. d Alkyne **1a** was put in *Z*-selective conditions of 120 °C.

2.6 The Color Change of the Two Hydrogenation Systems

To a sealed tube were added alkyne **1a** (30.9 mg, 0.15 mmol), $NiBr_2$ (3.3 mg, 0.015 mmol, 10 mol%), **L3** (7.1 mg, 0.033 mmol, 22 mol%), $PhCO_2Na$ (21.6 mg, 0.15 mmol, 1.0 equiv.) and B_2Pin_2 (114.3 mg, 0.45 mmol, 3.0 equiv.). The flask was evacuated and refilled with argon, followed by the addition of H_2O (8.1 µL, 0.45 mmol, 3.0 equiv.) and DMF (2 mL) and stirred at 80 °C. The color of the solution was recorded as follows.

To a sealed tube were added alkyne **1a** (30.9 mg, 0.15 mmol), $NiBr_2$ (3.3 mg, 0.015 mmol, 10 mol%), **L1** (5.2 mg, 0.033 mmol, 22 mol%), CF_3CO_2Na (30.8 mg, 0.3 mmol, 2.0 equiv.) and B_2Pin_2 (114.3 mg, 0.45 mmol, 3.0 equiv.). The flask was evacuated and refilled with argon, followed by the addition of H_2O (8.1 µL, 0.45 mmol, 3.0 equiv.) and DMF (2 mL) and stirred at 80 °C. The color of the solution was recorded as follows.

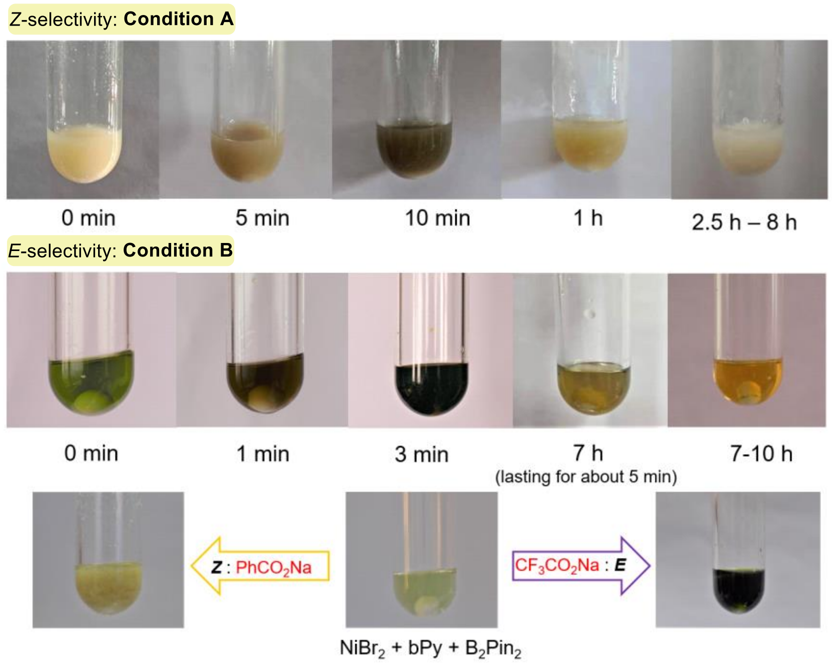

Supplementary Figure 4. The Color Change of the Two Hydrogenation Systems. a Condition A: **1**, $NiBr_2$ (10 mol%), **L3** (22 mol%), $PhCO_2Na$ (1.0 equiv.), B_2Pin_2 (3.0 equiv.), H_2O (3.0 equiv.), DMF, 80 °C in Ar atmosphere; b Condition B: **1**, $NiBr_2$ (10 mol%), **L1** (22 mol%), CF_3CO_2Na (2.0 equiv.), B_2Pin_2 (3.0 equiv.), H_2O (3.0 equiv.), DMF, 80 °C in Ar atmosphere.

2.7 Control Experiments on Base Effect

To a sealed tube were added alkyne **1a** (30.9 mg, 0.15 mmol), $NiBr_2$ (3.3 mg, 0.015 mmol, 10 mol%), **L3** (3.6 mg, 0.0165 mmol, 11 mol%), $PhCO_2Na$ (21.6 mg, 0.15 mmol, 1.0 equiv.) and B_2Pin_2 (114.3 mg, 0.45 mmol, 3.0 equiv.). The flask was evacuated and refilled with argon, followed by the addition of H_2O (8.1 μL, 0.45 mmol, 3.0 equiv.) and DMF (2 mL), and stirred at 80 °C for 1 h. CF_3CO_2Na (4.08 mg, 0.3 mmol, 2.0 equiv.) was added to the reaction mixture, which was stirred at 80 °C for another 10 h The resultant solution was diluted with ethyl acetate, washed with HCl aqueous solution (1 M) and concentrated in vacuum. The residue was detected by ^1H NMR analysis with CH_2Br_2 as the internal standard (88% conv., 78% yield, 96/4 *Z*/*E*).

To a sealed tube were added alkyne **1a** (30.9 mg, 0.15 mmol), $NiBr_2$ (3.3 mg, 0.015 mmol, 10 mol%), **L1** (5.2 mg, 0.033 mmol, 22 mol%), CF_3COONa (30.8 mg, 0.3 mmol, 2.0 equiv.) and B_2Pin_2 (114.3 mg, 0.45 mmol, 3.0 equiv.). The flask was evacuated and refilled with argon, followed by the addition of H_2O (8.1 μL, 0.45 mmol, 3.0 equiv.) and DMF (2 mL), and stirred at 80 °C for 1 h. PhCOONa (43.2mg, 0.3 mmol, 2.0 equiv.) was added to the reaction mixture, which was stirred at 80 °C for another 10 h. The resultant solution was diluted with ethyl acetate, washed with HCl aqueous solution (1 M) and concentrated in vacuum. The residue was detected by ^1H NMR analysis with CH_2Br_2 as the internal standard (>99% conv., 88% yield, 28/72 *Z*/*E*).

2.8. Mercury Poison Test

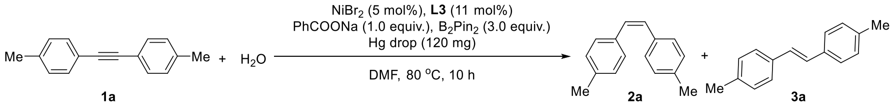

To a sealed tube were added alkyne **1a** (30.9 mg, 0.15 mmol), $NiBr_2$ (1.7 mg, 0.0075 mmol, 5 mol%), **L3** (3.6 mg, 0.0165 mmol, 11 mol%), $PhCO_2Na$ (21.6 mg, 0.15 mmol, 1.0 equiv.) and B_2Pin_2

(114.3 mg, 0.45 mmol, 3.0 equiv.). The flask was evacuated and refilled with argon, followed by the addition of H_2O (8.1 μL, 0.45 mmol, 3.0 equiv.), DMF (2 mL) and 1 drop of mercury (120 mg, 240 equiv), and stirred at 80 °C for 10 h. The resultant solution was diluted with ethyl acetate, washed with HCl aqueous solution (1 M) and concentrated in vacuum. The residue was detected by ^{1}H NMR analysis with CH_2Br_2 as the internal standard (83% yield, 92/8 *Z*/*E*).

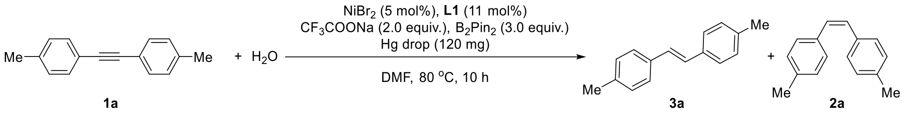

To a sealed tube were added alkyne **1a** (30.9 mg, 0.15 mmol), $NiBr_2$ (1.7 mg, 0.0075 mmol, 5 mol%), **L1** (2.6 mg, 0.0165 mmol, 11 mol%), CF_3COONa (40.8 mg, 0.3 mmol, 2.0 equiv.) and B_2Pin_2 (114.3 mg, 0.45 mmol, 3.0 equiv.). The flask was evacuated and refilled with argon, followed by the addition of H_2O (8.1 μL, 0.45 mmol, 3.0 equiv.), DMF (2 mL) and 1 drop of mercury (120 mg, 240 equiv), and stirred at 80 °C for 10 h. The resultant solution was diluted with ethyl acetate, washed with HCl aqueous solution (1 M) and concentrated in vacuum. The residue was detected by ^{1}H NMR analysis with CH_2Br_2 as the internal standard (84% yield, 92/8 *E*/*Z*).

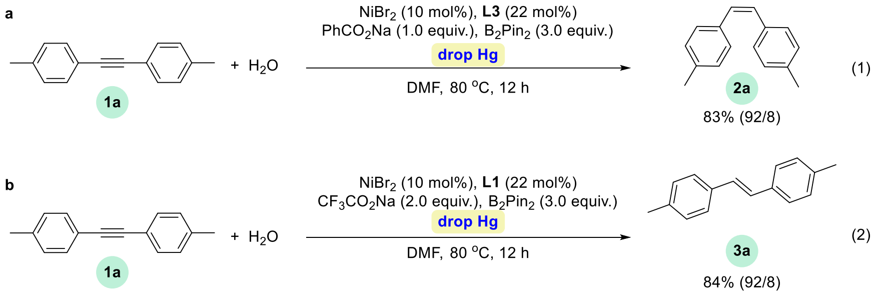

Supplementary Figure 5. Mercury Poison Test. a Mercury poison test in *Z*-selective reaction. b Mercury poison test in *E*-selective catalytic system.

2.9 EPR Experiments.

Condition A: To a sealed tube were added alkyne **1a** (30.9 mg, 0.15 mmol), $NiBr_2$ (3.3 mg, 0.015 mmol, 10 mol%), **L3** (7.1 mg, 0.033 mmol, 22 mol%), $PhCO_2Na$ (21.6 mg, 0.15 mmol, 1.0 equiv.) and B_2Pin_2 (114.3 mg, 0.45 mmol, 3.0 equiv.). The flask was evacuated and refilled with argon, followed by the addition of H_2O (8.1 μL, 0.45 mmol, 3.0 equiv.) and DMF (2 mL), and stirred at rt for 5 min. 10 μL of the resultant mixture was taken out into a small tube in glovebox and analyzed by EPR (353 K) after 10 min, 20 min and 30 min, respectively.

Condition B: To a sealed tube were added alkyne **1a** (30.9 mg, 0.15 mmol), $NiBr_2$ (3.3 mg, 0.015 mmol, 10 mol%), **L1** (5.2 mg, 0.033 mmol, 22 mol%), CF_3CO_2Na (30.8 mg, 0.3 mmol, 2.0 equiv.) and B_2Pin_2 (114.3 mg, 0.45 mmol, 3.0 equiv.). The flask was evacuated and refilled with argon, followed by the addition of H_2O (8.1 μL, 0.45 mmol, 3.0 equiv.) and DMF (2 mL), and stirred at rt for 5 min. 10 μL of the resultant mixture was taken out into a small tube in glovebox and analyzed by EPR (353 K) after 10 min, 20 min and 30 min, respectively.

Condition B with $PhCO_2Na$: To a sealed tube were added alkyne **1a** (30.9 mg, 0.15 mmol), $NiBr_2$ (3.3 mg, 0.015 mmol, 10 mol%), **L1** (5.2 mg, 0.033 mmol, 22 mol%), CF_3CO_2Na (30.8 mg, 0.3 mmol, 2.0 equiv.) and B_2Pin_2 (114.3 mg, 0.45 mmol, 3.0 equiv.). The flask was evacuated and refilled with argon, followed by the addition of H_2O (8.1 μL, 0.45 mmol, 3.0 equiv.) and DMF (2 mL), and stirred at 80 °C for 1 h. 10 μL of the resultant mixture was taken out into a small tube in glovebox and analyzed by EPR (353 K). $PhCO_2Na$ (43.2 mg, 0.3 mmol, 2.0 equiv.) was added to

the remained mixture, which was stirred at 80 °C for another 10 min. 10 μL of this solution sample was taken out into another small tube in glovebox, and analyzed by EPR (353 K) at 10 min, 15 min, 30 min and 45 min, respectively.

Condition C: To a sealed tube were added alkyne **1a** (30.9 mg, 0.15 mmol), $NiBr_2$ (3.3 mg, 0.015 mmol, 10 mol%), **L1** (5.2 mg, 0.033 mmol, 22 mol%), HCO_2Na (20.4 mg, 0.3 mmol, 2.0 equiv.) and B_2Pin_2 (114.3 mg, 0.45 mmol, 3.0 equiv.). The flask was evacuated and refilled with argon, followed by the addition of H_2O (8.1 μL, 0.45 mmol, 3.0 equiv.) and DMF (2 mL), and stirred at rt for 5 min. 10 μL of the resultant mixture was taken out into a small tube in glovebox and analyzed by EPR (353 K) after 10 min.

Condition A without alkyne 1a and water: To a sealed tube were added $NiBr_2$ (3.3 mg, 0.015 mmol), **L3** (7.1 mg, 0.033 mmol), $PhCO_2Na$ (21.6 mg, 0.15 mmol,) and B_2Pin_2 (114.3 mg, 0.45 mmol). The flask was evacuated and refilled with argon, followed by DMF (2 mL), and stirred at rt for 5 min. 10 μL of the resultant mixture was taken out into a small tube in glovebox and analyzed by EPR (353 K) after 10 min.

Condition B without alkyne 1a and water: To a sealed tube were added $NiBr_2$ (3.3 mg, 0.015 mmol), **L1** (5.2 mg, 0.033 mmol), CF_3CO_2Na (30.8 mg, 0.3 mmol) and B_2Pin_2 (114.3 mg, 0.45 mmol). The flask was evacuated and refilled with argon, followed by the addition of DMF (2 mL), and stirred at rt for 5 min. 10 μL of the resultant mixture was taken out into a small tube in glovebox and analyzed by EPR (353 K) after 10 min.

In situ generation of Ni(I) species[7-12]: To a sealed tube were added $NiBr_2$ (1.6 mg, 0.0075 mmol), $Ni(COD)_2$ (2.1 mg, 0.0075 mmol), **L1** (5.2 mg, 0.033 mmol) and B_2Pin_2 (114.3 mg, 0.45 mmol, 3.0 equiv.). The flask was evacuated and refilled with argon, followed by the addition of DMF (2 mL), and stirred at rt for 5 min. 10 μL of the resultant mixture was taken out into a small tube in glovebox and analyzed by EPR (353 K) after 10 min.

2.10 Control Experiments on Ni(I) Species

To a sealed tube were added $NiBr_2$ (1.6 mg, 0.0075 mmol, 5 mol%), $Ni(COD)_2$ (2.1 mg, 0.0075 mmol, 5 mol%) and **L1** (5.2 mg, 0.033 mmol, 22 mol%). The flask was evacuated and refilled with argon, followed by DMF (1 mL), and stirred at room temperature for 2 h. After that, alkyne **1a** (30.9 mg, 0.15 mmol), H_2O (8.1 μL, 0.45 mmol, 3.0 equiv.) and B_2Pin_2 (114.3 mg, 0.45 mmol, 3.0 equiv.) were added into this sealing tube in glove box. This tube was heated in a preheated oil bath at 80 °C for 10 h. The resultant solution was diluted with ethyl acetate, washed with aqueous HCl solution (1 M) and concentrated in vacuum. The residue was detected by ^1H NMR analysis with CH_2Br_2 as the internal standard (63% conv., 40% yield, 18/82 *Z*/*E*).

2.11 Investigations on Adding Reductants in Condition A

To a sealed tube were added alkyne **1a** (30.9 mg, 0.15 mmol), $NiBr_2$ (3.3 mg, 0.015 mmol, 10 mol%), **L3** (7.1 mg, 0.033 mmol, 22 mol%), $PhCO_2Na$ (21.6 mg, 0.15 mmol, 1.0 equiv.), Zn (9.6 mg, 0.15 mmol, 1.0 equiv.) and B_2Pin_2 (114.3 mg, 0.45 mmol, 3.0 equiv.). The flask was evacuated and refilled with argon, followed by the addition of H_2O (8.1 μL, 0.45 mmol, 3.0 equiv.) and DMF (2 mL) and stirred at 80 °C for 12 h. The resultant solution was diluted with ethyl acetate, washed with HCl aqueous solution (1 M) and concentrated in vacuum. The residue was detected by ^1H NMR analysis with CH_2Br_2 as the internal standard (82% yield, 78/22 *Z*/*E*).

To a sealed tube were added alkyne **1a** (30.9 mg, 0.15 mmol), $NiBr_2$ (3.3 mg, 0.015 mmol, 10 mol%), **L3** (7.1 mg, 0.033 mmol, 22 mol%), $PhCO_2Na$ (21.6 mg, 0.15 mmol, 1.0 equiv.), Mn (8.3

mg, 0.15 mmol, 1.0 equiv.) and B_2Pin_2 (114.3 mg, 0.45 mmol, 3.0 equiv.). The flask was evacuated and refilled with argon, followed by the addition of H_2O (8.1 µL, 0.45 mmol, 3.0 equiv.) and DMF (2 mL), and stirred at 80 °C for 12 h. The resultant solution was diluted with ethyl acetate, washed with HCl aqueous solution (1 M) and concentrated in vacuum. The residue was detected by ^{1}H NMR analysis with CH_2Br_2 as the internal standard (55% yield, 69/31 *Z*/*E*).

2.12 Control Experiments with Reductants Instead of B_2Pin_2 in Condition A

To a sealed tube were added alkyne **1a** (30.9 mg, 0.15 mmol), $NiBr_2$ (3.3 mg, 0.015 mmol, 10 mol%), **L3** (7.1 mg, 0.033 mmol, 22 mol%), $PhCO_2Na$ (21.6 mg, 0.15 mmol, 1.0 equiv.) and Zn (28.8 mg, 0.45 mmol, 3.0 equiv.). The flask was evacuated and refilled with argon, followed by the addition of H_2O (8.1 µL, 0.45 mmol, 3.0 equiv.) and DMF (2 mL), and stirred at 80 °C for 12 h. The resultant solution was diluted with ethyl acetate, washed with aqueous HCl solution (1 M) and concentrated in vacuum. The residue was detected by ^{1}H NMR analysis with CH_2Br_2 as the internal standard (Not react).

To a sealed tube were added alkyne **1a** (30.9 mg, 0.15 mmol), $NiBr_2$ (3.3 mg, 0.015 mmol, 10 mol%), **L3** (7.1 mg, 0.033 mmol, 22 mol%), $PhCO_2Na$ (21.6 mg, 0.15 mmol, 1.0 equiv.) and Mn (24.7 mg, 0.45 mmol, 3.0 equiv.). The flask was evacuated and refilled with argon, followed by the addition of H_2O (8.1 µL, 0.45 mmol, 3.0 equiv.) and DMF (2 mL), and stirred at 80 °C for 12 h. The resultant solution was diluted with ethyl acetate, washed with aqueous HCl solution (1 M) and concentrated in vacuum. The residue was detected by ^{1}H NMR analysis with CH_2Br_2 as the internal standard (Not react).

2.13 In Situ ^{1}H NMR Analysis of the Reaction System

As shown in supplementary Figure 4 and supplementary Figure 5, in situ NMR was monitored at 80 °C. The *Z*-selective system with $PhCO_2Na$ as base displayed a new peak at the chemical shift of 4.69 ppm, which still remains in the absence of water and alkyne **1a**. This new peak might be assigned to the methyl of Bpin in the [PhCOONi(II)BpinL] **C** species, since the NMR chemical shifts of protons in the metal complex might move to the low-field area.[13] Meanwhile, a new peak at 3.33 ppm in the *E*-selective system using CF_3CO_2Na as base was observed, which did not disappear in the absence of water and **1a**. This new peak may be the signal of [LNi(I)Bpin] **J** species in the *E*-selective catalytic cycle. A similar signal (3.27 ppm) was found in the in-situ NMR of the mixture of $NiBr_2$, $Ni(cod)_2$ and B_2Pin_2, further supporting our speculation. A faint peak in the chemical shift of 4.69 ppm was observed in the close-up of 4 ppm to 6 ppm (line 4 and 5), which is probably the hydrogen signal of [CF_3COONi(II)BpinL] **H** species corresponded with [PhCOONi(II)BpinL] **C**.

In situ ^{1}H NMR reactions of *Z*-selective catalytic cycles: a NMR tube was charged with alkyne **1a** (9.3 mg, 0.045 mmol), $NiBr_2$ (1 mg, 0.0045 mmol, 10 mol%), **L3** (2.1 mg, 0.0099 mmol, 22 mol%), $PhCO_2Na$ (6.5 mg, 0.045 mmol, 1.0 equiv.), B_2Pin_2 (34.3 mg, 0.135 mmol, 3.0 equiv.), H_2O (2.5 µL, 0.135 mmol, 3.0 equiv.) and DMF-*d7* (0.5 ml) in glovebox. The mixture was dispersed by ultrasonic dispersion and analyzed by NMR at 80 °C.

In situ ^{1}H NMR reactions of *E*-selective catalytic cycles: a NMR tube was charged with alkyne **1a** (9.3 mg, 0.045 mmol), $NiBr_2$ (1 mg, 0.0045 mmol, 10 mol%), **L1** (1.5 mg, 0.0099 mmol, 22 mol%), CF_3CO_2Na (12.2 mg, 0.09 mmol, 2.0 equiv.), B_2Pin_2 (34.3 mg, 0.135 mmol, 3.0 equiv.), H_2O (2.5 µL, 0.135 mmol, 3.0 equiv.) and DMF-*d7* (0.5 ml) in glovebox. The mixture was dispersed by ultrasonic dispersion and analyzed by NMR at 80 °C.

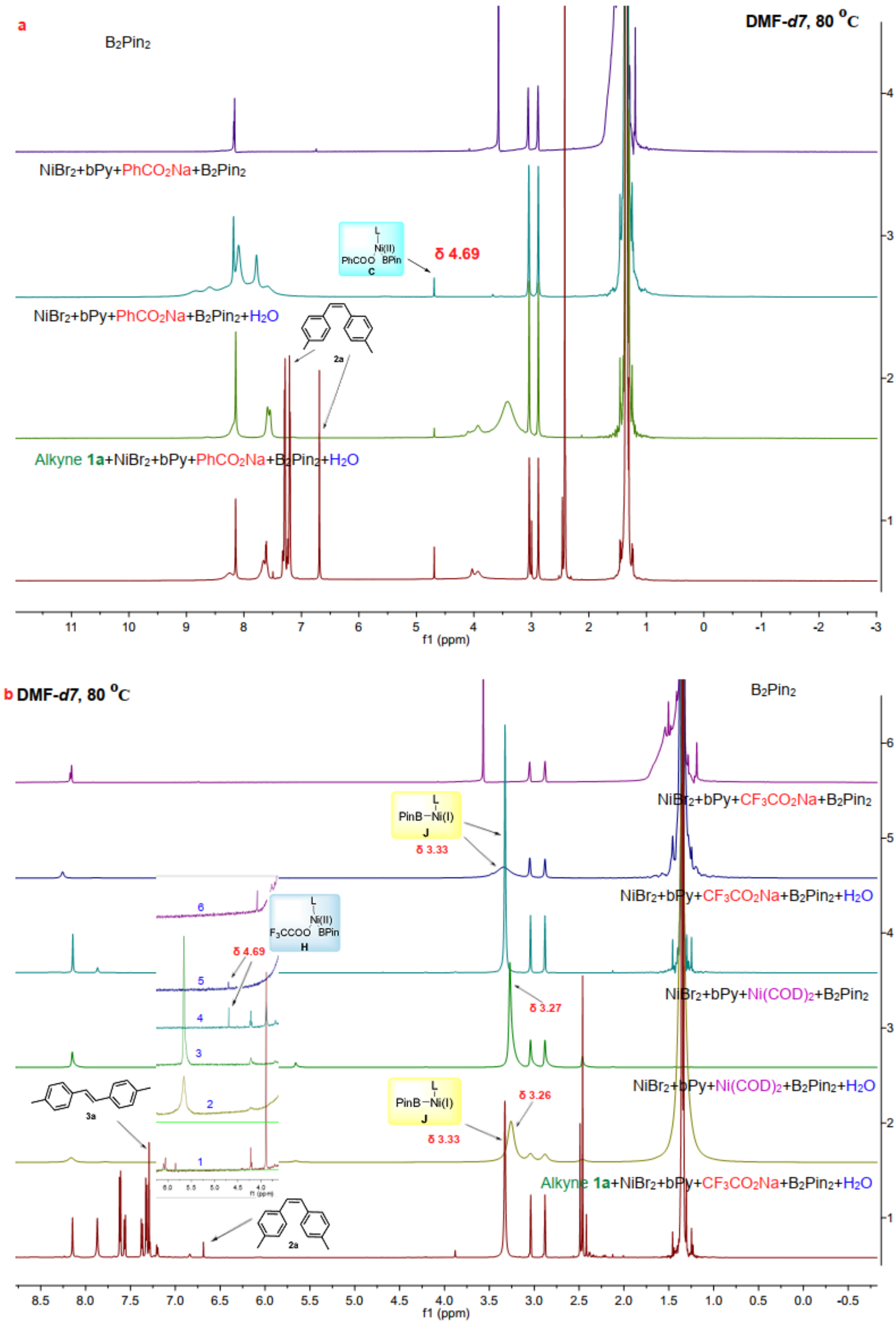

Supplementary Figure 6. *In Situ* ^{1}H NMR Analysis. a *In Situ* ^{1}H NMR Analysis of the *Z*-selective Reaction System. b *In Situ* ^{1}H NMR Analysis of the *E*-selective Reaction System.

2.14 Density functional theory (DFT) calculations

To further understand the mechanism of the reaction, especially the remarkable different

impact of $PhCO_2Na$ and CF_3CO_2Na on the variation of Ni species, density functional theory (DFT) calculations were carried out with Gaussian 09 software package.[14] The geometry optimization and frequency calculations were carried out with B3LYP-D3 method and combined basis sets.[15-17] That is, 6-31G(d) for nonmetallic atoms and SDD for Ni atom.[18,19] Truhlar and coworkers' SMD solvation model was employed to consider the solvent effect of DMF (ε=8.93).[20] The geometry optimizations were performed without symmetry constraints and the nature of the extrema was checked by analytical frequency calculations. The intrinsic reaction coordinate (IRC) calculations[21,22] was also performed to verify the connectivity of the transition state and the energy minima.

2.14.1 Computations for *Z*-selective Hydrogenation

The free-energy profiles of generation of Ni(I) in the reaction with $PhCO_2Na$ as the base are shown in Supplementary Figure 6. The complex of nickel bromide and 2,2'-bipyridine **A** reacted with $PhCO_2Na$ to afford nickel benzoate **B**, which is exergonic by 44.7 kcal/mol. The activation free energy barrier for one-ligand exchange of **B** towards PhCOO-Ni(II)-BPin **C** is 25.2 kcal/mol. The transition state for further ligand exchange of **C** with B_2pin_2 towards BPin -Ni(II)-BPin **P** could not be located, which is favored thermodynamically. Subsequent reductive elimination and comproportionation process could deliver thermodynamically more stable Ni(I) species **J** or **Q**. However, the transition state for the subsequent comproportionation process to **J** or **J**+**Q** was not successfully located, which might be too high to proceed due to the intersystem crossing. Corresponding computational study will be further explored in our laboratory.

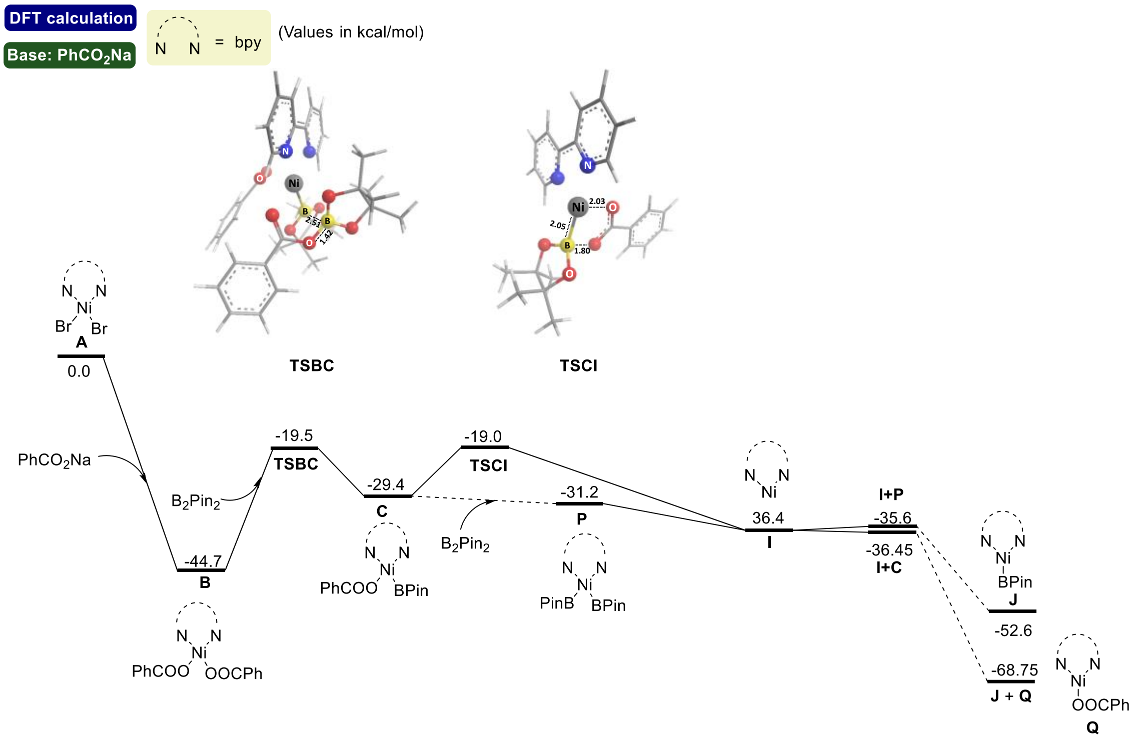

Supplementary Figure 7. *Z*-selective hydrogenation free-energy profiles. a The free-energy profiles of generation of Ni(I) in the *Z*-selective reaction.

2.14.2 Computations for Isomerization

In order to explain the selectivity of the two hydrogenation systems, we have located the free energy reaction profiles of the two reactions, and compared the corresponding reaction pathways. The *cis*/*trans* isomerization of vinyl Ni(I) intermediate **M** was found to proceed via

a three-membered ring transition state[23] (Supplementary Figure 7), with an activation barrier of 19.3 kcal/mol. As expected, the trans isomer **N** is 3.4 kcal/mol more stable than **M**. However, transformation from **E** to **S** turned out to be also achievable, although the energy barrier is higher than the isomerization of **M-N**, which might make it more predisposed to undergo hydrolysis directly once the vinyl nickel is formed. We also tried to calculate other possible process including direct trans-addition of Ni-H species to alkenes as well as isomerization via carbene nickel intermediates,[23, 24] but failed to locate the transition states. Taking consideration of the computational results, the supportive mechanistic experiments and also existed reports on isomerization of alkenyl Ni(I) species,[25-27] the proposed catalytic cycles are still the most reasonable pathways.

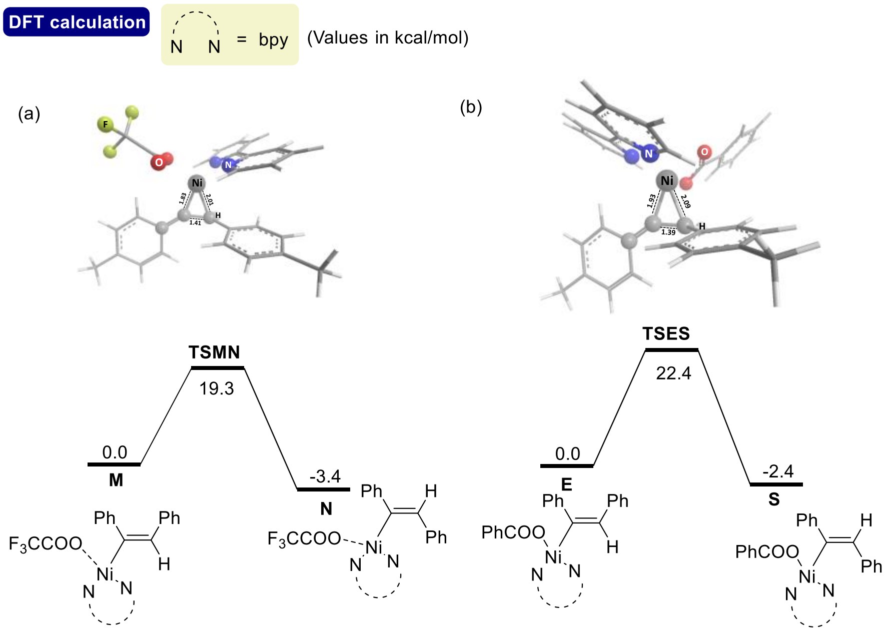

Supplementary Figure 8. Calculations for isomerization. a The *cis/trans* isomerization of vinyl Ni(I) intermediate. b The *cis/trans* isomerization of vinyl Ni(II) intermediate.

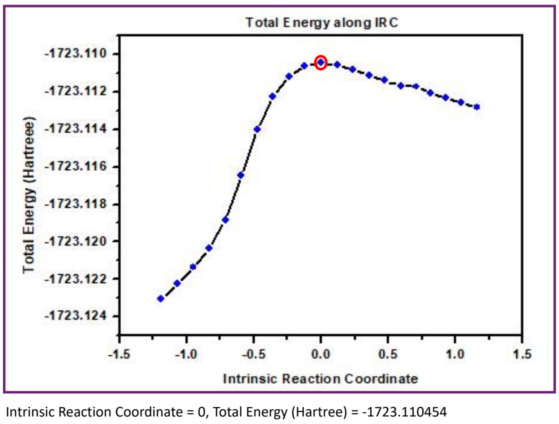

Supplementary Figure 9. IRC graph of **TSCD**. a The intrinsic reaction coordinate (IRC) calculation was performed to verify the connectivity of the transition state and the energy minima.

2.15 Synthesis of Alkynes 1

Substrates **1g**, **1nn** and **1qq** are commercially available and used without further purification. Substrates **1a-1f**, **1h-1kk**, **1mm** and **1nn** were synthesized according to published procedures. The

spectral data of the substrates were consisted with that reported in the literature (**1a-1c**, **1i**[28]; **1d**, **1l-1o**, **1k**, **1u**, **1jj**,[29]; **1e**, **1p**[30]; **1f**, **1q-1s**, **1y**, **1bb**, **1ff**[31]; **1h**, **1j**[32]; **1t**, **1w**, **1z**, **1hh**[33]; **1v**[34], **1x**[35]; **1aa**[36]; **1cc**[37]; **1dd**[38]; **1ee**, **1ii**[39]; **1kk**[40]; **1ll**[41]; **1mm**[42]; **1oo**[43]; **1pp**[44]).

Synthesis of 1gg

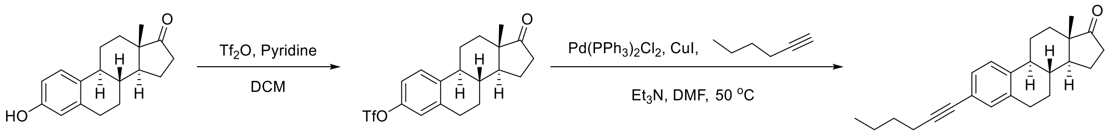

1gg, 465.6 mg

The preparations of **1gg** was performed according to a literature reference.[45] To a solution of (8*R*,9*S*,13*S*,14*S*)-3-hydroxy-13-methyl-6,7,8,9,11,12,13,14,15,16-decahydro-17*H*-cyclopenta[*a*]phenanthren-17-one (1.35 g, 5 mmol) in DCM (20 mL) was added pyridine (0.8 mL, 10 mmol). Tf_2O (1 mL, 6 mmol, diluted with 20 mL DCM) was added dropwise to the mixture at 0 °C. The resulting solution was warmed up to room temperature and stirred for 2 h. Then the mixture was quenched with saturated NH_4Cl solution and extracted with dichloromethane. The combined organic extracts were washed with water and brine, dried over anhydrous Na_2SO_4 and concentrated in vacuo. The crude product was purified by flash column chromatography on silica gel to give the (8*R*,9*S*,13*S*,14*S*)-13-methyl-17-oxo-7,8,9,11,12,13,14,15,16,17-decahydro-6*H*-cyclopenta[*a*]phenanthren-3-yl trifluoromethanesulfonate as a white solid.

To a solution of the above compound (1.006 g, 2.5 mmol) in triethylamine (1.9 mL) and DMF (10 mL) were added hex-1-yne (254.7 mg, 3.1 mmol), $Pd(PPh_3)_2Cl_2$ (52.6 mg, 0.075 mmol) and CuI (14.3 mg, 0.075 mmol) at room temperature. The mixture was stirred at 50 °C for 12 h under Ar. After the starting material was consumed as monitored by TLC, the reaction mixture was quenched with saturated NH_4Cl solution and extracted with dichloromethane. The combined organic extracts were washed with water and brine, dried over anhydrous Na_2SO_4 and concentrated in vacuo. The crude product was purified by flash column chromatography on silica gel to give the (8*R*,9*S*,13*S*,14*S*)-3-(hex-1-yn-1-yl)-13-methyl-6,7,8,9,11,12,13,14,15,16-decahydro-17*H*-cyclopenta[*a*]phenanthren-17-one **1gg** as a white solid (465.6 mg, 58% yield). ^{1}H NMR (400 MHz, $CDCl_3$) δ 7.16 (d, *J* = 16.9 Hz, 3 H), 2.86 (d, *J* = 4.6 Hz, 2 H), 2.50 (dd, *J* = 18.7, 8.6 Hz, 1 H), 2.42 – 2.36 (m, 3 H), 2.28 (d, *J* = 8.7 Hz, 1 H), 2.15 (dd, *J* = 18.4, 9.1 Hz, 1 H), 2.07 (dd, *J* = 13.1, 7.6 Hz, 1 H), 2.03 – 1.93 (m, 2 H), 1.59 (ddd, *J* = 19.5, 17.4, 8.4 Hz, 5 H), 1.52 – 1.36 (m, 5 H), 0.94 (dd, *J* = 15.4, 8.2 Hz, 6 H). ^{13}C NMR (101 MHz, $CDCl_3$) δ 220.48, 139.28, 136.33, 131.95, 128.84, 125.15, 121.46, 89.60, 80.47, 50.53, 47.90, 44.38, 38.04, 35.79, 31.57, 30.90, 29.05, 26.36, 25.59, 21.96, 21.55, 19.08, 13.81, 13.59. HRMS (EI) Calcd. for $C_{24}H_{30}O^+$: 334.2291, Found: 334.2293.

2.16 General Procedure for *Z*-Selective Transfer Semihydrogenation of Alkynes 1

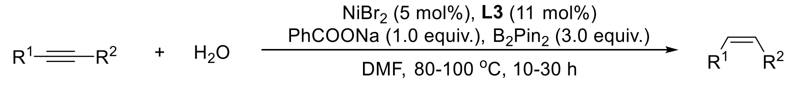

1 **2**

To a dry sealed tube were added alkyne **1** (0.3 mmol), $NiBr_2$ (3.3 mg, 0.015 mmol, 5 mol%), **L3** (7.1 mg, 0.033 mmol, 11 mol%), $PhCO_2Na$ (43.2 mg, 0.3 mmol, 1.0 equiv.) and B_2Pin_2 (228.5 mg, 0.9 mmol, 3.0 equiv.). The flask was evacuated and refilled with argon, followed by the addition of H_2O (16.2 μL, 0.9 mmol, 3.0 equiv.) and DMF (4 mL). The mixture was stirred at 80-100°C for 8-30 h until the reaction was completed as monitored by TLC. The resultant solution was diluted with ethyl acetate, washed with HCl aqueous solution (1 M) (the reaction of **1k**, **1z** was washed with water) and concentrated in vacuum. The mixture was detected by GC directly or after simple

filtration in some cases to determine the *Z*/*E* ratio (GC analysis was acquired on SHIMADZU GC-2030AF gas chromatograph fitted with SH-RTX-5 column (30 m length × 250 µm × 0.25 µm) using the following method: SHIMADZU GC-2030AF GC system: FID starting temp: 80 °C, Time at starting temp: 2 min, Ramp: 27.5 °C/min up to 300 °C with hold time = 10 min, Flow rate (carrier): 1.62 mL/min (N_2), Split ratio: 39, inlet temperature: 300 °C, detector temperature: 300 °C). The crude product was purified by chromatography on silica gel (300-400 mesh), eluted with petroleum ether with 0-20 % of ethyl acetate to give alkene product. Extremely careful column chromatography was able to partially deliver the major product in a pure form to provide precise NMR spectra of the major product. The overall isolated yield was calculated based on the combination of all parts.

Characterization Data of *Z*-alkenes

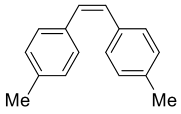

2a

(*Z*)-1,2-di-p-tolylethene. According to the general procedure, the sealed tube was charged with the corresponding alkyne (0.3 mmol, 1.0 equiv.), $NiBr_2$ (3.3 mg, 0.015 mmol, 5 mol%), **L3** (7.1 mg, 0.033 mmol, 11 mol%), $PhCO_2Na$ (43.2 mg, 0.3 mmol, 1.0 equiv.), B_2Pin_2 (228.5 mg, 0.9 mmol, 3.0 equiv.), H_2O (16.2 µL, 0.9 mmol, 3.0 equiv.) and DMF (4 mL). The reaction was carried out under Ar at 80 °C for 12 h. Flash column chromatography (petroleum ether) afforded the product **2a** as a white solid (56.2 mg, 90%, 94/6 *Z/E*). ^1H NMR (400 MHz, $CDCl_3$) δ 7.21 (d, *J* = 8.0 Hz, 4 H), 7.08 (d, *J* = 7.9 Hz, 4 H), 6.56 (s, 2 H), 2.36 (s, 6 H). ^{13}C NMR (101 MHz, $CDCl_3$) δ 136.68, 134.49, 129.50, 128.86, 128.73, 21.20. The spectroscopic data correspond to reported data.[46]

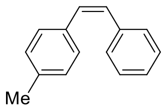

2b

(*Z*)-1-methyl-4-styrylbenzene. According to the general procedure, the sealed tube was charged with the corresponding alkyne (0.3 mmol, 1.0 equiv.), $NiBr_2$ (3.3 mg, 0.015 mmol, 5 mol%), **L3** (7.1 mg, 0.033 mmol, 11 mol%), $PhCO_2Na$ (43.2 mg, 0.3 mmol, 1.0 equiv.), B_2Pin_2 (228.5 mg, 0.9 mmol, 3.0 equiv.), H_2O (16.2 µL, 0.9 mmol, 3.0 equiv.) and DMF (4 mL). The reaction was carried out under Ar at 80 °C for 12 h. Flash column chromatography (petroleum ether) afforded the product **2b** as a white solid (51.9 mg, 89%, 96/4 *Z/E*). ^1H NMR (400 MHz, $CDCl_3$) δ 7.25 (d, *J* = 7.0 Hz, 2 H), 7.19 (dt, *J* = 8.4, 6.8 Hz, 3 H), 7.13 (d, *J* = 8.0 Hz, 2 H), 7.01 (d, *J* = 7.9 Hz, 2 H), 6.59 – 6.49 (m, 2 H), 2.29 (s, 3 H). ^{13}C NMR (101 MHz, $CDCl_3$) δ 137.48, 136.83, 134.25, 130.18, 129.54, 128.88, 128.82, 128.77, 128.16, 126.94, 21.20. The spectroscopic data correspond to reported data.[46]

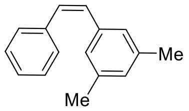

2c

(*Z*)-1,3-dimethyl-5-styrylbenzene. According to the general procedure, the sealed tube was charged with the corresponding alkyne (0.3 mmol, 1.0 equiv.), $NiBr_2$ (3.3 mg, 0.015 mmol, 5 mol%), **L3** (7.1 mg, 0.033 mmol, 11 mol%), $PhCO_2Na$ (43.2 mg, 0.3 mmol, 1.0 equiv.), B_2Pin_2 (228.5 mg, 0.9 mmol, 3.0 equiv.), H_2O (16.2 µL, 0.9 mmol, 3.0 equiv.) and DMF (4 mL). The reaction was

carried out under Ar at 80 °C for 12 h. Flash column chromatography (petroleum ether) afforded the product **2c** as a white solid (58.1 mg, 93%, 93/7 *Z/E*). ^{1}H NMR (400 MHz, $CDCl_3$) δ 7.25 (d, *J* = 6.7 Hz, 2 H), 7.19 (td, *J* = 8.6, 4.3 Hz, 3 H), 6.87 (s, 2 H), 6.82 (s, 1H), 6.53 (s, 2 H), 2.20 (s, 6 H). ^{13}C NMR (101 MHz, $CDCl_3$) δ 137.58, 137.36, 137.12, 130.43, 129.85, 128.85, 128.74, 128.06, 126.98, 126.57, 21.17. The spectroscopic data correspond to reported data.[28]

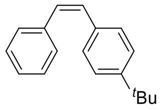

2d

(*Z*)-1-(*tert*-butyl)-4-styrylbenzene. According to the general procedure, the sealed tube was charged with the corresponding alkyne (0.3 mmol, 1.0 equiv.), $NiBr_2$ (3.3 mg, 0.015 mmol, 5 mol%), **L3** (7.1 mg, 0.033 mmol, 11 mol%), $PhCO_2Na$ (43.2 mg, 0.3 mmol, 1.0 equiv.), B_2Pin_2 (228.5 mg, 0.9 mmol, 3.0 equiv.), H_2O (16.2 µL, 0.9 mmol, 3.0 equiv.) and DMF (4 mL). The reaction was carried out under Ar at 80 °C for 12 h. Flash column chromatography (petroleum ether) afforded the product **2d** as a white solid (69.5 mg, 98%, 93/7 *Z/E*). ^{1}H NMR (400 MHz, $CDCl_3$) δ 7.29 (d, *J* = 6.9 Hz, 2 H), 7.26-7.14 (m, 7 H), 6.55 (s, 2 H), 1.29 (s, 9 H). ^{13}C NMR (101 MHz, $CDCl_3$) δ 150.15, 137.60, 134.18, 130.08, 129.56, 128.80, 128.57, 128.19, 126.94, 125.06, 34.53, 31.27. The spectroscopic data correspond to reported data.[29]

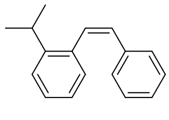

2e

(*Z*)-1-isopropyl-2-styrylbenzene. According to the general procedure, the sealed tube was charged with the corresponding alkyne (0.3 mmol, 1.0 equiv.), $NiBr_2$ (3.3 mg, 0.015 mmol, 5 mol%), **L3** (7.1 mg, 0.033 mmol, 11 mol%), $PhCO_2Na$ (43.2 mg, 0.3 mmol, 1.0 equiv.), B_2Pin_2 (228.5 mg, 0.9 mmol, 3.0 equiv.), H_2O (16.2 µL, 0.9 mmol, 3.0 equiv.) and DMF (4 mL). The reaction was carried out under Ar at 80 °C for 12 h. Flash column chromatography (petroleum ether) afforded the product **2e** as a colorless oil (62.1 mg, 94%, 99/1 *Z/E*). ^{1}H NMR (400 MHz, $CDCl_3$) δ 7.32 (d, *J* = 7.7 Hz, 1H), 7.24 (t, *J* = 7.4 Hz, 1H), 7.19-6.99 (m, 7 H), 6.76 (d, *J* = 12.2 Hz, 1 H), 6.60 (d, *J* = 12.2 Hz, 1 H), 3.20 (dt, *J* = 13.7, 6.9 Hz, 1 H), 1.21 (d, *J* = 6.9 Hz, 6 H). ^{13}C NMR (101 MHz, $CDCl_3$) δ 146.60, 136.85, 136.18, 130.54, 129.65, 129.27, 129.08, 128.01, 127.61, 126.95, 125.68, 125.10, 30.18, 23.32. HRMS (EI) Calcd. for $C_{17}H_{18}^+$: 222.1403, Found: 222.1405.

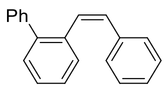

2f

(*Z*)-2-styryl-1,1'-biphenyl. According to the general procedure, the sealed tube was charged with the corresponding alkyne (0.3 mmol, 1.0 equiv.), $NiBr_2$ (3.3 mg, 0.015 mmol, 5 mol%), **L3** (7.1 mg, 0.033 mmol, 11 mol%), $PhCO_2Na$ (43.2 mg, 0.3 mmol, 1.0 equiv.), B_2Pin_2 (228.5 mg, 0.9 mmol, 3.0 equiv.), H_2O (16.2 µL, 0.9 mmol, 3.0 equiv.) and DMF (4 mL). The reaction was carried out under Ar at 80 °C for 12 h. Flash column chromatography (petroleum ether) afforded the product **2f** as a colorless oil (68.5 mg, 89%, 97/3 *Z/E*). ^{1}H NMR (400 MHz, $CDCl_3$) δ 7.43 (d, *J* = 6.9 Hz, 2 H), 7.40-7.33 (m, 3 H), 7.30 (dd, *J* = 12.4, 5.5 Hz, 3 H), 7.25 (d, *J* = 6.9 Hz, 2 H), 7.21-7.10 (m, 4 H), 6.50 (d, *J* = 12.2 Hz, 1 H), 6.41 (d, *J* = 12.2 Hz, 1 H). ^{13}C NMR (101 MHz, $CDCl_3$) δ 141.18, 141.03,

137.02, 135.76, 130.48, 129.99, 129.91, 129.89, 129.43, 128.93, 128.14, 127.98, 127.46, 127.06, 127.00. The spectroscopic data correspond to reported data.[47]

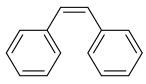

2g

(*Z*)-1,2-diphenylethene. According to the general procedure, the sealed tube was charged with the corresponding alkyne (0.3 mmol, 1.0 equiv), $NiBr_2$ (3.3 mg, 0.015 mmol, 5 mol%), **L3** (7.1 mg, 0.033 mmol, 11 mol%), $PhCO_2Na$ (43.2 mg, 0.3 mmol, 1.0 equiv.), B_2Pin_2 (228.5 mg, 0.9 mmol, 3.0 equiv.), H_2O (16.2 µL, 0.9 mmol, 3.0 equiv.) and DMF (4 mL). The reaction was carried out under Ar at 80 °C for 12 h. Flash column chromatography (petroleum ether) afforded the product **2g** as a colorless oil (44.3 mg, 82%, 97/3 *Z/E*). ^1H NMR (400 MHz, $CDCl_3$) δ 7.29-7.14 (m, 10 H), 6.59 (s, 2 H). ^{13}C NMR (101 MHz, $CDCl_3$) δ 137.23, 130.23, 128.85, 128.18, 127.06. The spectroscopic data correspond to reported data.[46]

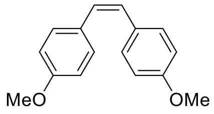

2h

(*Z*)-1,2-bis(4-methoxyphenyl)ethene. According to the general procedure, the sealed tube was charged with the corresponding alkyne (0.3 mmol, 1.0 equiv.), $NiBr_2$ (3.3 mg, 0.015 mmol, 5 mol%), **L3** (7.1 mg, 0.033 mmol, 11 mol%), $PhCO_2Na$ (43.2 mg, 0.3 mmol, 1.0 equiv.), B_2Pin_2 (228.5 mg, 0.9 mmol, 3.0 equiv.), H_2O (16.2 µL, 0.9 mmol, 3.0 equiv.) and DMF (4 mL). The reaction was carried out under Ar at 80 °C for 12 h. Flash column chromatography (petroleum ether : AcOEt = 40:1) afforded the product **2h** as a white solid (68.9 mg, 96%, 95/5 *Z/E*). ^1H NMR (400 MHz, $CDCl_3$) δ 7.19 (d, J = 8.7 Hz, 4 H), 6.76 (d, J = 8.7 Hz, 4 H), 6.43 (s, 2 H), 3.77 (s, 6 H). ^{13}C NMR (101 MHz, $CDCl_3$) δ 158.49, 130.00, 129.96, 128.33, 113.56, 55.12. The spectroscopic data correspond to reported data.[32]

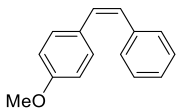

2i

(*Z*)-1-methoxy-4-styrylbenzene. According to the general procedure, the sealed tube was charged with the corresponding alkyne (0.3 mmol, 1.0 equiv.), $NiBr_2$ (3.3 mg, 0.015 mmol, 5 mol%), **L3** (7.1 mg, 0.033 mmol, 11 mol%), $PhCO_2Na$ (43.2 mg, 0.3 mmol, 1.0 equiv.), B_2Pin_2 (228.5 mg, 0.9 mmol, 3.0 equiv.), H_2O (16.2 µL, 0.9 mmol, 3.0 equiv.) and DMF (4 mL). The reaction was carried out under Ar at 80 °C for 12 h. Flash column chromatography (petroleum ether : AcOEt = 80:1) afforded the product **2i** as a yellow oil (59.4 mg, 94%, 93/7 *Z/E*). ^1H NMR (400 MHz, $CDCl_3$) δ 7.30-7.20 (m, 4 H), 7.17 (d, J = 8.7 Hz, 3 H), 6.74 (d, J = 8.6 Hz, 2 H), 6.51 (s, 2 H), 3.76 (s, 3 H). ^{13}C NMR (101 MHz, $CDCl_3$) δ 158.64, 137.58, 130.11, 129.73, 129.61, 128.77, 128.71, 128.19, 126.86, 113.55, 55.12. The spectroscopic data correspond to reported data.[46]

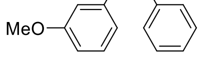

2j

(*Z*)-1-methoxy-3-styrylbenzene. According to the general procedure, the sealed tube was charged

with the corresponding alkyne (0.3 mmol, 1.0 equiv.), $NiBr_2$ (3.3 mg, 0.015 mmol, 5 mol%), **L3** (7.1 mg, 0.033 mmol, 11 mol%), $PhCO_2Na$ (43.2 mg, 0.3 mmol, 1.0 equiv.), B_2Pin_2 (228.5 mg, 0.9 mmol, 3.0 equiv.), H_2O (16.2 μL, 0.9 mmol, 3.0 equiv.) and DMF (4 mL). The reaction was carried out under Ar at 80 °C for 12 h. Flash column chromatography (petroleum ether : AcOEt = 80:1) afforded the product **2j** as a white solid (57.5 mg, 91%, 95/5 *Z/E*). ^{1}H NMR (400 MHz, $CDCl_3$) δ 7.41-7.06 (m, 6 H), 6.91-6.70 (m, 3 H), 6.59 (q, *J* = 12.2 Hz, 2 H), 3.64 (s, 3 H). ^{13}C NMR (101 MHz, $CDCl_3$) δ 159.34, 138.53, 137.26, 130.47, 130.13, 129.19, 128.89, 128.18, 127.11, 121.50, 113.72, 113.31, 55.00. The spectroscopic data correspond to reported data.[32]

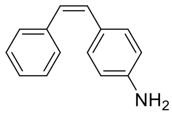

2k

(*Z*)-4-styrylaniline. According to the general procedure, the sealed tube was charged with the corresponding alkyne (0.3 mmol, 1.0 equiv.), $NiBr_2$ (3.3 mg, 0.015 mmol, 5 mol%), **L3** (7.1 mg, 0.033 mmol, 11 mol%), $PhCO_2Na$ (43.2 mg, 0.3 mmol, 1.0 equiv.), B_2Pin_2 (228.5 mg, 0.9 mmol, 3.0 equiv.), H_2O (16.2 μL, 0.9 mmol, 3.0 equiv.) and DMF (4 mL). The reaction was carried out under Ar at 80 °C for 12 h. Flash column chromatography (petroleum ether : AcOEt = 3:1) afforded the product **2k** as a yellow oil (42.3 mg, 72%, 84/16 *Z/E*). ^{1}H NMR (400 MHz, $CDCl_3$) δ 7.29 (d, *J* = 7.2 Hz, 2 H), 7.19 (dt, *J* = 23.6, 7.0 Hz, 3 H), 7.06 (d, *J* = 8.2 Hz, 2 H), 6.56-6.39 (m, 4 H), 3.64 (s, 2 H). ^{13}C NMR (101 MHz, $CDCl_3$) δ 145.46, 137.95, 130.16, 130.05, 128.78, 128.13, 127.59, 127.46, 126.66, 114.67. The spectroscopic data correspond to reported data.[46]

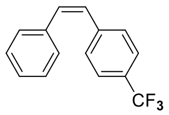

2l

(*Z*)-1-styryl-4-(trifluoromethyl)benzene. According to the general procedure, the sealed tube was charged with the corresponding alkyne (0.3 mmol, 1.0 equiv.), $NiBr_2$ (3.3 mg, 0.015 mmol, 5 mol%), **L3** (7.1 mg, 0.033 mmol, 11 mol%), $PhCO_2Na$ (43.2 mg, 0.3 mmol, 1.0 equiv.), B_2Pin_2 (228.5 mg, 0.9 mmol, 3.0 equiv.), H_2O (16.2 μL, 0.9 mmol, 3.0 equiv.) and DMF (4 mL). The reaction was carried out under Ar at 80 °C for 12 h. Flash column chromatography (petroleum ether : AcOEt = 100:1) afforded the product **2l** as a white solid (70.2 mg, 94%, 96/4 *Z/E*). ^{1}H NMR (400 MHz, $CDCl_3$) δ 7.46 (d, *J* = 8.1 Hz, 1H), 7.32 (d, *J* = 8.1 Hz, 1H), 7.28-7.16 (m, 2H), 6.71 (d, *J* = 12.2 Hz, 1H), 6.58 (d, *J* = 12.2 Hz, 1H). ^{19}F NMR (376 MHz, $CDCl_3$) δ -62.53. ^{13}C NMR (101 MHz, $CDCl_3$) δ 140.90, 136.55, 132.32, 129.13, 128.81, 128.73, 128.41, 127.56, 125.14 (q, *J* = 3.8 Hz), 124.18 (q, *J* = 271.9 Hz). The spectroscopic data correspond to reported data.[46]

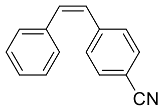

2m

(*Z*)-4-styrylbenzonitrile. According to the general procedure, the sealed tube was charged with the corresponding alkyne (0.3 mmol, 1.0 equiv.), $NiBr_2$ (3.3 mg, 0.015 mmol, 5 mol%), **L3** (7.1 mg, 0.033 mmol, 11 mol%), $PhCO_2Na$ (43.2 mg, 0.3 mmol, 1.0 equiv.), B_2Pin_2 (228.5 mg, 0.9 mmol, 3.0 equiv.), H_2O (16.2 μL, 0.9 mmol, 3.0 equiv.) and DMF (4 mL). The reaction was carried out under Ar at 80 °C for 12 h. Flash column chromatography (petroleum ether : AcOEt = 100:1)

afforded the product **2m** as a yellow oil (53.3 mg, 87%, 97/3 *Z/E*). ^{1}H NMR (400 MHz, $CDCl_3$) δ 7.47 (d, *J* = 8.3 Hz, 2 H), 7.30 (d, *J* = 8.2 Hz, 2 H), 7.24 (dd, *J* = 5.2, 1.7 Hz, 3 H), 7.18 (dd, *J* = 6.9, 2.2 Hz, 2 H), 6.75 (d, *J* = 12.2 Hz, 1 H), 6.56 (d, *J* = 12.2 Hz, 1 H). ^{13}C NMR (101 MHz, $CDCl_3$) δ 142.04, 136.20, 133.29, 131.98, 129.50, 128.73, 128.47, 128.34, 127.79, 118.91, 110.44. The spectroscopic data correspond to reported data.[29]

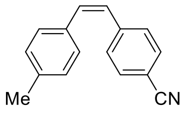

2n

(*Z*)-4-(4-methylstyryl)benzonitrile. According to the general procedure, the sealed tube was charged with the corresponding alkyne (0.3 mmol, 1.0 equiv.), $NiBr_2$ (3.3 mg, 0.015 mmol, 5 mol%), **L3** (7.1 mg, 0.033 mmol, 11 mol%), $PhCO_2Na$ (43.2 mg, 0.3 mmol, 1.0 equiv.), B_2Pin_2 (228.5 mg, 0.9 mmol, 3.0 equiv.), H_2O (16.2 μL, 0.9 mmol, 3.0 equiv.) and DMF (4 mL). The reaction was carried out under Ar at 80 °C for 12 h. Flash column chromatography (petroleum ether : AcOEt = 50:1) afforded the product **2n** as a white solid (63.1mg, 95%, 96/4 *Z/E*). ^{1}H NMR (400 MHz, $CDCl_3$) δ 7.50 (d, *J* = 8.3 Hz, 2 H), 7.34 (d, *J* = 8.2 Hz, 2 H), 7.08 (q, *J* = 8.2 Hz, 4 H), 6.73 (d, *J* = 12.2 Hz, 1 H), 6.53 (d, *J* = 12.2 Hz, 1 H), 2.34 (s, 3 H). ^{13}C NMR (101 MHz, $CDCl_3$) δ 142.27, 137.67, 133.20, 131.90, 129.43, 129.11, 128.63, 127.59, 118.90, 110.25, 21.17. The spectroscopic data correspond to reported data.[29]

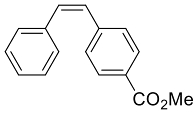

2o

Methyl (*Z*)-4-styrylbenzoate. According to the general procedure, the sealed tube was charged with the corresponding alkyne (0.3 mmol, 1.0 equiv.), $NiBr_2$ (3.3 mg, 0.015 mmol, 5 mol%), **L3** (7.1 mg, 0.033 mmol, 11 mol%), $PhCO_2Na$ (43.2 mg, 0.3 mmol, 1.0 equiv.), B_2Pin_2 (228.5 mg, 0.9 mmol, 3.0 equiv.), H_2O (16.2 μL, 0.9 mmol, 3.0 equiv.) and DMF (4 mL). The reaction was carried out under Ar at 80 °C for 12 h. Flash column chromatography (petroleum ether : AcOEt = 50:1) afforded the product **2o** as a white solid (69.2 mg, 96%, 93/7 *Z/E*). ^{1}H NMR (400 MHz, $CDCl_3$) δ 7.88 (d, *J* = 8.3 Hz, 2 H), 7.29 (d, *J* = 8.3 Hz, 2 H), 7.21 (s, 5 H), 6.70 (d, *J* = 12.3 Hz, 1 H), 6.59 (d, *J* = 12.3 Hz, 1 H), 3.88 (s, 3 H). ^{13}C NMR (101 MHz, $CDCl_3$) δ 166.82, 142.04, 136.62, 132.18, 129.48, 129.17, 128.80, 128.55, 128.29, 127.46, 51.96. The spectroscopic data correspond to reported data.[29]

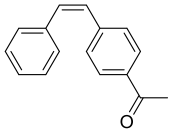

2p

(*Z*)-1-(4-styrylphenyl)ethan-1-one. According to the general procedure, the sealed tube was charged with the corresponding alkyne (0.3 mmol, 1.0 equiv.), $NiBr_2$ (3.3 mg, 0.015 mmol, 5 mol%), **L3** (7.1 mg, 0.033 mmol, 11 mol%), $PhCO_2Na$ (43.2 mg, 0.3 mmol, 1.0 equiv.), B_2Pin_2 (228.5 mg, 0.9 mmol, 3.0 equiv.), H_2O (16.2 μL, 0.9 mmol, 3.0 equiv.) and DMF (4 mL). The reaction was carried out under Ar at 80 °C for 12 h. Flash column chromatography (petroleum ether : AcOEt = 35:1) afforded the product **2p** as a white solid (51.8 mg, 78%, 95/5 *Z/E*). ^{1}H NMR (400 MHz, $CDCl_3$) δ 7.80 (d, *J* = 8.2 Hz, 2 H), 7.32 (d, *J* = 8.2 Hz, 2 H), 7.22 (s, 5 H), 6.72 (d, *J* = 12.2 Hz, 1 H), 6.59 (d, *J* = 12.3 Hz, 1 H), 2.55 (s, 3 H). ^{13}C NMR (101 MHz, $CDCl_3$) δ 197.49, 142.23, 136.63, 135.59, 132.38,

129.07, 128.99, 128.78, 128.32, 128.26, 127.50, 26.45. The spectroscopic data correspond to reported data.[35]

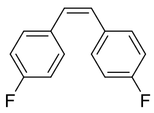

2q

(*Z*)-1,2-bis(4-fluorophenyl)ethene. According to the general procedure, the sealed tube was charged with the corresponding alkyne (0.3 mmol, 1.0 equiv.), $NiBr_2$ (3.3 mg, 0.015 mmol, 5 mol%), **L3** (7.1 mg, 0.033 mmol, 11 mol%), $PhCO_2Na$ (43.2 mg, 0.3 mmol, 1.0 equiv.), B_2Pin_2 (228.5 mg, 0.9 mmol, 3.0 equiv.), H_2O (16.2 µL, 0.9 mmol, 3.0 equiv.) and DMF (4 mL). The reaction was carried out under Ar at 80 °C for 12 h. Flash column chromatography (petroleum ether) afforded the product **2q** as a white solid (56.2 mg, 87%, 94/6 *Z/E*). ^{1}H NMR (400 MHz, $CDCl_3$) δ 7.17 (dd, J = 8.5, 5.7 Hz, 4 H), 6.91 (t, J = 8.7 Hz, 4 H), 6.53 (s, 2 H). ^{19}F NMR (376 MHz, $CDCl_3$) δ -114.42. ^{13}C NMR (101 MHz, $CDCl_3$) δ 161.84 (d, J = 246.9 Hz), 132.95 (d, J = 3.5 Hz), 130.46 (d, J = 7.9 Hz), 129.07 (d, J = 1.0 Hz), 115.24 (d, J = 21.4 Hz). The spectroscopic data correspond to reported data.[46]

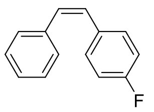

2r

(*Z*)-1-fluoro-4-styrylbenzene. According to the general procedure, the sealed tube was charged with the corresponding alkyne (0.3 mmol, 1.0 equiv.), $NiBr_2$ (3.3 mg, 0.015 mmol, 5 mol%), **L3** (7.1 mg, 0.033 mmol, 11 mol%), $PhCO_2Na$ (43.2 mg, 0.3 mmol, 1.0 equiv.), B_2Pin_2 (228.5 mg, 0.9 mmol, 3.0 equiv.), H_2O (16.2 µL, 0.9 mmol, 3.0 equiv.) and DMF (4 mL). The reaction was carried out under Ar at 80 °C for 12 h. Flash column chromatography (petroleum ether) afforded the product **2r** as a colorless oil (43.4 mg, 73%, 98/2 *Z/E*). ^{1}H NMR (400 MHz, $CDCl_3$) δ 7.34-7.09 (m, 1H), 6.90 (t, J = 8.7 Hz, 1H), 6.56 (q, J = 12.2 Hz, 1H). ^{19}F NMR (376 MHz, $CDCl_3$) δ -114.67. ^{13}C NMR (101 MHz, $CDCl_3$) δ 161.81 (d, J = 246.7 Hz), 137.03, 133.17 (d, J = 3.5 Hz), 130.51 (d, J = 7.9 Hz), 130.25 (d, J = 1.0 Hz), 129.06, 128.80, 128.29, 127.17, 115.13 (d, J = 21.4 Hz). The spectroscopic data correspond to reported data.[46]

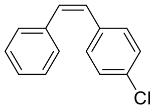

2s

(*Z*)-1-chloro-4-styrylbenzene. According to the general procedure, the sealed tube was charged with the corresponding alkyne (0.3 mmol, 1.0 equiv.), $NiBr_2$ (3.3 mg, 0.015 mmol, 5 mol%), **L3** (7.1 mg, 0.033 mmol, 11 mol%), $PhCO_2Na$ (43.2 mg, 0.3 mmol, 1.0 equiv.), B_2Pin_2 (228.5 mg, 0.9 mmol, 3.0 equiv.), H_2O (16.2 µL, 0.9 mmol, 3.0 equiv.) and DMF (4 mL). The reaction was carried out under Ar at 80 °C for 12 h. Flash column chromatography (petroleum ether) afforded the product **2s** as a colorless oil (59.4 mg, 92%, 95/5 *Z/E*). ^{1}H NMR (400 MHz, $CDCl_3$) δ 7.24-7.19 (m, 5 H), 7.19-7.11 (m, 4 H), 6.62 (d, J = 12.2 Hz, 1 H), 6.51 (d, J = 12.2 Hz, 1 H). ^{13}C NMR (101 MHz, $CDCl_3$) δ 136.86, 135.63, 132.74, 130.94, 130.19, 128.90, 128.78, 128.39, 128.32, 127.30. The spectroscopic data correspond to reported data.[46]

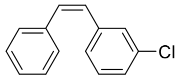

2t

(*Z*)-1-chloro-3-styrylbenzene. According to the general procedure, the sealed tube was charged with the corresponding alkyne (0.3 mmol, 1.0 equiv.), $NiBr_2$ (3.3 mg, 0.015 mmol, 5 mol%), **L3** (7.1 mg, 0.033 mmol, 11 mol%), $PhCO_2Na$ (43.2 mg, 0.3 mmol, 1.0 equiv.), B_2Pin_2 (228.5 mg, 0.9 mmol, 3.0 equiv.), H_2O (16.2 μL, 0.9 mmol, 3.0 equiv.) and DMF (4 mL). The reaction was carried out under Ar at 80 °C for 12 h. Flash column chromatography (petroleum ether) afforded the product **2t** as a colorless oil (60 mg, 93%, 94/6 *Z/E*). ^1H NMR (400 MHz, $CDCl_3$) δ 7.36-7.25 (m, 1H), 7.24-7.13 (m, 1H), 6.71 (d, *J* = 12.2 Hz, 1H), 6.58 (d, *J* = 12.2 Hz, 1H). ^{13}C NMR (101 MHz, $CDCl_3$) δ 139.09, 136.60, 134.05, 131.55, 129.40, 128.82, 128.80, 128.70, 128.30, 127.43, 127.11, 126.98. The spectroscopic data correspond to reported data.[46]

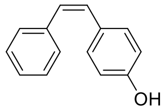

2u

(*Z*)-4-styrylphenol. According to the general procedure, the sealed tube was charged with the corresponding alkyne (0.3 mmol, 1.0 equiv.), $NiBr_2$ (3.3 mg, 0.015 mmol, 5 mol%), **L3** (7.1 mg, 0.033 mmol, 11 mol%), $PhCO_2Na$ (43.2 mg, 0.3 mmol, 1.0 equiv.), B_2Pin_2 (228.5 mg, 0.9 mmol, 3.0 equiv.), H_2O (16.2 μL, 0.9 mmol, 3.0 equiv.) and DMF (4 mL). The reaction was carried out under Ar at 80 °C for 8 h. Flash column chromatography (petroleum ether : AcOEt = 5:1) afforded the product **2u** as a white solid (41.5 mg, 71%, 61/39 *Z/E*). ^1H NMR (400 MHz, $CDCl_3$) δ 7.28-7.17 (m, 5H), 7.13 (d, *J* = 8.4 Hz, 2H), 6.67 (d, *J* = 8.4 Hz, 2H), 6.51 (s, 2H), 4.86 (s, 1H). ^{13}C NMR (101 MHz, $CDCl_3$) δ 154.58, 137.55, 130.34, 129.68, 128.89, 128.81, 128.21, 126.91, 115.09. The spectroscopic data correspond to reported data.[29]

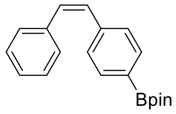

2v

(*Z*)-4,4,5,5-tetramethyl-2-(4-styrylphenyl)-1,3,2-dioxaborolane. According to the general procedure, the sealed tube was charged with the corresponding alkyne (0.3 mmol, 1.0 equiv.), $NiBr_2$ (3.3 mg, 0.015 mmol, 5 mol%), **L3** (7.1 mg, 0.033 mmol, 11 mol%), $PhCO_2Na$ (43.2 mg, 0.3 mmol, 1.0 equiv.), B_2Pin_2 (228.5 mg, 0.9 mmol, 3.0 equiv.), H_2O (16.2 μL, 0.9 mmol, 3.0 equiv.) and DMF (4 mL). The reaction was carried out under Ar at 80 °C for 12 h. Flash column chromatography (petroleum ether : AcOEt = 30:1) afforded the product **2v** as a yellow solid (71.2 mg, 78%, 97/3 *Z/E*). ^1H NMR (400 MHz, $CDCl_3$) δ 7.84-7.50 (m, 2H), 7.32-7.11 (m, 7H), 6.74-6.46 (m, 2H), 1.32 (s, 12H). ^{13}C NMR (101 MHz, $CDCl_3$) δ 140.16, 137.07, 134.63, 130.91, 130.17, 128.86, 128.17, 128.14, 127.15, 83.69, 24.85. The spectroscopic data correspond to reported data.[48]

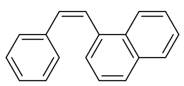

2w

(*Z*)-2-styrylnaphthalene. According to the general procedure, the sealed tube was charged with the corresponding alkyne (0.3 mmol, 1.0 equiv.), $NiBr_2$ (3.3 mg, 0.015 mmol, 5 mol%), **L3** (7.1 mg, 0.033 mmol, 11 mol%), $PhCO_2Na$ (43.2 mg, 0.3 mmol, 1.0 equiv.), B_2Pin_2 (228.5 mg, 0.9 mmol,

3.0 equiv.), H_2O (16.2 μL, 0.9 mmol, 3.0 equiv.) and DMF (4 mL). The reaction was carried out under Ar at 80 °C for 12 h. Flash column chromatography (petroleum ether) afforded the product **2w** as a white solid (66.8 mg, 96%, 97/3 *Z/E*). ^{1}H NMR (400 MHz, $CDCl_3$) δ 8.11-8.01 (m, 1 H), 7.90 -7.81 (m, 1 H), 7.74 (d, *J* = 7.8 Hz, 1 H), 7.47 (dt, *J* = 6.3, 4.2 Hz, 2 H), 7.39-7.26 (m, 2 H), 7.12-6.99 (m, 6 H), 6.81 (d, *J* = 12.2 Hz, 1 H). ^{13}C NMR (101 MHz, $CDCl_3$) δ 136.72, 135.25, 133.68, 132.01, 131.57, 129.03, 128.46, 128.41, 128.01, 127.49, 127.05, 126.43, 126.01, 125.92, 125.57, 124.90. The spectroscopic data correspond to reported data.[32]

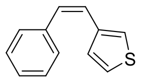

2x

(*Z*)-3-styrylthiophene. According to the general procedure, the sealed tube was charged with the corresponding alkyne (0.3 mmol, 1.0 equiv.), $NiBr_2$ (3.3 mg, 0.015 mmol, 5 mol%), **L3** (7.1 mg, 0.033 mmol, 11 mol%), $PhCO_2Na$ (43.2 mg, 0.3 mmol, 1.0 equiv.), B_2Pin_2 (228.5 mg, 0.9 mmol, 3.0 equiv.), H_2O (16.2 μL, 0.9 mmol, 3.0 equiv.) and DMF (4 mL). The reaction was carried out under Ar at 80 °C for 12 h. Flash column chromatography (petroleum ether : AcOEt = 30:1) afforded the product **2x** as a white solid (48.6 mg, 87%, 93/7 *Z/E*). ^{1}H NMR (400 MHz, $CDCl_3$) δ 7.38-7.17 (m, 3H), 7.10 (d, *J* = 5.4 Hz, 1H), 6.86 (d, *J* = 4.2 Hz, 1H), 6.66-6.47 (m, 1H). ^{13}C NMR (101 MHz, $CDCl_3$) δ 138.25, 137.80, 129.51, 128.71, 128.27, 127.99, 127.14, 124.82, 124.39, 124.04. The spectroscopic data correspond to reported data.[35]

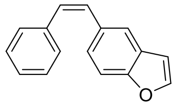

2y

(*Z*)-5-styrylbenzofuran. According to the general procedure, the sealed tube was charged with the corresponding alkyne (0.3 mmol, 1.0 equiv.), $NiBr_2$ (3.3 mg, 0.015 mmol, 5 mol%), **L3** (7.1 mg, 0.033 mmol, 11 mol%), $PhCO_2Na$ (43.2 mg, 0.3 mmol, 1.0 equiv.), B_2Pin_2 (228.5 mg, 0.9 mmol, 3.0 equiv.), H_2O (16.2 μL, 0.9 mmol, 3.0 equiv.) and DMF (4 mL). The reaction was carried out under Ar at 80 °C for 12 h. Flash column chromatography (petroleum ether : AcOEt = 30:1) afforded the product **2y** as a white solid (52.9 mg, 82%, 89/11 *Z/E*). ^{1}H NMR (400 MHz, $CDCl_3$) δ 7.56 (d, *J* = 1.5 Hz, 1 H), 7.48 (s, 1 H), 7.33 (d, *J* = 8.5 Hz, 1 H), 7.22 (dd, *J* = 21.4, 6.4 Hz, 6 H), 6.67 (dd, *J* = 17.1, 6.5 Hz, 2 H), 6.58 (d, *J* = 12.2 Hz, 1 H). ^{13}C NMR (101 MHz, $CDCl_3$) δ 154.12, 145.19, 137.34, 132.04, 130.40, 129.51, 128.91, 128.19, 127.44, 126.98, 125.50, 121.42, 111.07, 106.65. The spectroscopic data correspond to reported data.[35]

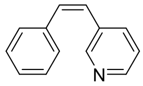

2z

(*Z*)-3-styrylpyridine. According to the general procedure, the sealed tube was charged with the corresponding alkyne (0.3 mmol, 1.0 equiv.), $NiBr_2$ (3.3 mg, 0.015 mmol, 5 mol%), **L3** (7.1 mg, 0.033 mmol, 11 mol%), $PhCO_2Na$ (43.2 mg, 0.3 mmol, 1.0 equiv.), B_2Pin_2 (228.5 mg, 0.9 mmol, 3.0 equiv.), H_2O (16.2 μL, 0.9 mmol, 3.0 equiv.) and DMF (4 mL). The reaction was carried out under Ar at 80 °C for 12 h, the result solution was diluted with ethyl acetate, washed with water and concentrated in vacuum. Flash column chromatography (petroleum ether : AcOEt = 11:1) afforded the product **2z** as a white solid (39 mg, 71%, 95/5 *Z/E*). ^{1}H NMR (400 MHz, $CDCl_3$) δ 8.48 (s, 1H), 8.42 (d, *J* = 4.1 Hz, 1H), 7.51 (d, *J* = 8.0 Hz, 1H), 7.23 (dt, *J* = 12.2, 5.9 Hz, 5H), 7.12 (dd, *J* = 7.7,

4.9 Hz, 1H), 6.75 (d, *J* = 12.2 Hz, 1H), 6.54 (d, *J* = 12.2 Hz, 1H). ^{13}C NMR (101 MHz, $CDCl_3$) δ 150.17, 148.07, 136.54, 135.77, 133.00, 132.69, 128.68, 128.50, 127.57, 126.43, 122.96. The spectroscopic data correspond to reported data.[46]

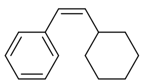

2bb

(*Z*)-(2-cyclohexylvinyl)benzene. According to the general procedure, the sealed tube was charged with the corresponding alkyne (0.3 mmol, 1.0 equiv.), $NiBr_2$ (3.3 mg, 0.015 mmol, 5 mol%), **L3** (7.1 mg, 0.033 mmol, 11 mol%), $PhCO_2Na$ (43.2 mg, 0.3 mmol, 1.0 equiv.), B_2Pin_2 (228.5 mg, 0.9 mmol, 3.0 equiv.), H_2O (16.2 µL, 0.9 mmol, 3.0 equiv.) and DMF (4 mL). The reaction was carried out under Ar at 80 °C for 30 h. Flash column chromatography (petroleum ether) afforded the product **2bb** as a colorless oil (49.9 mg, 89%, >99/1 *Z/E*). ^1H NMR (400 MHz, $CDCl_3$) δ 7.32 (t, *J* = 7.5 Hz, 2 H), 7.25 (d, *J* = 7.3 Hz, 2 H), 7.21 (t, *J* = 7.2 Hz, 1 H), 6.30 (d, *J* = 11.7 Hz, 1 H), 5.48 (t, *J* = 10.9 Hz, 1 H), 2.58 (q, *J* = 10.5 Hz, 1 H), 1.74-1.64 (m, 4 H), 1.35-1.10 (m, 6 H). ^{13}C NMR (101 MHz, $CDCl_3$) δ 138.95, 137.97, 128.58, 128.15, 126.84, 126.38, 36.88, 33.26, 26.03, 25.67. The spectroscopic data correspond to reported data.[49]

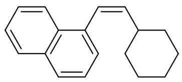

2cc

(*Z*)-1-(2-cyclohexylvinyl)naphthalene. According to the general procedure, the sealed tube was charged with the corresponding alkyne (0.3 mmol, 1.0 equiv.), $NiBr_2$ (3.3 mg, 0.015 mmol, 5 mol%), **L3** (7.1 mg, 0.033 mmol, 11 mol%), $PhCO_2Na$ (43.2 mg, 0.3 mmol, 1.0 equiv.), B_2Pin_2 (228.5 mg, 0.9 mmol, 3.0 equiv.), H_2O (16.2 µL, 0.9 mmol, 3.0 equiv.) and DMF (4 mL). The reaction was carried out under Ar at 80 °C for 30 h. Flash column chromatography (petroleum ether) afforded the product **2cc** as a colorless oil (63.9 mg, 90%, >99/1 *Z/E*). ^1H NMR (400 MHz, $CDCl_3$) δ 8.08 (dd, *J* = 6.1, 3.4 Hz, 1H), 7.90 (dd, *J* = 6.1, 3.3 Hz, 1H), 7.82 (d, *J* = 8.2 Hz, 1H), 7.58-7.49 (m, 3H), 7.40 (d, *J* = 7.0 Hz, 1H), 6.82 (d, *J* = 11.5 Hz, 1H), 5.82 (t, *J* = 10.8 Hz, 1H), 2.38 (dd, *J* = 13.5, 6.3 Hz, 1H), 1.70 (m, 5H), 1.31-1.16 (m, 5H). ^{13}C NMR (101 MHz, $CDCl_3$) δ 140.32, 135.33, 133.49, 132.03, 128.25, 127.03, 126.05, 125.69, 125.67, 125.33, 125.13, 125.04, 37.10, 33.29, 25.97, 25.56. The spectroscopic data correspond to reported data.[50]

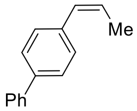

2dd

(*Z*)-4-(prop-1-en-1-yl)-1,1'-biphenyl. According to the general procedure, the sealed tube was charged with the corresponding alkyne (0.3 mmol, 1.0 equiv.), $NiBr_2$ (3.3 mg, 0.015 mmol, 5 mol%), **L3** (7.1 mg, 0.033 mmol, 11 mol%), $PhCO_2Na$ (43.2 mg, 0.3 mmol, 1.0 equiv.), B_2Pin_2 (228.5 mg, 0.9 mmol, 3.0 equiv.), H_2O (16.2 µL, 0.9 mmol, 3.0 equiv.) and DMF (4 mL). The reaction was carried out under Ar at 100 °C for 12 h. Flash column chromatography (petroleum ether) afforded the product **2dd** as a white solid (46.2 mg, 79%, >99/1 *Z/E*). ^1H NMR (400 MHz, $CDCl_3$) δ 7.64 (dd, *J* = 12.3, 7.9 Hz, 4H), 7.54-7.28 (m, 5H), 6.52 (d, *J* = 11.6 Hz, 1H), 5.87 (dq, *J* = 11.7, 7.2 Hz, 1H), 2.00 (dd, *J* = 7.2, 1.6 Hz, 3H). ^{13}C NMR (101 MHz, $CDCl_3$) δ 140.87, 139.16, 136.68, 129.45,

129.25, 128.74, 127.17, 126.96, 126.79, 14.74. The spectroscopic data correspond to reported data.[51]

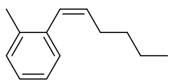

2ee

(*Z*)-1-(hex-1-en-1-yl)-2-methylbenzene. According to the general procedure, the sealed tube was charged with the corresponding alkyne (0.3 mmol, 1.0 equiv.), $NiBr_2$ (3.3 mg, 0.015 mmol, 5 mol%), **L3** (7.1 mg, 0.033 mmol, 11 mol%), $PhCO_2Na$ (43.2 mg, 0.3 mmol, 1.0 equiv.), B_2Pin_2 (228.5 mg, 0.9 mmol, 3.0 equiv.), H_2O (16.2 μL, 0.9 mmol, 3.0 equiv.) and DMF (4 mL). The reaction was carried out under Ar at 100 °C for 12 h. Flash column chromatography (petroleum ether) afforded the product **2ee** as a colorless oil (44.6 mg, 85%, >99/1 *Z/E*). ^{1}H NMR (400 MHz, $CDCl_3$) δ 7.25-7.01 (m, 4 H), 6.41 (d, *J* = 11.4 Hz, 1 H), 5.70 (dt, *J* = 11.4, 7.4 Hz, 1 H), 2.24 (s, 3 H), 2.14 (dd, *J* = 13.8, 6.7 Hz, 2 H), 1.43-1.23 (m, 4 H), 0.85 (t, *J* = 7.1 Hz, 3 H). ^{13}C NMR (101 MHz, $CDCl_3$) δ 136.92, 136.19, 132.89, 129.69, 129.03, 127.81, 126.66, 125.22, 32.01, 28.06, 22.32, 19.86, 13.91. The spectroscopic data correspond to reported data.[39]

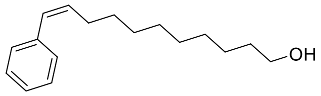

2ff

(*Z*)-11-phenylundec-10-en-1-ol. According to the general procedure, the sealed tube was charged with the corresponding alkyne (0.3 mmol, 1.0 equiv.), $NiBr_2$ (3.3 mg, 0.015 mmol, 5 mol%), **L3** (7.1 mg, 0.033 mmol, 11 mol%), $PhCO_2Na$ (43.2 mg, 0.3 mmol, 1.0 equiv.), B_2Pin_2 (228.5 mg, 0.9 mmol, 3.0 equiv.), H_2O (16.2 μL, 0.9 mmol, 3.0 equiv.) and DMF (4 mL). The reaction was carried out under Ar at 100 °C for 12 h. Flash column chromatography (petroleum ether : AcOEt = 10:1) afforded the product **2ff** as a yellow solid (56.2 mg, 76%, >99/1 *Z/E*). ^{1}H NMR (400 MHz, $CDCl_3$) δ 7.36-7.24 (m, 4 H), 7.21 (t, *J* = 7.1 Hz, 1 H), 6.40 (d, *J* = 11.6 Hz, 1 H), 5.66 (dt, *J* = 11.7, 7.3 Hz, 1 H), 3.62 (t, *J* = 6.6 Hz, 2 H), 2.32 (dd, *J* = 13.8, 6.8 Hz, 2 H), 1.54 (dd, *J* = 13.7, 6.8 Hz, 2 H), 1.43 (dd, *J* = 14.0, 7.0 Hz, 2 H), 1.28 (m, 10 H). ^{13}C NMR (101 MHz, $CDCl_3$) δ 137.81, 133.19, 128.72, 128.69, 128.05, 126.37, 63.03, 32.77, 29.92, 29.49, 29.38, 29.36, 29.27, 28.58, 25.69. The spectroscopic data correspond to reported data.[52]

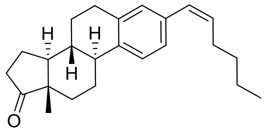

2gg

(8*R*,9*S*,13*S*,14*S*)-3-((*Z*)-hex-1-en-1-yl)-13-methyl-6,7,8,9,11,12,13,14,15,16-decahydro-17*H*-cyclopenta[*a*]phenanthren-17-one. According to the general procedure, the sealed tube was charged with the corresponding alkyne (0.3 mmol, 1.0 equiv.), $NiBr_2$ (3.3 mg, 0.015 mmol, 5 mol%), **L3** (7.1 mg, 0.033 mmol, 11 mol%), $PhCO_2Na$ (43.2 mg, 0.3 mmol, 1.0 equiv.), B_2Pin_2 (228.5 mg, 0.9 mmol, 3.0 equiv.), H_2O (16.2 μL, 0.9 mmol, 3.0 equiv.) and DMF (4 mL). The reaction was carried out under Ar at 100 °C for 12 h. Flash column chromatography (petroleum ether : AcOEt = 30:1) afforded the product **2gg** as a colorless oil (89.1 mg, 92%, >99/1 *Z/E*). ^{1}H NMR (400 MHz, $CDCl_3$) δ 7.30 (d, *J* = 7.8 Hz, 1 H), 7.14 (d, *J* = 8.0 Hz, 1 H), 7.06 (s, 1 H), 6.38 (d, *J* = 11.6 Hz, 1 H), 5.67 (dt, *J* = 11.7, 7.2 Hz, 1 H), 3.03-2.86 (m, 2 H), 2.64-2.29 (m, 5 H), 2.26-1.96 (m, 4 H), 1.74-1.37 (m, 10 H), 1.04-0.89 (m, 6 H). ^{13}C NMR (101 MHz, $CDCl_3$) δ 220.68, 137.92, 136.04,

135.33, 132.71, 129.31, 128.28, 126.15, 125.00, 50.46, 47.91, 44.33, 38.13, 35.77, 32.14, 31.56, 29.38, 28.39, 26.49, 25.63, 22.38, 21.52, 13.94, 13.78. HRMS (EI) Calcd. for $C_{24}H_{32}O^+$: 336.2448, Found: 336.2445.

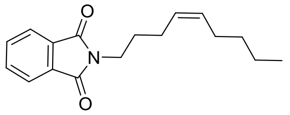

2ii

(*Z*)-2-(non-4-en-1-yl)isoindoline-1,3-dione. According to the general procedure, the sealed tube was charged with the corresponding alkyne (0.3 mmol, 1.0 equiv.), $NiBr_2$ (3.3 mg, 0.015 mmol, 5 mol%), **L3** (7.1 mg, 0.033 mmol, 11 mol%), $PhCO_2Na$ (43.2 mg, 0.3 mmol, 1.0 equiv.), B_2Pin_2 (228.5 mg, 0.9 mmol, 3.0 equiv.), H_2O (16.2 μL, 0.9 mmol, 3.0 equiv.) and DMF (4 mL). The reaction was carried out under Ar at 100 °C for 12 h. Flash column chromatography (petroleum ether : AcOEt = 35:1) afforded the product **2ii** as a colorless oil (72.4 mg, 89%, >99/1 *Z/E*). ^{1}H NMR (400 MHz, $CDCl_3$) δ 7.81 (dd, *J* = 5.3, 3.1 Hz, 2 H), 7.68 (dd, *J* = 5.3, 3.0 Hz, 2 H), 5.45-5.22 (m, 2 H), 3.66 (t, *J* = 7.4 Hz, 2 H), 2.08 (dd, *J* = 13.5, 6.9 Hz, 2 H), 1.99 (d, *J* = 5.7 Hz, 2 H), 1.76-1.59 (m, 2 H), 1.26 (dd, *J* = 10.6, 6.8 Hz, 4 H), 0.86 (d, *J* = 6.7 Hz, 3 H). ^{13}C NMR (101 MHz, $CDCl_3$) δ 168.26, 133.74, 132.14, 130.98, 127.99, 123.05, 37.70, 31.74, 28.46, 26.86, 24.59, 22.23, 13.87. The spectroscopic data correspond to reported data.[39]

2.17 General Procedure for *E*-Selective Transfer Semihydrogenation of Alkynes 1

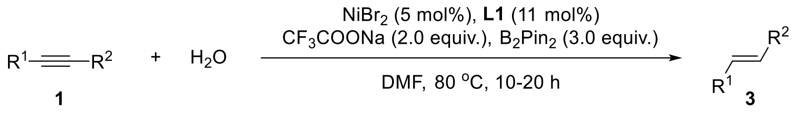

To a sealed tube were added alkyne **1** (0.3 mmol), $NiBr_2$ (3.3 mg, 0.015 mmol, 5 mol%), **L1** (5.2 mg, 0.033 mmol, 11 mol%), CF_3CO_2Na (81.6 mg, 0.6 mmol, 2.0 equiv.) and B_2Pin_2 (228.5 mg, 0.9 mmol, 3.0 equiv.). The flask was evacuated and refilled with argon, followed by the addition of H_2O (16.2 μL, 0.9 mmol, 3.0 equiv.) and DMF (4 mL). The mixture was stirred at 80 °C for 10-20 h until the reaction was completed as monitored by TLC. The resultant solution was diluted with ethyl acetate, washed with HCl aqueous solution (1 M) (the reaction of **1k**, **1z**, **1aa** was washed with water) and concentrated in vacuum. The mixture was detected by GC directly or after simple filtration in some cases to determine the *Z*/*E* ratio (GC analysis was acquired on SHIMADZU GC-2030AF gas chromatograph fitted with SH-RTX-5 column (30 m length × 250 μm × 0.25 μm) using the following method: SHIMADZU GC-2030AF GC system: FID starting temp: 80 °C, Time at starting temp: 2 min, Ramp: 27.5 °C/min up to 300 °C with hold time = 10 min, Flow rate (carrier): 1.62 mL/min (N_2), Split ratio: 39, inlet temperature: 300 °C, detector temperature: 300 °C). The crude product was purified by chromatography on silica gel (300-400 mesh), eluted with petroleum ether with 0-20 % of ethyl acetate to give alkene product. Extremely careful column chromatography was able to partially deliver the major product in a pure form to provide precise NMR spectra of the major product. The overall isolated yield was calculated based on the combination of all parts.

Characterization Data of *E*-alkenes

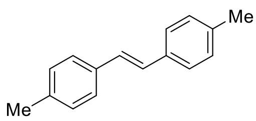

3a

(*E*)-1,2-di-p-tolylethene. According to the general procedure, the sealed tube was charged with the corresponding alkyne (0.3 mmol, 1.0 equiv.), $NiBr_2$ (3.3 mg, 0.015 mmol, 5 mol%), **L1** (5.2 mg, 0.033 mmol, 11 mol%), CF_3CO_2Na (81.6 mg, 0.6 mmol, 2.0 equiv.), B_2Pin_2 (228.5 mg, 0.9 mmol, 3.0 equiv.), H_2O (16.2 µL, 0.9 mmol, 3.0 equiv.) and DMF (4 mL). The reaction was carried out under Ar at 80 °C for 12 h. Flash column chromatography (petroleum ether) afforded the product **3a** as a white solid (51.9 mg, 83%, 95/5 *E/Z*). ^1H NMR (400 MHz, $CDCl_3$) δ 7.38 (d, *J* = 8.0 Hz, 4 H), 7.14 (d, *J* = 7.9 Hz, 4 H), 7.02 (s, 2 H), 2.34 (s, 6 H). ^{13}C NMR (101 MHz, $CDCl_3$) δ 137.22, 134.74, 129.34, 127.64, 126.29, 21.19. The spectroscopic data correspond to reported data.[28]

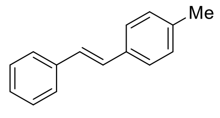

3b

(*E*)-1-methyl-4-styrylbenzene. According to the general procedure, the sealed tube was charged with the corresponding alkyne (0.3 mmol, 1.0 equiv.), $NiBr_2$ (3.3 mg, 0.015 mmol, 5 mol%), **L1** (5.2 mg, 0.033 mmol, 11 mol%), CF_3CO_2Na (81.6 mg, 0.6 mmol, 2.0 equiv.), B_2Pin_2 (228.5 mg, 0.9 mmol, 3.0 equiv.), H_2O (16.2 µL, 0.9 mmol, 3.0 equiv.) and DMF (4 mL). The reaction was carried out under Ar at 80 °C for 12 h. Flash column chromatography (petroleum ether) afforded the product **3b** as a white solid (54 mg, 93%, 95/5 *E/Z*). ^1H NMR (400 MHz, $CDCl_3$) δ 7.49 (d, *J* = 7.1 Hz, 2 H), 7.40 (d, *J* = 7.5 Hz, 2 H), 7.36-7.29 (m, 2 H), 7.27-7.19 (m, 1 H), 7.16 (d, *J* = 7.3 Hz, 2 H), 7.12-6.97 (m, 2 H), 2.35 (s, 3 H). ^{13}C NMR (101 MHz, $CDCl_3$) δ 137.51, 137.48, 134.55, 129.37, 128.62, 127.70, 127.38, 126.41, 126.38, 21.23. The spectroscopic data correspond to reported data.[28]

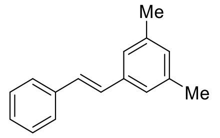

3c

(*E*)-1,3-dimethyl-5-styrylbenzene. According to the general procedure, the sealed tube was charged with the corresponding alkyne (0.3 mmol, 1.0 equiv.), $NiBr_2$ (3.3 mg, 0.015 mmol, 5 mol%), **L1** (5.2 mg, 0.033 mmol, 11 mol%), CF_3CO_2Na (81.6 mg, 0.6 mmol, 2.0 equiv.), B_2Pin_2 (228.5 mg, 0.9 mmol, 3.0 equiv.), H_2O (16.2 µL, 0.9 mmol, 3.0 equiv.) and DMF (4 mL). The reaction was carried out under Ar at 80 °C for 12 h. Flash column chromatography (petroleum ether) afforded the product **3c** as a white solid (53.6 mg, 86%, 97/3 *E/Z*). ^1H NMR (400 MHz, $CDCl_3$) δ 7.48 (d, *J* = 7.2 Hz, 2 H), 7.33 (t, *J* = 7.4 Hz, 2 H), 7.22 (dd, *J* = 15.0, 8.0 Hz, 1 H), 7.13 (s, 2 H), 7.07 (t, *J* = 10.4 Hz, 2 H), 6.89 (s, 1 H), 2.32 (s, 6 H). ^{13}C NMR (101 MHz, $CDCl_3$) δ 138.06, 137.50, 137.21, 129.40, 128.87, 128.62, 128.26, 127.42, 126.42, 124.41, 21.27. The spectroscopic data correspond to reported data.[28]

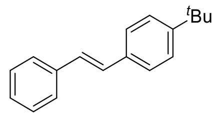

3d

(*E*)-1-(*tert*-butyl)-4-styrylbenzene. According to the general procedure, the sealed tube was charged with the corresponding alkyne (0.3 mmol, 1.0 equiv.), $NiBr_2$ (3.3 mg, 0.015 mmol, 5 mol%), **L1** (5.2 mg, 0.033 mmol, 11 mol%), CF_3CO_2Na (81.6 mg, 0.6 mmol, 2.0 equiv.), B_2Pin_2 (228.5 mg, 0.9 mmol, 3.0 equiv.), H_2O (16.2 µL, 0.9 mmol, 3.0 equiv.) and DMF (4 mL). The reaction was

carried out under Ar at 80 °C for 12 h. Flash column chromatography (petroleum ether) afforded the product **3d** as a white solid (61.2 mg, 86%, 96/4 *E/Z*). ^{1}H NMR (400 MHz, $CDCl_3$) δ 7.54 (d, *J* = 7.3 Hz, 2 H), 7.50 (d, *J* = 8.2 Hz, 2 H), 7.45-7.35 (m, 4 H), 7.29 (d, *J* = 7.2 Hz, 1 H), 7.18-7.07 (m, 2 H), 1.38 (s, 9 H). ^{13}C NMR (101 MHz, $CDCl_3$) δ 150.77, 137.55, 134.56, 128.63, 128.50, 127.93, 127.39, 126.41, 126.24, 125.59, 34.60, 31.28. The spectroscopic data correspond to reported data.[29]

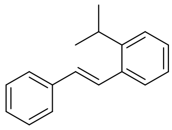

3e

(*E*)-1-isopropyl-2-styrylbenzene. According to the general procedure, the sealed tube was charged with the corresponding alkyne (0.3 mmol, 1.0 equiv.), $NiBr_2$ (3.3 mg, 0.015 mmol, 5 mol%), **L1** (5.2 mg, 0.033 mmol, 11 mol%), CF_3CO_2Na (81.6 mg, 0.6 mmol, 2.0 equiv.), B_2Pin_2 (228.5 mg, 0.9 mmol, 3.0 equiv.), H_2O (16.2 μL, 0.9 mmol, 3.0 equiv.) and DMF (4 mL). The reaction was carried out under Ar at 80 °C for 12 h. Flash column chromatography (petroleum ether) afforded the product **3e** as a colorless oil (89.1 mg, 66.7%, 93/7 *E/Z*). ^{1}H NMR (400 MHz, $CDCl_3$) δ 7.58-7.43 (m, 4 H), 7.35 (t, *J* = 7.6 Hz, 2 H), 7.26 (tdd, *J* = 7.3, 6.5, 3.7 Hz, 3 H), 7.22-7.16 (m, 1 H), 6.95 (d, *J* = 16.0 Hz, 1 H), 3.35 (dt, *J* = 13.7, 6.8 Hz, 1 H), 1.27 (d, *J* = 6.9 Hz, 6 H). ^{13}C NMR (101 MHz, $CDCl_3$) δ 146.18, 137.77, 135.56, 130.64, 128.66, 127.89, 127.52, 126.59, 126.52, 126.18, 125.92, 125.02, 29.25, 23.46. The spectroscopic data correspond to reported data.[53]

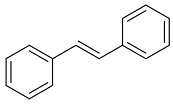

3g

(*E*)-1,2-diphenylethene. According to the general procedure, the sealed tube was charged with the corresponding alkyne (0.3 mmol, 1.0 equiv.), $NiBr_2$ (3.3 mg, 0.015 mmol, 5 mol%), **L1** (5.2 mg, 0.033 mmol, 11 mol%), CF_3CO_2Na (81.6 mg, 0.6 mmol, 2.0 equiv.), B_2Pin_2 (228.5 mg, 0.9 mmol, 3.0 equiv.), H_2O (16.2 μL, 0.9 mmol, 3.0 equiv.) and DMF (4 mL). The reaction was carried out under Ar at 80 °C for 12 h. Flash column chromatography (petroleum ether) afforded the product **3g** as a white solid (50.9 mg, 94%, 97/3 *E/Z*). ^{1}H NMR (400 MHz, $CDCl_3$) δ 7.51 (d, *J* = 7.2 Hz, 4 H), 7.35 (t, *J* = 7.4 Hz, 4 H), 7.30-7.22 (m, 2 H), 7.11 (s, 2 H). ^{13}C NMR (101 MHz, $CDCl_3$) δ 137.33, 128.69, 128.66, 127.60, 126.50. The spectroscopic data correspond to reported data.[28]

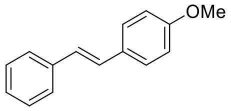

3i

(*E*)-1-methoxy-4-styrylbenzene. According to the general procedure, the sealed tube was charged with the corresponding alkyne (0.3 mmol, 1.0 equiv.), $NiBr_2$ (3.3 mg, 0.015 mmol, 5 mol%), **L1** (5.2 mg, 0.033 mmol, 11 mol%), CF_3CO_2Na (81.6 mg, 0.6 mmol, 2.0 equiv.), B_2Pin_2 (228.5 mg, 0.9 mmol, 3.0 equiv.), H_2O (16.2 μL, 0.9 mmol, 3.0 equiv.) and DMF (4 mL). The reaction was carried out under Ar at 80 °C for 12 h. Flash column chromatography (petroleum ether : AcOEt = 50:1) afforded the product **3i** as a white solid (61.3 mg, 97%, 96/4 *E/Z*). ^{1}H NMR (400 MHz, $CDCl_3$) δ 7.46 (dd, *J* = 14.4, 8.2 Hz, 4 H), 7.34 (t, *J* = 7.5 Hz, 2 H), 7.22 (t, *J* = 7.0 Hz, 1 H), 7.06 (d, *J* = 16.3 Hz, 1 H), 6.97 (d, *J* = 16.3 Hz, 1 H), 6.89 (d, *J* = 8.5 Hz, 2 H), 3.81 (s, 3 H). ^{13}C NMR (101 MHz, $CDCl_3$) δ 159.31, 137.65, 130.16, 128.61, 128.21, 127.70, 127.18, 126.62, 126.24, 114.13, 55.29.

The spectroscopic data correspond to reported data.[28]

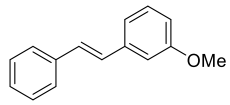

3j

(*E*)-1-methoxy-3-styrylbenzene. According to the general procedure, the sealed tube was charged with the corresponding alkyne (0.3 mmol, 1.0 equiv.), $NiBr_2$ (3.3 mg, 0.015 mmol, 5 mol%), **L1** (5.2 mg, 0.033 mmol, 11 mol%), CF_3CO_2Na (81.6 mg, 0.6 mmol, 2.0 equiv.), B_2Pin_2 (228.5 mg, 0.9 mmol, 3.0 equiv.), H_2O (16.2 μL, 0.9 mmol, 3.0 equiv.) and DMF (4 mL). The reaction was carried out under Ar at 80 °C for 12 h. Flash column chromatography (petroleum ether : AcOEt = 60:1) afforded the product **3j** as a white solid (62.4 mg, 98%, 96/4 *E/Z*). ^1H NMR (400 MHz, $CDCl_3$) δ 7.50 (d, *J* = 7.2 Hz, 2 H), 7.34 (t, *J* = 7.3 Hz, 2 H), 7.29-7.21 (m, 2 H), 7.08 (t, *J* = 13.4 Hz, 4 H), 6.81 (d, *J* = 6.7 Hz, 1 H), 3.83 (s, 3 H). ^{13}C NMR (101 MHz, $CDCl_3$) δ 159.88, 138.77, 137.21, 129.60, 128.98, 128.65, 128.57, 127.64, 126.52, 119.22, 113.27, 111.75, 55.20. The spectroscopic data correspond to reported data.[39]

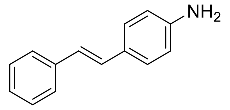

3k

(*E*)-4-styrylaniline. According to the general procedure, the sealed tube was charged with the corresponding alkyne (0.3 mmol, 1.0 equiv.), $NiBr_2$ (3.3 mg, 0.015 mmol, 5 mol%), **L1** (5.2 mg, 0.033 mmol, 11 mol%), CF_3CO_2Na (81.6 mg, 0.6 mmol, 2.0 equiv.), B_2Pin_2 (228.5 mg, 0.9 mmol, 3.0 equiv.), H_2O (16.2 μL, 0.9 mmol, 3.0 equiv.) and DMF (4 mL). The reaction was carried out under Ar at 80 °C for 12 h. Flash column chromatography (petroleum ether : AcOEt = 3:1) afforded the product **3k** as a yellow solid (41.7 mg, 70%, 94/6 *E/Z*). ^1H NMR (400 MHz, $CDCl_3$) δ 7.46 (d, *J* = 7.5 Hz, 2 H), 7.32 (t, *J* = 7.0 Hz, 4 H), 7.21 (dd, *J* = 14.0, 6.7 Hz, 1 H), 7.02 (d, *J* = 16.3 Hz, 1 H), 6.91 (d, *J* = 16.3 Hz, 1 H), 6.66 (d, *J* = 8.3 Hz, 2 H), 3.72 (s, 2 H). ^{13}C NMR (101 MHz, $CDCl_3$) δ 146.13, 137.95, 128.68, 128.57, 128.02, 127.73, 126.86, 126.08, 125.10, 115.18. The spectroscopic data correspond to reported data.[29]

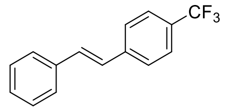

3l

(*E*)-1-styryl-4-(trifluoromethyl)benzene. According to the general procedure, the sealed tube was charged with the corresponding alkyne (0.3 mmol, 1.0 equiv.), $NiBr_2$ (3.3 mg, 0.015 mmol, 5 mol%), **L1** (5.2 mg, 0.033 mmol, 11 mol%), CF_3CO_2Na (81.6 mg, 0.6 mmol, 2.0 equiv.), B_2Pin_2 (228.5 mg, 0.9 mmol, 3.0 equiv.), H_2O (16.2 μL, 0.9 mmol, 3.0 equiv.) and DMF (4 mL). The reaction was carried out under Ar at 80 °C for 12 h. Flash column chromatography (petroleum ether) afforded the product **3l** as a white solid (58.2 mg, 78%, 96/4 *E/Z*). ^1H NMR (300 MHz, $CDCl_3$) δ 7.61 (s, 4 H), 7.55 (dd, *J* = 7.3, 1.5 Hz, 2 H), 7.44-7.36 (m, 2 H), 7.35-7.29 (m, 1 H), 7.21 (d, *J* = 16.4 Hz, 1 H), 7.12 (d, *J* = 16.4 Hz, 1 H). ^{19}F NMR (376 MHz, $CDCl_3$) δ -62.42. ^{13}C NMR (75 MHz, $CDCl_3$) δ 140.79, 136.61, 131.18, 129.24 (q, *J* = 32.3 Hz), 128.77, 128.27, 127.10, 126.75, 126.55, 125.60 (q, *J* = 3.7 Hz), 124.22 (q, *J* = 270.0 Hz). The spectroscopic data correspond to reported data.[29]

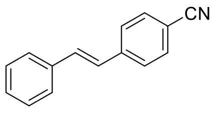

3m

(*E*)-4-styrylbenzonitrile. According to the general procedure, the sealed tube was charged with the corresponding alkyne (0.3 mmol, 1.0 equiv.), $NiBr_2$ (3.3 mg, 0.015 mmol, 5 mol%), **L1** (5.2 mg, 0.033 mmol, 11 mol%), CF_3CO_2Na (81.6 mg, 0.6 mmol, 2.0 equiv.), B_2Pin_2 (228.5 mg, 0.9 mmol, 3.0 equiv.), H_2O (16.2 μL, 0.9 mmol, 3.0 equiv.) and DMF (4 mL). The reaction was carried out under Ar at 80 °C for 12 h. Flash column chromatography (petroleum ether : AcOEt = 50:1) afforded the product **3m** as a white solid (59.1 mg, 96%, 96/4 *E/Z*). ^1H NMR (400 MHz, $CDCl_3$) δ 7.67-7.48 (m, 6 H), 7.38 (t, *J* = 7.4 Hz, 2 H), 7.29 (dd, *J* = 20.1, 13.0 Hz, 1 H), 7.20 (d, *J* = 16.3 Hz, 1 H), 7.07 (d, *J* = 16.3 Hz, 1 H). ^{13}C NMR (101 MHz, $CDCl_3$) δ 141.78, 136.24, 132.41, 132.36, 128.80, 128.58, 126.86, 126.80, 126.67, 118.94, 110.53. The spectroscopic data correspond to reported data.[29]

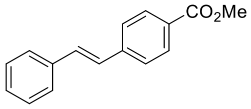

3o

Methyl (*E*)-4-styrylbenzoate. According to the general procedure, the sealed tube was charged with the corresponding alkyne (0.3 mmol, 1.0 equiv.), $NiBr_2$ (3.3 mg, 0.015 mmol, 5 mol%), **L1** (5.2 mg, 0.033 mmol, 11 mol%), CF_3CO_2Na (81.6 mg, 0.6 mmol, 2.0 equiv.), B_2Pin_2 (228.5 mg, 0.9 mmol, 3.0 equiv.), H_2O (16.2 μL, 0.9 mmol, 3.0 equiv.) and DMF (4 mL). The reaction was carried out under Ar at 80 °C for 12 h. Flash column chromatography (petroleum ether : AcOEt = 30:1) afforded the product **3o** as a white solid (58.7 mg, 82%, 97/3 *E/Z*). ^1H NMR (400 MHz, $CDCl_3$) δ 8.04 (d, *J* = 7.8 Hz, 2 H), 7.62-7.48 (t, *J* = 8.4 Hz, 4 H), 7.37 (d, *J* = 6.8 Hz, 2 H), 7.31 (d, *J* = 6.8 Hz, 1 H), 7.22 (d, *J* = 16.3 Hz, 1 H), 7.12 (d, *J* = 16.3 Hz, 1 H), 3.93 (s, 3 H). ^{13}C NMR (101 MHz, $CDCl_3$) δ 166.84, 141.88, 136.84, 131.30, 130.03, 129.03, 128.77, 128.22, 127.65, 126.80, 126.32, 51.98. The spectroscopic data correspond to reported data.[29]

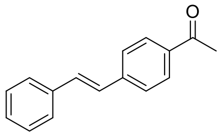

3p

(*E*)-1-(4-styrylphenyl)ethan-1-one. According to the general procedure, the sealed tube was charged with the corresponding alkyne (0.3 mmol, 1.0 equiv.), $NiBr_2$ (6.6 mg, 0.03 mmol, 10 mol%), **L1** (10.3 mg, 0.066 mmol, 22 mol%), CF_3CO_2Na (81.6 mg, 0.6 mmol, 2.0 equiv.), B_2Pin_2 (228.5 mg, 0.9 mmol, 3.0 equiv.), H_2O (16.2 μL, 0.9 mmol, 3.0 equiv.) and DMF (4 mL). The reaction was carried out under Ar at 80 °C for 12 h. Flash column chromatography (petroleum ether : AcOEt = 35:1) afforded the product **3p** as a yellow solid (52.8 mg, 79%, 94/6 *E/Z*). ^1H NMR (400 MHz, $CDCl_3$) δ 7.94 (d, *J* = 8.2 Hz, 1H), 7.55 (dd, *J* = 16.3, 7.9 Hz, 1H), 7.37 (t, *J* = 7.5 Hz, 1H), 7.28 (dd, *J* = 16.5, 9.3 Hz, 1H), 7.21 (d, *J* = 16.4 Hz, 1H), 7.11 (d, *J* = 16.3 Hz, 1H), 2.59 (s, 1H). ^{13}C NMR (101 MHz, $CDCl_3$) δ 197.34, 141.94, 136.65, 135.92, 131.41, 128.81, 128.74, 128.26, 127.40, 126.77, 126.44, 26.49. The spectroscopic data correspond to reported data.[36]

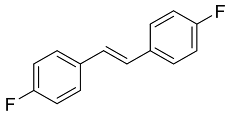

3q

(*E*)-1,2-bis(4-fluorophenyl)ethene. According to the general procedure, the sealed tube was charged with the corresponding alkyne (0.3 mmol, 1.0 equiv.), $NiBr_2$ (3.3 mg, 0.015 mmol, 5 mol%), **L1** (5.2 mg, 0.033 mmol, 11 mol%), CF_3CO_2Na (81.6 mg, 0.6 mmol, 2.0 equiv.), B_2Pin_2 (228.5 mg, 0.9 mmol, 3.0 equiv.), H_2O (16.2 μL, 0.9 mmol, 3.0 equiv.) and DMF (4 mL). The reaction was carried out under Ar at 80 °C for 12 h. Flash column chromatography (petroleum ether) afforded the product **3q** as a white solid (55.6 mg, 86%, 97/3 *E/Z*). ^{1}H NMR (300 MHz, $CDCl_3$) δ 7.51 – 7.42 (m, 4 H), 7.08 (d, J = 2.2 Hz, 1 H), 7.06-7.05 (m, 2 H), 7.04-7.01 (m, 1 H), 6.98 (s, 2 H). ^{19}F NMR (376 MHz, $CDCl_3$) δ -114.15. ^{13}C NMR (75 MHz, $CDCl_3$) δ 162.33 (d, J = 247.3 Hz), 133.34 (d, J = 3.2 Hz), 127.89 (d, J = 8.1 Hz), 127.25, 115.63 (d, J = 21.7 Hz). The spectroscopic data correspond to reported data.[54]

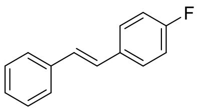

3r

(*E*)-1-fluoro-4-styrylbenzene. According to the general procedure, the sealed tube was charged with the corresponding alkyne (0.3 mmol, 1.0 equiv.), $NiBr_2$ (3.3 mg, 0.015 mmol, 5 mol%), **L1** (5.2 mg, 0.033 mmol, 11 mol%), CF_3CO_2Na (81.6 mg, 0.6 mmol, 2.0 equiv.), B_2Pin_2 (228.5 mg, 0.9 mmol, 3.0 equiv.), H_2O (16.2 μL, 0.9 mmol, 3.0 equiv.) and DMF (4 mL). The reaction was carried out under Ar at 80 °C for 12 h. Flash column chromatography (petroleum ether) afforded the product **3r** as a white solid (54 mg, 91%, 93/7 *E/Z*). ^{1}H NMR (400 MHz, $CDCl_3$) δ 7.56-7.43 (m, 4 H), 7.39-7.30 (m, 2 H), 7.25 (dd, J = 9.2, 5.1 Hz, 1 H), 7.11-6.97 (m, 4 H). ^{19}F NMR (376 MHz, $CDCl_3$) δ -114.25. ^{13}C NMR (101 MHz, $CDCl_3$) δ 162.36 (d, J = 247.2 Hz), 133.55 (d, J = 3.4 Hz), 128.53 (d, J = 2.3 Hz), 127.98 (d, J = 7.9 Hz), 127.66, 127.50, 115.60 (d, J = 21.7 Hz), 137.20, 128.70, 126.44. The spectroscopic data correspond to reported data.[29]

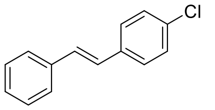

3s

(*E*)-1-chloro-4-styrylbenzene. According to the general procedure, the sealed tube was charged with the corresponding alkyne (0.3 mmol, 1.0 equiv.), $NiBr_2$ (3.3 mg, 0.015 mmol, 5 mol%), **L1** (5.2 mg, 0.033 mmol, 11 mol%), CF_3CO_2Na (81.6 mg, 0.6 mmol, 2.0 equiv.), B_2Pin_2 (228.5 mg, 0.9 mmol, 3.0 equiv.), H_2O (16.2 μL, 0.9 mmol, 3.0 equiv.) and DMF (4 mL). The reaction was carried out under Ar at 80 °C for 12 h. Flash column chromatography (petroleum ether) afforded the product **3s** as a white solid (53.6 mg, 83%, 96/4 *E/Z*). ^{1}H NMR (300 MHz, $CDCl_3$) δ 7.49 (dd, J = 8.3, 1.2 Hz, 2 H), 7.45-7.40 (m, 2 H), 7.37-7.31 (m, 3 H), 7.31-7.23 (m, 2 H), 7.05 (d, J = 1.6 Hz, 2 H). ^{13}C NMR (75 MHz, $CDCl_3$) δ 136.96, 135.82, 133.14, 129.29, 128.81, 128.71, 127.84, 127.63, 127.34, 126.53. The spectroscopic data correspond to reported data.[29]

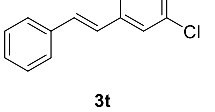

3t

(*E*)-1-chloro-3-styrylbenzene. According to the general procedure, the sealed tube was charged with the corresponding alkyne (0.3 mmol, 1.0 equiv.), $NiBr_2$ (3.3 mg, 0.015 mmol, 5 mol%), **L1** (5.2 mg, 0.033 mmol, 11 mol%), CF_3CO_2Na (81.6 mg, 0.6 mmol, 2.0 equiv.), B_2Pin_2 (228.5 mg, 0.9 mmol, 3.0 equiv.), H_2O (16.2 μL, 0.9 mmol, 3.0 equiv.) and DMF (4 mL). The reaction was carried out under Ar at 80 °C for 12 h. Flash column chromatography (petroleum ether) afforded the product **3t** as a white solid (51 mg, 79%, 96/4 *E/Z*). ^{1}H NMR (400 MHz, $CDCl_3$) δ 7.49 (d, *J* = 6.8 Hz, 3H), 7.35 (t, *J* = 7.3 Hz, 3H), 7.30-7.17 (m, 3H), 7.09 (d, *J* = 16.3 Hz, 1H), 7.00 (d, *J* = 16.3 Hz, 1H). ^{13}C NMR (101 MHz, $CDCl_3$) δ 139.23, 136.80, 134.63, 130.12, 129.83, 128.72, 128.00, 127.45, 127.19, 126.64, 126.28, 124.71. The spectroscopic data correspond to reported data.[31]

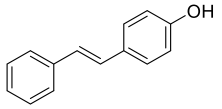

3u

(*E*)-4-styrylphenol. According to the general procedure, the sealed tube was charged with the corresponding alkyne (0.3 mmol, 1.0 equiv.), $NiBr_2$ (3.3 mg, 0.015 mmol, 5 mol%), **L1** (5.2 mg, 0.033 mmol, 11 mol%), CF_3CO_2Na (81.6 mg, 0.6 mmol, 2.0 equiv.), B_2Pin_2 (228.5 mg, 0.9 mmol, 3.0 equiv.), H_2O (16.2 μL, 0.9 mmol, 3.0 equiv.) and DMF (4 mL). The reaction was carried out under Ar at 80 °C for 12 h. Flash column chromatography (petroleum ether : AcOEt = 5:1) afforded the product **3u** as a white solid (56.2 mg, 95%, >99/1 *E/Z*). ^{1}H NMR (400 MHz, $CDCl_3$) δ 7.48 (d, *J* = 7.4 Hz, 2H), 7.41 (d, *J* = 8.5 Hz, 2H), 7.34 (t, *J* = 7.6 Hz, 2H), 7.29-7.19 (m, 1H), 7.05 (d, *J* = 16.3 Hz, 1H), 6.96 (d, *J* = 16.3 Hz, 1H), 6.83 (d, *J* = 8.6 Hz, 2H), 4.86 (s, 1H). ^{13}C NMR (101 MHz, $CDCl_3$) δ 155.26, 137.64, 130.45, 128.64, 128.17, 127.92, 127.25, 126.77, 126.27, 115.63. The spectroscopic data correspond to reported data.[29]

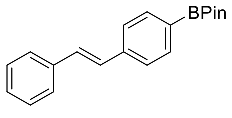

3v

(*E*)-4,4,5,5-tetramethyl-2-(4-styrylphenyl)-1,3,2-dioxaborolane. According to the general procedure, the sealed tube was charged with the corresponding alkyne (0.3 mmol, 1.0 equiv.), $NiBr_2$ (3.3 mg, 0.015 mmol, 5 mol%), **L1** (5.2 mg, 0.033 mmol, 11 mol%), CF_3CO_2Na (81.6 mg, 0.6 mmol, 2.0 equiv.), B_2Pin_2 (228.5 mg, 0.9 mmol, 3.0 equiv.), H_2O (16.2 μL, 0.9 mmol, 3.0 equiv.) and DMF (4 mL). The reaction was carried out under Ar at 80 °C for 12 h. Flash column chromatography (petroleum ether : AcOEt = 40:1) afforded the product **3v** as a yellow solid (59.1 mg, 64%, 85/15 *E/Z*). ^{1}H NMR (400 MHz, $CDCl_3$) δ 7.86-7.76 (m, 1.7H), 7.72-7.62 (m, 0.7H), 7.59-7.46 (m, 3.6H), 7.41-7.31 (m, 2H), 7.28-7.17 (m, 3.3H), 7.16-7.07 (m, 2H), 6.64-6.56 (m, 0.6H), 1.34 (s, 12H), 1.32 (s, 3.9H). ^{13}C NMR (101 MHz, $CDCl_3$) δ 140.17, 140.02, 137.22, 137.09, 135.15, 134.63, 130.92, 130.19, 129.65, 128.87, 128.66, 128.19, 128.15, 127.16, 126.60, 125.78, 83.75, 83.71, 24.86. The spectroscopic data correspond to reported data.[48]

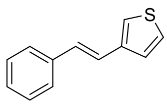

3x

(*E*)-3-styrylthiophene. According to the general procedure, the sealed tube was charged with the corresponding alkyne (0.3 mmol, 1.0 equiv.), $NiBr_2$ (3.3 mg, 0.015 mmol, 5 mol%), **L1** (5.2 mg,

0.033 mmol, 11 mol%), CF_3COONa (81.6 mg, 0.6 mmol, 2.0 equiv.), B_2Pin_2 (228.5 mg, 0.9 mmol, 3.0 equiv.), H_2O (16.2 μL, 0.9 mmol, 3.0 equiv.) and DMF (4 mL). The reaction was carried out under Ar at 80 °C for 12 h. Flash column chromatography (petroleum ether) afforded the product **3x** as a colorless oil (45 mg, 80%, 92/8 *E/Z*). ^{1}H NMR (400 MHz, $CDCl_3$) δ 7.46 (d, *J* = 7.3 Hz, 2 H), 7.38-7.20 (m, 6 H), 7.11 (d, *J* = 16.3 Hz, 1 H), 6.94 (d, *J* = 16.3 Hz, 1 H). ^{13}C NMR (101 MHz, $CDCl_3$) δ 140.09, 137.35, 128.64, 127.42, 126.25, 126.14, 124.91, 122.87, 122.31. The spectroscopic data correspond to reported data.[36]

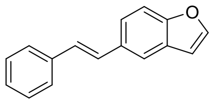

3y

(*E*)-5-styrylbenzofuran. According to the general procedure, the sealed tube was charged with the corresponding alkyne (0.3 mmol, 1.0 equiv.), $NiBr_2$ (3.3 mg, 0.015 mmol, 5 mol%), **L1** (5.2 mg, 0.033 mmol, 11 mol%), CF_3COONa (81.6 mg, 0.6 mmol, 2.0 equiv.), B_2Pin_2 (228.5 mg, 0.9 mmol, 3.0 equiv.), H_2O (16.2 μL, 0.9 mmol, 3.0 equiv.) and DMF (4 mL). The reaction was carried out under Ar at 80 °C for 12 h. Flash column chromatography (petroleum ether) afforded the product **3y** as a white solid (64.9 mg, 98%, 97/3 *E/Z*). ^{1}H NMR (400 MHz, $CDCl_3$) δ 7.69 (s, 1 H), 7.59 (d, *J* = 2.1 Hz, 1 H), 7.55-7.41 (m, 4 H), 7.34 (t, *J* = 7.6 Hz, 2 H), 7.22 (d, *J* = 9.9 Hz, 1 H), 7.19 (d, *J* = 13.0 Hz, 1 H), 7.07 (d, *J* = 16.3 Hz, 1 H), 6.74 (d, *J* = 1.8 Hz, 1 H). ^{13}C NMR (101 MHz, $CDCl_3$) δ 154.68, 145.50, 137.52, 132.47, 128.92, 128.65, 127.87, 127.69, 127.36, 126.34, 123.00, 119.21, 111.51, 106.66. The spectroscopic data correspond to reported data.[31]

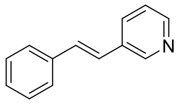

3z

(*E*)-3-styrylpyridine. According to the general procedure, the sealed tube was charged with the corresponding alkyne (0.3 mmol, 1.0 equiv.), $NiBr_2$ (3.3 mg, 0.015 mmol, 5 mol%), **L1** (5.2 mg, 0.033 mmol, 11 mol%), CF_3COONa (81.6 mg, 0.6 mmol, 2.0 equiv.), B_2Pin_2 (228.5 mg, 0.9 mmol, 3.0 equiv.), H_2O (16.2 μL, 0.9 mmol, 3.0 equiv.) and DMF (4 mL). The reaction was carried out under Ar at 80 °C for 12 h, the result solution was diluted with ethyl acetate, washed with water and concentrated in vacuum. Flash column chromatography (petroleum ether : AcOEt = 10:1) afforded the product **3z** as a white solid (38.2 mg, 71%, 94/6 *E/Z*). ^{1}H NMR (400 MHz, $CDCl_3$) δ 8.73 (s, 1 H), 8.49 (d, *J* = 4.0 Hz, 1 H), 7.85 (d, *J* = 7.9 Hz, 1 H), 7.54 (d, *J* = 7.5 Hz, 2 H), 7.39 (t, *J* = 7.5 Hz, 2 H), 7.34-7.28 (m, 2 H), 7.18 (d, *J* = 16.4 Hz, 1 H), 7.08 (d, *J* = 16.4 Hz, 1 H). ^{13}C NMR (101 MHz, $CDCl_3$) δ 148.34, 136.62, 133.11, 132.83, 130.99, 128.79, 128.25, 126.68, 124.81, 123.59. The spectroscopic data correspond to reported data.[36]

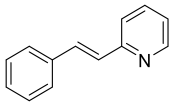

3aa

(*E*)-2-styrylpyridine. According to the general procedure, the sealed tube was charged with the corresponding alkyne (0.3 mmol, 1.0 equiv.), $NiBr_2$ (3.3 mg, 0.015 mmol, 5 mol%), **L1** (5.2 mg, 0.033 mmol, 11 mol%), CF_3COONa (81.6 mg, 0.6 mmol, 2.0 equiv.), B_2Pin_2 (228.5 mg, 0.9 mmol, 3.0 equiv.), H_2O (16.2 μL, 0.9 mmol, 3.0 equiv.) and DMF (4 mL). The reaction was carried out

under Ar at 80 °C for 12 h, the result solution was diluted with ethyl acetate, washed with water and concentrated in vacuum. Flash column chromatography (petroleum ether : AcOEt = 10:1) afforded the product **3aa** as a yellow solid (31.6 mg, 62%, 98/2 *E/Z*). ^{1}H NMR (300 MHz, $CDCl_3$) δ 8.61 (d, *J* = 4.0 Hz, 1 H), 7.70-7.54 (m, 4 H), 7.42-7.34 (m, 3 H), 7.33-7.25 (m, 1 H), 7.21-7.11 (m, 2 H). ^{13}C NMR (101 MHz, $CDCl_3$) δ 155.62, 149.64, 136.65, 136.46, 132.71, 128.68, 128.28, 127.95, 127.07, 122.03, 122.00. The spectroscopic data correspond to reported data.[55]

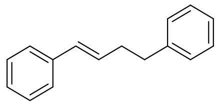

3hh

(*E*)-but-1-ene-1,4-diyldibenzene. According to the general procedure, the sealed tube was charged with the corresponding alkyne (0.3 mmol, 1.0 equiv.), $NiBr_2$ (3.3 mg, 0.015 mmol, 5 mol%), **L1** (5.2 mg, 0.033 mmol, 11 mol%), CF_3COONa (81.6 mg, 0.6 mmol, 2.0 equiv.), B_2Pin_2 (228.5 mg, 0.9 mmol, 3.0 equiv.), H_2O (16.2 μL, 0.9 mmol, 3.0 equiv.) and DMF (4 mL). The reaction was carried out under Ar at 80 °C for 12 h. Flash column chromatography (petroleum ether) afforded the product **3hh** as a white solid (33.1 mg, 53%, 80/20 *E/Z*). ^{1}H NMR (300 MHz, $CDCl_3$) δ 7.41-7.10 (m, 10 H), 6.41 (d, *J* = 15.9 Hz, 1 H), 6.25 (dt, *J* = 15.8, 6.6 Hz, 1 H), 2.85-2.74 (m, 2 H), 2.53 (dd, *J* = 15.0, 7.0 Hz, 2 H). ^{13}C NMR (101 MHz, $CDCl_3$) δ 141.75, 137.73, 130.38, 129.95, 128.46, 128.34, 126.91, 125.98, 125.87, 35.87, 34.83. The spectroscopic data correspond to reported data.[56]

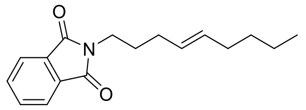

3ii

(*E*)-2-(non-4-en-1-yl)isoindoline-1,3-dione. According to the general procedure, the sealed tube was charged with the corresponding alkyne (0.3 mmol, 1.0 equiv.), NiBr2 (3.3 mg, 0.015 mmol, 5 mol%), **L1** (5.2 mg, 0.033 mmol, 11 mol%), CF3COONa (81.6 mg, 0.6 mmol, 2.0 equiv.), B2Pin2 (228.5 mg, 0.9 mmol, 3.0 equiv.), H2O (16.2 μL, 0.9 mmol, 3.0 equiv.) and DMF (4 mL). The reaction was carried out under Ar at 80 oC for 12 h, the result solution was diluted with ethyl acetate, washed with water and concentrated in vacuum. Flash column chromatography (petroleum ether : AcOEt = 35:1) afforded the mixture of **2ii** and **3ii** as a colorless oil (19.9 mg, 24%, 63/37 E/Z). 1H NMR (400 MHz, CDCl3) δ 7.92-7.81 (m, 2H), 7.75-7.68 (m, 2H), 5.63-5.16 (m, 2H), 3.92-3.53 (m, 2H), 2.27-1.89 (m, 4H), 1.80-1.71 (m, 2H), 1.38-1.24 (m, 4H), 0.99-0.81 (m, 3H). 13C NMR (101 MHz, CDCl3) δ 168.39, 168.36, 133.80, 133.79, 132.22, 132.21, 131.51, 131.06, 128.49, 128.04, 123.13, 123.11, 37.77, 37.68, 32.17, 31.80, 31.63, 29.88, 28.52, 28.27, 26.93, 24.65, 22.29, 22.14, 13.92, 13.89. The spectroscopic data correspond to reported data.[57]

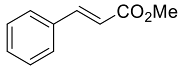

3jj

Methyl cinnamate. According to the general procedure, the sealed tube was charged with the corresponding alkyne (0.3 mmol, 1.0 equiv.), $NiBr_2$ (3.3 mg, 0.015 mmol, 5 mol%), **L1** (5.2 mg, 0.033 mmol, 11 mol%), CF_3COONa (81.6 mg, 0.6 mmol, 2.0 equiv.), B_2Pin_2 (228.5 mg, 0.9 mmol, 3.0 equiv.), H_2O (16.2 μL, 0.9 mmol, 3.0 equiv.) and DMF (4 mL). The reaction was carried out under Ar at 80 °C for 12 h. Flash column chromatography (petroleum ether : AcOEt = 40:1) afforded the product **3jj** as a colorless oil (21.1 mg, 43%, >99/1 *E/Z*). ^{1}H NMR (400 MHz, $CDCl_3$) δ 7.70 (d,

J = 16.0 Hz, 1 H), 7.52 (d, *J* = 3.3 Hz, 2 H), 7.38 (s, 3 H), 6.45 (d, *J* = 16.0 Hz, 1 H), 3.81 (s, 3 H). ^{13}C NMR (101 MHz, $CDCl_3$) δ 167.37, 144.83, 134.39, 130.25, 128.86, 128.04, 117.81, 51.63. The spectroscopic data correspond to reported data.[29]

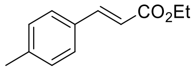

3kk

Ethyl (*E*)-3-(p-tolyl)acrylate. According to the general procedure, the sealed tube was charged with the corresponding alkyne (0.3 mmol, 1.0 equiv.), $NiBr_2$ (3.3 mg, 0.015 mmol, 5 mol%), **L1** (5.2 mg, 0.033 mmol, 11 mol%), CF_3COONa (81.6 mg, 0.6 mmol, 2.0 equiv.), B_2Pin_2 (228.5 mg, 0.9 mmol, 3.0 equiv.), H_2O (16.2 μL, 0.9 mmol, 3.0 equiv.) and DMF (4 mL). The reaction was carried out under Ar at 80 °C for 12 h. Flash column chromatography (petroleum ether : AcOEt = 30:1) afforded the product **3kk** as a colorless oil (29.5 mg, 52%, >99/1 *E/Z*). ^{1}H NMR (400 MHz, $CDCl_3$) δ 7.66 (d, *J* = 15.9 Hz, 1 H), 7.41 (d, *J* = 7.2 Hz, 2 H), 7.29-7.06 (m, 2 H), 6.39 (d, *J* = 15.9 Hz, 1 H), 4.26 (d, *J* = 6.8 Hz, 2 H), 2.36 (s, 3 H), 1.33 (t, *J* = 6.6 Hz, 3 H). ^{13}C NMR (101 MHz, $CDCl_3$) δ 167.13, 144.53, 140.55, 131.74, 129.56, 128.00, 117.18, 60.34, 21.39, 14.30. The spectroscopic data correspond to reported data.[58]

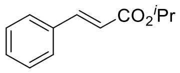

3ll

Isopropyl cinnamate. According to the general procedure, the sealed tube was charged with the corresponding alkyne (0.3 mmol, 1.0 equiv.), $NiBr_2$ (3.3 mg, 0.015 mmol, 5 mol%), **L1** (5.2 mg, 0.033 mmol, 11 mol%), CF_3COONa (81.6 mg, 0.6 mmol, 2.0 equiv.), B_2Pin_2 (228.5 mg, 0.9 mmol, 3.0 equiv.), H_2O (16.2 μL, 0.9 mmol, 3.0 equiv.) and DMF (4 mL). The reaction was carried out under Ar at 80 °C for 12 h. Flash column chromatography (petroleum ether : AcOEt = 30:1) afforded the product **3ll** as a colorless oil (26.3 mg, 46%, >99/1 *E/Z*). ^{1}H NMR (300 MHz, $CDCl_3$) δ 7.67 (d, *J* = 16.0 Hz, 1 H), 7.52 (dd, *J* = 6.7, 3.0 Hz, 2 H), 7.42-7.34 (m, 3 H), 6.42 (d, *J* = 16.0 Hz, 1 H), 5.22 – 5.08 (m, 1 H), 1.32 (d, *J* = 6.3 Hz, 6 H). ^{13}C NMR (101 MHz, $CDCl_3$) δ 166.48, 144.26, 134.56, 130.09, 128.83, 127.99, 118.84, 67.77, 21.93. The spectroscopic data correspond to reported data.[59]

2.18 Procedure for Transfer Semihydrogenation of Alkyne 1mm

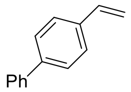

6

4-vinyl-1,1'-biphenyl. To a sealed tube were added alkyne **1mm** (53.5 mg, 0.3 mmol), $NiBr_2$ (3.3 mg, 0.015 mmol, 5 mol%), **L3** (7.1 mg, 0.033 mmol, 11 mol%), $PhCO_2Na$ (43.2 mg, 0.3 mmol, 1.0 equiv.) and B_2Pin_2 (228.5 mg, 0.9 mmol, 3.0 equiv.). The flask was evacuated and refilled with argon, followed by the addition of H_2O (16.2 μL, 0.9 mmol, 3.0 equiv.) and DMF (4 mL). The mixture was stirred at 80 °C for 12 h until the reaction was completed as monitored by TLC. The resultant solution was diluted with ethyl acetate, washed with HCl aqueous solution (1 M) and concentrated in vacuum. The residue was purified by chromatography on silica gel, eluting with petroleum ether to give alkene product **6** as a white solid (48.1 mg, 77%). ^{1}H NMR (400 MHz, $CDCl_3$) δ 7.67 – 7.56 (m, 4 H), 7.51 (d, *J* = 8.2 Hz, 2 H), 7.46 (t, *J* = 7.6 Hz, 2 H), 7.37 (t, *J* = 7.3 Hz, 1 H), 6.79 (dd, *J* = 17.6, 10.9 Hz, 1 H), 5.82 (d, *J* = 17.6 Hz, 1 H). ^{13}C NMR (101 MHz, $CDCl_3$) δ 140.73, 140.59,

136.61, 136.42, 128.76, 127.29, 127.21, 126.95, 126.63, 113.85. The spectroscopic data correspond to reported data.[60]

2.19 Procedure for Transfer Semihydrogenation of Alkyne 1nn

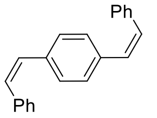

7

1,4-di((*Z*)-styryl)benzene. To a sealed tube were added alkyne **1nn** (83.5 mg, 0.3 mmol), $NiBr_2$ (6.6 mg, 0.03 mmol, 10 mol%), **L3** (14.2 mg, 0.066 mmol, 22 mol%), $PhCO_2Na$ (86.5 mg, 0.6 mmol, 2.0 equiv.), B_2Pin_2 (457 mg, 1.8 mmol, 6.0 equiv.). The flask was evacuated and refilled with argon, followed by the addition of H_2O (32.4 µL, 1.8 mmol, 6.0 equiv.) and DMF (8 mL). The mixture was stirred at 80 °C for 12 h until the reaction was completed as monitored by TLC. The resultant solution was diluted with ethyl acetate, washed with HCl aqueous solution (1 M) and concentrated in vacuum. The residue was detected by GC to report *Z*/*E* ratios, and purified by chromatography on silica gel (300-400 mesh), eluting with petroleum ether to give alkene product **7** as a white solid (63.2 mg, 76%, *ZZ*: *ZE*: *EE* = 81: 11: 8). ^{1}H NMR (400 MHz, $CDCl_3$) δ 7.21 (ddd, *J* = 17.6, 11.3, 7.1 Hz, 10 H), 7.10 (s, 4 H), 6.54 (q, *J* = 12.2 Hz, 4 H). ^{13}C NMR (101 MHz, $CDCl_3$) δ 137.31, 136.06, 130.28, 129.94, 128.83, 128.73, 128.17, 127.10. The spectroscopic data correspond to reported data.[33]

2.20 Procedure for Transfer Semihydrogenation of Alkyne 1oo

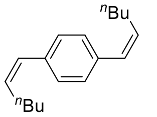

8

1,4-di((*Z*)-hex-1-en-1-yl)benzene. To a sealed tube were added alkyne **1oo** (71.5 mg, 0.3 mmol), $NiBr_2$ (6.6 mg, 0.03 mmol, 10 mol%), **L3** (14.2 mg, 0.066 mmol, 22 mol%), $PhCO_2Na$ (86.5 mg, 0.6 mmol, 2.0 equiv.), B_2Pin_2 (457 mg, 1.8 mmol, 6.0 equiv.). The flask was evacuated and refilled with argon, followed by the addition of H_2O (32.4 µL, 1.8 mmol, 6.0 equiv.) and DMF (8 mL). The mixture was stirred at 100 °C for 12 h until the reaction was completed as monitored by TLC. The resultant solution was diluted with ethyl acetate, washed with HCl aqueous solution (1 M) and concentrated in vacuum. The residue was detected by GC to report *Z*/*E* ratios, and purified by chromatography on silica gel (300-400 mesh), eluting with petroleum ether to give alkene product **8** as a colorless oil (57.4 mg, 79%, *ZZ*: *EE* = 86:14). ^{1}H NMR (400 MHz, $CDCl_3$) δ 7.35-7.17 (m, 4 H), 6.38 (d, *J* = 11.6 Hz, 2 H), 5.65 (dt, *J* = 11.7, 7.3 Hz, 2 H), 2.35 (dd, *J* = 13.8, 6.9 Hz, 4 H), 1.44 (dd, *J* = 14.9, 7.7 Hz, 4 H), 1.36 (dd, *J* = 14.6, 7.1 Hz, 4 H), 0.90 (t, *J* = 7.2 Hz, 6 H). ^{13}C NMR (101 MHz, $CDCl_3$) δ 136.01, 133.13, 128.91, 128.52, 128.44, 125.62, 32.18, 28.49, 22.43, 13.96. HRMS (EI) Calcd. for $C_{18}H_{26}^+$: 242.2029, Found: 242.2027.

2.21 Procedure for Transfer Semihydrogenation of Alkyne 1pp

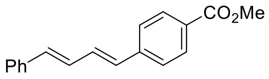

9

Methyl 4-((1*E*,3*E*)-4-phenylbuta-1,3-dien-1-yl)benzoate. To a sealed tube were added alkyne **1pp**

(78.7 mg, 0.3 mmol), $NiBr_2$ (3.3 mg, 0.015 mmol, 5 mol%), **L3** (7.1 mg, 0.033 mmol, 11 mol%), $PhCO_2Na$ (43.2 mg, 0.3 mmol, 1.0 equiv.) and B_2Pin_2 (228.5 mg, 0.9 mmol, 3.0 equiv.). The flask was evacuated and refilled with argon, followed by the addition of H_2O (16.2 μL, 0.9 mmol, 3.0 equiv.) and DMF (4 mL). The mixture was stirred at 100 °C for 12 h until the reaction was completed as monitored by TLC. The resultant solution was diluted with ethyl acetate, washed with HCl aqueous solution (1 M) and concentrated in vacuum. The residue was detected by GC to report *Z*/*E* ratios, and purified by chromatography on silica gel (Petroleum ether : AcOEt = 40:1) to give alkene product **9** as a white solid (50 mg, 63%, *E*: *Z* = 96:4). ^{1}H NMR (400 MHz, $CDCl_3$) δ 7.99 (d, *J* = 8.3 Hz, 2 H), 7.52-7.42 (m, 4 H), 7.34 (t, *J* = 7.5 Hz, 2 H), 7.25 (t, *J* = 7.2 Hz, 1 H), 7.00 (ddd, *J* = 37.0, 15.1, 10.6 Hz, 2 H), 6.70 (dd, *J* = 21.8, 15.1 Hz, 2 H), 3.91 (s, 3 H). ^{13}C NMR (101 MHz, $CDCl_3$) δ 166.84, 141.86, 137.04, 134.51, 131.75, 131.52, 129.99, 128.78, 128.76, 128.70, 127.94, 126.56, 126.13, 52.01. The spectroscopic data correspond to reported data.[61]

2.22 Procedure for Transfer Semihydrogenation of Alkyne 1qq

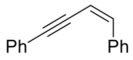

10

(*Z*)-but-1-en-3-yne-1,4-diyldibenzene. To a sealed tube were added alkyne **1qq** (60.7 mg, 0.3 mmol), $NiBr_2$ (3.3 mg, 0.015 mmol, 5 mol%), **L1** (5.2 mg, 0.015 mmol, 11 mol%), CF_3COONa (40.8 mg, 0.3 mmol, 1.0 equiv.) and B_2Pin_2 (228.5 mg, 0.9 mmol, 3.0 equiv.). The flask was evacuated and refilled with argon, followed by the addition of H_2O (16.2 μL, 0.9 mmol, 3.0 equiv.) and DMF (4 mL). The mixture was stirred at 80 °C for 12 h until the reaction was completed as monitored by TLC. The resultant solution was diluted with ethyl acetate, washed with HCl aqueous solution (1 M) and concentrated in vacuum. The residue was detected by GC to report *Z*/*E* ratios, and purified by chromatography on silica gel, eluting with petroleum ether to give alkene product **10** as a colorless oil (33.1 mg, 53%, *Z*: *E* = 93:7). ^{1}H NMR (400 MHz, $CDCl_3$) δ 7.92 (d, *J* = 7.4 Hz, 2 H), 7.52-7.47 (m, 2 H), 7.44-7.27 (m, 7 H), 6.70 (d, *J* = 11.9 Hz, 1 H), 5.92 (d, *J* = 11.9 Hz, 1 H). ^{13}C NMR (101 MHz, $CDCl_3$) δ 138.66, 136.57, 131.45, 128.76, 128.49, 128.40, 128.35, 128.28, 123.48, 107.40, 95.84, 88.25. The spectroscopic data correspond to reported data.[31]

3. Supplementary References

1. González, M. J., Bauer, F. & Breit, B. Cobalt-Catalyzed Hydroboration of Terminal and Internal Alkynes. *Org. Lett.* **23**, 8199-8203 (2021).
2. Liang, M. Z. & Meek, S. J. Catalytic Enantioselective Synthesis of 1,4-Keto-Alkenylboronate Esters and 1,4-Dicarbonyls. *Angew. Chem. Int. Ed.* **58**, 14234-14239 (2019).
3. Wen, H. et al. Stereoselective Synthesis of Trisubstituted Alkenes via CobaltCatalyzed Double Dehydrogenative Borylations of 1-Alkenes. *ACS Catal.* **7**, 6419-6425 (2017).
4. Peng, S., Liu, G. & Huang, Z. Mixed Diboration of Alkynes Catalyzed by LiOH: Regio- and Stereoselective Synthesis of cis-1,2-Diborylalkenes. *Org. Lett.* **20**, 7363-7366 (2018).
5. Liu, X., Ming, W., Friedrich, A., Kerner, F. & Marder, T. B. Copper-Catalyzed Triboration of Terminal Alkynes Using B_2pin_2: Efficient Synthesis of 1,1,2-Triborylalkenes. *Angew. Chem. Int. Ed.* **59**, 304-309 (2020).
6. Zhang, M., Yao, Y., Stang, P. J. & Zhao, W. Divergent and Stereoselective Synthesis of

Tetraarylethylenes from Vinylboronates. *Angew. Chem. Int. Ed.* **59**, 20090-20098 (2020).

7. Day, C. S., Somerville, R. J. & Martin, R. Deciphering the Dichotomy Exerted by Zn(II) in the Catalytic sp^2 C-O Bond Functionalization of Aryl Esters at the Molecular Level. *Nat. Catal.* **4**, 124-133 (2021).
8. Durr, A. B., Fisher, H. C., Kalvet, I., Truong, K. N. & Schoenebeck, F. Divergent Reactivity of a Dinuclear (NHC)Nickel(I) Catalyst versus Nickel(0) Enables Chemoselective Trifluoromethylselenolation. *Angew. Chem. Int. Ed.* 5**6**, 13431-13435 (2017).
9. Mohadjer Beromi, M. et al. Mechanistic Study of an Improved Ni Precatalyst for Suzuki-Miyaura Reactions of Aryl Sulfamates: Understanding the Role of Ni(I) Species. *J. Am. Chem. Soc.* **139**, 922-936 (2017).
10. Zarate, C., Yang, H., Bezdek, M. J., Hesk, D. & Chirik, P. J. Ni(I)-X Complexes Bearing a Bulky alpha-Diimine Ligand: Synthesis, Structure, and Superior Catalytic Performance in the Hydrogen Isotope Exchange in Pharmaceuticals. *J. Am. Chem. Soc.* **141**, 5034-5044 (2019).
11. Somerville, R. J. et al. Ni(I)-Alkyl Complexes Bearing Phenanthroline Ligands: Experimental Evidence for CO_2 Insertion at Ni(I) Centers. *J. Am. Chem. Soc.* **142**, 10936-10941 (2020).
12. Sheng, J. et al. Diversity-Oriented Synthesis of Aliphatic Fluorides via Reductive $C(sp^3)$-$C(sp^3)$ Cross-Coupling Fluoroalkylation. *Angew. Chem. Int. Ed.* **60**, 15020-15027 (2021).
13. Yanagi, T., Somerville, R. J., Nogi, K., Martin, R. & Yorimitsu, H. Ni-Catalyzed Carboxylation of $C(sp^2)$-S Bonds with CO_2: Evidence for the Multifaceted Role of Zn. *ACS Catal.* **10**, 2117-2123 (2020).
14. Frisch, M. J. et al. *Gaussian 09*, Revision D.01; Gaussian, Inc.: Wallingford, CT (2009).
15. Becke, A. D. Density-Functional Thermochemistry. III. The Role of Exact Exchange. *J. Chem. Phys.* **98**, 5648-5652 (1993).
16. Stephens, P. J., Devlin, F. J., Chabalowski, C. F. & Frisch, M. J. Ab Initio Calculation of Vibrational Absorption and Circular Dichroism Spectra Using Density Functional Force Fields. *J. Phys. Chem.* **98**, 11623-11627 (1994).
17. Lee, C., Yang, W. & Parr, R. G. Development of the Colic-Salvetti Correlation-Energy Formula into a Functional of the Electron Density. *Phys. Rev. B*, **37**, 785-789 (1988).
18. Dunning Jr, T. H. & Hay, P. J. In Modern Theoretical Chemistry Vol. 3 (Plenum, New York, 1977).
19. Fuentealba, P., Preuss, H., Stoll, H. & Von Szentpály, L. A Proper Account of Core-Polarization with Pseudopotentials: Single Valence-Electron Alkali Compounds. *Chem. Phys. Lett.* **89**, 418 (1982).
20. Marenich, A. V., Cramer, C. J. & Truhlar, D. G. Universal Solvation Model Based on Solute Electron Density and on a Continuum Model of the Solvent Defined by the Bulk Dielectric Constant and Atomic Surface Tensions. *J. Phys. Chem. B* **113**, 6378-6396 (2009).
21. Truhlar Donald, G. & Gordon Mark, S. From Force Fields to Dynamics: Classical and Quantal Paths. *Science* **249**, 491-498 (1990).
22. Gonzalez, C. & Bernhard Schlegel H. An Improved Algorithm for Reaction Path Following. *J. Chem. Phys.* **90**, 2154-2161 (1989).
23. Similar process was proposed for vinyl Pd species in previous work: Cheng, C. & Zhang, Y. Palladium-Catalyzed anti-Carbosilylation of Alkynes to Access Isoquinolinone-Containing Exocyclic Vinylsilanes. *Org. Lett.* **23**, 5772-5776 (2021).
24. Sperger, T., Le, C. M., Lautens, M. & Schoenebeck, F. Mechanistic insights on the Pd-catalyzed

addition of C-X bonds across alkynes-a combined experimental and computational study. *Chem. Sci.* **8**, 2914-2922 (2017).

25. Zhang, X., Xie, X. & Liu, Y. Nickel-catalyzed cyclization of alkyne-nitriles with organoboronic acids involving anti-carbometalation of alkynes. *Chem. Sci.* **7**, 5815-5820 (2016).
26. Zhou, Z., Chen, J., Chen, H. & Kong, W. Stereoselective synthesis of pentasubstituted 1,3-dienes via Ni-catalyzed reductive coupling of unsymmetrical internal alkynes. *Chem. Sci.* **11**, 10204-10211 (2020).
27. Zhou, Z., Liu, W. & Kong, W. Ni-Catalyzed Reductive Antiarylative Cyclization of Alkynones. *Org. Lett.* **22**, 6982-6987 (2020).
28. Liu, Y. & Du, H. An Alken e-Promoted Borane-Catalyzed Highly Stereoselective Hydrogenation of Alkynes to Give *Z*- and *E*-Alkenes. *Chem. Eur. J.* **21**,3495-3501 (2015).
29. Li, K. K. et al. Cobalt Catalyzed Stereodivergent semi-hydrogenation of Alkynes using H_2O as the Hydrogen Source. *Chem. Commun*. **55**, 5663-5666 (2019).
30. Hamasaka, G. et al. Arylation of Terminal Alkynes by Aryl Iodides Catalyzed by a Parts per Million Loading of Palladium Acetate. *ACS Catal.* **9**, 11640-11646 (2019)
31. Wang, Y., Huang, Z. & Huang, Z. Catalyst as Colour Indicator for Endpoint Detection to enable Selective Alkyne *trans*-hydrogenation with Ethanol. *Nat. Catal.* **2**, 529-536 (2019).
32. Tian, W. F. et al. *Cis*-Selective Transfer semihydrogenation of Alkynes by Merging Visible-Light Catalysis with Cobalt Catalysis. *Adv. Synth. Catal.* **362**, 1032-1038 (2020).
33. Sklyaruk, J. et al. Methanol as the Hydrogen Source in the Selective Transfer Hydrogenation of Alkynes Enabled by a Manganese Pincer Complex. *Org. Lett.* **22**, 6067-6971 (2020).
34. Eisele, P., Ullwer, F., Scholz, S. & Plietker, B. Mild, Selective Ru-Catalyzed Deuteration Using D_2O as a Deuterium Source. *Chem. Eur. J.* **25**,16550-16554 (2019).
35. Han, X. et al. Copper-catalysed, Diboron-mediated *cis*-dideuterated semihydrogenation of Alkynes with Heavy Water. *Chem. Commun.* **55**, 6922-6925 (2019).
36. Luo, X. et al. Xanthate-mediated Synthesis of (*E*)-alkenes by Semi-hydrogenation of Alkynes using Water as the Hydrogen Donor. *Chem. Commun.* **55**, 2170-2173 (2019).
37. Song, Z., Zhang, C. & Ye, S. Visible Light Promoted Coupling of Alkynyl Bromides and Hantzsch Esters for the Synthesis of Internal Alkynes. *Org. Biomol. Chem.* **17**, 181-185 (2019).
38. Berthold, D. et al. Chemo-, Regio-, and Enantioselective Rhodium-Catalyzed Allylation of Triazoles with Internal Alkynes and Terminal Allenes. *Org. Lett.* **20**, 598-601 (2018)
39. Gregori, B. J. et al. Stereoselective Chromium-Catalyzed semi-Hydrogenation of Alkynes. *ChemCatChem* **12**, 5359-5363 (2020).
40. Gao, Y. et al. Cobalt(II)-catalyzed Hydroarylation of 1,3-diynes and Internal Alkynes with Picolinamides Promoted by Alcohol. *Chem. Commun.* **56**, 14231-14234 (2020).
41. Fritzemeier, R. G. et al. Organocatalytic *trans* Phosphinoboration of Internal Alkynes. *Angew. Chem. Int. Ed.* **59**, 14358-14362 (2020).
42. Wang, B. et al. Hydromagnesiation of 1,3-Enynes by Magnesium Hydride for Synthesis of *Tri*- and *Tetra*-substituted Allenes. *Angew. Chem. Int. Ed.* **60**, 217-221 (2021).
43. Lu, B., Li, C. & Zhang, L. Gold-catalyzed Highly Regioselective Oxidation of C-C Triple Bonds without Acid Additives: Propargyl Moieties as Masked α,β-unsaturated Carbonyls. *J. Am. Chem. Soc.* **132**,14070-14072 (2010).
44. Zhang, Y., Li, B. & Liu, S. Y. Pd-Senphos Catalyzed *trans*-Selective Cyanoboration of 1,3-Enynes. *Angew. Chem. Int. Ed.* **59**, 15928-15932 (2020).

45. Chen, M., Sun, N., Chen, Y. & Liu, Y. A New Mild Nitrene Transfer Reagent in Gold Catalysis: Highly Efficient Synthesis of Functionalized Oxazoles. *Chem. Commun.* **52**, 6324-6327 (2016).
46. Xu, J. et al. Photocatalyst-Free Visible Light Promoted $E \rightarrow Z$ Isomerization of Alkenes. *Green Chem.* **22**, 2739-2743 (2020).
47. Tiecco, M. & Tingoli, M. Regiochemistry and Stereochemistry of Nickel-promoted, Carbon-carbon Bond-forming Reactions of Cyclic Sulfur Compounds. *J. Org. Chem.* **50**, 3828-3831 (1985).
48. Murugesan, K. et al. Nickel-Catalyzed Stereodivergent Synthesis of E- and Z-Alkenes by Hydrogenation of Alkynes. *ChemSusChem* **12**, 3363-3369 (2019).
49. Lai, S. Z. et al. Photoinduced Deaminative Coupling of Alkylpyridium Salts with Terminal Arylalkynes. *J. Org. Chem.* **85**, 15638-15644 (2020)
50. Kancherla, R. et al. Oxidative Addition to Palladium(0) Made Easy through Photoexcited-State Metal Catalysis: Experiment and Computation. *Angew. Chem. Int. Ed.* **58**, 3412-3416 (2019).
51. Maccoss, R. N., Balskus, E. P. & Ley, S. V. A Sequential *tetra-n*-propylammonium Perruthenate (TPAP)-Wittig Oxidation Olefination Protocol. *Tetrahedron Lett.* **44**, 7779-7781 (2003).
52. Moran, M. J. et al. Copper(0) nanoparticle catalyzed Z-Selective Transfer Semihydrogenation of Internal Alkynes. *Adv. Synth. Catal.* **363**, 2850-2860 (2021).
53. Bhunia, A. & Studer, A. Synthesis of Highly Substituted Arenes via Cyclohexadiene–Alkene C-H Cross Coupling and Aromatization. *ACS Catal.* **8**, 1213-1217 (2018).
54. Li, Q., Zhou, T. & Yang, H. Encapsulation of Hoveyda–Grubbs^{2nd} Catalyst within Yolk–Shell Structured Silica for Olefin Metathesis. *ACS Catal*. **5**, 2225-2231 (2015).
55. Li, Q. Q. et al. Direct Wittig Olefination of Alcohols. *J. Org. Chem.* **83**, 296-302 (2018).
56. Zhang, Y. L. et al. Vinyl Sulfonium Salts as the Radical Acceptor for Metal-Free Decarboxylative Alkenylation. *Org. Lett.* **22**, 7768-7772 (2020).
57. Hazelden, I. R., Carmona, R. C., Langer, T., Pringle, P. G. & Bower, J. F. Pyrrolidines and Piperidines by Ligand-Enabled Aza-Heck Cyclizations and Cascades of N-(Pentafluorobenzoyloxy)carbamates. *Angew. Chem. Int. Ed.* **57**, 5124-5128 (2018).
58. Xu, W. et al. Palladium Catalyst Immobilized on Functionalized Microporous Organic Polymers for C-C Coupling Reactions. *RSC Adv*. **9**, 34595-34600 (2019).
59. Daub, M. E. et al. Enantioselective [2+2] Cycloadditions of Cinnamate Esters: Generalizing Lewis Acid Catalysis of Triplet Energy Transfer. *J. Am. Chem. Soc.* **141**, 9543-9547 (2019).
60. Li, X. et al. Copper-Catalyzed Defluorinative Hydroarylation of Alkenes with Polyfluoroarenes. *Angew. Chem. Int. Ed.* **59**, 23056-23060 (2020).
61. Liu, J. et al. Preparation of Vinyl Arenes by Nickel-Catalyzed Reductive Coupling of Aryl Halides with Vinyl Bromides. *Angew. Chem. Int. Ed.* **55**, 15544-15548 (2016).
