# Supplementary material for: Modulation of metal species as control point for Ni-catalyzed stereodivergent semihydrogenation of alkynes with water
Source: Nat Commun. 2023 Mar 24;14:1655. doi: 10.1038/s41467-023-37022-w (PMC10039052; doi:10.1038/s41467-023-37022-w)
Supplement: Supplementary file 3 — Description of Additional Supplementary Files [file 41467_2023_37022_MOESM3_ESM.docx]

File Name: Supplementary Data 1

Description: The data of the imaginary frequencies, free energies and coordinates of the optimized structures.

File Name: Supplementary Data 2

Description: The ^1^H, ^19^F, ^13^C NMR Spectra.
