# Supplementary material for: Modulation of metal species as control point for Ni-catalyzed stereodivergent semihydrogenation of alkynes with water
Source: Nat Commun. 2023 Mar 24;14:1655. doi: 10.1038/s41467-023-37022-w (PMC10039052; doi:10.1038/s41467-023-37022-w)
Supplement: Supplementary file 4 — Supplementary Dataset 1 [file 41467_2023_37022_MOESM4_ESM.docx]

**SUPPLEMENTARY DATA** **1**

**Modulation of metal species as control point for Ni-catalyzed stereodivergent semihydrogenation of alkynes with water**

Yuanqi Wu^1^, Yuhui Ao^1^, Zhiming Li^2^*, Chunhui Liu^3^, Jinbo Zhao^1^, Wenyu Gao^1^, Xuemeng Li^1^, Hui Wang^1^, Yongsheng Liu^1^ & Yu Liu^1^*

^1^Jilin Provincial Key Laboratory of Carbon Fiber Development and Application, College of Chemistry and Life Science, Advanced Institute of Materials Science, Changchun University of Technology, 130012 Changchun, PR China. ^2^Department of Chemistry, Fudan University, 200438 Shanghai, PR China. ^3^College of Chemical and Materials Engineering, Xuchang University, 461000 Xuchang, PR China. ^✉^email: zmli@fudan.edu.cn; yuliu@ccut.edu.cn

## Density functional theory (DFT) calculations

**Supplementary Data**. The imaginary frequencies, free energies and coordinates of the optimized structures

**A**

-693.16858 a.u. (zero imaginary freq)

C -3.16661900 -2.76260000 -0.89713200

C -1.90596500 -3.33931300 -1.04072200

N -0.88768800 -1.28354600 -0.36385900

C -2.10753300 -0.70891600 -0.22093900

N -0.88769600 1.28352900 0.36387900

H -4.06423600 -3.33666300 -1.10517500

H -1.78349500 -4.36844000 -1.35985200

Ni 0.68081200 0.00000100 0.00000500

C -3.27149200 -1.43652600 -0.48109000

H -4.24729800 -0.98081900 -0.36694300

C -0.78735200 -2.56180100 -0.75809800

H 0.21789900 -2.96112300 -0.83777700

C -2.10753800 0.70889700 0.22094200

C -3.27150300 1.43650100 0.48108200

H -4.24730600 0.98079300 0.36692100

C -3.16663800 2.76257300 0.89713500

H -4.06426000 3.33663100 1.10517200

C -0.78737000 2.56178100 0.75812800

C -1.90598800 3.33928700 1.04074500

H -1.78352600 4.36841200 1.35988800

H 0.21787900 2.96110700 0.83782200

Br 2.11408100 1.47822200 -1.31819600

Br 2.11412700 -1.47819600 1.31817800

**PhCOONa**

-582.572164 a.u. (zero imaginary freq)

Na -3.67938800 -0.00173500 0.01316300

O -1.73761600 -1.12108500 -0.02712200

C -1.14391500 0.00126100 -0.00768500

O -1.73780500 1.12413400 0.00877900

C 0.36735200 0.00013800 -0.00340900

C 1.07632500 -1.20845500 0.00571800

C 1.07708900 1.20799000 -0.00780000

C 2.47108800 -1.20978100 0.01061900

H 0.52024600 -2.14032800 0.01122600

C 2.47175900 1.20897000 -0.00448000

H 0.52128700 2.13949000 -0.01416400

C 3.17199100 -0.00056200 0.00488600

H 3.01255000 -2.15209700 0.01884500

H 3.01361000 2.15108900 -0.00902000

H 4.25881100 -0.00082500 0.00798100

**B**

-1506.734475 a.u. (zero imaginary freq)

C -2.76051300 3.42495800 -2.10904400

C -1.37223200 3.30870400 -2.12370800

N -1.49757000 1.43345800 -0.65223000

C -2.85309600 1.52682000 -0.64351900

N -2.62884300 -0.36437400 0.75701800

H -3.25634000 4.20678000 -2.67496900

H -0.75246700 3.98746100 -2.69848100

O 1.59518400 -2.52187100 2.09218700

C 0.79853600 -2.11185200 1.24839300

O -0.22388800 -1.34662600 1.54885200

Ni -0.78644000 0.02910200 0.42748900

C -3.51307100 2.52047900 -1.36181100

H -4.59425900 2.58606300 -1.34057100

C -0.77388200 2.29674100 -1.37948600

H 0.29779900 2.15042200 -1.35547900

C -3.50513200 0.48768100 0.16517700

C -4.87969000 0.33798800 0.32967900

H -5.56743800 1.02445700 -0.14901600

C -5.35363800 -0.70967800 1.11748200

H -6.42131200 -0.84341600 1.25786300

C -3.08309700 -1.37325400 1.51396400

C -4.44431400 -1.57833600 1.71778100

H -4.77451200 -2.40558700 2.33576500

H -2.32115600 -2.00728200 1.94939800

C 1.59534500 1.23971700 0.84458500

O 1.05526700 1.86810500 1.76225000

O 0.97801500 0.44981300 0.01015800

C 0.94988500 -2.53882900 -0.19073500

C 2.22591300 -2.86737200 -0.66541500

C -0.14695200 -2.63155900 -1.05786100

C 2.41185300 -3.24100400 -1.99600800

H 3.06689100 -2.80979300 0.01770300

C 0.03456500 -3.02405600 -2.38551500

H -1.14523300 -2.40978000 -0.69361300

C 1.31533200 -3.31842500 -2.86050700

H 3.40924800 -3.47366100 -2.35914200

H -0.82363200 -3.10265100 -3.04755000

H 1.45768500 -3.61366400 -3.89647800

C 3.07555600 1.35193800 0.59566600

C 3.82795900 2.26419700 1.34655600

C 3.71595900 0.55310100 -0.36191300

C 5.20200000 2.38154400 1.14150500

H 3.32033200 2.87228000 2.08811400

C 5.09154200 0.66729500 -0.56379900

H 3.13119100 -0.15709800 -0.93515600

C 5.83654400 1.58237200 0.18566400

H 5.77886100 3.09309400 1.72598700

H 5.58289500 0.04213600 -1.30448400

H 6.90780200 1.67141000 0.02655200

**NaBr**

-175.812232 a.u. (zero imaginary freq)

Na 0.00000000 0.00000000 -2.02542500

Br 0.00000000 0.00000000 0.63656200

**B_2_Pin_2_**

-822.329786 a.u. (zero imaginary freq)

B -0.85062500 -0.00014200 0.00039300

O -1.60925200 -1.10419100 -0.30293400

O -1.60920900 1.10400400 0.30351600

C -3.00077200 -0.78431900 0.01608800

C -3.00063500 0.78435800 -0.01614700

C -3.27224300 1.36229700 -1.40714000

H -4.31671600 1.21837400 -1.70106600

H -3.06502700 2.43722900 -1.39527900

H -2.63100700 0.90078600 -2.16507600

C -3.90308300 1.44921500 1.01427200

H -3.82584100 2.53810200 0.92568300

H -4.94883500 1.17049000 0.84510600

H -3.62874000 1.17112500 2.03446000

C -3.90284400 -1.44901700 -1.01476200

H -3.82575100 -2.53791700 -0.92620900

H -4.94863700 -1.17019500 -0.84601100

H -3.62806500 -1.17088900 -2.03482300

C -3.27314400 -1.36222300 1.40694400

H -4.31773800 -1.21812400 1.70035800

H -3.06610300 -2.43718900 1.39518200

H -2.63220700 -0.90082100 2.16520000

B 0.85143600 -0.00017700 0.00049200

O 1.61002200 1.03498600 0.49015200

O 1.60998100 -1.03518400 -0.48955800

C 3.00147000 -0.77503900 -0.11973400

C 3.00141100 0.77503700 0.11973700

C 3.26880500 -1.58696100 1.14955000

H 3.05687000 -2.64207900 0.94852600

H 4.31268200 -1.50147500 1.46743500

H 2.62548400 -1.26318000 1.97418200

C 3.90618900 -1.24987700 -1.24843400

H 4.95122100 -1.00398500 -1.03052600

H 3.83015200 -2.33752700 -1.35180400

H 3.63397000 -0.79757900 -2.20479400

C 3.90660900 1.25019600 1.24793800

H 4.95154400 1.00414100 1.02974600

H 3.83070700 2.33788900 1.35094800

H 3.63470400 0.79828000 2.20456200

C 3.26802600 1.58683300 -1.14976100

H 3.05599600 2.64193900 -0.94877600

H 4.31178400 1.50147300 -1.46808500

H 2.62442700 1.26280800 -1.97408100

**PhCO_2_BPin**

-831.397399 a.u. (zero imaginary freq)

O -1.10805200 1.83222600 -0.53942400

C -1.41363800 0.66525800 -0.40397000

O -0.50682100 -0.34491900 -0.50684200

B 0.85279500 -0.17198200 -0.27151000

O 1.75373200 -0.96293200 -0.92110600

O 1.37807800 0.68544300 0.64738800

C 3.07719600 -0.44548900 -0.55402000

C 2.78259400 0.29103900 0.80420800

C 2.84077100 -0.63609000 2.01818900

H 3.87074600 -0.92806400 2.24452000

H 2.43434400 -0.11094300 2.88826600

H 2.24849500 -1.54311700 1.85875100

C 3.61221000 1.54260800 1.04731500

H 3.33005700 1.99081200 2.00568700

H 4.67711300 1.29083200 1.09078700

H 3.46067900 2.28825500 0.26344900

C 4.04411600 -1.61490400 -0.44976000

H 4.18187700 -2.07036500 -1.43604500

H 5.02146700 -1.27008900 -0.09605000

H 3.67700600 -2.38416400 0.23350600

C 3.49838000 0.50379300 -1.67576300

H 4.50934000 0.88847200 -1.51052100

H 3.48993300 -0.04009500 -2.62562700

H 2.81279700 1.35318600 -1.76183500

C -2.79385700 0.18027800 -0.15597100

C -3.08024800 -1.17807600 0.04169900

C -3.82862500 1.12604300 -0.10781300

C -4.39149300 -1.58272500 0.28508800

H -2.28034300 -1.90873800 0.00666400

C -5.13671500 0.71751700 0.13416800

H -3.59303600 2.17370800 -0.26099000

C -5.41937800 -0.63754600 0.33108600

H -4.61159100 -2.63494700 0.43936400

H -5.93566300 1.45231500 0.16994700

H -6.44057600 -0.95591700 0.52081600

**TSBC**

-2329.05045 a.u. (one imaginary freq: -82.43)

O 0.68486600 -2.46789100 0.27313900

B 0.40427000 0.24611000 1.18744500

B -0.56121900 -2.04219900 0.80284600

O 0.12152900 0.77783900 2.43803800

O 1.77174300 0.11188700 1.02104300

O -1.70897600 -1.88498000 0.02141100

O -0.82878100 -2.41786100 2.09401900

C -2.27090300 -2.31587900 2.29228100

C -2.82866300 -2.40134700 0.82094200

C 2.43535700 0.34415900 2.29226900

C 1.36780500 1.18216600 3.07968500

C 1.26830800 0.85828900 4.56564400

H 2.20855500 1.09257700 5.07793800

H 0.47610700 1.46250300 5.02161400

H 1.03199700 -0.19486000 4.73617600

C 2.69932500 -1.03542900 2.90367200

H 3.31113200 -1.61820600 2.20774900

H 3.23809500 -0.95953800 3.85423600

H 1.76448100 -1.57913500 3.06884900

C 3.75349900 1.06282800 2.03170800

H 4.26469900 1.28665200 2.97527000

H 4.40951000 0.41996300 1.43518800

H 3.60472200 1.99505600 1.48430200

C 1.50354600 2.69321900 2.87888100

H 0.65131300 3.19135200 3.35542900

H 2.41910400 3.07731300 3.34160500

H 1.49259200 2.95970400 1.81915400

C -4.06540100 -1.55892800 0.55490900

H -4.35050200 -1.64086500 -0.49877100

H -4.90119600 -1.92231800 1.16256800

H -3.89819100 -0.50733900 0.78665800

C -3.04786300 -3.83486400 0.33559300

H -3.89839600 -4.30065500 0.84277800

H -3.25595000 -3.81665000 -0.73910400

H -2.16104600 -4.45483100 0.50176100

C -2.56374000 -0.98220700 2.97677400

H -3.62895600 -0.89403100 3.21354800

H -1.99536200 -0.92984300 3.91002700

H -2.26334100 -0.13780600 2.35845000

C -2.70134200 -3.46819700 3.19171100

H -2.26425200 -3.33744000 4.18732700

H -3.79126900 -3.48259800 3.29896100

H -2.37497100 -4.43436600 2.79975600

Ni -0.86198400 0.23916300 -0.52904300

C -2.13089400 -0.77165700 -3.15587500

C -3.35543000 0.83700100 -2.01189200

C -4.39431700 0.75217200 -2.94383700

C -4.27141200 -0.13174200 -4.01341000

C -3.11954600 -0.90877200 -4.12751300

H -1.21561400 -1.35241900 -3.18320800

H -5.28929800 1.35231500 -2.83294200

H -5.06954000 -0.21383000 -4.74472300

H -2.98689600 -1.60941700 -4.94475200

C -3.39429900 1.72636200 -0.82594300

C -4.37719600 2.70025500 -0.63328300

C -4.33853000 3.48172800 0.51988600

H -5.15445700 2.85505000 -1.37165200

C -2.36420100 2.29703600 1.17923300

C -3.31793300 3.27725100 1.44521300

H -5.09533600 4.24207200 0.68631100

H -1.54023600 2.09620700 1.85343400

H -3.25007300 3.86627200 2.35354300

N -2.24835700 0.07686600 -2.13036400

N -2.40966800 1.53643600 0.07968000

C 1.16636200 -1.98922700 -0.87080500

O 0.53988800 -1.19709600 -1.57775900

O 0.34001700 3.57336300 -0.26422700

C 0.82110000 2.65203400 -0.94693600

O 0.16467200 1.70153500 -1.51782800

C 2.51871100 -2.47234600 -1.21749600

C 3.07326100 -2.09799400 -2.45034500

C 3.26051200 -3.26262000 -0.32712600

C 4.36260600 -2.50337300 -2.78565400

H 2.48878700 -1.49040200 -3.13218100

C 4.55225100 -3.66128800 -0.66486200

H 2.82761700 -3.54951600 0.62383100

C 5.10474000 -3.28164000 -1.89146300

H 4.78957600 -2.21299100 -3.74096000

H 5.12876200 -4.26627400 0.02859400

H 6.11244600 -3.59296800 -2.15149300

C 2.32306200 2.61477900 -1.15826700

C 2.93877600 1.46561600 -1.66903700

C 3.11509300 3.71000500 -0.79335200

C 4.32573200 1.40824800 -1.80568100

H 2.31227000 0.61970000 -1.92269000

C 4.50281700 3.66107300 -0.93856700

H 2.62665700 4.59040600 -0.38778800

C 5.11138300 2.50698100 -1.44221100

H 4.79533100 0.50451300 -2.18601800

H 5.11009300 4.51724700 -0.65565300

H 6.19235900 2.46365500 -1.54834800

**C**

-1497.667697 a.u. (zero imaginary freq)

C -1.35719900 -4.28882800 2.21499700

C -2.05893400 -3.09459400 2.37241600

N -0.63893600 -2.04033200 0.76181300

C 0.05299100 -3.19025500 0.60513700

N 1.37343300 -1.92948200 -0.94999500

H -1.63448200 -5.17300400 2.78041200

H -2.89358800 -3.01381600 3.06020300

O 1.27713900 0.53154500 0.92839500

C 1.83269000 1.22740800 0.01416300

O 1.48733200 1.10957500 -1.19988100

Ni -0.01089500 -0.39964500 -0.42210500

C -0.29044800 -4.34112600 1.32069400

H 0.26227100 -5.26305800 1.18862600

C -1.66794700 -1.99210200 1.61720400

H -2.18136900 -1.03795600 1.68148600

C 1.18129400 -3.12835900 -0.35579200

C 2.01038100 -4.21678300 -0.64094300

H 1.85266900 -5.17564700 -0.16255400

C 3.05296100 -4.05399500 -1.55060300

H 3.70533400 -4.88986800 -1.78301700

C 2.38173500 -1.77288000 -1.81740700

C 3.24826000 -2.81099100 -2.14983300

H 4.05202200 -2.64283800 -2.85817600

H 2.48823400 -0.77661300 -2.23230500

B -1.76437600 0.68366400 -0.46768200

O -2.55291200 0.99206300 -1.57359100

O -2.37543100 1.15390000 0.69600400

C -3.84830800 1.47458200 -1.10790400

C -3.51262000 1.98966700 0.32919900

C -3.02330400 3.44072300 0.35067300

H -3.83656600 4.14470700 0.14424100

H -2.61886300 3.66725700 1.34294600

H -2.22821800 3.60211900 -0.38446500

C -4.61856400 1.79502700 1.35913400

H -4.28779800 2.16891400 2.33459100

H -5.51845400 2.35159600 1.07334700

H -4.88422600 0.74111300 1.47408100

C -4.35530000 2.53904300 -2.07271200

H -4.54770400 2.08966300 -3.05337200

H -5.29428600 2.97482300 -1.71228200

H -3.62823200 3.34373100 -2.20704500

C -4.79454000 0.26967800 -1.10991300

H -5.81471100 0.55313000 -0.82963600

H -4.82431800 -0.15914500 -2.11721400

H -4.44695100 -0.50741600 -0.42130400

C 2.90617900 2.20048600 0.39328600

C 3.56879900 2.93552400 -0.59891500

C 3.25729100 2.38267300 1.73783500

C 4.56997700 3.84159400 -0.25134800

H 3.28924800 2.78674200 -1.63655300

C 4.25714800 3.29032700 2.08547800

H 2.73914700 1.80983600 2.49953300

C 4.91539100 4.02070900 1.09154400

H 5.08079000 4.40809200 -1.02509700

H 4.52373400 3.42910000 3.12963500

H 5.69516400 4.72715300 1.36277200

**P**

-1488.602967 a.u. (zero imaginary freq)

C 4.61800100 2.77824100 -0.15552200

C 3.33595800 3.38246300 -0.25624300

N 2.29468300 1.21511700 -0.14147200

C 3.54302400 0.60502600 -0.06052500

N 2.27910000 -1.42490000 0.00162200

H 5.51587400 3.38955100 -0.15615900

H 3.21915100 4.45726900 -0.34228000

Ni 0.78252700 -0.09972800 -0.09141800

C 4.71391700 1.40861600 -0.06267600

H 5.68789300 0.93635300 0.00641300

C 2.22748500 2.56232500 -0.24497900

H 1.22599100 2.96947000 -0.33422500

C 3.53484900 -0.82260700 0.01560200

C 4.69706200 -1.63350200 0.10507300

H 5.67614200 -1.16684400 0.11164500

C 4.58665200 -3.00256800 0.18787300

H 5.47820000 -3.61920100 0.25640500

C 2.19813700 -2.77199400 0.09303200

C 3.29711500 -3.59935300 0.18669900

H 3.16729300 -4.67364000 0.25886100

H 1.19263800 -3.17616400 0.09596200

B -0.82135500 1.03901700 0.02490100

O -1.60483200 1.30039000 1.12417100

O -0.97701300 1.96875800 -0.97743500

C -2.16979600 2.64213000 0.95669000

C -2.07120400 2.86760300 -0.59719800

C -3.30616200 2.40073800 -1.36635400

H -4.16688800 3.04789000 -1.16978700

H -3.09086900 2.43373800 -2.43952200

H -3.57273600 1.37383000 -1.10585400

C -1.69402800 4.28328500 -1.01275000

H -1.61867600 4.33805100 -2.10386500

H -2.46256000 4.99427300 -0.69068300

H -0.73559900 4.59161700 -0.58829500

C -3.58629500 2.63576900 1.51419800

H -3.55653400 2.43901800 2.59116100

H -4.06520500 3.60909900 1.36122100

H -4.20252700 1.86581800 1.04414000

C -1.28143600 3.58564100 1.76923300

H -1.65403200 4.61403300 1.73432000

H -1.27754200 3.25845100 2.81404500

H -0.24878900 3.57458600 1.40665900

B -0.93148800 -1.09500900 -0.13022000

O -1.77382400 -1.33051100 -1.19297200

O -1.19880900 -1.90517000 0.95093200

C -2.17288600 -2.91278500 0.52250600

C -2.83776200 -2.22559000 -0.72134200

C -4.02540900 -1.33576900 -0.35248800

H -3.75666500 -0.62352500 0.43305700

H -4.34117800 -0.77332400 -1.23628100

H -4.87585500 -1.93292500 -0.00902900

C -3.21501200 -3.16930300 -1.85426200

H -3.96636200 -3.89112800 -1.51576400

H -3.64497500 -2.59612200 -2.68244800

H -2.34986600 -3.71884400 -2.23231500

C -3.11674000 -3.19127600 1.68450400

H -3.90792800 -3.88428900 1.37841500

H -2.56073000 -3.65454700 2.50666000

H -3.57985300 -2.27573500 2.05920700

C -1.38950500 -4.17717900 0.16732200

H -0.78616200 -4.47963500 1.02919900

H -2.06464700 -5.00220400 -0.08166300

H -0.72000500 -4.01125900 -0.68188900

**TSCI**

-1497.652196 a.u. (one imaginary freq: -170.69)

C 2.99058000 -0.96128900 3.55130100

C 1.60160800 -1.07025400 3.50968100

N 1.63816100 -0.74319200 1.14133400

C 2.98656300 -0.63372500 1.16840000

N 2.79187300 -0.33336900 -1.20245100

H 3.52532600 -1.04683100 4.49208600

H 1.02024600 -1.24143600 4.40924300

O 0.33482400 1.46181200 -0.56456600

C -0.88387500 1.66179300 -0.26635600

O -1.75640000 0.74601300 -0.14484500

Ni 0.74287400 -0.52294100 -0.71729900

C 3.69392500 -0.74483800 2.36826700

H 4.77380500 -0.66418300 2.38515900

C 0.96131800 -0.96016900 2.27758200

H -0.11345200 -1.06338600 2.16554100

C 3.63259400 -0.39257100 -0.14419700

C 5.01177900 -0.23383400 -0.30768100

H 5.68053800 -0.28165300 0.54309100

C 5.52140100 -0.00552800 -1.58377700

H 6.58983900 0.12140700 -1.72699200

C 3.28596500 -0.11269700 -2.42744800

C 4.64637700 0.05920300 -2.66735100

H 5.00303300 0.23678300 -3.67595300

H 2.56008600 -0.07513400 -3.23432400

B -1.25227900 -0.95284500 -0.47846400

O -2.14891300 -1.33605100 -1.52827600

O -1.62143000 -1.67809800 0.69977100

C -3.00077000 -2.39335000 -1.05159600

C -2.94520500 -2.21617600 0.50666400

C -3.97030500 -1.20093700 1.03030900

H -4.99182000 -1.59664600 0.99113600

H -3.73549800 -0.96963300 2.07569600

H -3.92714500 -0.26897600 0.46288200

C -3.06131400 -3.51297700 1.30324600

H -3.01024700 -3.29550900 2.37673300

H -4.01750800 -4.01307400 1.10855300

H -2.25074400 -4.20578700 1.06257200

C -4.39108000 -2.22645100 -1.66011100

H -4.33988400 -2.35711700 -2.74749400

H -5.08844900 -2.97553900 -1.26577000

H -4.79965800 -1.23248000 -1.46046400

C -2.39305500 -3.72471000 -1.51636200

H -3.02734200 -4.58206200 -1.26410100

H -2.27402000 -3.69867500 -2.60532000

H -1.40433800 -3.87933200 -1.07224000

C -1.33075400 3.07330300 -0.04036400

C -2.66105100 3.34704300 0.30635700

C -0.41821000 4.12961900 -0.16661500

C -3.07328500 4.66128100 0.52168000

H -3.36078200 2.52431400 0.40485200

C -0.83167600 5.44352100 0.04830700

H 0.60988900 3.90964300 -0.43265800

C -2.15970400 5.71156300 0.39277700

H -4.10564600 4.86761400 0.78999300

H -0.11976400 6.25813900 -0.05162500

H -2.48140100 6.73570700 0.56094100

**I**

-666.282559 a.u. (zero imaginary freq)

C -2.89590700 1.80192500 -0.00305300

C -3.50727800 0.51933900 0.00151400

N -1.33645100 -0.52120000 0.00294600

C -0.71590500 0.72902900 0.00022800

N 1.33651000 -0.52147000 -0.00016300

H -3.50585700 2.70083100 -0.00639400

H -4.58536600 0.40227600 0.00259000

Ni -0.00015500 -1.92050500 -0.00118500

C -1.52307600 1.89901300 -0.00383400

H -1.05051300 2.87528900 -0.00872500

C -2.68555900 -0.59018100 0.00378300

H -3.10048500 -1.59488700 0.00647700

C 0.71606500 0.72896500 0.00071700

C 1.52320000 1.89888200 0.00276000

H 1.05046800 2.87509100 0.00616200

C 2.89605800 1.80186800 0.00172700

H 3.50601300 2.70075300 0.00334100

C 2.68566000 -0.59030900 -0.00093800

C 3.50734400 0.51921000 -0.00073600

H 4.58542500 0.40200400 -0.00148300

H 3.10063000 -1.59498900 -0.00127400

**J**

-1077.456326 a.u. (zero imaginary freq)

C 2.72625500 3.52232900 0.08625000

C 1.33212100 3.52729000 0.07856000

N 1.31468900 1.13509900 0.02222300

C 2.66760100 1.12033800 0.02591900

N 2.37175700 -1.25362000 -0.01106000

H 3.28292100 4.45387000 0.11269300

H 0.76989200 4.45469600 0.09773200

Ni 0.42134800 -0.72095200 -0.00056000

C 3.40607800 2.30541300 0.06017300

H 4.48946100 2.28787100 0.06861900

C 0.66289600 2.30555100 0.04695700

H -0.42074100 2.23344400 0.04360200

C 3.26753100 -0.23365100 -0.00664900

C 4.64234700 -0.47488700 -0.03316200

H 5.34815800 0.34693100 -0.03664300

C 5.10225600 -1.79013100 -0.05741500

H 6.16818500 -1.99378700 -0.07687100

C 2.82236800 -2.51809100 -0.03478200

C 4.17775600 -2.83337900 -0.05667000

H 4.49254100 -3.87100200 -0.07413100

H 2.06250700 -3.29313800 -0.03606800

B -1.58494700 -0.54986100 0.00183600

O -2.57933700 -1.52166900 -0.14228500

O -2.19183200 0.70893600 0.14249000

C -3.87952400 -0.92373000 0.12487900

C -3.61195900 0.59215700 -0.14984500

C -3.80022500 0.98051600 -1.62025300

H -4.85738600 0.98173800 -1.90707500

H -3.40222800 1.98884300 -1.77734800

H -3.26020700 0.29631500 -2.28261500

C -4.38159400 1.55739600 0.74408500

H -4.11746000 2.58990400 0.48854200

H -5.46237900 1.44209900 0.60204600

H -4.15169100 1.40198000 1.80108200

C -4.91682000 -1.56919500 -0.78708300

H -5.02674000 -2.62943700 -0.53294900

H -5.89542300 -1.09105200 -0.66345100

H -4.62845100 -1.50255200 -1.83913900

C -4.21741300 -1.21956800 1.59002600

H -5.21411200 -0.85275400 1.85782000

H -4.19811300 -2.30287500 1.75034500

H -3.48552500 -0.76377800 2.26457900

**TSPQ**

-1717.809247 a.u. (one imaginary freq: -964.79)

C -2.99546100 -1.50640800 -0.12947500

C -1.33968200 -2.62159000 1.07326500

C -2.24509600 -3.57845700 1.52329400

C -3.57255700 -3.48569500 1.11051200

C -3.95528100 -2.43833500 0.27292200

C -3.29507200 -0.34210200 -0.99284300

C -2.38715200 1.47125500 -2.14233600

C -3.64327200 1.89017100 -2.57077900

C -4.75853400 1.14957000 -2.18166500

C -4.58549200 0.02143700 -1.38248100

H -0.28958200 -2.64434300 1.34215600

H -1.90988000 -4.37404800 2.17944700

H -4.30626900 -4.21744100 1.43323500

H -4.98211400 -2.35457500 -0.06179600

H -1.48438200 2.01827000 -2.39003500

H -3.73798200 2.77634100 -3.18842400

H -5.75548900 1.44723500 -2.49075900

H -5.44334000 -0.55618100 -1.06074400

N -2.21992600 0.38384700 -1.37721800

N -1.70606200 -1.61765200 0.26673200

Ni -0.42425300 -0.25635100 -0.58461400

B 0.37883700 1.49518700 0.34632800

O 1.37141200 1.78076900 1.26615400

O 0.35942500 2.40523000 -0.69850600

C 2.22754600 2.82231200 0.68082600

C 2.70983600 3.74122700 1.79509800

H 3.30644100 4.56050700 1.37907200

H 1.87941400 4.16960600 2.36002700

H 3.34473700 3.17872900 2.48815700

C 1.27146600 3.49879900 -0.36089700

C 1.94889800 3.97686200 -1.63695000

H 1.20326200 4.41986800 -2.30618500

H 2.69780500 4.74365600 -1.41021600

H 2.43898500 3.15603300 -2.16659000

C 0.40610900 4.60775100 0.24217700

H 1.00175600 5.50110200 0.45828400

H -0.36949900 4.88287500 -0.48075000

H -0.07824500 4.27381100 1.16570500

C 3.41135800 2.10394000 0.03646800

H 4.12050100 2.81555100 -0.39821000

H 3.93484000 1.52720300 0.80457200

H 3.08187100 1.41215500 -0.74324000

O -1.93425500 1.38007100 1.68159700

H -0.82784900 0.99591100 0.83868900

H -2.50571600 1.83406300 1.03921600

B 1.39331400 -1.14743500 -0.45881400

O 2.32036900 -1.28988200 -1.47657500

O 1.73647100 -1.93246000 0.63522900

C 3.40851200 -2.15574900 -1.01548400

C 2.76021200 -2.88386300 0.21232400

C 2.02643400 -4.17481700 -0.16436000

H 2.72651300 -4.96408800 -0.45684900

H 1.45683000 -4.52870900 0.70129100

H 1.32423900 -4.00850900 -0.98782900

C 3.70201300 -3.14273300 1.38094600

H 3.15452600 -3.63428000 2.19264400

H 4.52166200 -3.80355300 1.07727900

H 4.12804800 -2.21564600 1.77166900

C 3.81022000 -3.07177400 -2.16524200

H 4.23876200 -2.47584200 -2.97857300

H 4.56894700 -3.79149600 -1.83789500

H 2.95474400 -3.62371600 -2.56231100

C 4.58296200 -1.25494300 -0.63364800

H 5.46470100 -1.84798900 -0.36845500

H 4.84226000 -0.62026100 -1.48744500

H 4.33391200 -0.60762000 0.20975900

O -0.46929900 3.00979600 3.24864700

H 0.35831000 2.64161800 2.89827300

H -1.12785700 2.48627100 2.71436600

O -3.16723200 -0.53243400 3.12581100

H -3.70261900 -1.00578900 2.47282100

H -2.72493000 0.17913200 2.58577800

**Q**

-1086.545362 a.u. (zero imaginary freq)

C 4.05432500 -2.90945600 0.02294400

C 2.78392500 -3.48478200 -0.00129800

N 1.78201000 -1.30704900 -0.01559900

C 3.01454600 -0.74018600 0.00214400

N 1.78199400 1.30705300 0.01557100

H 4.94409500 -3.53078200 0.04035900

H 2.64881600 -4.56085500 -0.00507500

O -1.53488600 -1.10485000 -0.00948700

C -2.16768300 -0.00010700 0.00010000

O -1.53480000 1.10460700 0.00971100

Ni 0.27024100 -0.00002500 0.00001400

C 4.17184100 -1.52099500 0.02561600

H 5.15098600 -1.05793100 0.04968600

C 1.67461100 -2.64473300 -0.01794200

H 0.66315000 -3.03732800 -0.03308700

C 3.01452800 0.74025000 -0.00202000

C 4.17179200 1.52112500 -0.02542900

H 5.15097300 1.05812600 -0.04927800

C 4.05417600 2.90957000 -0.02299100

H 4.94390200 3.53096000 -0.04039400

C 1.67447400 2.64473600 0.01771900

C 2.78373500 3.48483900 0.00100900

H 2.64859800 4.56090900 0.00455600

H 0.66297700 3.03723300 0.03280800

C -3.66296000 -0.00003900 0.00004000

C -4.36961200 1.21072400 0.00818500

C -4.36970800 -1.21074800 -0.00815900

C -5.76383000 1.21054000 0.00814000

H -3.81529800 2.14305200 0.01458700

C -5.76392300 -1.21045500 -0.00822200

H -3.81546600 -2.14311900 -0.01451700

C -6.46329800 0.00007100 -0.00006800

H -6.30576100 2.15221400 0.01459100

H -6.30593000 -2.15208600 -0.01471300

H -7.54994500 0.00011200 -0.00010900

**H_2_O**

-76.417974 a.u. (zero imaginary freq)

C 4.05432500 -2.90945600 0.02294400

C 2.78392500 -3.48478200 -0.00129800

N 1.78201000 -1.30704900 -0.01559900

C 3.01454600 -0.74018600 0.00214400

N 1.78199400 1.30705300 0.01557100

H 4.94409500 -3.53078200 0.04035900

H 2.64881600 -4.56085500 -0.00507500

O -1.53488600 -1.10485000 -0.00948700

C -2.16768300 -0.00010700 0.00010000

O -1.53480000 1.10460700 0.00971100

Ni 0.27024100 -0.00002500 0.00001400

C 4.17184100 -1.52099500 0.02561600

H 5.15098600 -1.05793100 0.04968600

C 1.67461100 -2.64473300 -0.01794200

H 0.66315000 -3.03732800 -0.03308700

C 3.01452800 0.74025000 -0.00202000

C 4.17179200 1.52112500 -0.02542900

H 5.15097300 1.05812600 -0.04927800

C 4.05417600 2.90957000 -0.02299100

H 4.94390200 3.53096000 -0.04039400

C 1.67447400 2.64473600 0.01771900

C 2.78373500 3.48483900 0.00100900

H 2.64859800 4.56090900 0.00455600

H 0.66297700 3.03723300 0.03280800

C -3.66296000 -0.00003900 0.00004000

C -4.36961200 1.21072400 0.00818500

C -4.36970800 -1.21074800 -0.00815900

C -5.76383000 1.21054000 0.00814000

H -3.81529800 2.14305200 0.01458700

C -5.76392300 -1.21045500 -0.00822200

H -3.81546600 -2.14311900 -0.01451700

C -6.46329800 0.00007100 -0.00006800

H -6.30576100 2.15221400 0.01459100

H -6.30593000 -2.15208600 -0.01471300

H -7.54994500 0.00011200 -0.00010900

**TSCD**

-1726.899982 a.u. (one imaginary freq: -724.30)

C -3.34639300 -1.24969900 -0.36517900

C -2.32455100 -2.65703300 1.19247900

C -3.54388100 -3.24023800 1.52784100

C -4.69577400 -2.79952100 0.88071400

C -4.59925700 -1.78964800 -0.07496200

C -3.13059800 -0.16571700 -1.35284300

C -1.52999000 1.18344600 -2.38556800

C -2.49961400 1.84968400 -3.12747100

C -3.83276500 1.47667300 -2.96065400

C -4.15517500 0.45712300 -2.06734200

H -1.39916000 -2.92523900 1.69578000

H -3.58071500 -4.01706700 2.28373100

H -5.66349700 -3.23068800 1.11720700

H -5.48723700 -1.43179200 -0.58078300

H -0.47739700 1.42814100 -2.46499600

H -2.21206800 2.63809900 -3.81384900

H -4.61884200 1.97394200 -3.51997600

H -5.18786300 0.16220800 -1.92859000

N -1.84357700 0.20681600 -1.52622300

N -2.23359200 -1.69509500 0.26403000

Ni -0.50271100 -0.78557400 -0.36069900

B 0.20368300 1.00944600 0.83412900

O 1.46368500 1.15401400 1.35014000

O -0.32200100 2.18911700 0.35396300

C 1.92486400 2.50581400 1.01972800

C 2.75338200 3.03097400 2.18355800

H 3.05257700 4.06896400 2.00125800

H 2.20332400 2.98668600 3.12652000

H 3.66252500 2.42946100 2.28877400

C 0.57402500 3.27124000 0.77899800

C 0.61559500 4.32025500 -0.32147500

H -0.37459100 4.77435400 -0.43529600

H 1.32605500 5.11393100 -0.06621600

H 0.90610500 3.88739000 -1.28159100

C -0.03577000 3.85279500 2.05524900

H 0.53909100 4.71169900 2.41504800

H -1.05689300 4.18557100 1.84356700

H -0.08370600 3.10501600 2.85333200

C 2.78860500 2.38044200 -0.23568700

H 3.21019000 3.34879600 -0.52346000

H 3.61506500 1.69344800 -0.02965000

H 2.21698900 1.97326500 -1.07303700

O -2.97368500 1.55044700 1.50270700

H -0.57568100 0.05853600 1.42298200

H -2.27658000 1.95967700 0.96309700

C 5.49162500 -1.03533000 -1.79418700

C 6.10433900 -2.00749700 -0.99765800

C 5.34003400 -2.77299800 -0.11194700

C 3.96463100 -2.56763300 -0.02141200

C 3.34685800 -1.59379900 -0.81841600

C 4.11645000 -0.82784300 -1.70588800

H 6.08604900 -0.44050000 -2.48167600

H 7.17659500 -2.16831600 -1.06687400

H 5.81666800 -3.52768000 0.50698500

H 3.36104300 -3.15384600 0.66312600

H 3.62850200 -0.07583000 -2.31581400

C 1.88364200 -1.36223700 -0.71323500

O 1.31772400 -0.47246700 -1.42934900

O 1.17942800 -2.04821100 0.10532800

O -1.19260800 -0.14655100 2.72006900

H -2.44450000 0.89523800 2.02710400

H -0.69107200 0.47595800 3.27673700

O 0.40266000 -2.37945500 2.83612200

H -0.17940800 -1.57434500 2.86934900

H 0.84278600 -2.28893000 1.97179400

**D**

-4002.438833 a.u. (zero imaginary freq)

C 4.68070400 -1.45282400 1.16039900

C 4.07276700 -2.52802800 0.51531100

N 2.31732400 -1.08938300 -0.23789600

C 2.89591100 -0.03917900 0.38641900

N 1.01629900 1.20010000 -0.43758500

H 5.60710100 -1.58912700 1.70925800

H 4.50181400 -3.52342100 0.54239800

O -0.91607200 -1.10510800 0.23069400

C -1.81890400 -0.50512200 -0.44369800

O -1.53595000 0.02258900 -1.56481300

Ni 0.51647800 -0.68014300 -1.21721100

C 4.08899400 -0.19287300 1.09625000

H 4.55091900 0.64945200 1.59607200

C 2.88788700 -2.29785100 -0.17856600

H 2.37229500 -3.09266600 -0.70755100

C 2.17246800 1.24915300 0.26244800

C 2.62333200 2.44440000 0.82654600

H 3.55096900 2.47923600 1.38403600

C 1.86050400 3.59929200 0.66554300

H 2.19734700 4.53594800 1.09822700

C 0.28076700 2.30945100 -0.58651300

C 0.66654800 3.53502300 -0.04986900

H 0.04299700 4.41045000 -0.19321100

H -0.64278100 2.18770700 -1.14097400

C -3.20901400 -0.42360800 0.09532300

C -4.19666600 0.27473900 -0.61261100

C -3.53276400 -1.03951100 1.31210400

C -5.49424900 0.35514200 -0.10941100

H -3.93515300 0.74926500 -1.55240600

C -4.83124900 -0.96007300 1.81329500

H -2.76144600 -1.57695700 1.85338000

C -5.81334800 -0.26273400 1.10348800

H -6.25678300 0.89792600 -0.66095200

H -5.07862200 -1.44114200 2.75543900

H -6.82522000 -0.20109800 1.49444400

H 0.49385900 -1.94883200 -2.23369200

**HOBPin**

-487.044734 a.u. (zero imaginary freq)

B -1.59154700 0.05965200 -0.01197100

O -0.81033000 1.10714700 -0.43332100

O -0.88270900 -1.03812100 0.41972200

C 0.55830400 0.77516700 -0.04877200

C 0.51015400 -0.79501000 0.05008400

C 0.73877700 -1.48890900 -1.29359300

H 1.78356600 -1.41284800 -1.61023200

H 0.48744600 -2.54993100 -1.19495500

H 0.10610500 -1.06034000 -2.07782900

C 1.41211500 -1.39651700 1.11833400

H 1.30689400 -2.48671400 1.12099900

H 2.46112800 -1.15921200 0.91125100

H 1.16099100 -1.02732100 2.11561400

C 1.50912900 1.31797000 -1.10639700

H 1.47136800 2.41265400 -1.11180700

H 2.53908100 1.01743900 -0.88595700

H 1.24780700 0.96316000 -2.10622700

C 0.81813900 1.45123400 1.29854800

H 1.85162800 1.30450500 1.62690800

H 0.63947200 2.52671300 1.19916700

H 0.14858600 1.06591100 2.07464100

O -2.95447900 0.13500100 -0.03127700

H -3.34588700 -0.70810500 0.24806600

**CF_3_CO_2_Na**

-688.592541 a.u. (zero imaginary freq)

Na -2.27429300 0.02447500 0.00022500

O -0.66902300 1.51166800 -0.00042700

C 0.50665900 1.03812400 -0.00004600

O 1.60738900 1.58561400 0.00032400

C 0.49580400 -0.52750800 -0.00006600

F -0.20565700 -1.00284500 1.08492100

F 1.68782200 -1.11772300 0.00040200

F -0.20488500 -1.00289600 -1.08543200

**G**

-1718.806402 a.u. (zero imaginary freq)

C 1.78176900 -2.28179200 1.30833100

C 0.34179100 4.20219600 1.67980800

C -0.71769600 3.42836400 2.14838700

N -0.01456200 1.61881400 0.75605400

C 1.01760600 2.36075700 0.28976100

N 1.54172600 0.42034100 -1.01884200

H 0.48684900 5.21608600 2.03851100

H -1.42218600 3.81150100 2.87784800

O 1.27969000 -4.14802700 -0.04576800

C 0.99390900 -2.97502700 0.15583200

O 0.16394600 -2.22996400 -0.47387300

Ni -0.11462500 -0.28649900 -0.01936700

C 1.21983200 3.66564600 0.73987000

H 2.04619600 4.25734000 0.36672600

C -0.86067800 2.13406500 1.65743800

H -1.66690000 1.48447800 1.97877800

C 1.88272000 1.69348700 -0.71271500

C 2.97568600 2.30628800 -1.32672300

H 3.24948300 3.32501000 -1.08217400

C 3.71397900 1.58906000 -2.26648500

H 4.56582100 2.05295000 -2.75309700

C 2.24910900 -0.27018900 -1.92178300

C 3.34800600 0.28029700 -2.57396600

H 3.89630000 -0.30763100 -3.30120800

H 1.91534200 -1.28334000 -2.11471200

F 1.13101900 -1.15414900 1.78601500

F 1.97097200 -3.07866700 2.36122400

F 2.98545700 -1.85473500 0.87673100

C -2.57000300 -0.28398900 -0.37386100

C -4.07998000 -0.28460900 -0.67206800

F -4.81612100 -0.47733300 0.43233100

F -4.37397300 -1.27018700 -1.54587200

F -4.45751900 0.88381100 -1.22595800

O -1.80732200 0.06084800 -1.31797900

O -2.17210800 -0.63717100 0.76511100

**TSGH**

-2541.090416 a.u. (one imaginary freq: -125.46)

O 1.01986600 1.98706200 1.43444900

B 0.90761600 0.29745900 -0.89558000

B -0.00621200 2.17277400 0.44122600

O 0.90310900 0.61151600 -2.24182100

O 2.19560400 0.11062400 -0.43334600

O -1.34883400 1.93152900 0.69774800

O 0.22744000 3.18696000 -0.44486900

C -1.05110400 3.86093400 -0.66433300

C -2.10665800 2.76169500 -0.26049400

C 3.13691200 0.54233800 -1.45810200

C 2.26415100 0.49371400 -2.76543000

C 2.49807200 1.64617800 -3.73482100

H 3.52981800 1.63888400 -4.10407700

H 1.83011900 1.54089300 -4.59688700

H 2.30036700 2.61448500 -3.26912500

C 3.58190600 1.95578400 -1.07317300

H 4.00697400 1.92910700 -0.06560100

H 4.34404000 2.33632500 -1.76146000

H 2.73593500 2.64850300 -1.06394200

C 4.32532800 -0.41037700 -1.43861600

H 5.03821300 -0.16217300 -2.23290100

H 4.84314100 -0.32412800 -0.47688400

H 4.00851800 -1.44775300 -1.55848900

C 2.34532900 -0.84228100 -3.50543100

H 1.60383900 -0.84699600 -4.31269000

H 3.33339600 -0.98467000 -3.95668200

H 2.12911000 -1.67858900 -2.83633600

C -2.52807400 1.88446000 -1.43391700

H -3.14981200 1.06521600 -1.06753400

H -3.12101300 2.46612700 -2.14650300

H -1.66575100 1.46395500 -1.95468200

C -3.33425300 3.28316800 0.47181900

H -3.89945100 3.96549100 -0.17190900

H -3.98560800 2.44200000 0.73222900

H -3.06787700 3.80833200 1.39177000

C -1.11574400 4.30411400 -2.11889300

H -2.08871200 4.75491700 -2.34074500

H -0.34234400 5.05690300 -2.30425200

H -0.94930600 3.46827500 -2.80164100

C -1.05961000 5.07608600 0.26469500

H -0.19319300 5.70453900 0.03569900

H -1.96523900 5.67484500 0.12625800

H -0.99625400 4.77833000 1.31635600

Ni -0.71990200 -0.45661900 0.24274700

C -2.72206600 -0.59966900 2.56448900

C -3.45481100 -1.49204500 0.54653500

C -4.70562100 -1.78348500 1.09778100

C -4.94798800 -1.46553800 2.43208400

C -3.94073900 -0.86316500 3.18365500

H -1.90373700 -0.13042000 3.09781200

H -5.48407000 -2.23918300 0.49864000

H -5.91471500 -1.68317400 2.87521300

H -4.08974100 -0.59824500 4.22461500

C -3.09854000 -1.76760600 -0.86593900

C -3.90914400 -2.50323000 -1.73394000

C -3.49589700 -2.68680200 -3.05198600

H -4.84246200 -2.93324600 -1.39185000

C -1.52571700 -1.42318500 -2.54488200

C -2.28767400 -2.13383100 -3.46923400

H -4.11341000 -3.25546300 -3.74026700

H -0.57930700 -0.96883700 -2.80717000

H -1.93213800 -2.25061900 -4.48726200

N -2.48696200 -0.90654700 1.28406600

N -1.92356200 -1.24500400 -1.28067500

C 1.09357800 0.90939300 2.16890600

O 0.29593000 -0.00974800 2.19647800

O 1.04317100 -2.99919500 -1.32944700

C 1.04152500 -2.76733700 -0.12005100

O 0.13183200 -2.28105000 0.62561600

C 2.31082500 -3.16011800 0.68600600

C 2.35736600 0.89259300 3.04399000

F 3.36217100 -3.45541900 -0.10897000

F 2.06704200 -4.26245300 1.44321500

F 2.71310400 -2.18574100 1.52936200

F 3.46326900 1.00666900 2.29090800

F 2.43335100 -0.24381300 3.74174700

F 2.33123500 1.92620900 3.90803400

**H**

-1603.702801 a.u. (zero imaginary freq)

C -1.79869700 3.82238800 -2.14683200

C -0.69519800 3.29354300 -2.81713100

N -0.18904100 1.92810900 -0.92014800

C -1.25516200 2.43591800 -0.25829000

N -0.51941800 1.02653200 1.53090000

H -2.43126200 4.56474100 -2.62304300

H -0.43915400 3.60645500 -3.82332000

O 2.53406600 -0.30678600 1.19544800

C 3.19569700 -0.09011400 0.15018700

O 2.73293600 0.41640300 -0.90598200

Ni 0.77070200 0.41087300 0.06394100

C -2.08385000 3.39042400 -0.85397600

H -2.93351400 3.79589500 -0.31849900

C 0.08400800 2.34469000 -2.16340700

H 0.95131900 1.89490500 -2.63546300

C -1.46568300 1.89621200 1.10375100

C -2.56496600 2.21369500 1.90625800

H -3.32920700 2.89516700 1.55267600

C -2.67173900 1.63530600 3.16868900

H -3.51981100 1.87072000 3.80370200

C -0.62480500 0.46902000 2.74470800

C -1.68311200 0.75102500 3.60166900

H -1.72970500 0.28257900 4.57846900

H 0.16248700 -0.22440200 3.01924000

B -0.46300600 -1.13837200 -0.42218500

O -0.79193300 -2.14681700 0.47453200

O -1.34701100 -1.12112300 -1.49039800

C -2.13119800 -2.62347500 0.14132300

C -2.23365300 -2.27655700 -1.38043400

C -1.66319500 -3.36746400 -2.29010900

H -2.31367800 -4.24814600 -2.32182000

H -1.57426900 -2.97299700 -3.30795400

H -0.66791100 -3.68142000 -1.95942300

C -3.62242800 -1.86741200 -1.85437100

H -3.59453900 -1.62991100 -2.92365100

H -4.33864800 -2.68415100 -1.70920900

H -3.98587500 -0.98592400 -1.32032700

C -2.22721500 -4.10627900 0.47357800

H -2.12661700 -4.25135200 1.55491400

H -3.20013200 -4.50901500 0.16928400

H -1.44205500 -4.68392100 -0.02035300

C -3.10801400 -1.82232400 1.00867700

H -4.14040600 -2.16031500 0.87020700

H -2.84341500 -1.95706600 2.06222000

H -3.05830800 -0.75301500 0.78046100

C 4.66820700 -0.54022600 0.11698800

F 5.17774500 -0.71747800 1.34766800

F 4.76660100 -1.71800500 -0.54208100

F 5.44574700 0.35242300 -0.52550700

**TSHR**

-1832.919152 a.u. (one imaginary freq: -680.25)

C -3.35068400 -0.45368100 -0.18845900

C -2.63103800 -2.16105300 1.23471900

C -3.93592300 -2.42820500 1.64072000

C -4.97259500 -1.67203900 1.09943300

C -4.67893000 -0.66964200 0.17641100

C -2.92439600 0.59046800 -1.14923200

C -1.09849700 1.52581600 -2.26563200

C -1.90574600 2.46521800 -2.89791100

C -3.27401500 2.45005200 -2.63015200

C -3.79254900 1.50285500 -1.74958000

H -1.77696700 -2.68509200 1.65605100

H -4.12441400 -3.20961500 2.36846300

H -6.00185600 -1.85265100 1.39293800

H -5.47442900 -0.06980700 -0.24778600

H -0.02761500 1.48902000 -2.42630700

H -1.46787400 3.18680400 -3.57827200

H -3.93600600 3.16950600 -3.10142500

H -4.85295400 1.48477800 -1.53170400

N -1.60026300 0.61956100 -1.41805900

N -2.35383200 -1.20536300 0.33684100

Ni -0.51268500 -0.74303200 -0.39815900

B 0.75243700 0.71508700 0.78287600

O 2.03407300 0.44762900 1.17439700

O 0.55607800 2.01979200 0.39895500

C 2.84650000 1.63040000 0.86367800

C 3.87184500 1.81159900 1.97359800

H 4.44663700 2.73040500 1.81456600

H 3.40072900 1.85986400 2.95812600

H 4.57072800 0.96851100 1.96873700

C 1.76426500 2.76788600 0.77855600

C 2.02535600 3.82592700 -0.28190000

H 1.20728900 4.55406100 -0.28477900

H 2.95605900 4.36093000 -0.06458500

H 2.09871900 3.38858300 -1.28035900

C 1.45234000 3.41893000 2.12629600

H 2.28017700 4.05055600 2.46277600

H 0.56085700 4.04552500 2.02207600

H 1.24996700 2.66876300 2.89739200

C 3.54625100 1.34471300 -0.46517100

H 4.22855200 2.15777400 -0.73236700

H 4.12837500 0.42388700 -0.36526000

H 2.82794200 1.20123300 -1.27568200

O -2.10493700 2.06901400 1.71053400

H -0.21629300 -0.01250000 1.36494000

H -1.35694500 2.31778700 1.14242200

C 1.60780800 -1.86080200 -0.98291600

O 1.24399900 -0.84271200 -1.62635900

O 0.88809300 -2.43287200 -0.12106700

O -0.76222500 -0.08658300 2.74199500

H -1.73282900 1.27596000 2.17657800

H -0.06785300 0.35523800 3.26332100

O 0.15850800 -2.65969400 2.68737200

H -0.17569700 -1.72628500 2.77852500

H 0.58963900 -2.65257600 1.81642500

C 3.01891800 -2.42315200 -1.21915900

F 3.63290800 -1.82823900 -2.25184500

F 3.77626300 -2.23216800 -0.11874500

F 2.97033500 -3.74614000 -1.46258900

**R**

-1832.986978 a.u. (zero imaginary freq)

C 3.06099300 -1.04182400 0.18900800

C 2.15012700 -2.41665400 -1.46743800

C 3.41530500 -2.78284000 -1.91707000

C 4.53185600 -2.26680100 -1.26191800

C 4.35555600 -1.38195100 -0.20016000

C 2.75146900 -0.06899300 1.26272900

C 1.03408100 0.92468100 2.49581100

C 1.93001900 1.74887100 3.16791200

C 3.28628500 1.64161700 2.86168600

C 3.70542300 0.72411000 1.90041500

H 1.24071100 -2.75024400 -1.95914500

H 3.51384700 -3.45795100 -2.75981400

H 5.53363000 -2.54025800 -1.57781300

H 5.21418400 -0.96393800 0.31040700

H -0.03276800 0.96273600 2.68391400

H 1.56802000 2.45594000 3.90556900

H 4.01516200 2.27179000 3.36129900

H 4.75382700 0.64607100 1.64121200

N 1.44166500 0.04379500 1.57452900

N 1.98475400 -1.58161900 -0.43115700

Ni 0.22562800 -1.09504600 0.44262700

B -0.41578500 1.07317700 -1.34458000

O -1.75937300 1.46117000 -1.62412600

O 0.19714100 2.12354600 -0.55296200

C -2.10682000 2.51752800 -0.71199200

C -3.17341400 3.39311200 -1.36112700

H -3.39288400 4.26807300 -0.73869400

H -2.86136900 3.73777700 -2.35047900

H -4.10112300 2.82122100 -1.47849700

C -0.72436300 3.22785400 -0.48645100

C -0.57165400 3.91967600 0.86395200

H 0.43233000 4.35196100 0.94764100

H -1.29933600 4.73246300 0.97104200

H -0.70599600 3.21966200 1.69147800

C -0.37048700 4.20347000 -1.61628400

H -0.97574100 5.11560000 -1.57515400

H 0.68361300 4.48755400 -1.52331600

H -0.51096800 3.73905600 -2.59780900

C -2.65943600 1.88851400 0.57359800

H -3.03980300 2.64786900 1.26562300

H -3.48278100 1.21467700 0.31481400

H -1.89682000 1.29781300 1.08593200

O 2.76993700 1.59558900 -1.45244000

H -0.43734300 -0.00350100 -0.58229900

H 2.06397200 2.02716300 -0.93151700

C -2.04509700 -1.98520600 0.76973800

O -1.56619600 -1.18824400 1.61579200

O -1.37078100 -2.49833600 -0.16502000

O 0.31935100 0.57729500 -2.50490600

H 2.20148900 1.04423600 -2.02304100

H 0.08066800 1.14156400 -3.26070300

O -0.67815100 -2.03612800 -2.92238300

H -0.32161900 -1.12571900 -2.82319700

H -1.09945600 -2.20405900 -2.06220100

C -3.55481700 -2.26985700 0.81518700

F -4.04865400 -2.15557700 2.05676200

F -4.18974500 -1.37055600 0.02849400

F -3.84522400 -3.49701600 0.35622100

**TSHI**

-1603.680798 a.u. (one imaginary freq: -136.36)

C -0.85699200 -3.38055300 2.59857000

C 0.50112800 -3.21658700 2.27529100

N -0.10680100 -2.01915700 0.29368400

C -1.42997800 -2.17049700 0.59468200

N -1.74357200 -0.94853300 -1.43110700

H -1.14865600 -3.91328800 3.49824800

H 1.28518100 -3.61660500 2.90893800

O 2.15271700 -0.72300600 -1.41233200

C 2.67571500 0.10811500 -0.61233800

O 2.11611900 0.97808800 0.08169900

Ni 0.18195900 -0.75904800 -1.21610000

C -1.82392100 -2.85346400 1.75655100

H -2.87565800 -2.96768200 1.99252600

C 0.82742600 -2.52782700 1.12020700

H 1.85989900 -2.36096200 0.82859400

C -2.34669900 -1.54509500 -0.35217100

C -3.74034100 -1.49514000 -0.19703300

H -4.20548500 -1.94923700 0.67026900

C -4.51821100 -0.86506600 -1.15679200

H -5.59714800 -0.82580600 -1.04699800

C -2.51163800 -0.32883800 -2.35725500

C -3.88904700 -0.27466300 -2.26812700

H -4.46183600 0.21966400 -3.04519300

H -1.97550200 0.13082500 -3.18135700

B 0.11028400 0.95341500 -0.11842200

O -0.15422500 2.17632600 -0.72987900

O -0.37061800 0.90024700 1.18054400

C -1.05349100 2.90723400 0.15694300

C -0.75316100 2.25578700 1.55278900

C 0.43792600 2.89416300 2.27197900

H 0.19187800 3.89429600 2.64475000

H 0.71127100 2.26834200 3.12848000

H 1.30726100 2.96625500 1.61354100

C -1.95094500 2.17212200 2.49043800

H -1.64945100 1.70268900 3.43358100

H -2.33752300 3.17177000 2.71901800

H -2.75766900 1.57504700 2.05780500

C -0.73006900 4.39228500 0.06007300

H -0.96000000 4.75691300 -0.94738700

H -1.33319800 4.96673000 0.77255700

H 0.32623200 4.58820400 0.26008600

C -2.48271500 2.64376300 -0.32441600

H -3.21165300 3.23577000 0.23927500

H -2.56070900 2.92076900 -1.38090800

H -2.74500800 1.58708300 -0.22957500

C 4.19947700 -0.06045500 -0.44272100

F 4.81455000 -0.24180500 -1.62508800

F 4.43516900 -1.15684800 0.31638600

F 4.76865800 0.99025000 0.16518600

**CF3COOBPin**

-937.412027 a.u. (zero imaginary freq)

C 3.36360600 -0.11655100 -0.12407800

O 1.83924900 1.26214200 1.14319700

C 1.98283800 0.32509400 0.40478500

O 1.04675500 -0.50640900 -0.05852300

F 3.37934400 -0.09622100 -1.46931300

F 4.32236700 0.69790400 0.32594700

F 3.64132100 -1.37036200 0.27809300

B -0.33551400 -0.25474700 -0.00546000

O -1.18742400 -1.30854600 -0.06024500

O -0.89396500 0.97888500 0.03014200

C -2.52770000 -0.74154400 0.16362700

C -2.32704400 0.76681400 -0.24320600

C -2.52035800 1.02545000 -1.73628000

H -3.57598000 0.95464900 -2.01500800

H -2.17164200 2.03599600 -1.97070800

H -1.95068700 0.31646800 -2.34583100

C -3.12643600 1.76273100 0.58161200

H -2.91564200 2.78035900 0.23735500

H -4.19882200 1.57780000 0.45972500

H -2.88002100 1.70198200 1.64403300

C -3.52357200 -1.50678400 -0.69323500

H -3.58267800 -2.54507400 -0.35098800

H -4.52024400 -1.06245600 -0.60278800

H -3.23578800 -1.50920200 -1.74685700

C -2.83444200 -0.93135200 1.64811800

H -3.84149200 -0.57816300 1.88843600

H -2.77724000 -1.99638800 1.89346100

H -2.11775500 -0.39586200 2.27929200

**K**

-1192.569355 a.u. (zero imaginary freq)

C -4.14248000 -0.00558000 0.02026900

C 3.56642300 2.93414700 0.04971400

C 2.29212000 3.49773400 -0.00087100

N 1.31224300 1.31109900 -0.03073000

C 2.54857700 0.75493600 0.01202800

N 1.33900500 -1.30929200 0.00206100

H 4.44986500 3.56371200 0.08364600

H 2.14682300 4.57237400 -0.00923000

O -2.02947000 -1.15461900 -0.04804900

C -2.60590500 -0.03891400 -0.05524000

O -2.01313400 1.07150900 -0.06918700

Ni -0.17658700 -0.01892300 -0.03966900

C 3.69736500 1.54669100 0.05710900

H 4.68032200 1.09366400 0.10138100

C 1.19113100 2.64704600 -0.03766400

H 0.17689900 3.03090900 -0.07302300

C 2.56426500 -0.72638500 0.00896500

C 3.73081000 -1.49304300 0.00884300

H 4.70471300 -1.01866000 0.00435600

C 3.63021900 -2.88299500 0.00915200

H 4.52796300 -3.49292400 0.00956500

C 1.24749400 -2.64799700 0.00220400

C 2.36747000 -3.47417400 0.00766700

H 2.24551400 -4.55173400 0.00862100

H 0.24154400 -3.05459700 -0.00264500

F -4.65627600 0.96221000 -0.76300700

F -4.70115600 -1.17130100 -0.34773300

F -4.52834800 0.25026300 1.29224800

**E**

-1705.122772 a.u. (zero imaginary freq)

C 0.77129600 -3.33559200 3.11478800

C 1.41607000 -2.12112900 3.35269300

N 1.06646900 -1.48929200 1.07611900

C 0.44498900 -2.66377400 0.82914800

N 0.26244500 -1.84364100 -1.41483900

H 0.65020400 -4.06159100 3.91251900

H 1.81260400 -1.87134300 4.33053600

C 0.28279500 -3.61388200 1.84129200

H -0.21854500 -4.55317200 1.64322600

C 1.54201200 -1.22213500 2.29868900

H 2.02827500 -0.25904000 2.41431400

C -0.04494100 -2.84333700 -0.55742700

C -0.79194100 -3.94671200 -0.97741600

H -1.05318000 -4.73585100 -0.28351800

C -1.20609300 -4.01752300 -2.30541000

H -1.79051400 -4.86612300 -2.64649200

C -0.13499000 -1.91352700 -2.68951500

C -0.86737300 -2.98928000 -3.18351100

H -1.17013400 -3.00890300 -4.22449700

H 0.13581000 -1.07261800 -3.32061400

C -0.84747500 0.69621600 -0.12834400

C -0.99034300 2.04060600 -0.18142800

H -0.08880800 2.62917800 -0.36896100

C -2.19281800 2.89001800 -0.04670900

C -2.10229000 4.23761500 -0.44287100

C -3.43359400 2.45613200 0.46567700

C -3.19254000 5.10247900 -0.35629600

H -1.15609800 4.60826200 -0.83175900

C -4.51779200 3.32432300 0.55443900

H -3.54792400 1.43566100 0.80872800

C -4.42471200 4.66312100 0.14226100

H -3.08478300 6.13571800 -0.67895600

H -5.45961300 2.95713200 0.95755900

C -1.95246600 -0.25986000 0.06302600

C -2.14967100 -0.91628000 1.29167100

C -2.76937900 -0.65549100 -1.01555600

C -3.11054900 -1.91872000 1.43237800

H -1.53348300 -0.64138000 2.14382400

C -3.71840800 -1.66387700 -0.87267200

H -2.64176200 -0.16823100 -1.97870300

C -3.90459600 -2.32303500 0.35256000

H -3.23342200 -2.40677200 2.39707700

H -4.32357000 -1.95237900 -1.72999900

C -5.60877400 5.59075600 0.25067000

H -6.48859600 5.17787100 -0.25868700

H -5.89481100 5.75252400 1.29840500

H -5.39028700 6.56860800 -0.19061500

C -4.89881400 -3.45012100 0.48484600

H -5.83904500 -3.22127000 -0.03091000

H -4.50978000 -4.37839700 0.04371000

H -5.12884900 -3.65832300 1.53526100

Ni 0.96324300 -0.12921600 -0.44769600

O 3.01380600 -0.16169700 -1.13308400

C 3.28932400 0.79619900 -0.33884600

O 2.37430500 1.34107500 0.35796500

C 4.69971500 1.27508400 -0.21873100

C 5.01682100 2.31865900 0.66145800

C 5.71321400 0.68238700 -0.98422200

C 6.33291200 2.76451900 0.77431400

H 4.22513700 2.77036700 1.24955300

C 7.02900800 1.12897700 -0.87086100

H 5.45725100 -0.12502300 -1.66187000

C 7.34043400 2.17044400 0.00835400

H 6.57420300 3.57398300 1.45755400

H 7.81144200 0.66674100 -1.46622700

H 8.36601800 2.51839000 0.09632100

**TSES**

-1705.087037 a.u. (one imaginary freq: -40.40)

6 0.195517 -3.523192 3.391963

6 -0.587610 -3.784334 2.268014

7 -0.298227 -1.526170 1.534492

6 0.439463 -1.252909 2.633828

7 0.520884 0.969742 1.742077

1 0.398862 -4.303667 4.118314

1 -1.016193 -4.764719 2.090906

6 0.710797 -2.243139 3.582220

1 1.309669 -2.022771 4.457449

6 -0.809371 -2.752356 1.360981

1 -1.402686 -2.885619 0.463038

6 0.891476 0.153356 2.756001

6 1.606252 0.638921 3.854225

1 1.909431 -0.023646 4.655603

6 1.915011 1.996424 3.914921

1 2.469910 2.390089 4.760770

6 0.793787 2.277958 1.819178

6 1.488919 2.837033 2.889137

1 1.690364 3.902513 2.905410

1 0.443356 2.888800 0.996809

6 1.086400 -0.370527 -1.115392

6 0.459171 0.760493 -1.618407

1 -0.343103 0.655151 -2.355735

6 0.979430 2.130505 -1.453250

6 0.174942 3.243107 -1.749315

6 2.282320 2.369388 -0.976862

6 0.645209 4.540991 -1.550526

1 -0.834828 3.083384 -2.121004

6 2.747584 3.666085 -0.784792

1 2.922806 1.522500 -0.746143

6 1.937185 4.778998 -1.061018

1 -0.001783 5.385485 -1.776757

1 3.756665 3.822143 -0.409138

6 1.950753 -1.414872 -1.463205

6 2.647666 -1.386972 -2.712205

6 2.217509 -2.524117 -0.603904

6 3.531840 -2.394945 -3.063446

1 2.469963 -0.554373 -3.387229

6 3.104033 -3.520656 -0.975947

1 1.715059 -2.577026 0.354651

6 3.784681 -3.484215 -2.209084

1 4.042533 -2.345359 -4.022871

1 3.281574 -4.353628 -0.298685

6 2.451502 6.180637 -0.848511

1 1.655934 6.921081 -0.980366

1 2.868196 6.304913 0.158637

1 3.254226 6.423169 -1.557623

6 4.770298 -4.560153 -2.582491

1 5.742060 -4.399318 -2.093950

1 4.418618 -5.551389 -2.272809

1 4.948405 -4.581485 -3.662914

28 -0.357897 -0.053764 0.122980

8 -2.337938 0.724199 0.353226

6 -2.799087 -0.171728 -0.424229

8 -2.036020 -1.032589 -0.962102

6 -4.270648 -0.209325 -0.708103

6 -4.800385 -1.199139 -1.546958

6 -5.127344 0.741965 -0.137810

6 -6.168694 -1.236980 -1.812837

1 -4.128969 -1.931102 -1.983195

6 -6.495731 0.704432 -0.403742

1 -4.708565 1.503926 0.510951

6 -7.018588 -0.285002 -1.241780

1 -6.573302 -2.006786 -2.464261

1 -7.154880 1.445004 0.040921

1 -8.084851 -0.314138 -1.449123

**S**

-1705.126604 a.u. (zero imaginary freq)

C 3.68694000 -2.55923800 -1.82729800

C 2.49485900 -2.43424400 -2.53945300

N 1.44288300 -1.48516600 -0.61656200

C 2.58642100 -1.61880400 0.09271600

N 1.34774800 -0.58053900 1.86119000

H 4.57316200 -2.96953500 -2.30085200

H 2.41801700 -2.74405500 -3.57576200

C 3.73596800 -2.15066500 -0.49644500

H 4.65668800 -2.23972000 0.06645600

C 1.39250700 -1.88367100 -1.89238500

H 0.44042000 -1.73895900 -2.39206800

C 2.52190800 -1.14094400 1.49351000

C 3.58868500 -1.23091400 2.39268600

H 4.52852900 -1.67763300 2.09251300

C 3.43135600 -0.73066200 3.68171700

H 4.25103400 -0.78958600 4.39079600

C 1.19765500 -0.09895200 3.10118500

C 2.21470300 -0.15384800 4.04861400

H 2.05345900 0.24617400 5.04354800

H 0.23334900 0.34595700 3.32661400

C 0.80738500 1.53211500 -0.28587500

C -0.00004800 2.60215600 -0.42613600

H 0.35939300 3.54637100 -0.85299400

C -1.43739900 2.54697100 -0.08759600

C -2.41158500 3.10607700 -0.93082500

C -1.88657400 1.85204500 1.04970500

C -3.77058300 2.90132600 -0.69361600

H -2.09730300 3.66520000 -1.80961000

C -3.24525200 1.64774000 1.28419900

H -1.15781800 1.47332900 1.76374600

C -4.21327300 2.15130600 0.40629800

H -4.50281600 3.31297300 -1.38498700

H -3.55842900 1.07726300 2.15551100

C 2.23883500 1.56524000 -0.62946900

C 2.68181200 1.29270000 -1.93957500

C 3.22459000 1.72464700 0.36119100

C 4.03676200 1.16574700 -2.23379000

H 1.94423100 1.15604300 -2.72651200

C 4.58223300 1.60198300 0.05996900

H 2.91903600 1.92516200 1.38504100

C 5.01589400 1.30690700 -1.23777500

H 4.34295000 0.94264300 -3.25411500

H 5.31719100 1.72359600 0.85310300

C -5.68150100 1.90207500 0.64612000

H -5.84787300 0.91083000 1.08156800

H -6.10651400 2.63907200 1.34170300

H -6.25314100 1.96491800 -0.28566500

C 6.47828500 1.11603600 -1.55654200

H 7.11341200 1.44920900 -0.72891700

H 6.70985300 0.05892800 -1.74602900

H 6.77179300 1.67242800 -2.45514300

Ni 0.04720200 -0.26337300 0.26164400

O -1.64658000 -1.49048900 0.83499100

C -2.22571700 -1.17110700 -0.25644000

O -1.58454800 -0.58762500 -1.18454200

C -3.68919300 -1.43003100 -0.41910600

C -4.36062800 -0.95715200 -1.55470400

C -4.40969700 -2.08696900 0.58675300

C -5.73607900 -1.14235200 -1.68454700

H -3.79538900 -0.43437300 -2.31833100

C -5.78556800 -2.27563000 0.45506600

H -3.88324600 -2.43880100 1.46747400

C -6.45062000 -1.80276500 -0.68013800

H -6.25262300 -0.76891400 -2.56436700

H -6.33975200 -2.78674600 1.23739300

H -7.52314300 -1.94521900 -0.78022700

**CF_3_COO^-^**

-526.338604 a.u. (zero imaginary freq)

O 1.55624900 1.14097100 -0.01322800

C 1.05176900 -0.00001100 -0.03150600

O 1.55631700 -1.14096400 -0.01320600

C -0.51622800 -0.00003200 -0.01060400

F -0.98358800 0.00048500 1.27399800

F -1.07006500 1.08492500 -0.61161900

F -1.07009900 -1.08538800 -0.61080900

**M**

-1284.90267 a.u. (zero imaginary freq)

C 4.18640400 2.76756600 -0.84916400

C 2.82199800 2.99806400 -1.01749100

N 2.35116500 0.71382300 -0.46514800

C 3.67662200 0.48004800 -0.31133600

N 2.95359500 -1.74159400 0.19437000

H 4.90626500 3.56705400 -0.99350700

H 2.44161600 3.97428000 -1.29792300

C 4.62141600 1.49254000 -0.49370000

H 5.67831800 1.29516600 -0.36072600

C 1.93857500 1.94128300 -0.81342200

H 0.86832100 2.06938200 -0.92599200

C 4.01934700 -0.91248600 0.05261900

C 5.32498300 -1.37142800 0.23704500

H 6.16676200 -0.69978600 0.11977200

C 5.53686400 -2.70695500 0.57135000

H 6.54626500 -3.07826400 0.71678100

C 3.16521900 -3.02907700 0.51746200

C 4.43816100 -3.55392700 0.71386800

H 4.55784400 -4.60031700 0.97230300

H 2.27772600 -3.64500900 0.61955100

Ni 1.16564900 -0.92238300 -0.05952400

C -0.75355500 -0.60342000 -0.04176400

C -1.69174400 -1.57092200 -0.24136800

H -1.33842500 -2.56889100 -0.51271800

C -3.16622700 -1.50542300 -0.17010000

C -3.91877200 -2.48117100 -0.85116200

C -3.88766200 -0.54797500 0.57419300

C -5.31288900 -2.48352800 -0.82345300

H -3.39459100 -3.24917000 -1.41640800

C -5.27896600 -0.55691800 0.60335400

H -3.35316900 0.19917200 1.14840700

C -6.02309400 -1.51685000 -0.10110800

H -5.85889600 -3.25043800 -1.36865100

H -5.80248800 0.19329100 1.19284900

C -1.12841600 0.81099100 0.18911600

C -0.78090900 1.48183500 1.37743600

C -1.74172400 1.57716900 -0.82164400

C -1.04641600 2.83950700 1.55008400

H -0.29277300 0.92377900 2.17353800

C -1.98915700 2.93922100 -0.65191600

H -2.02038200 1.09203000 -1.75386200

C -1.64723200 3.60008300 0.53630800

H -0.77281800 3.32237200 2.48630100

H -2.46005800 3.50084200 -1.45647300

C -7.53091200 -1.49929100 -0.07763000

H -7.92884800 -0.64528900 -0.64241200

H -7.94718000 -2.41076100 -0.51912100

H -7.91537900 -1.41032500 0.94584800

C -1.88321500 5.08063800 0.70638800

H -0.99145400 5.66343300 0.43580500

H -2.70350100 5.43014200 0.06973400

H -2.12682500 5.33097400 1.74524600

**TSMN**

-1811.210597 a.u. (one imaginary freq: -100.72)

C -4.33714100 -0.23983200 1.67794500

C -3.22833400 0.25463300 2.36361200

N -1.77316400 -0.66196000 0.69788700

C -2.84031200 -1.16257600 0.03878900

N -1.21680300 -1.80769000 -1.58992100

H -5.34201200 -0.06438400 2.04962200

H -3.33718600 0.81903000 3.28354600

C -4.14386300 -0.96643900 0.50479100

H -4.99507200 -1.35587700 -0.04116000

C -1.96092200 0.01387100 1.83730900

H -1.05745000 0.35862000 2.32600300

C -2.50859900 -1.90164800 -1.20244200

C -3.43416800 -2.65320700 -1.93331000

H -4.46543700 -2.72925500 -1.60972000

C -3.01026300 -3.31770000 -3.08179200

H -3.71438100 -3.90788800 -3.66008700

C -0.81393400 -2.44981100 -2.69301200

C -1.67502700 -3.21884500 -3.47224600

H -1.30396000 -3.72331400 -4.35790400

H 0.23407100 -2.33355700 -2.95402300

C 1.03294900 0.92694100 -0.96440600

C -0.26497900 1.28214700 -1.37173900

H -0.60502100 1.12268300 -2.40880900

C -1.20054400 2.14147300 -0.60644800

C -2.54699000 2.25958900 -0.99598800

C -0.80439800 2.82980600 0.55436800

C -3.45884900 2.99705600 -0.24307900

H -2.88252300 1.74395700 -1.89436300

C -1.71461300 3.57889900 1.29745800

H 0.23300600 2.75905000 0.87133900

C -3.06366800 3.66769200 0.92379300

H -4.49803700 3.05136500 -0.56223200

H -1.37541200 4.10069900 2.19057000

C 2.36758600 1.36213600 -0.99133000

C 2.73204300 2.64937900 -1.50919600

C 3.46176900 0.57137300 -0.50239900

C 4.05118800 3.08962600 -1.52457400

H 1.94435900 3.29628000 -1.89050900

C 4.76749200 1.03372300 -0.52878400

H 3.24698800 -0.41788600 -0.10966000

C 5.10933000 2.30432500 -1.03813300

H 4.27043300 4.07824300 -1.92834000

H 5.55908000 0.38998000 -0.14400300

C -4.06176000 4.42071100 1.76802700

H -4.52867300 3.76261200 2.51481700

H -3.58602000 5.24242100 2.31511600

H -4.87033500 4.83960100 1.15823500

C 6.53977200 2.78371500 -1.06256800

H 6.99430900 2.78680100 -0.06127600

H 7.18144000 2.14970400 -1.69241300

H 6.60745900 3.80514200 -1.45487600

Ni -0.01639000 -0.48918000 -0.46186200

O 1.15653200 -1.86567700 0.41451700

C 1.49343300 -1.71524700 1.63017200

O 1.13474000 -0.89526600 2.47925500

C 2.56934600 -2.75397200 2.04757900

F 2.18286800 -4.02288800 1.77417800

F 2.86717900 -2.70604000 3.36055600

F 3.72880200 -2.54094000 1.37103600

**N**

-1284.908165 a.u. (zero imaginary freq)

C 3.86317900 0.08692800 2.01386300

C 2.82974100 0.99338000 2.25294500

N 1.30394700 -0.41295500 1.05853300

C 2.29446100 -1.30935300 0.84398700

N 0.59918900 -2.47259000 -0.37491700

H 4.86797300 0.28519100 2.37354500

H 2.99790200 1.91304300 2.80233600

C 3.59347500 -1.08270400 1.30667600

H 4.38364300 -1.79791200 1.11267500

C 1.56318200 0.69986600 1.75667800

H 0.72969500 1.37961200 1.89095600

C 1.88038600 -2.50476500 0.07823200

C 2.71210000 -3.59827700 -0.17157400

H 3.73150700 -3.60942400 0.19546400

C 2.21541900 -4.68101300 -0.89327600

H 2.84872800 -5.53927200 -1.09385200

C 0.12675000 -3.52254400 -1.06955900

C 0.89720900 -4.64604100 -1.34868800

H 0.46941500 -5.46874000 -1.91103900

H -0.90105200 -3.44306600 -1.40862800

Ni -0.36936000 -0.77307600 -0.07291300

C -1.57095500 0.76611600 -0.24082700

C -1.11694500 2.03402700 -0.45249900

H -1.79796700 2.89034100 -0.36714000

C 0.28661300 2.40422600 -0.68886000

C 0.79956800 3.60657900 -0.16903600

C 1.18589800 1.56882900 -1.38122000

C 2.15696500 3.91382900 -0.25591200

H 0.12739800 4.28805200 0.34843600

C 2.53961500 1.87896900 -1.47048100

H 0.81031800 0.65829800 -1.84060800

C 3.05806600 3.04589400 -0.88903000

H 2.52728800 4.83721000 0.18412600

H 3.20979200 1.20325300 -1.99833300

C -3.03290400 0.59455900 -0.05883200

C -3.53881100 -0.34987200 0.85995500

C -3.98469500 1.33147100 -0.79336400

C -4.90638100 -0.52041000 1.05854600

H -2.83123800 -0.94698200 1.43246700

C -5.35509200 1.14826100 -0.60463300

H -3.64047100 2.04637300 -1.53624400

C -5.84526200 0.22361400 0.32658800

H -5.25574000 -1.24846100 1.78830700

H -6.05906100 1.73139900 -1.19518700

C 4.53611400 3.34105400 -0.93195200

H 4.96226000 3.12308800 -1.91837400

H 4.74321700 4.38971200 -0.69398100

H 5.08081800 2.72341400 -0.20422800

C -7.32604900 0.00892300 0.51943900

H -7.59396000 -0.00320200 1.58302000

H -7.90983800 0.79628000 0.03111400

H -7.65038300 -0.95255600 0.09842000
