# Supplementary material for: Modulation of metal species as control point for Ni-catalyzed stereodivergent semihydrogenation of alkynes with water
Source: Nat Commun. 2023 Mar 24;14:1655. doi: 10.1038/s41467-023-37022-w (PMC10039052; doi:10.1038/s41467-023-37022-w)
Supplement: Supplementary file 5 — Supplementary Dataset 2 [file 41467_2023_37022_MOESM5_ESM.docx]

**SUPPLEMENTARY DATA 2**

**Modulation of metal species as control point for Ni-catalyzed stereodivergent semihydrogenation of alkynes with water**

Yuanqi Wu^1^, Yuhui Ao^1^, Zhiming Li^2^*, Chunhui Liu^3^, Jinbo Zhao^1^, Wenyu Gao^1^, Xuemeng Li^1^, Hui Wang^1^, Yongsheng Liu^1^ & Yu Liu^1^*

^1^Jilin Provincial Key Laboratory of Carbon Fiber Development and Application, College of Chemistry and Life Science, Advanced Institute of Materials Science, Changchun University of Technology, 130012 Changchun, PR China. ^2^Department of Chemistry, Fudan University, 200438 Shanghai, PR China. ^3^College of Chemical and Materials Engineering, Xuchang University, 461000 Xuchang, PR China. ^✉^email: zmli@fudan.edu.cn; yuliu@ccut.edu.cn

## ^1^H , ^19^F, ^13^C NMR Spectra


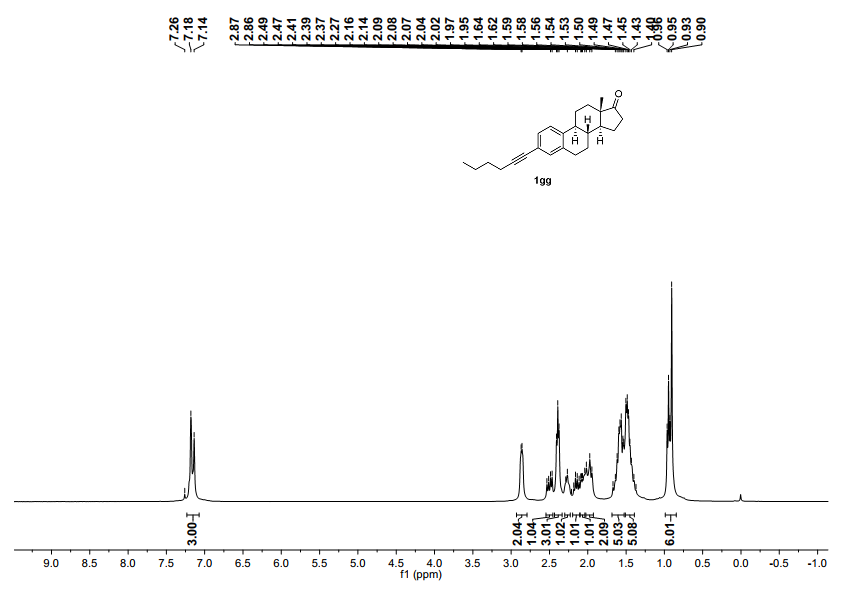
Supplementary Figure 1. ^1^H NMR (400 MHz, CDCl_3_) spectra for compound **1gg**


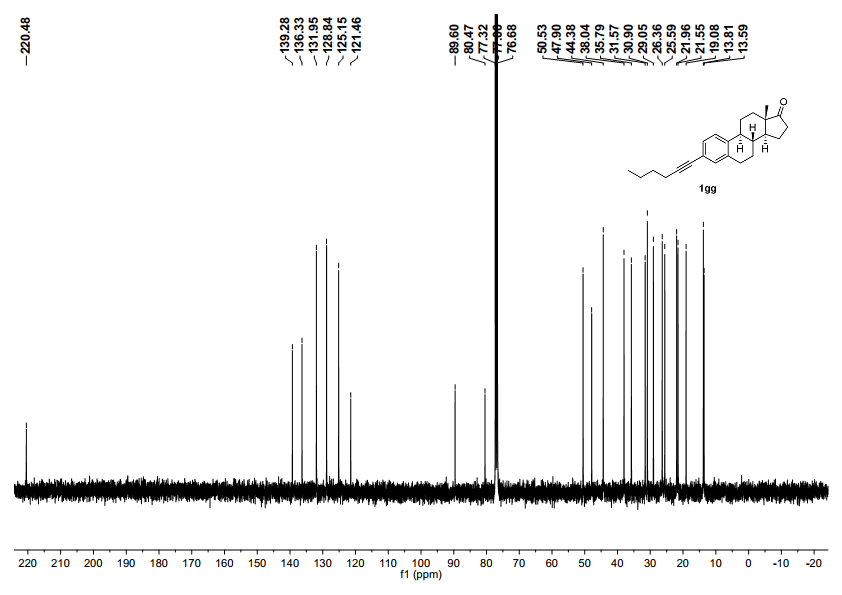
Supplementary Figure 2. ^13^C NMR (101 MHz, CDCl_3_) spectra for compound **1gg**


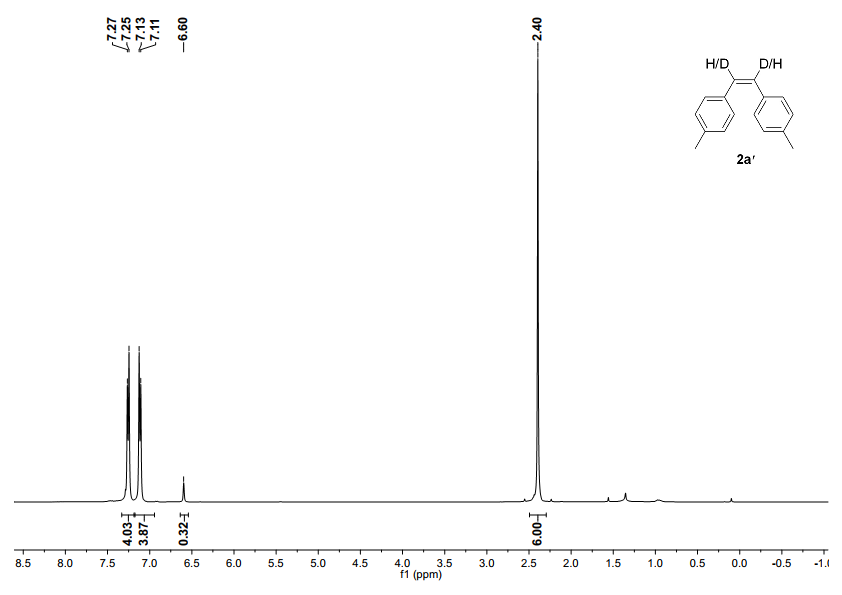
 Supplementary Figure 3. ^1^H NMR (400 MHz, CDCl_3_) spectra for compound **2a'**


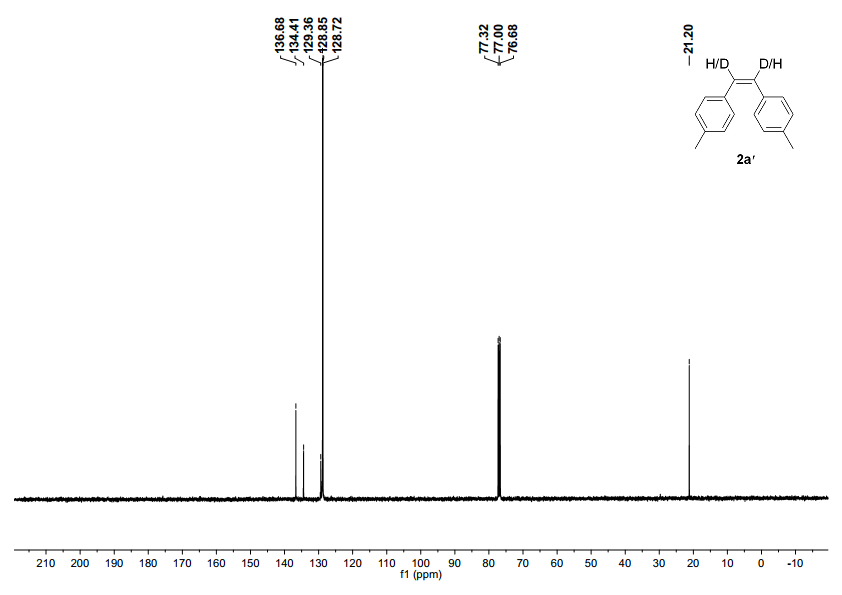
 Supplementary Figure 4. ^13^C NMR (101 MHz, CDCl_3_) spectra for compound **2a'
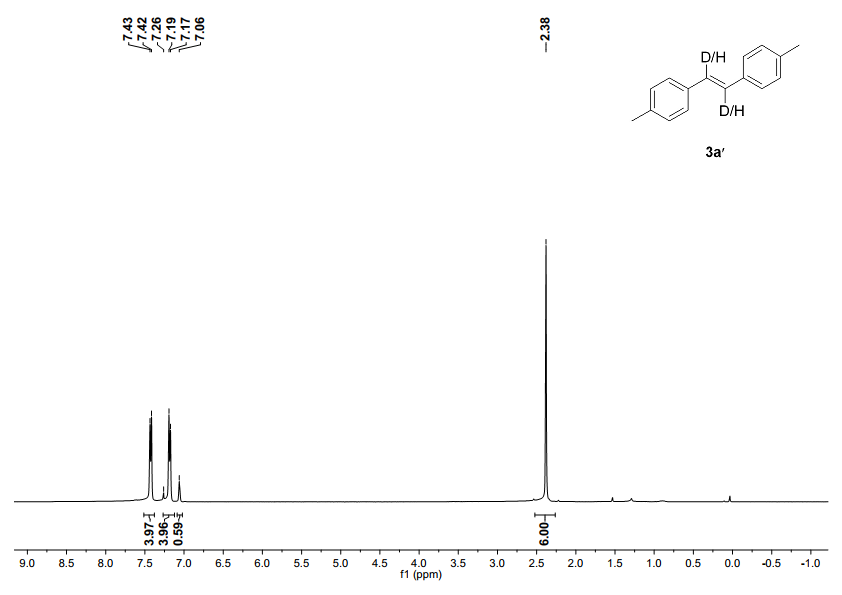
** Supplementary Figure 5. ^1^H NMR (400 MHz, CDCl_3_) spectra for compound **3a'**
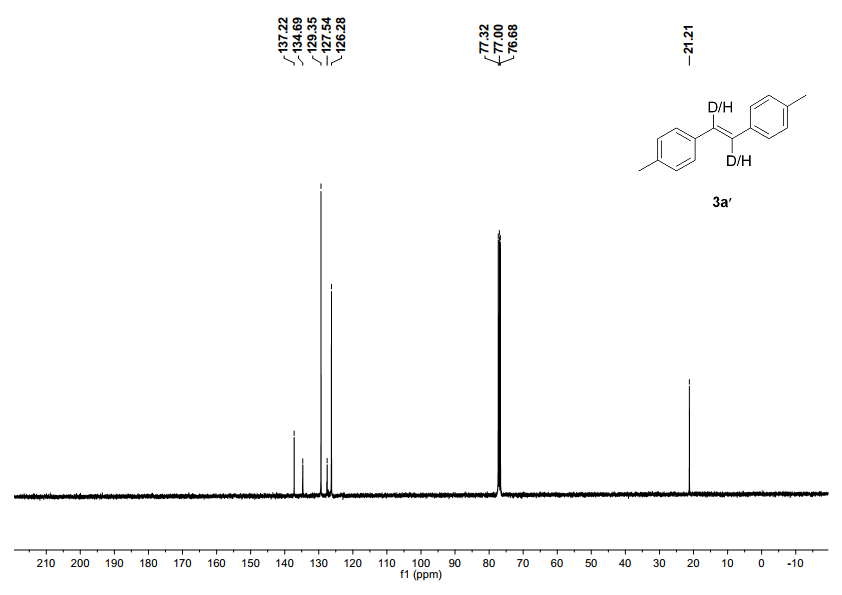
 Supplementary Figure 6. ^13^C NMR (101 MHz, CDCl_3_) spectra for compound **3a'**


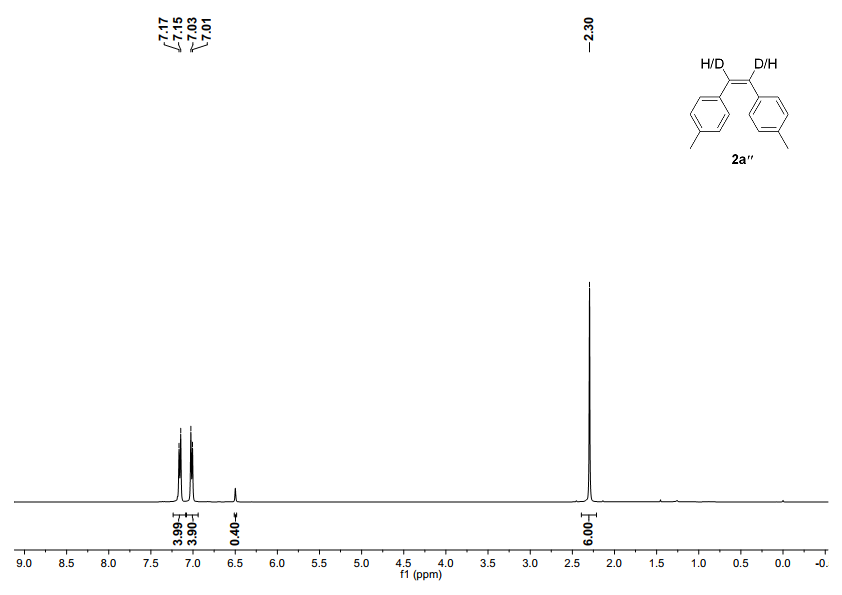
 Supplementary Figure 7. ^1^H NMR (400 MHz, CDCl_3_) spectra for compound **2a''**
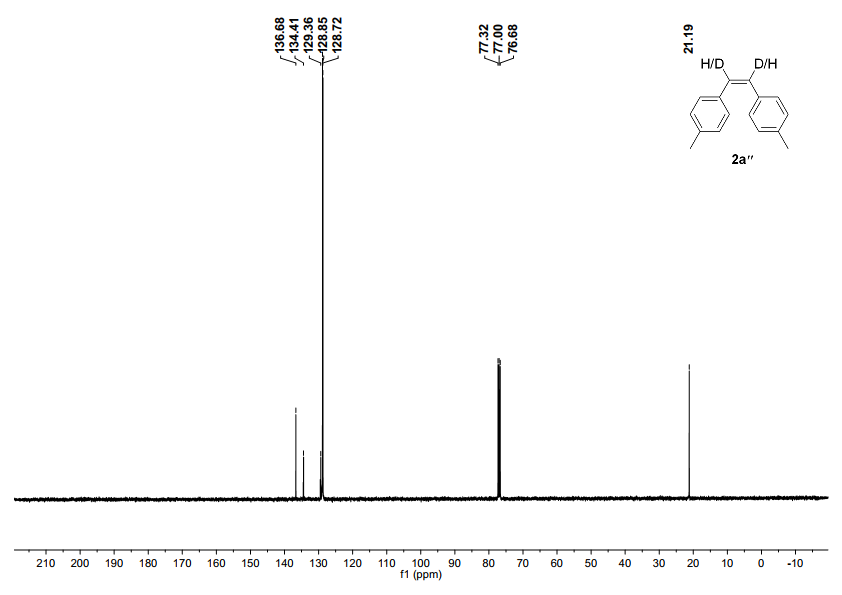
 Supplementary Figure 8. ^13^C NMR (101 MHz, CDCl_3_) spectra for compound **2a''**
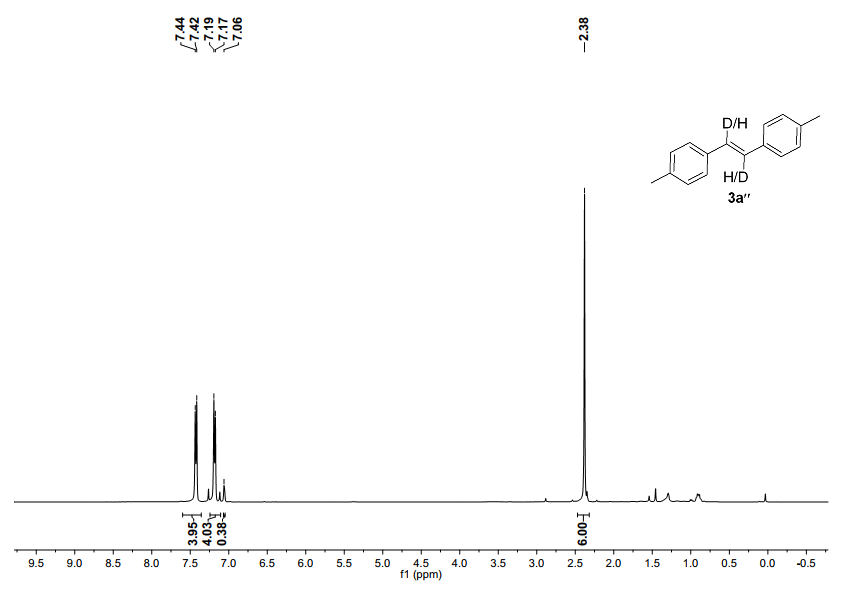
 Supplementary Figure 9. ^1^H NMR (400 MHz, CDCl_3_) spectra for compound **3a''**
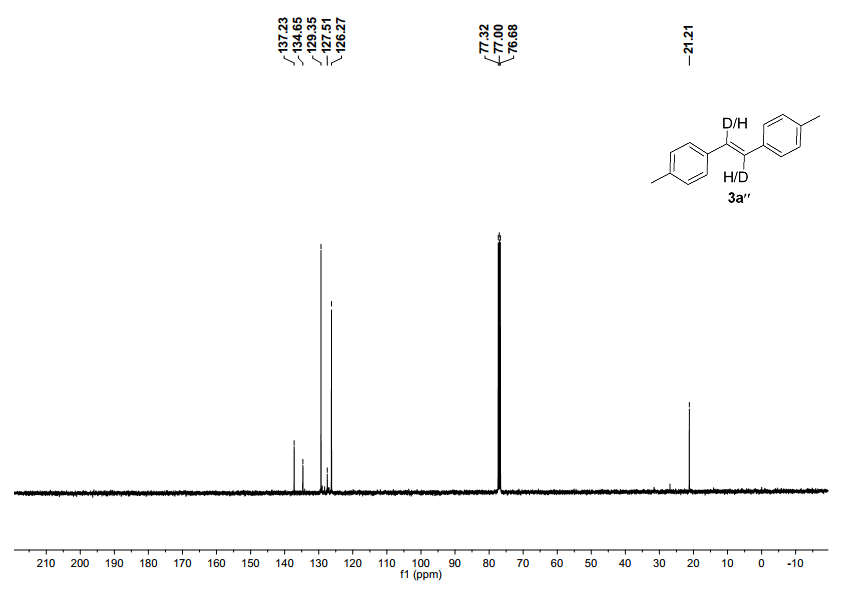
 Supplementary Figure 10. ^13^C NMR (101 MHz, CDCl_3_) spectra for compound **3a''**
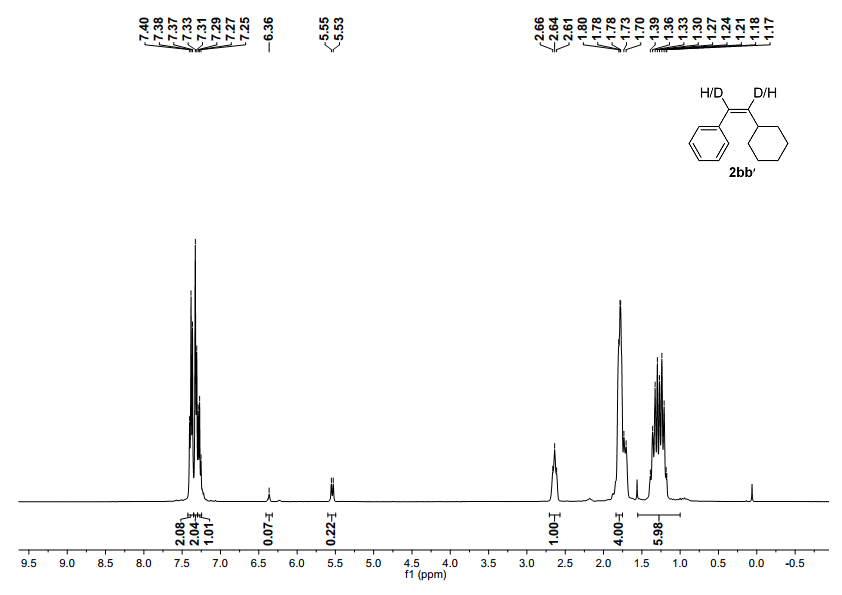
 Supplementary Figure 11. ^1^H NMR (400 MHz, CDCl_3_) spectra for compound **2bb'**
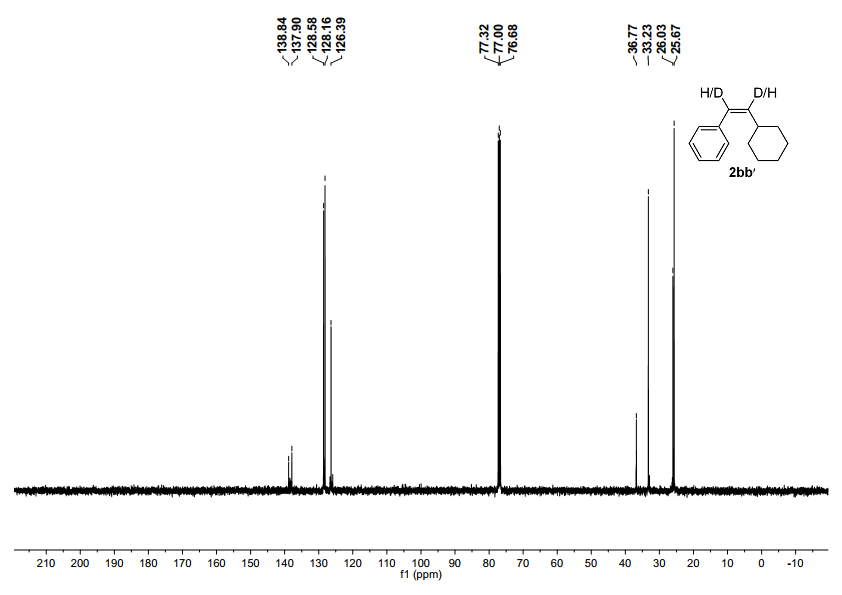
 Supplementary Figure 12. ^13^C NMR (101 MHz, CDCl_3_) spectra for compound **2bb'**
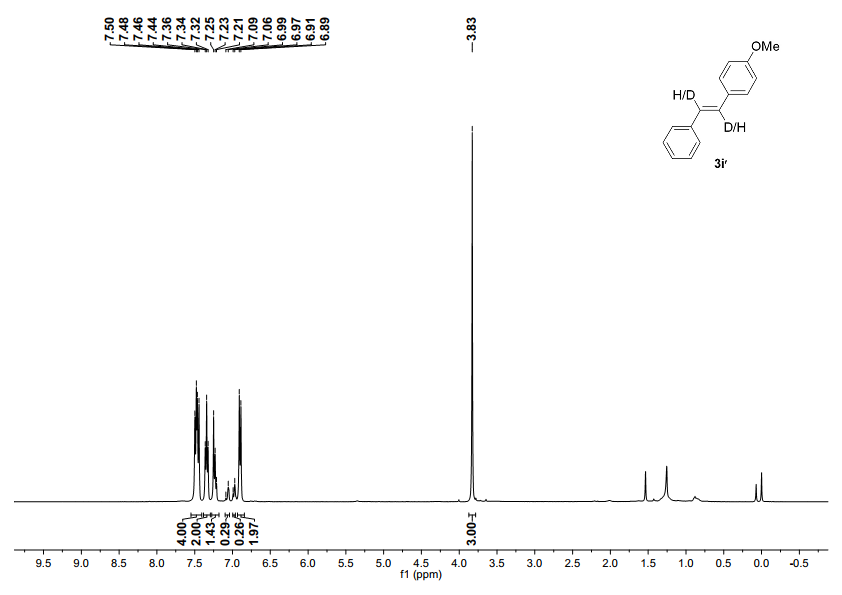
 Supplementary Figure 13. ^1^H NMR (400 MHz, CDCl_3_) spectra for compound **3i'**
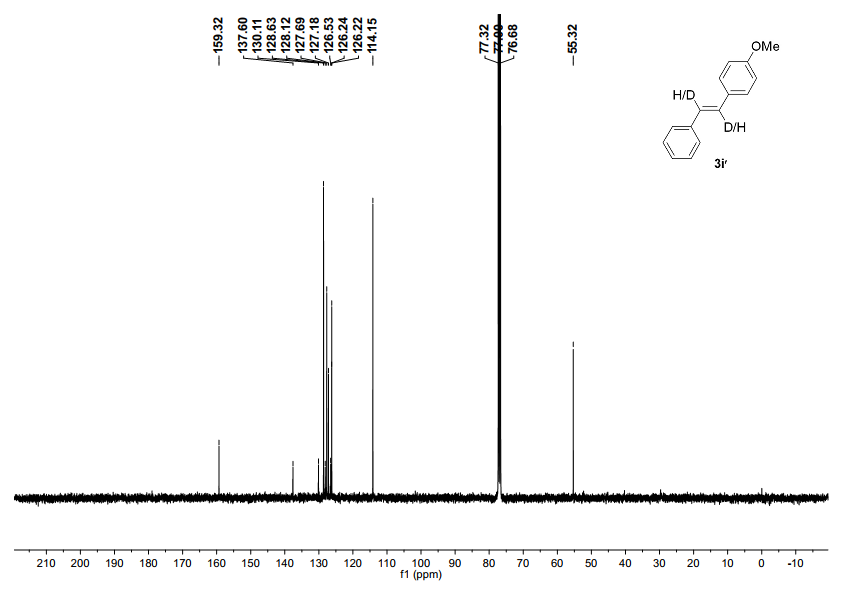
 Supplementary Figure 14. ^13^C NMR (101 MHz, CDCl_3_) spectra for compound **3i'**
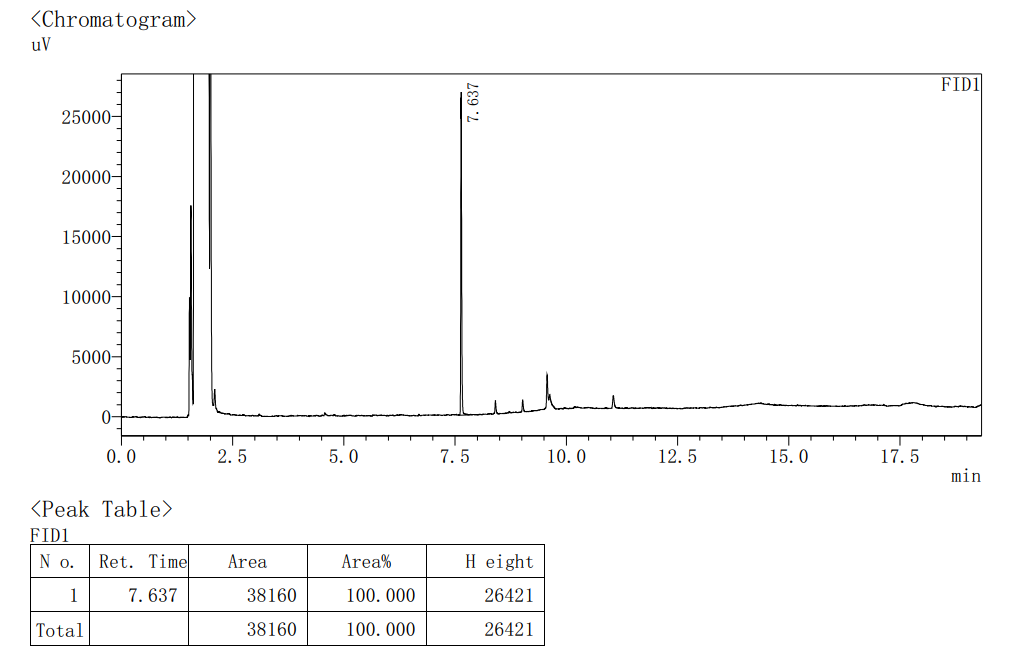

Supplementary Figure 15. GC spectra for **2a**
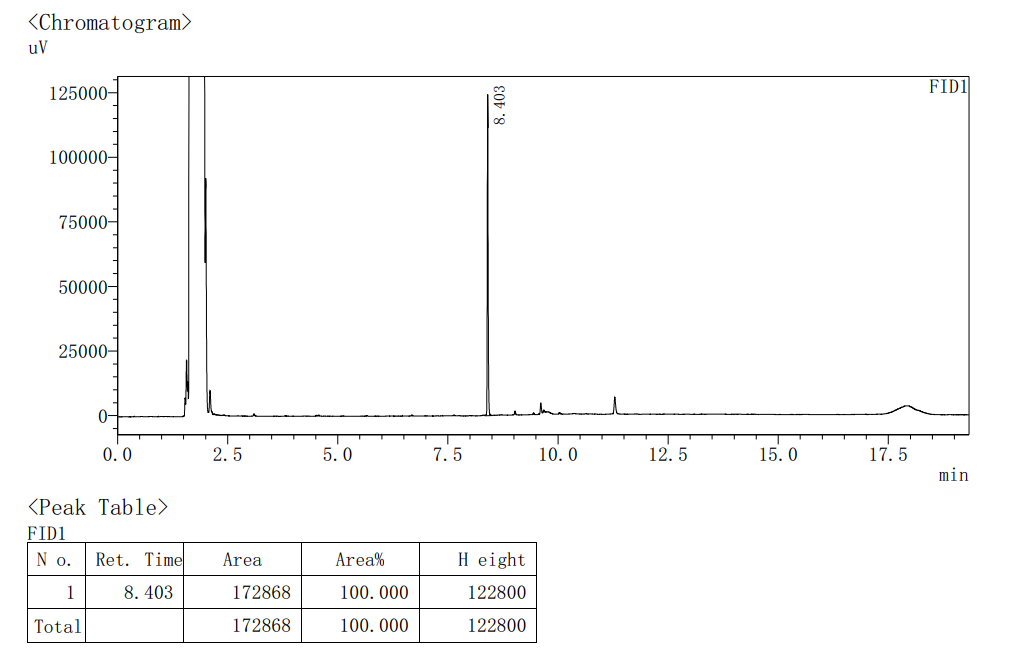
 Supplementary Figure 16. GC spectra for **3a**
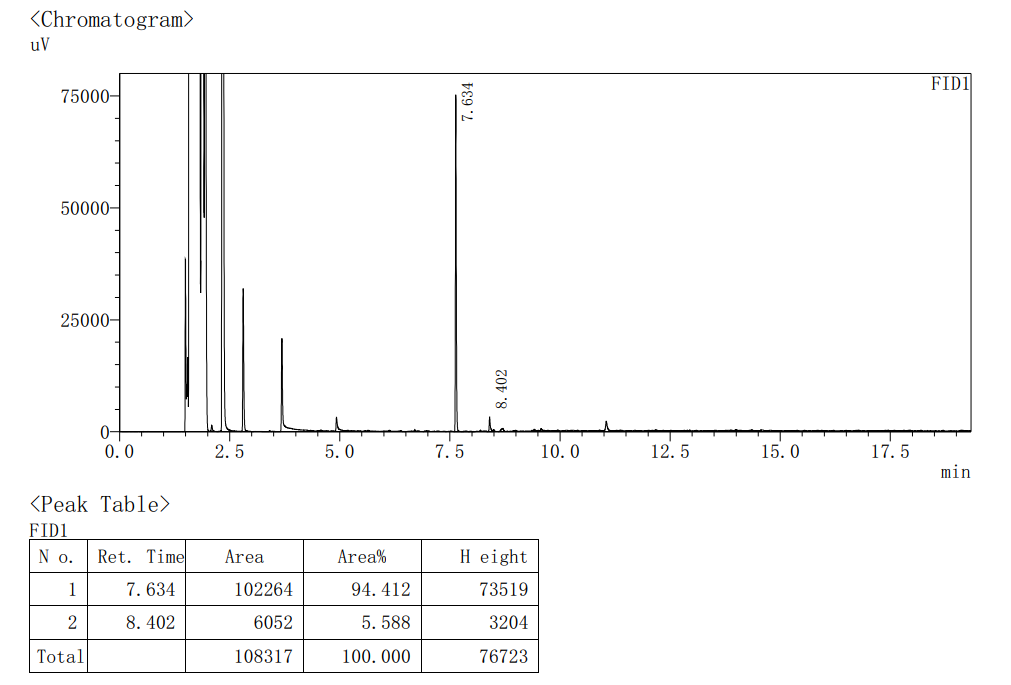


Supplementary Figure 17. GC spectra for crude product of **2a**


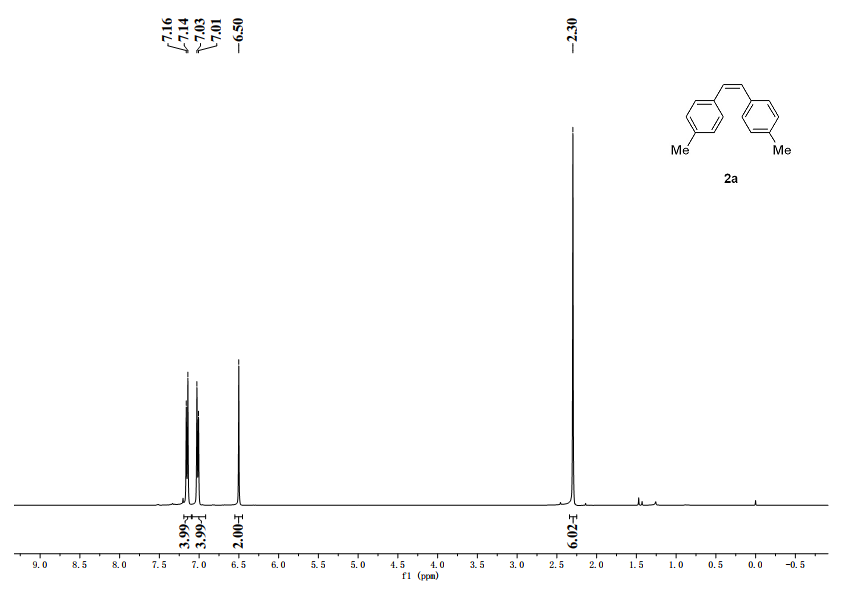
 Supplementary Figure 18. ^1^H NMR (400 MHz, CDCl_3_) spectra for compound **2a**
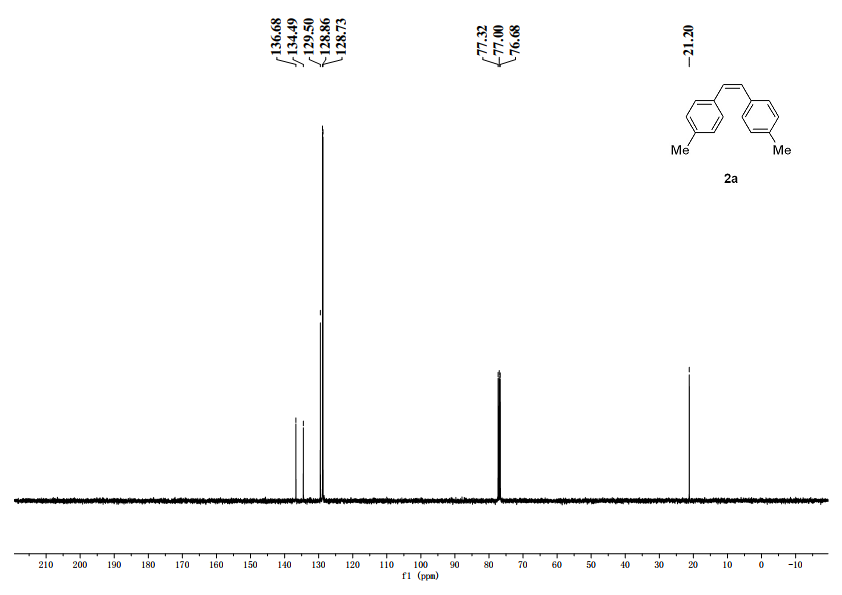
 Supplementary Figure 19. ^13^C NMR (101 MHz, CDCl_3_) spectra for compound **2a**


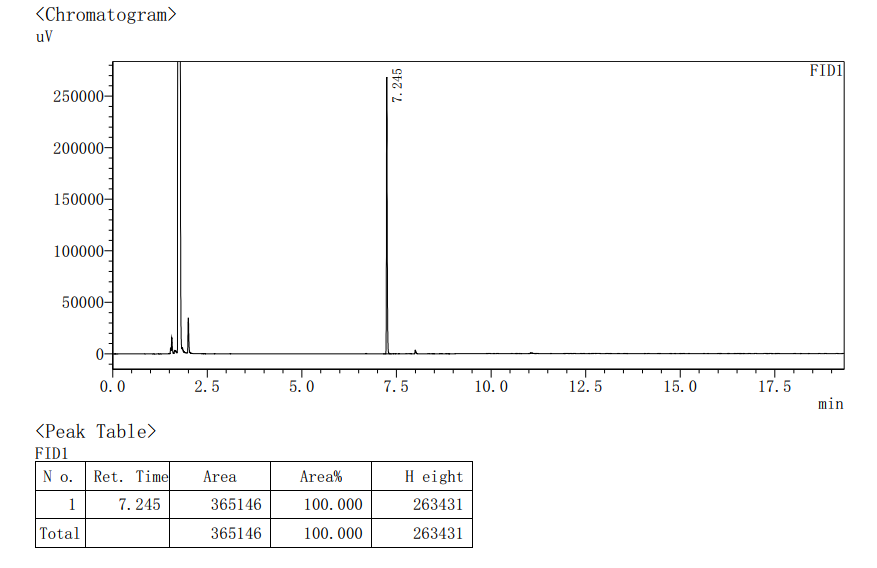
 Supplementary Figure 20. GC spectra for **2b**


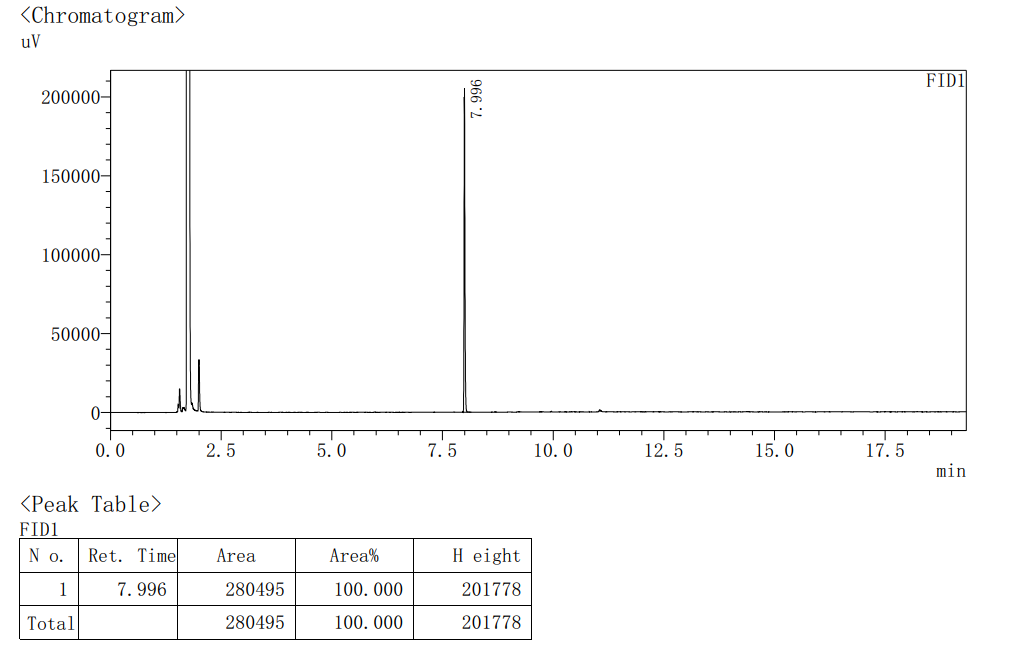
 Supplementary Figure 21. GC spectra for **3b**


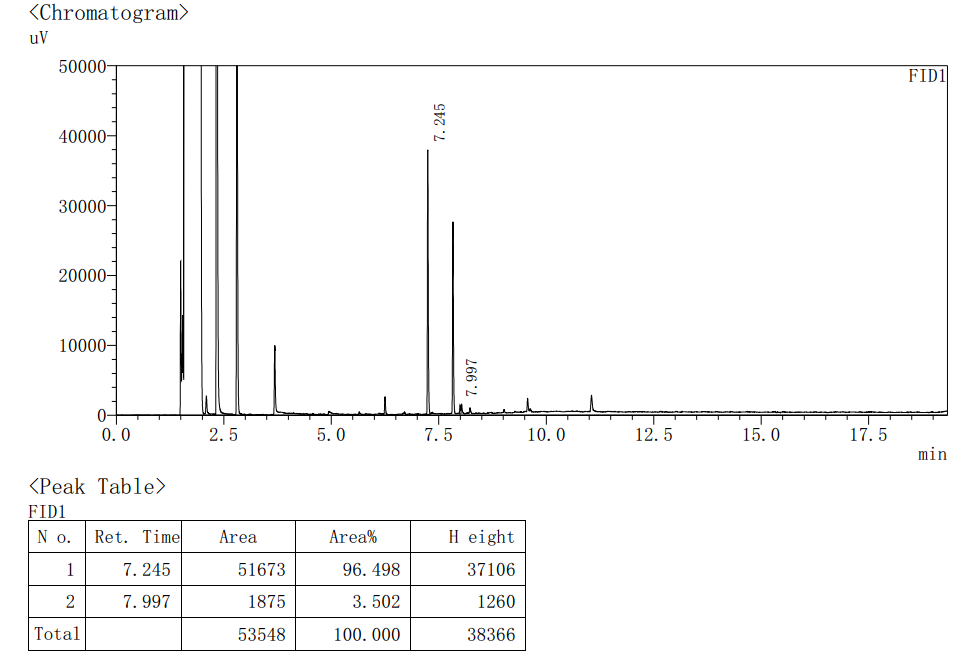
 Supplementary Figure 22. GC spectra for crude product of **2b**
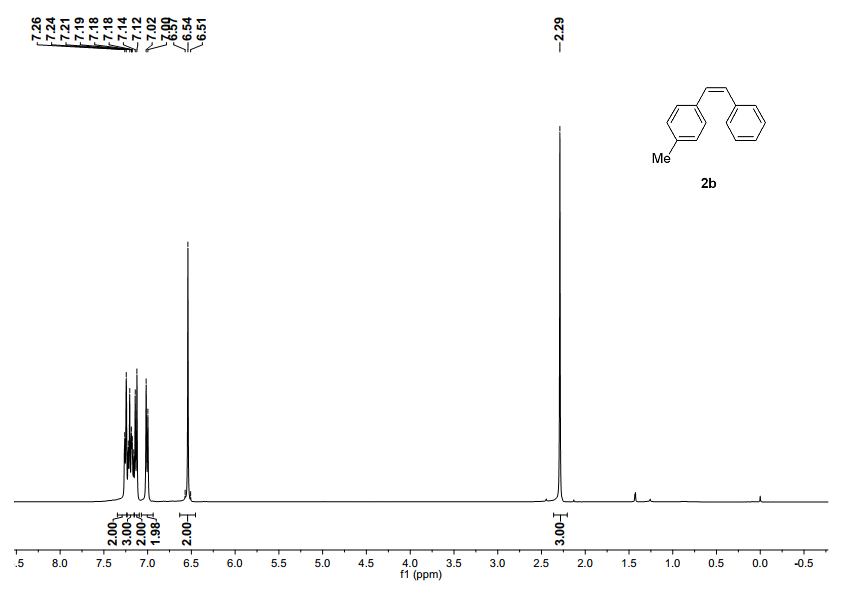
 Supplementary Figure 23. ^1^H NMR (400 MHz, CDCl_3_) spectra for compound **2b**
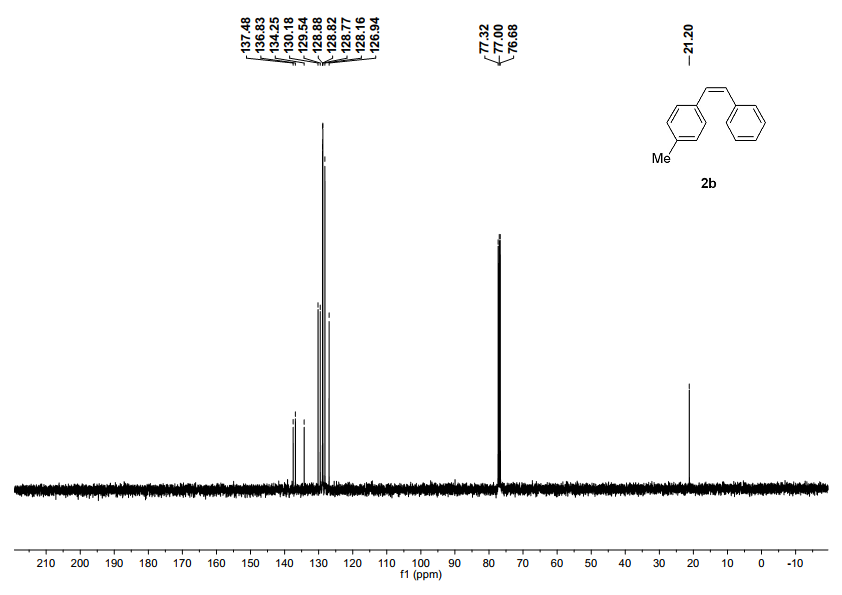
 Supplementary Figure 24. ^13^C NMR (101 MHz, CDCl_3_) spectra for compound **2b**


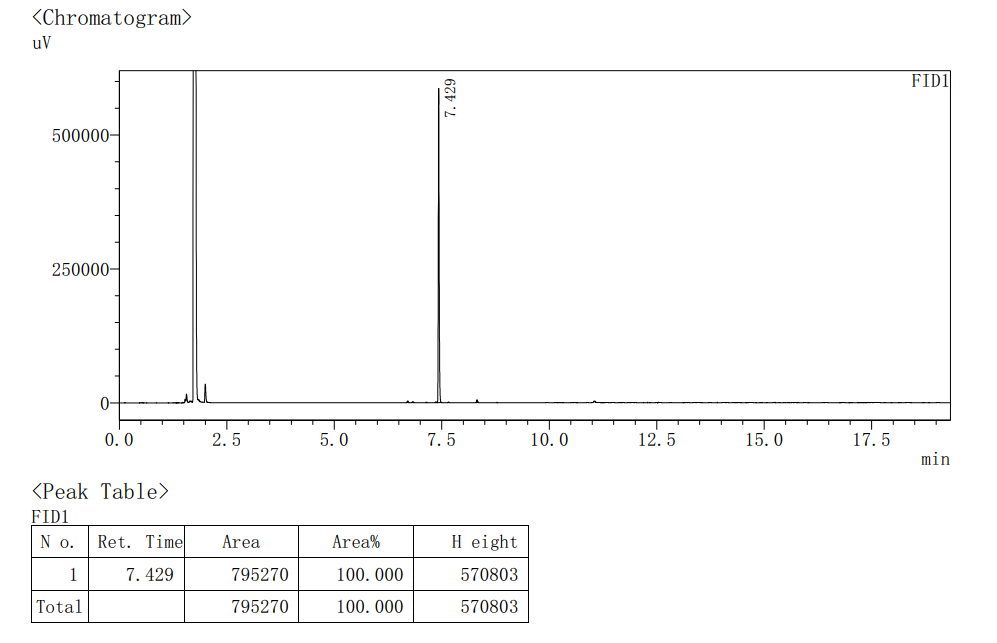
 Supplementary Figure 25. GC spectra for **2c**
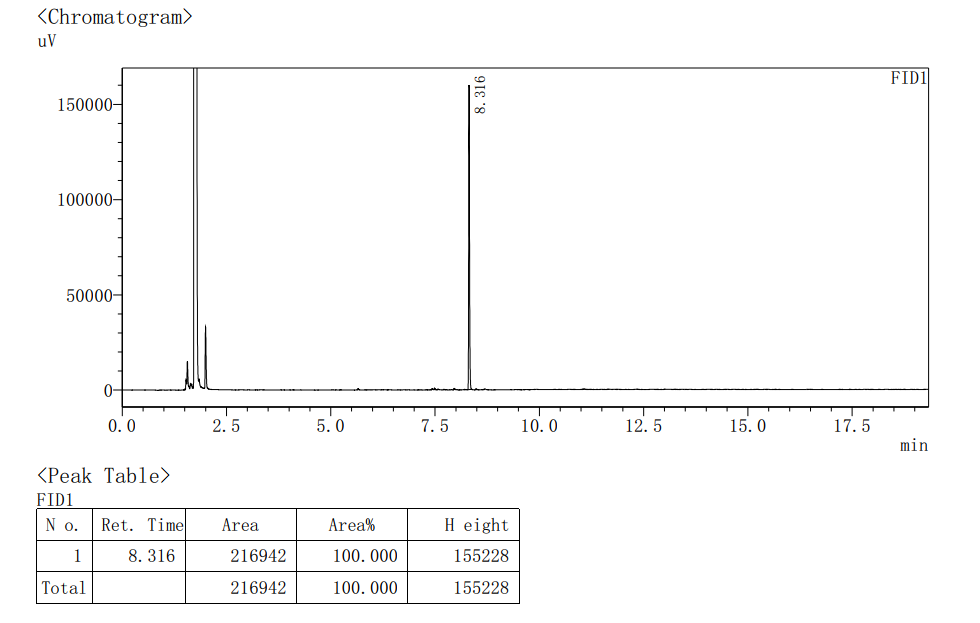
 Supplementary Figure 26. GC spectra for **3c**
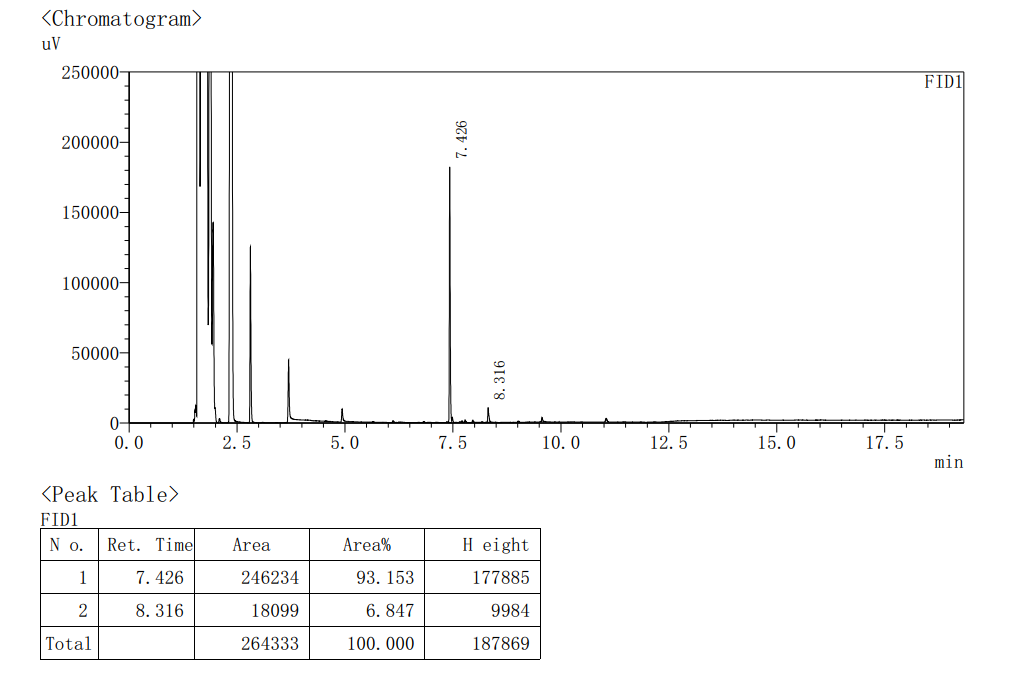
 Supplementary Figure 27. GC spectra for crude product of **2c**
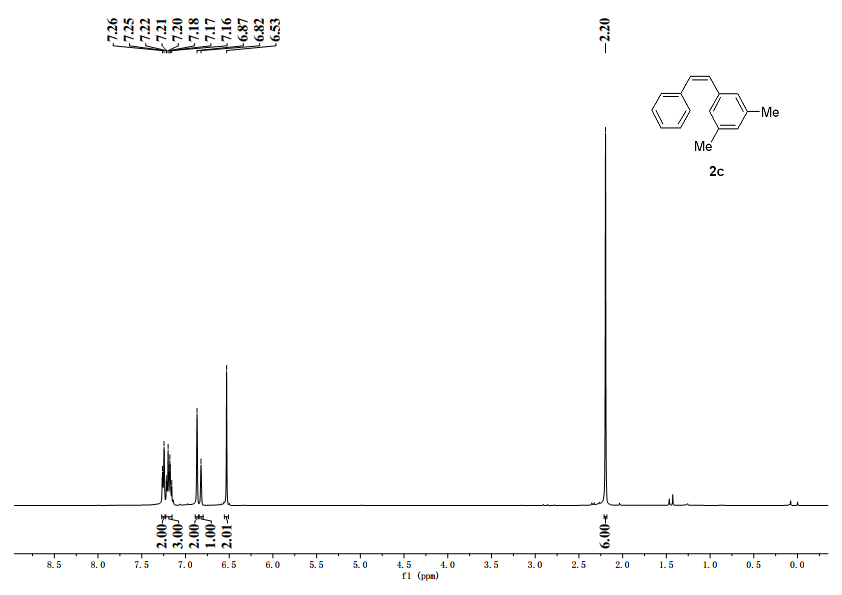
 Supplementary Figure 28. ^1^H NMR (400 MHz, CDCl_3_) spectra for compound **2c**
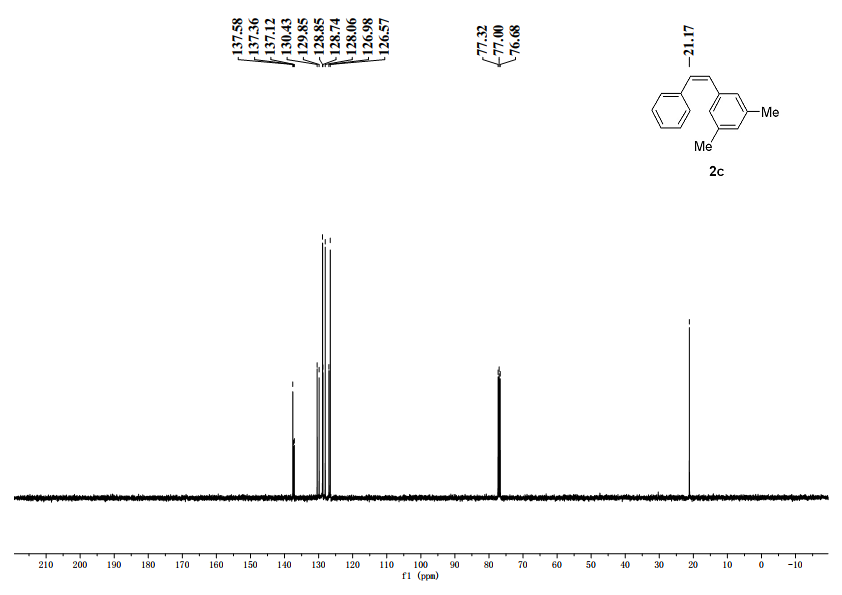
 Supplementary Figure 29. ^13^C NMR (101 MHz, CDCl_3_) spectra for compound **2c**


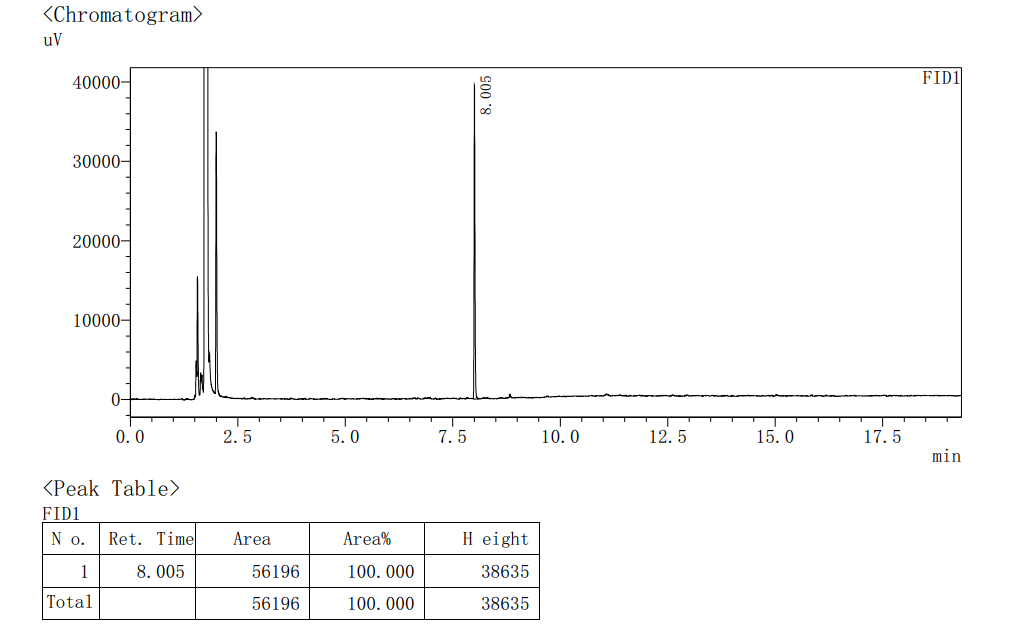
 Supplementary Figure 30. GC spectra for **2d**
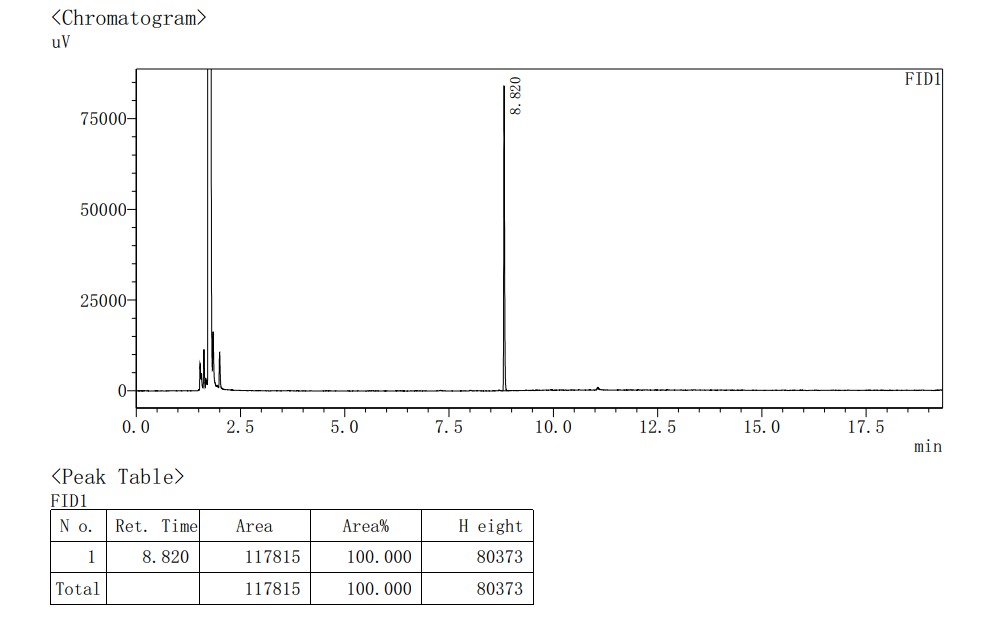
 Supplementary Figure 31. GC spectra for **3d**
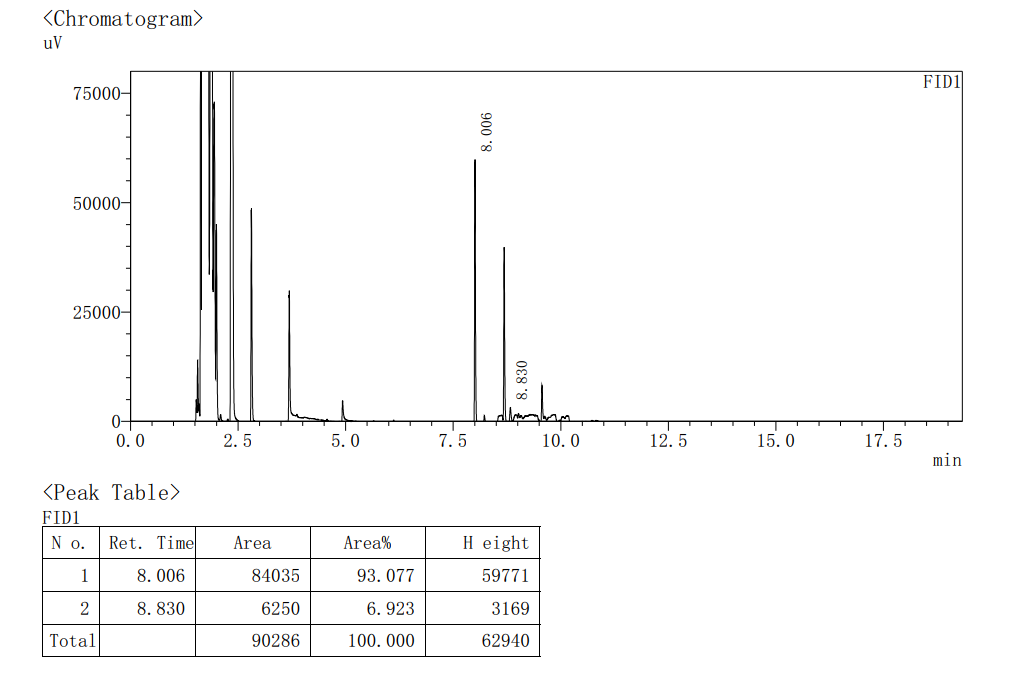
 Supplementary Figure 32. GC spectra for crude product of **2d**
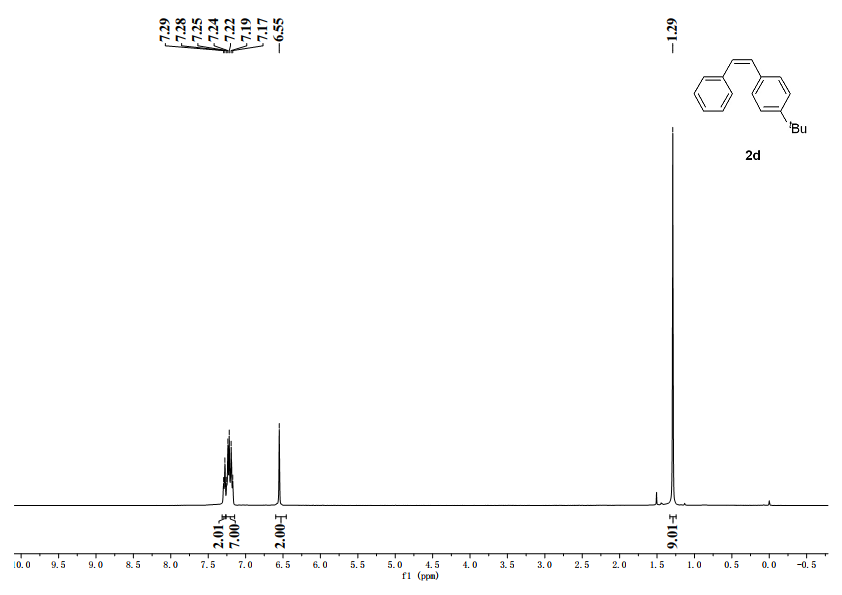
 Supplementary Figure 33. ^1^H NMR (400 MHz, CDCl_3_) spectra for compound **2d**
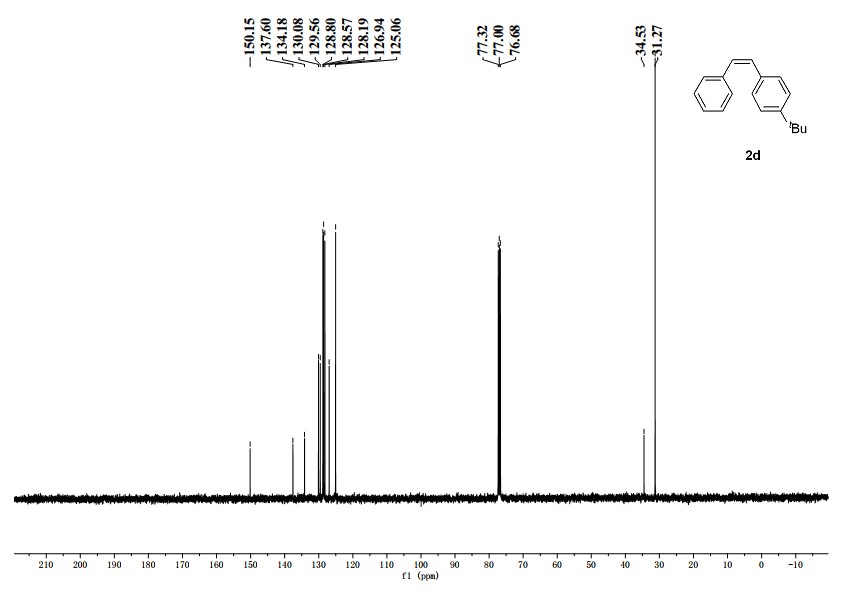
 Supplementary Figure 34. ^13^C NMR (101 MHz, CDCl_3_) spectra for compound **2d**


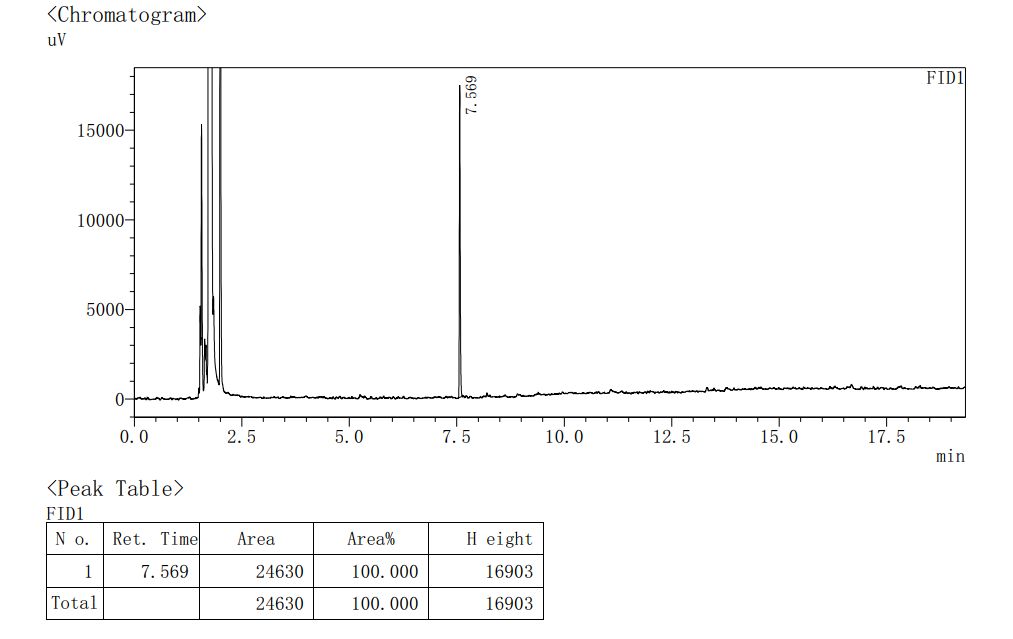
 Supplementary Figure 35. GC spectra for **2e**


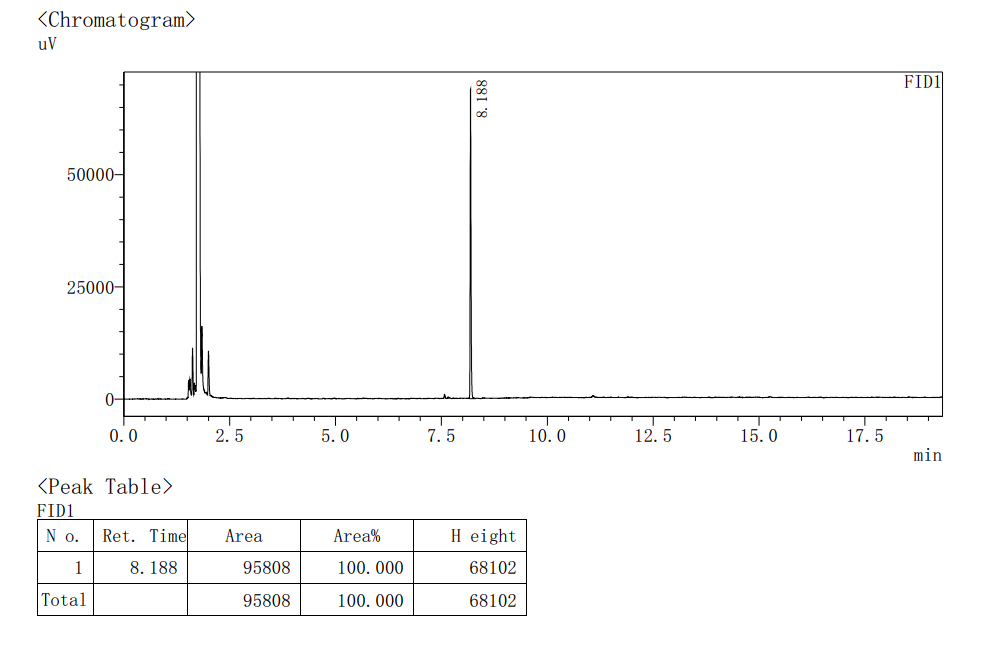
 Supplementary Figure 36. GC spectra for **3e**


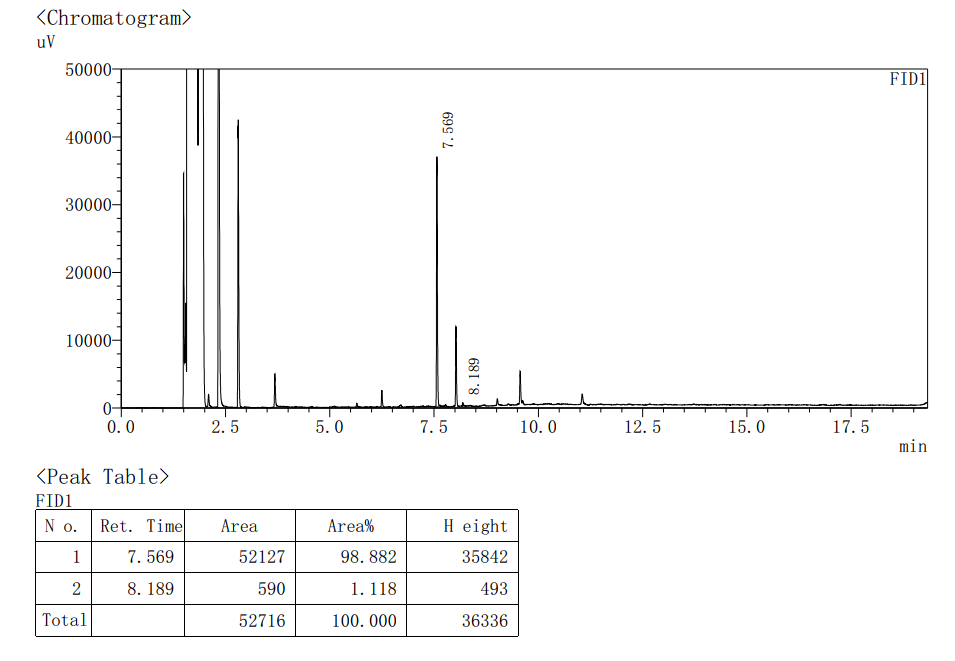
 Supplementary Figure 37. GC spectra for crude product of **2e**


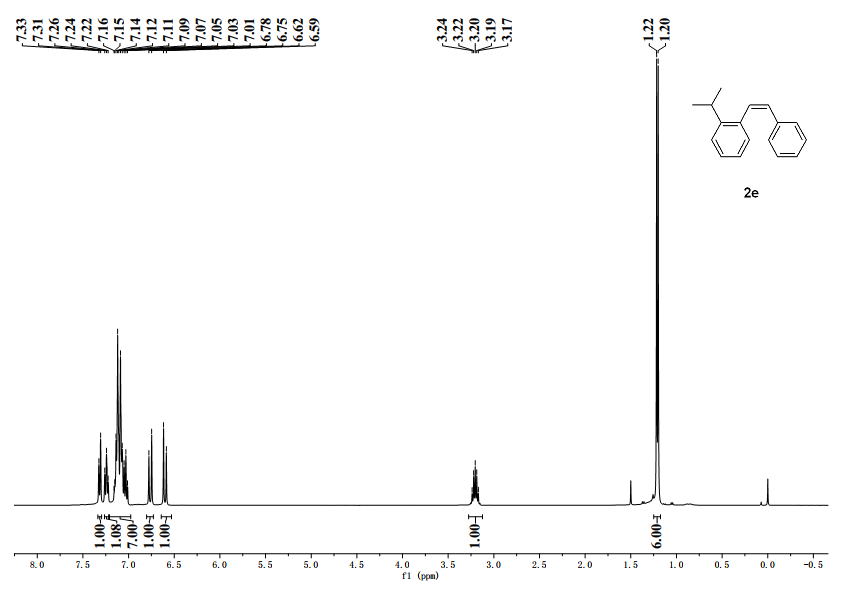
 Supplementary Figure 38. ^1^H NMR (400 MHz, CDCl_3_) spectra for compound **2e**
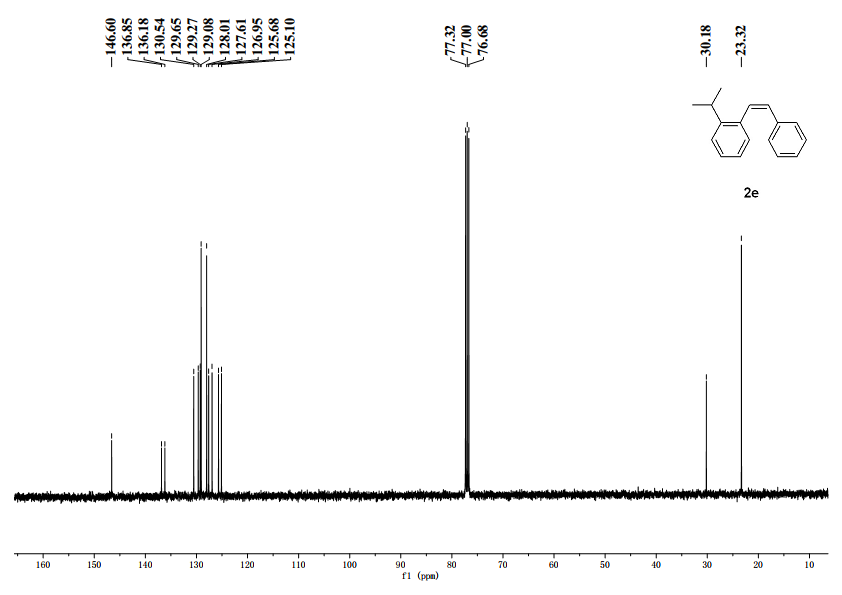
 Supplementary Figure 39. ^13^C NMR (101 MHz, CDCl_3_) spectra for compound **2e**


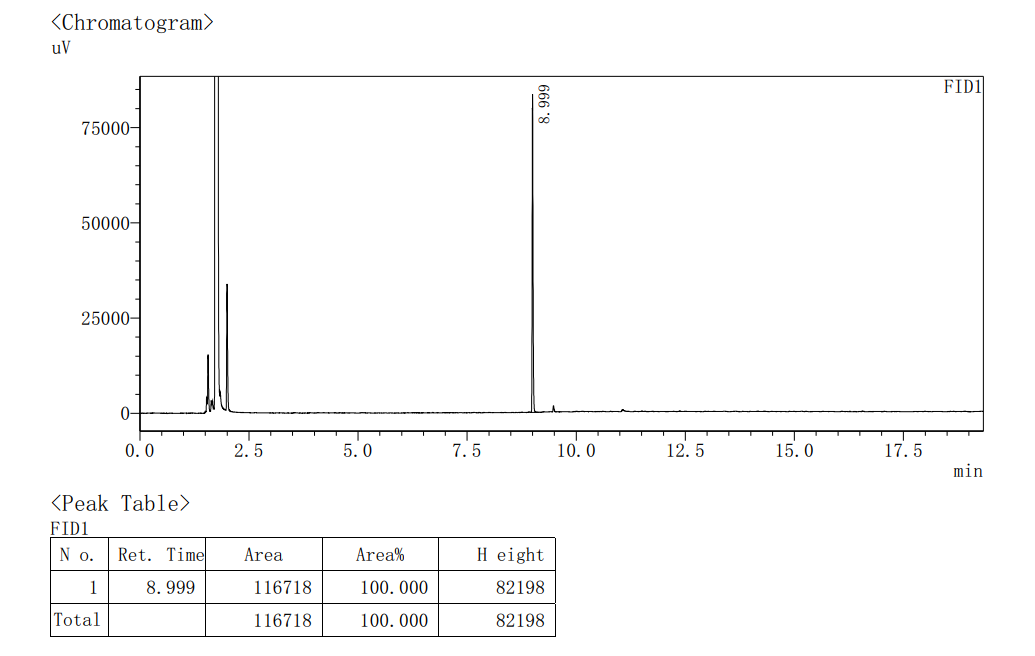
 Supplementary Figure 40. GC spectra for **2f**


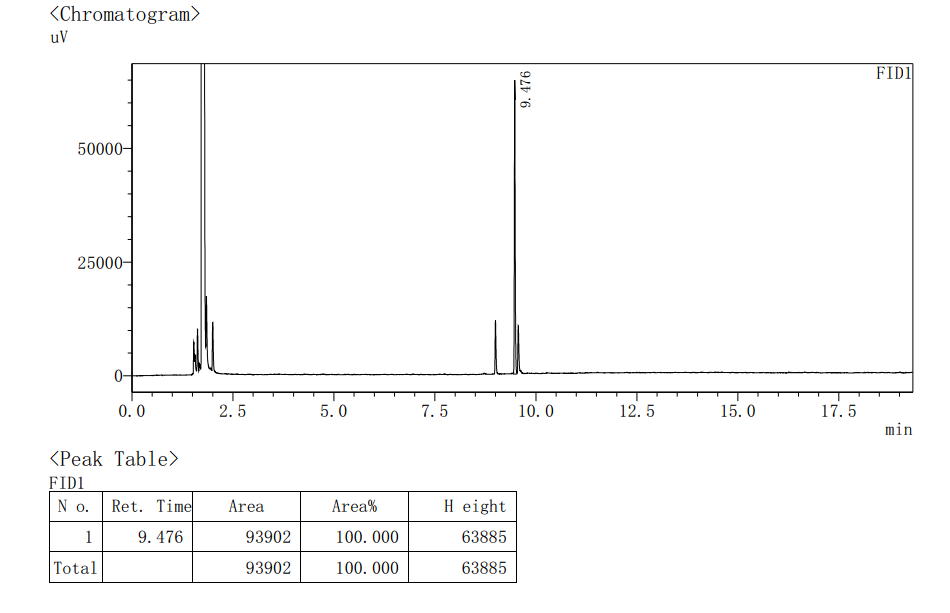
 Supplementary Figure 41. GC spectra for *E*-isomer of **2f**


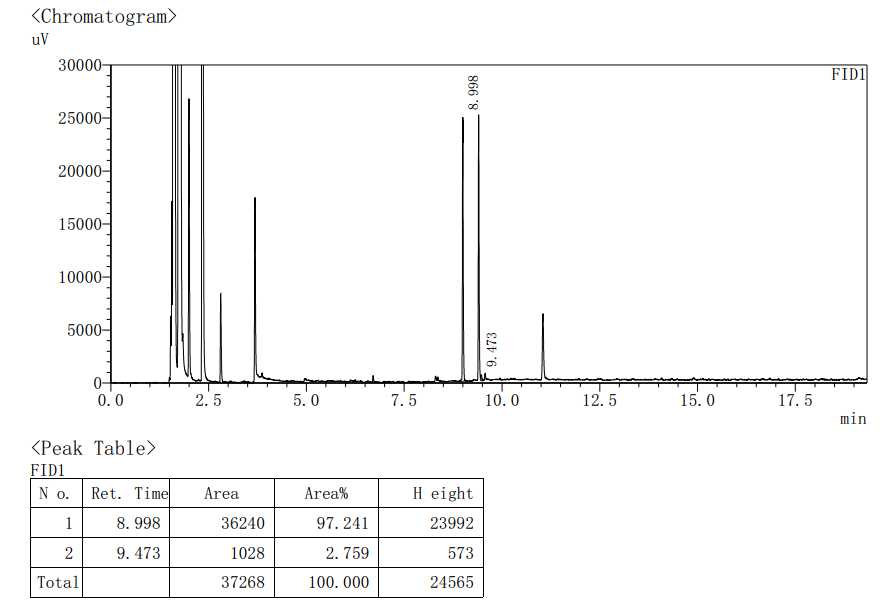
 Supplementary Figure 42. GC spectra for crude product of **2f**


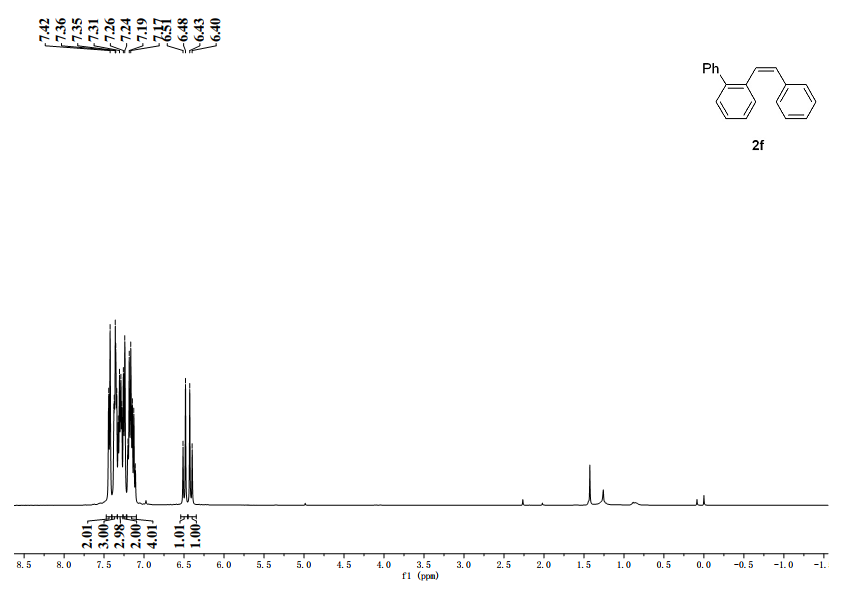
 Supplementary Figure 43. ^1^H NMR (400 MHz, CDCl_3_) spectra for compound **2f**
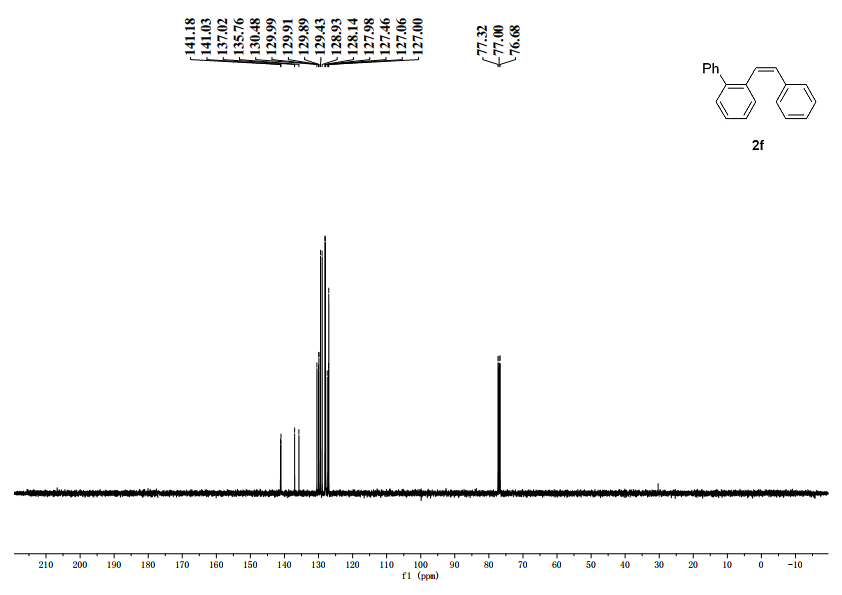
 Supplementary Figure 44. ^13^C NMR (101 MHz, CDCl_3_) spectra for compound **2f**


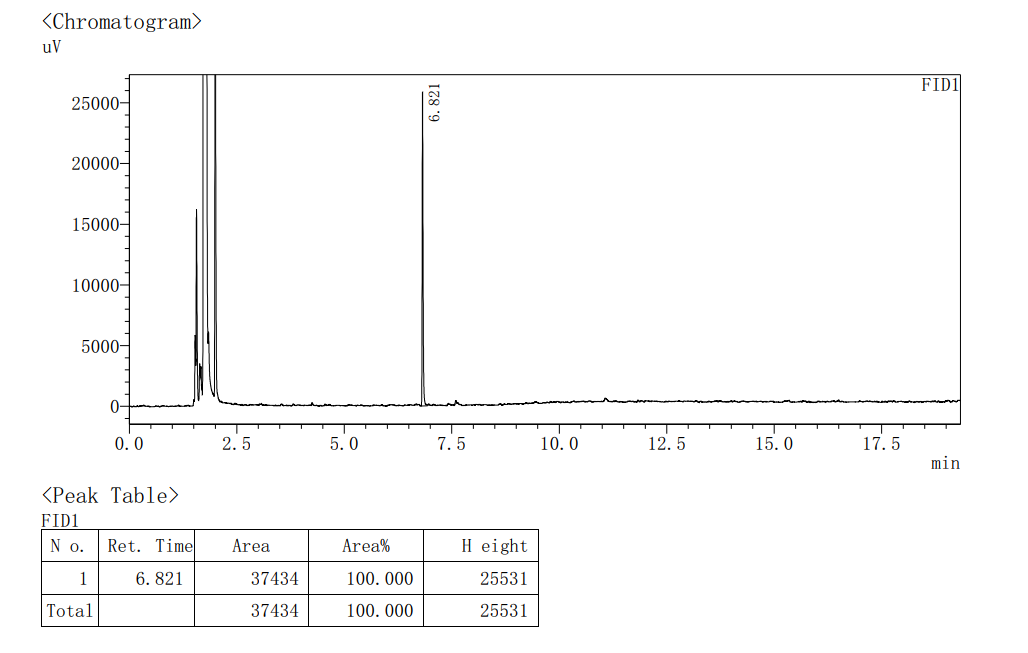
 Supplementary Figure 45. GC spectra for **2g**
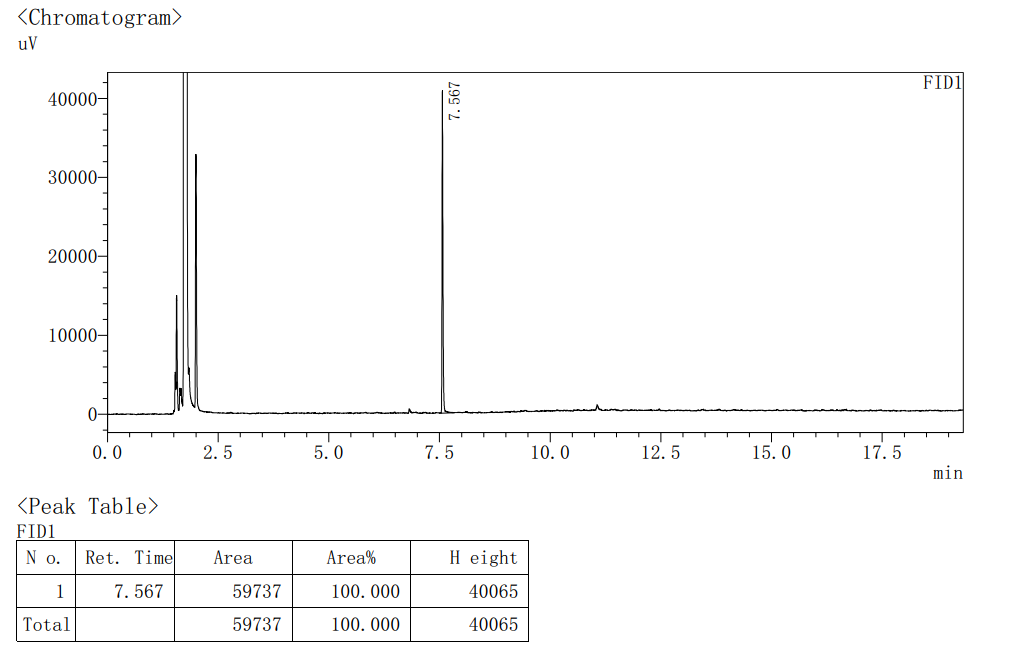
 Supplementary Figure 46. GC spectra for **3g**


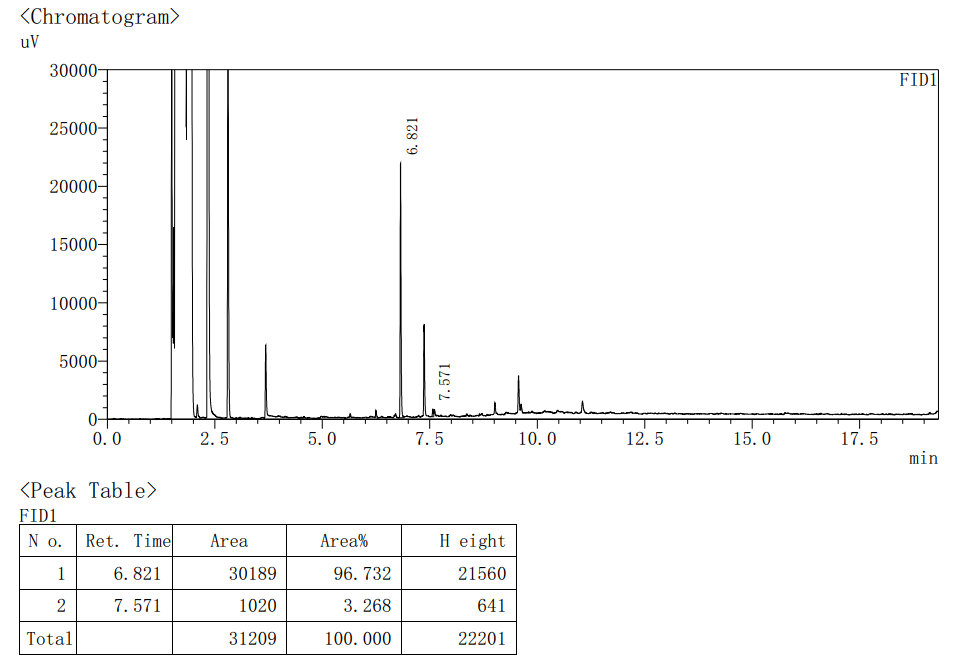


Supplementary Figure 47. GC spectra for crude product of **2g**
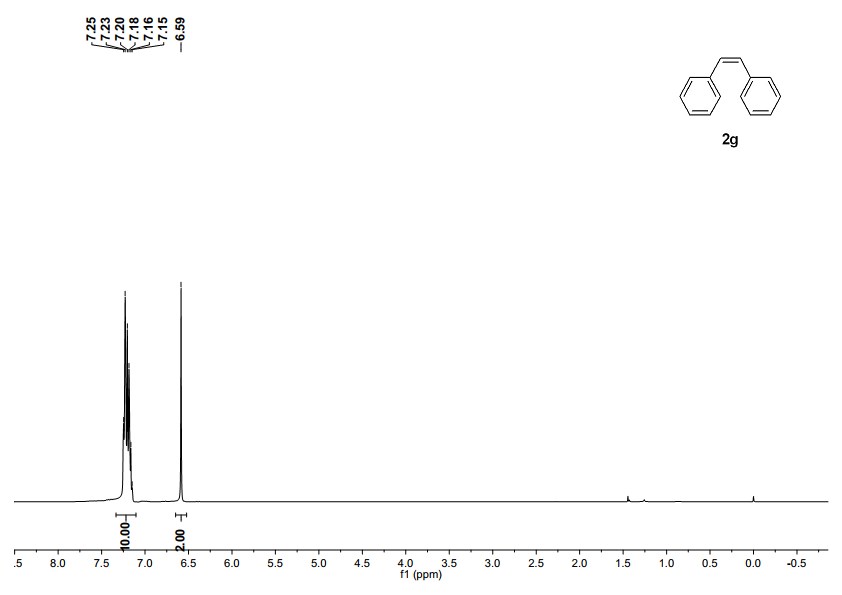
 Supplementary Figure 48. ^1^H NMR (400 MHz, CDCl_3_) spectra for compound **2g**
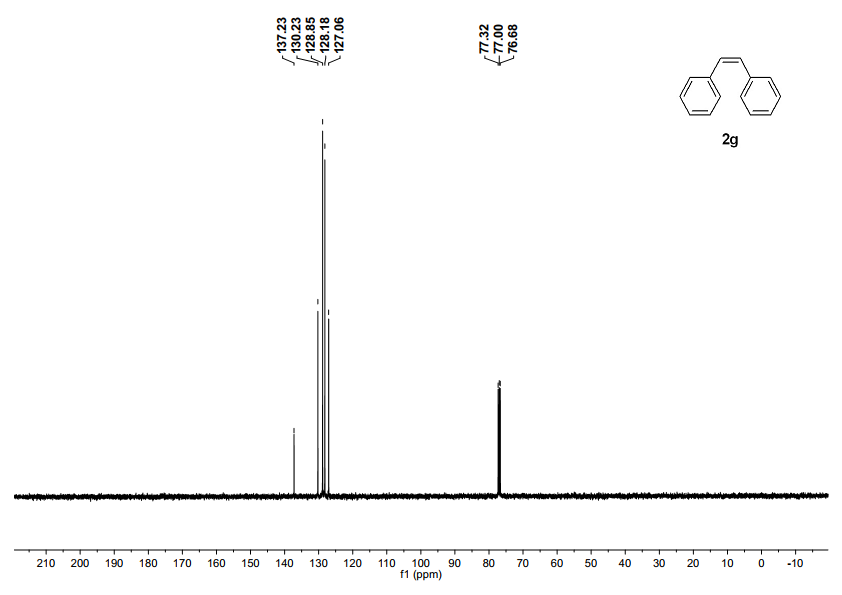
 Supplementary Figure 49. ^13^C NMR (101 MHz, CDCl_3_) spectra for compound **2g**


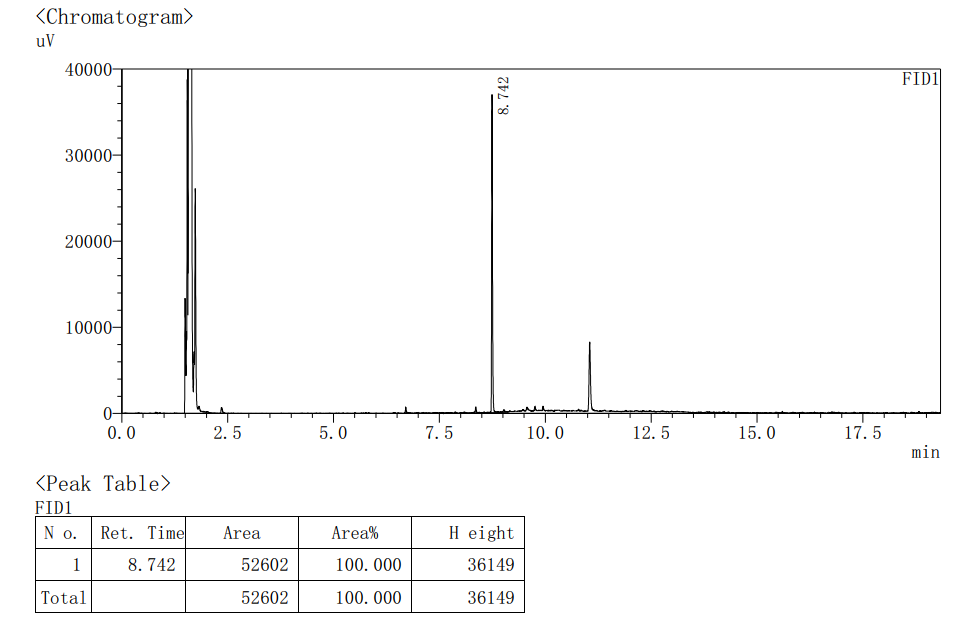
 Supplementary Figure 50. GC spectra for **2h**


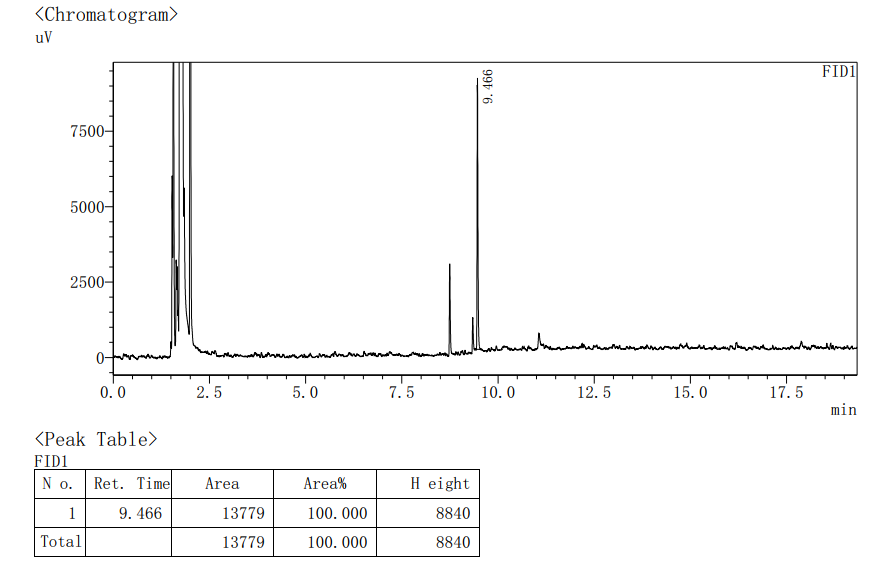
 Supplementary Figure 51. GC spectra for *E*- isomer of **2h**


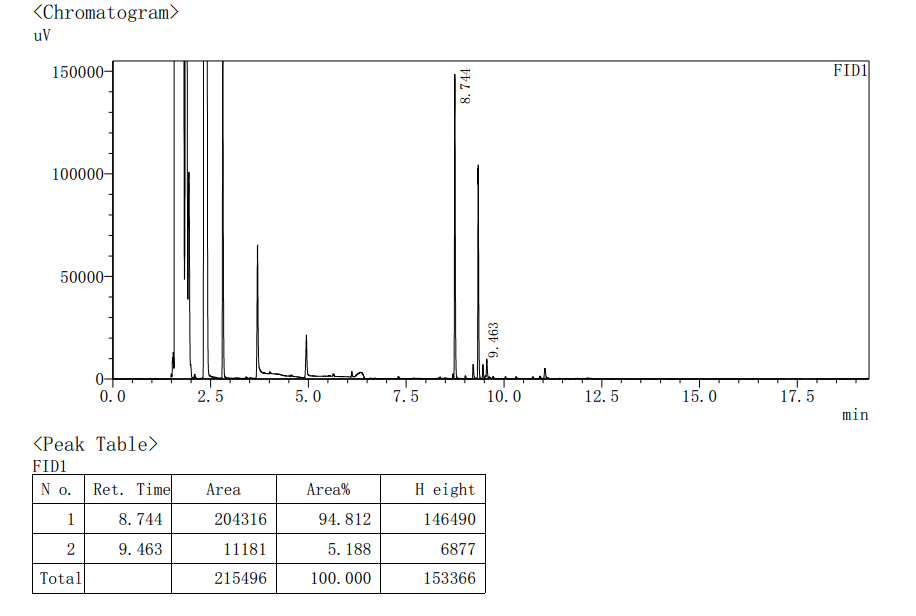
 Supplementary Figure 52. GC spectra for crude product of **2h**


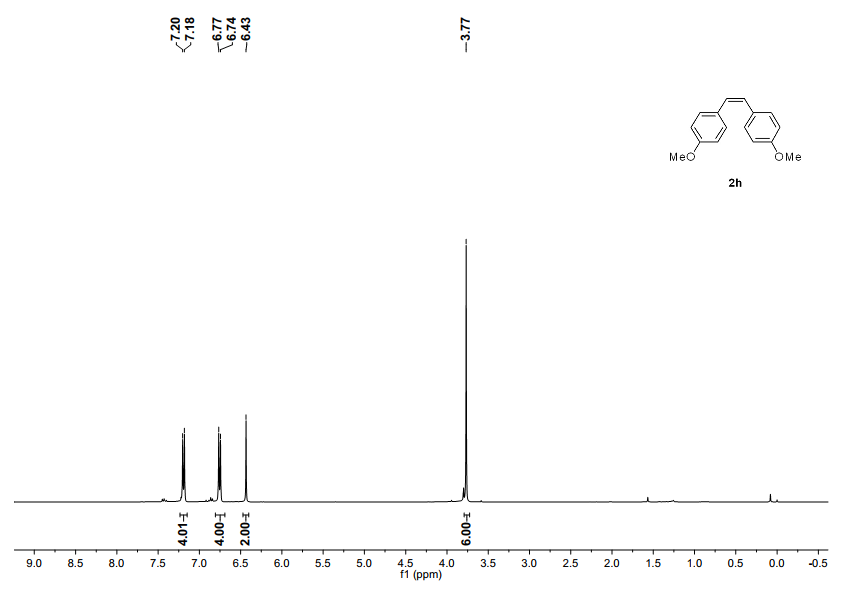
 Supplementary Figure 53. ^1^H NMR (400 MHz, CDCl_3_) spectra for compound **2h**
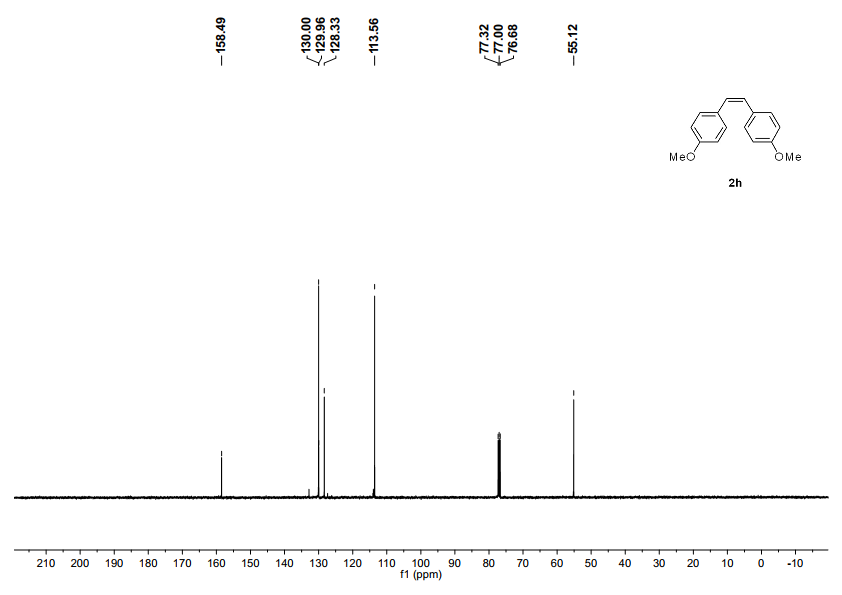
 Supplementary Figure 54. ^13^C NMR (101 MHz, CDCl_3_) spectra for compound **2h**


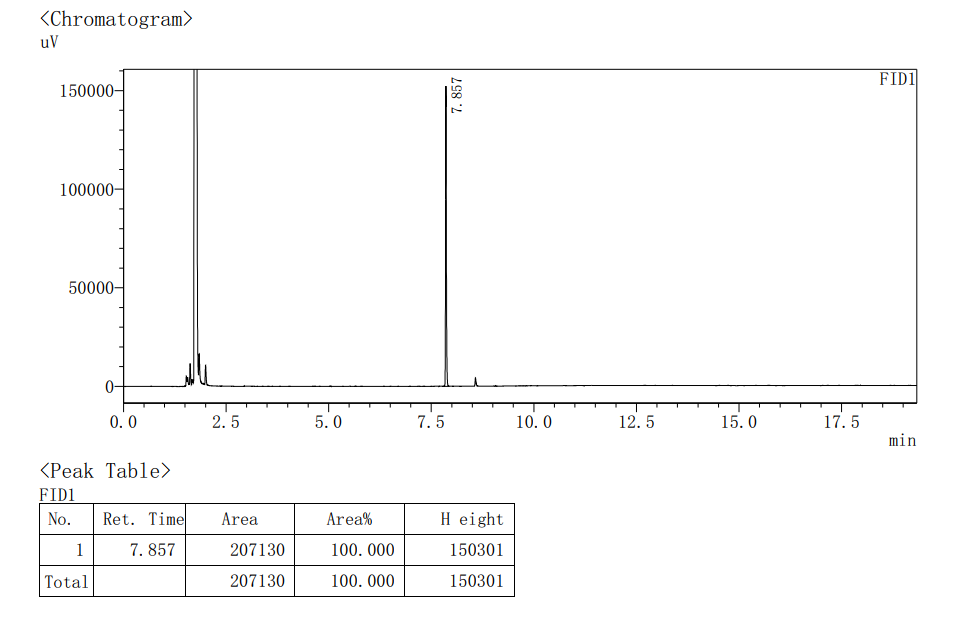
 Supplementary Figure 55. GC spectra for **2i**


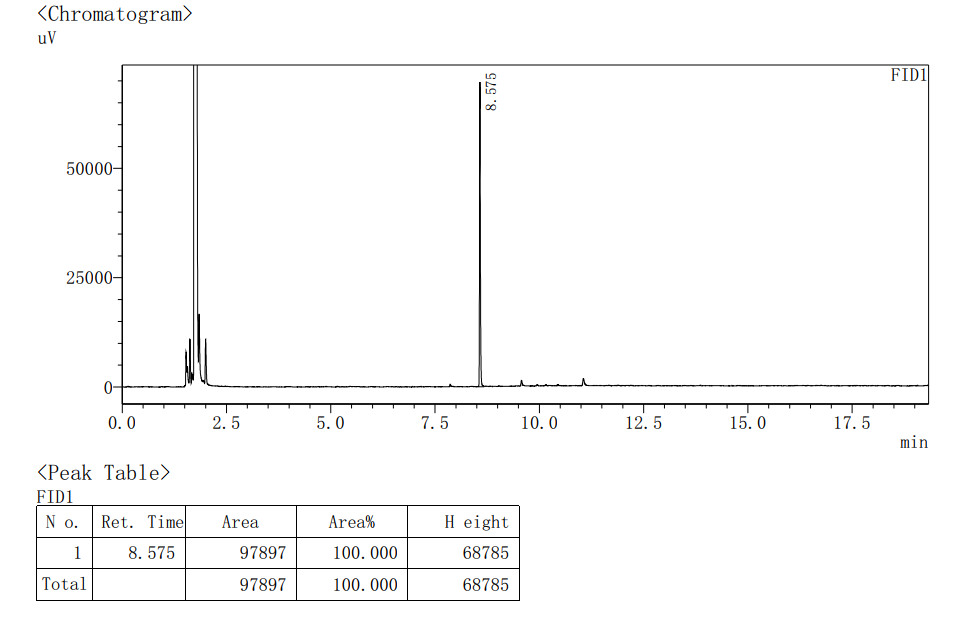
 Supplementary Figure 56. GC spectra for **3i**


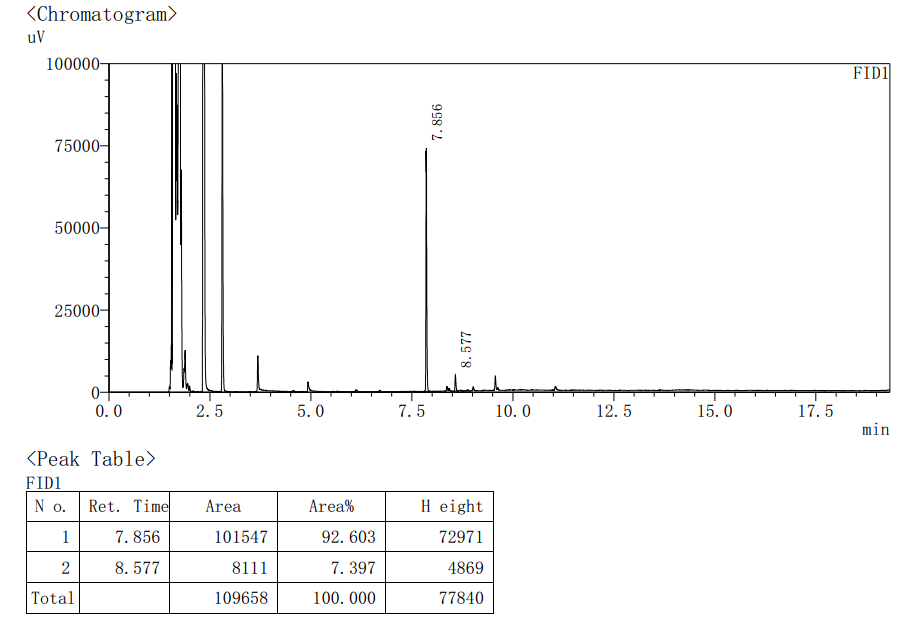


Supplementary Figure 57. GC spectra for crude product of **2i**
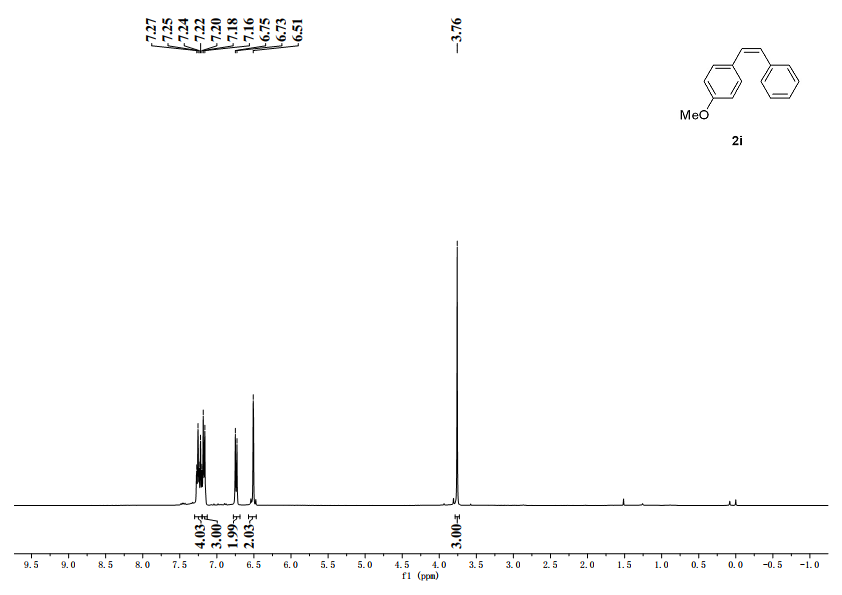
 Supplementary Figure 58. ^1^H NMR (400 MHz, CDCl_3_) spectra for compound **2i**
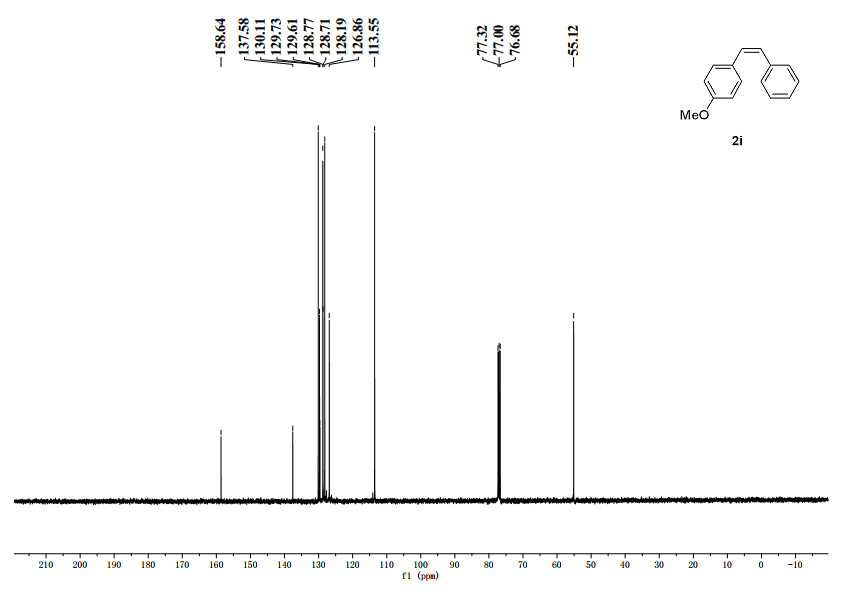
 Supplementary Figure 59. ^13^C NMR (101 MHz, CDCl_3_) spectra for compound **2i**


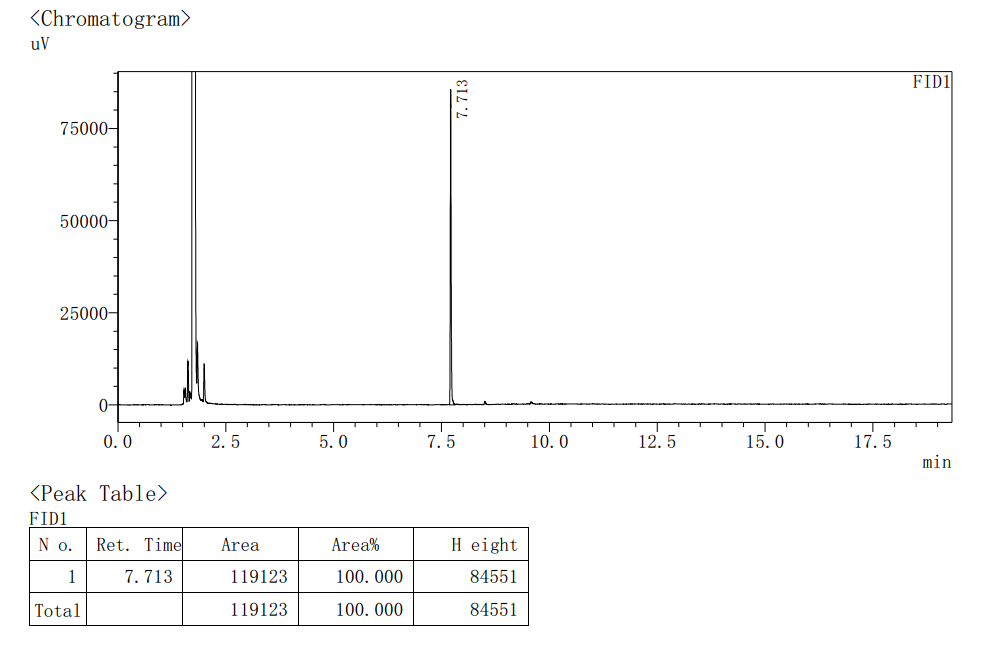
 Supplementary Figure 60. GC spectra for **2j**
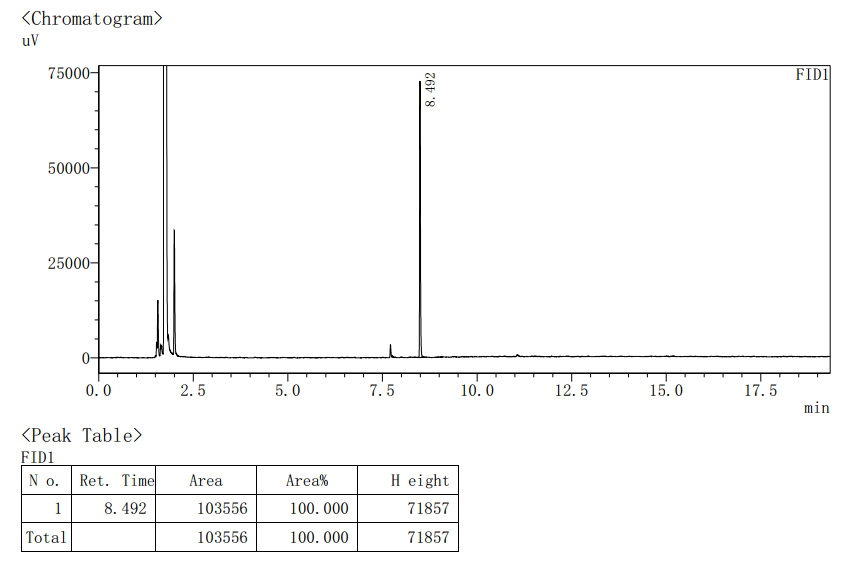
 Supplementary Figure 61. GC spectra for **3j**
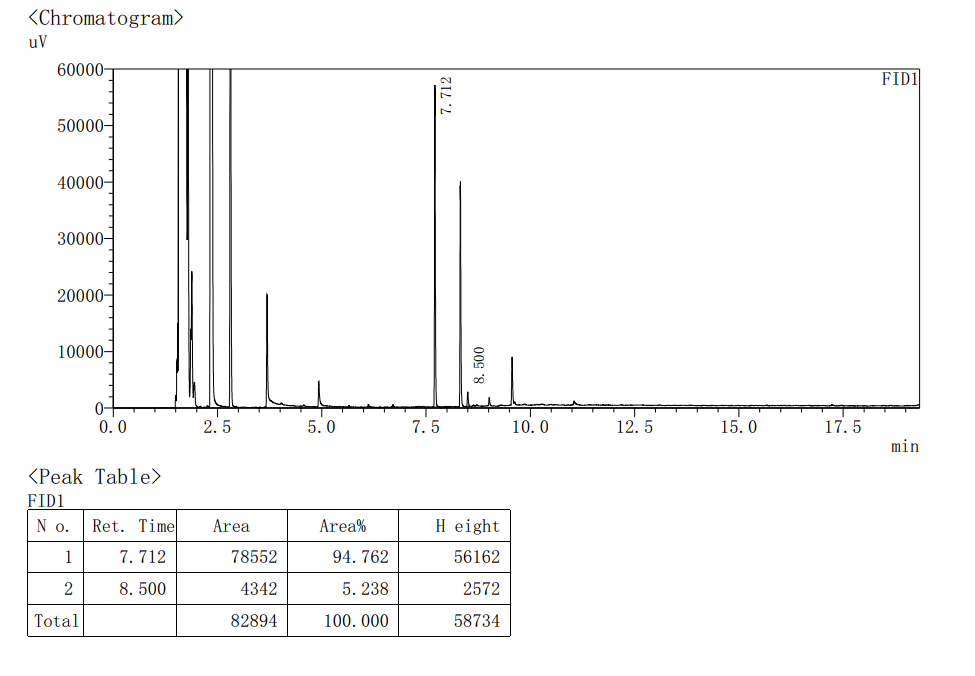
 Supplementary Figure 62. GC spectra for crude product of **2j**


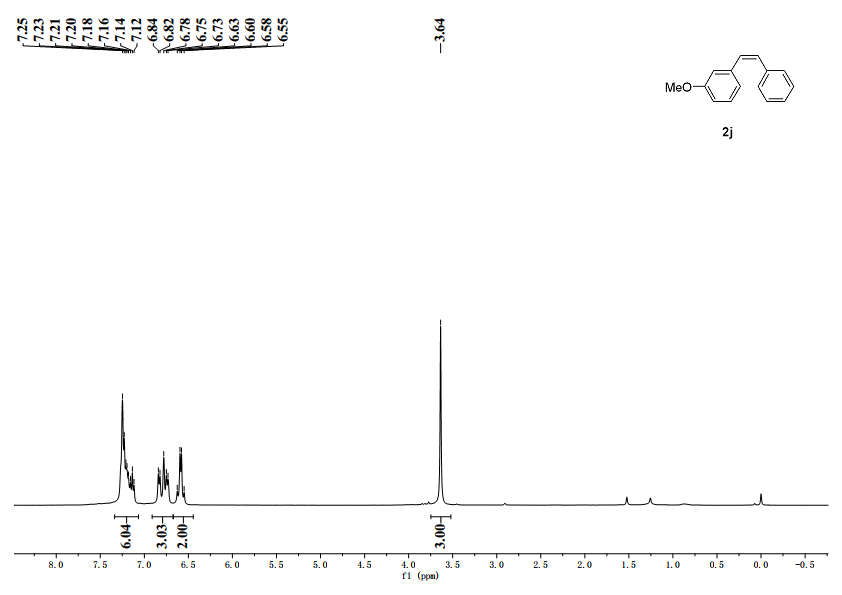
 Supplementary Figure 63. ^1^H NMR (400 MHz, CDCl_3_) spectra for compound **2j**
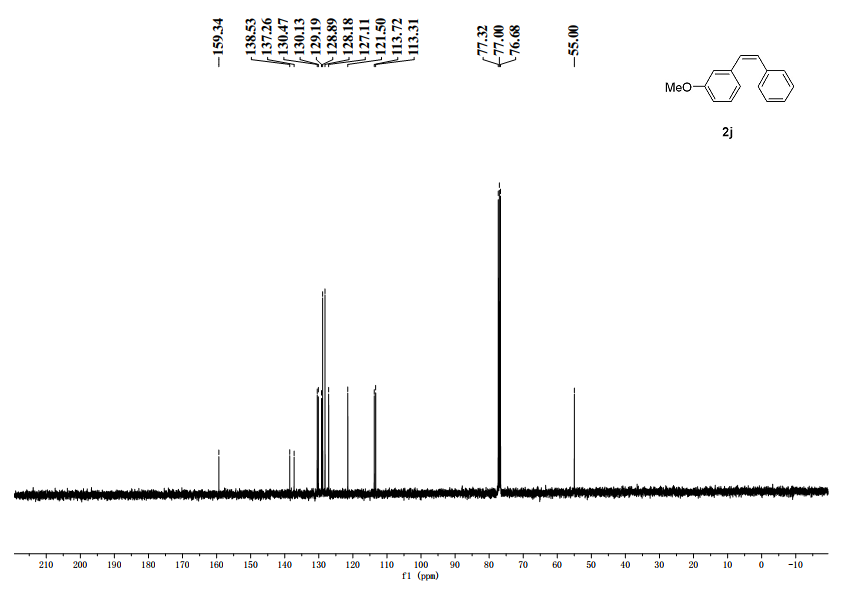
 Supplementary Figure 64. ^13^C NMR (101 MHz, CDCl_3_) spectra for compound **2j**


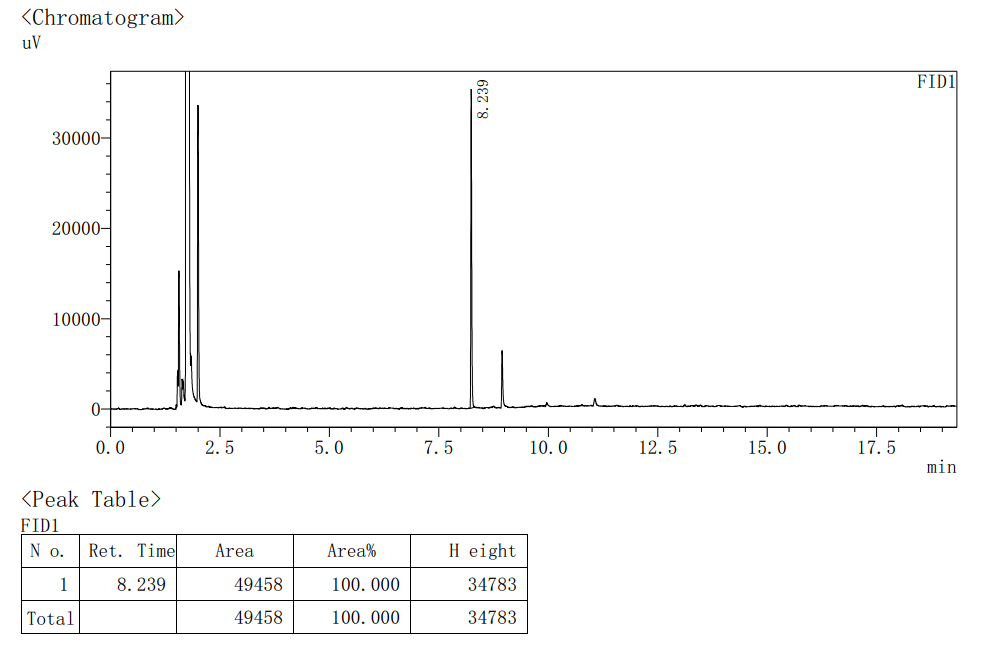
 Supplementary Figure 65. GC spectra for **2k**
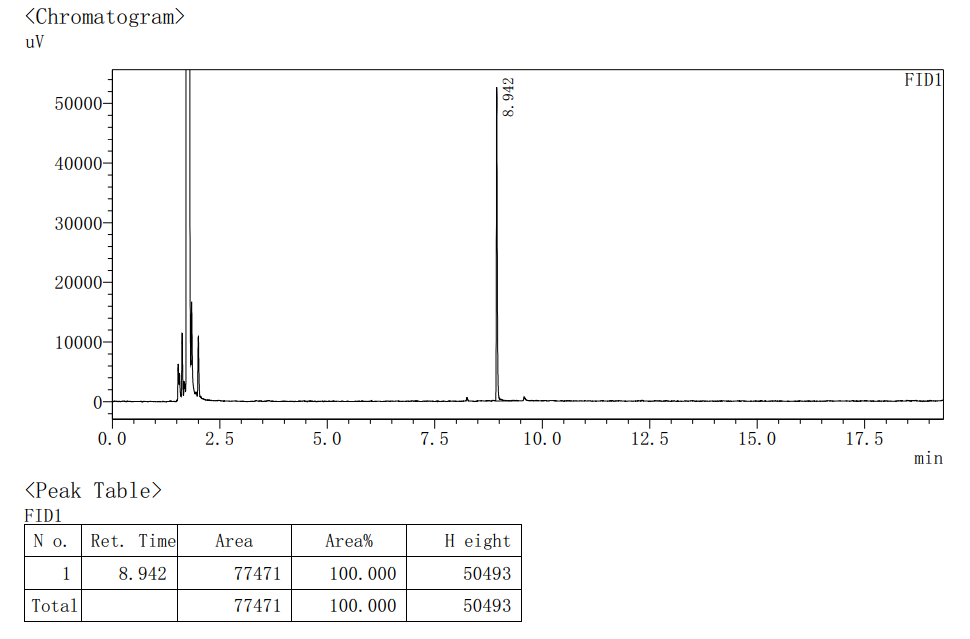
 Supplementary Figure 66. GC spectra for **3k**


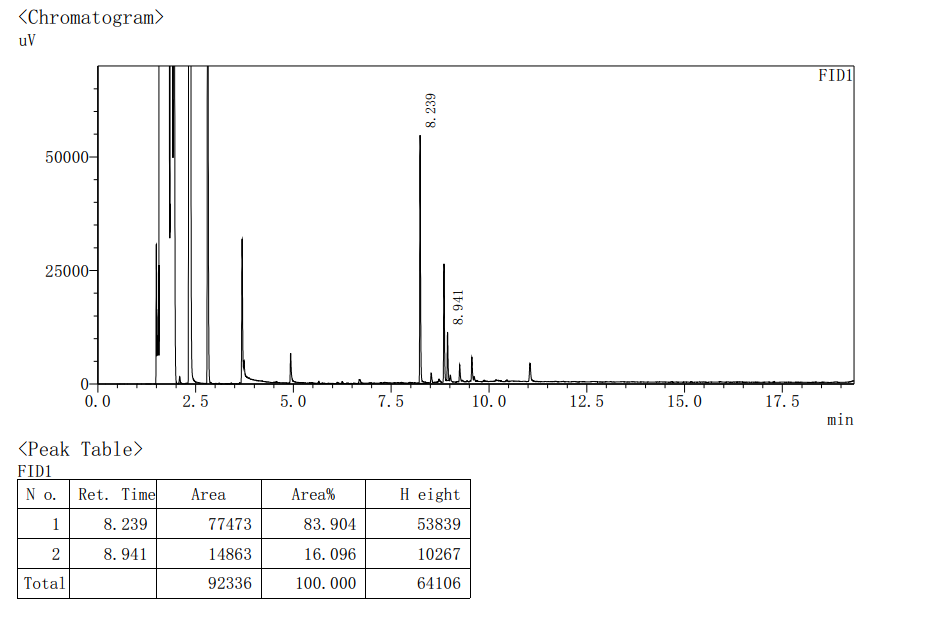
 Supplementary Figure 67. GC spectra for crude product of **2k**


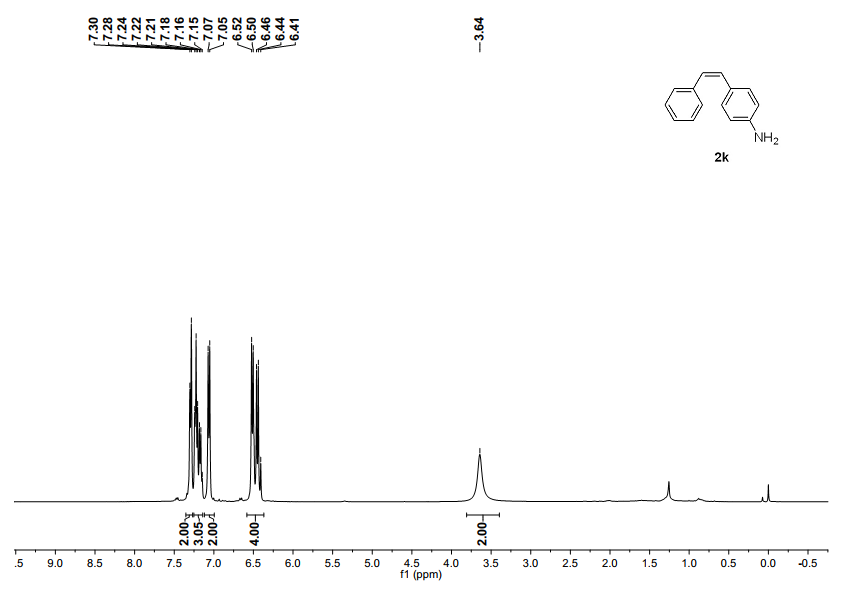
 Supplementary Figure 68. ^1^H NMR (400 MHz, CDCl_3_) spectra for compound **2k**
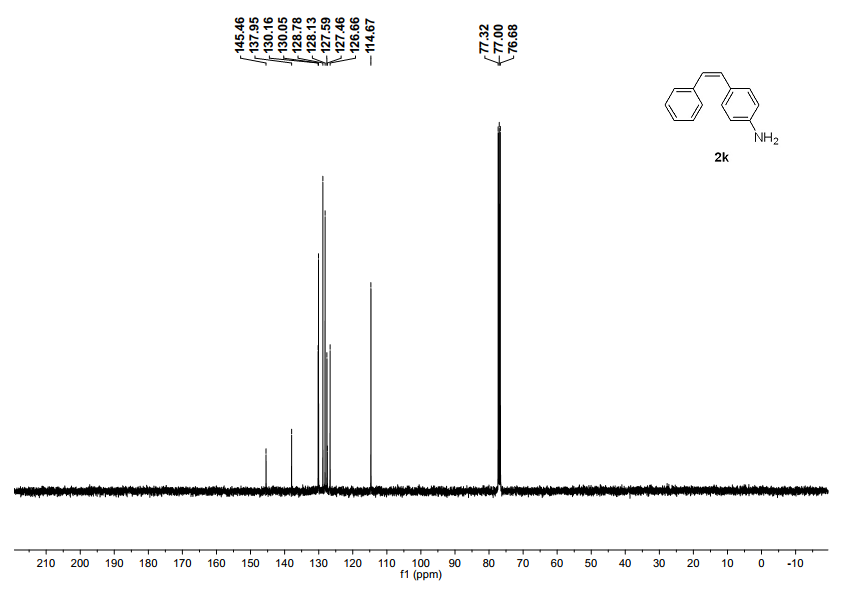
 Supplementary Figure 69. ^13^C NMR (101 MHz, CDCl_3_) spectra for compound **2k**


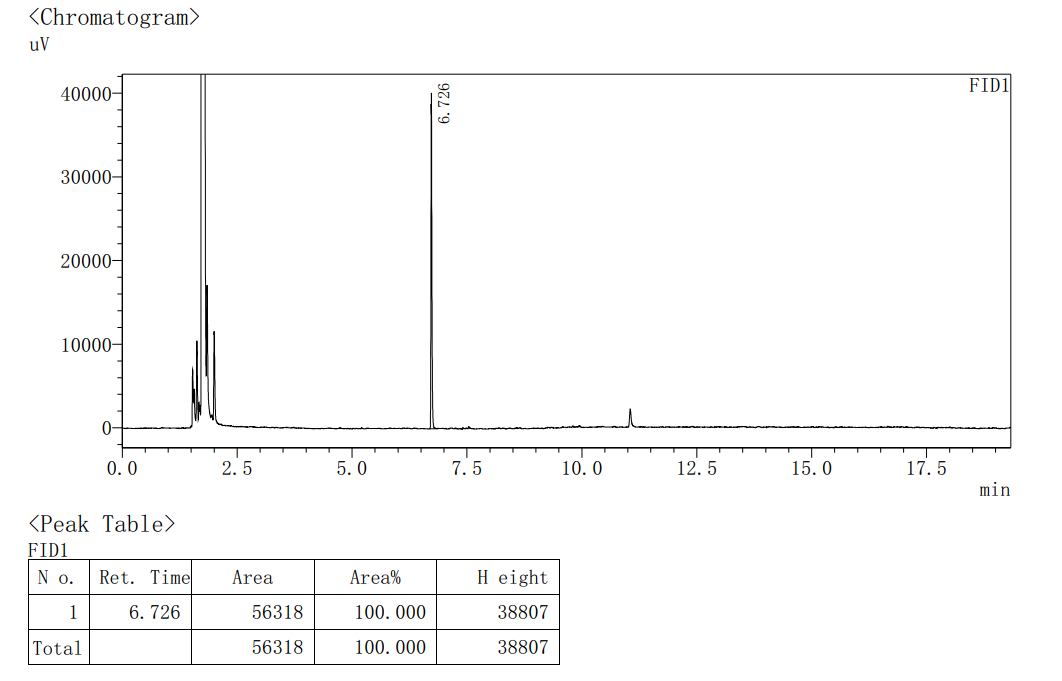
 Supplementary Figure 70. GC spectra for **2l**


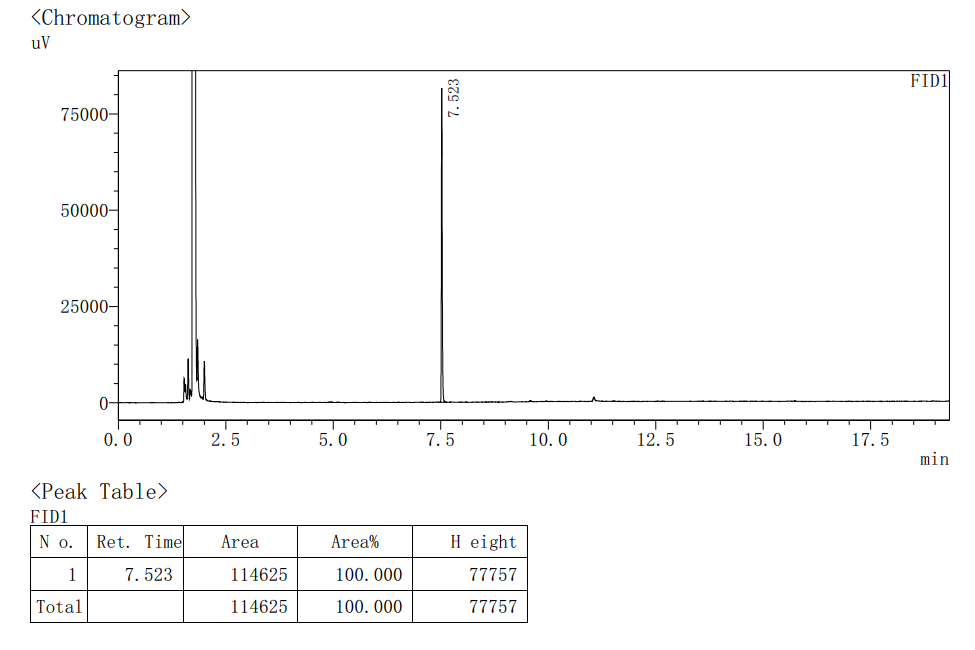
 Supplementary Figure 71. GC spectra for **3l**


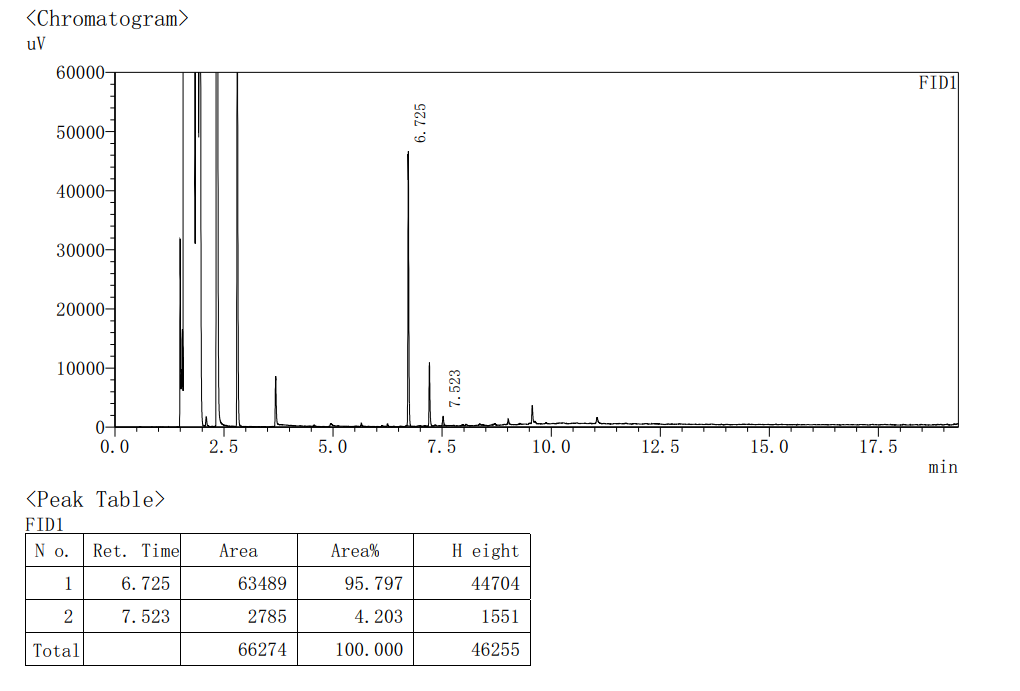
 Supplementary Figure 72. GC spectra for crude product of **2l**


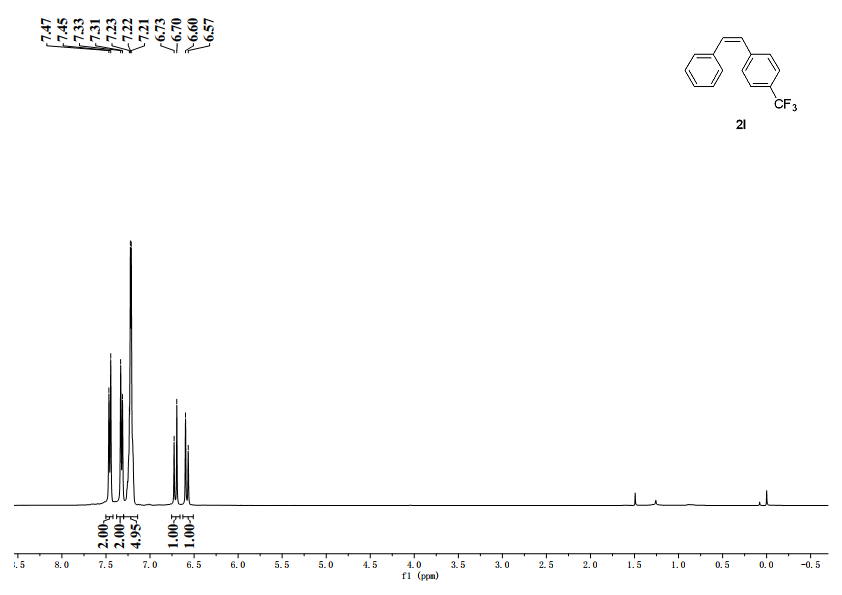
 Supplementary Figure 73. ^1^H NMR (400 MHz, CDCl_3_) spectra for compound **2l**
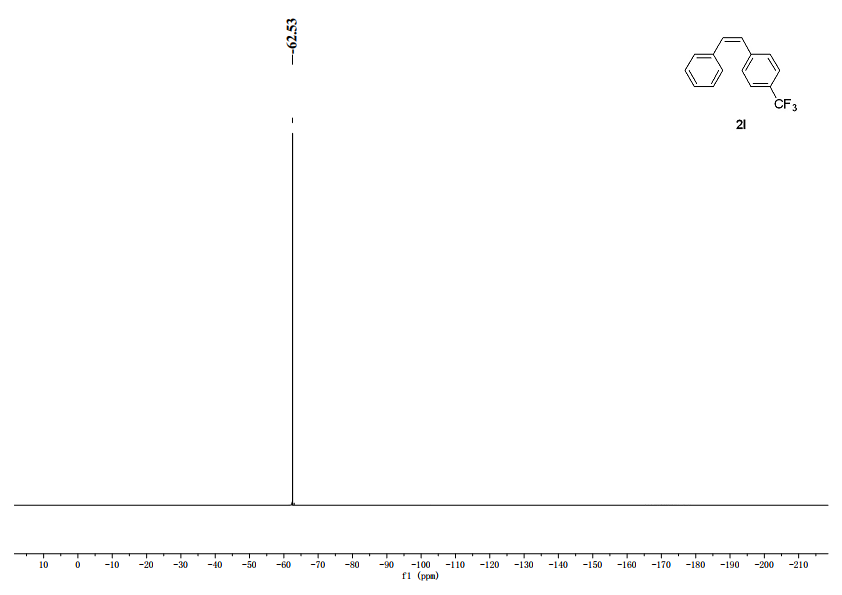
 Supplementary Figure 74. ^19^F NMR (376 MHz, CDCl_3_) spectra for compound **2l**
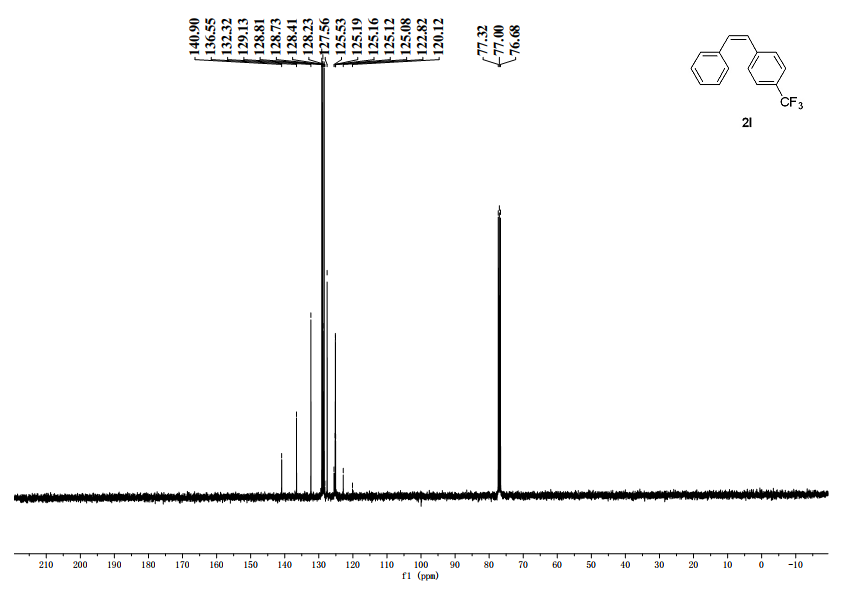
 Supplementary Figure 75. ^13^C NMR (101 MHz, CDCl_3_) spectra for compound **2l**


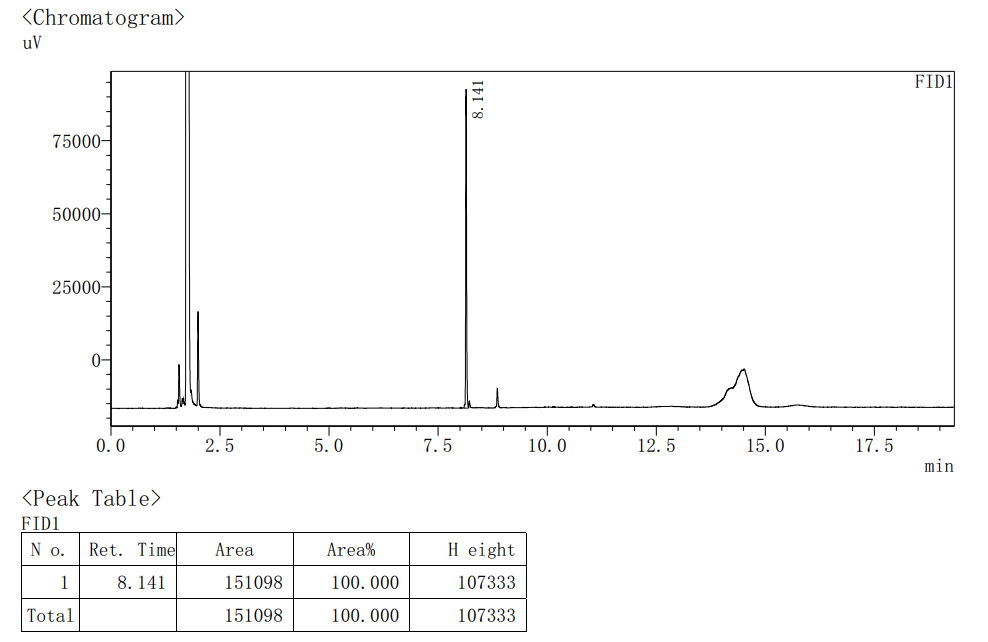
 Supplementary Figure 76. GC spectra for **2m**
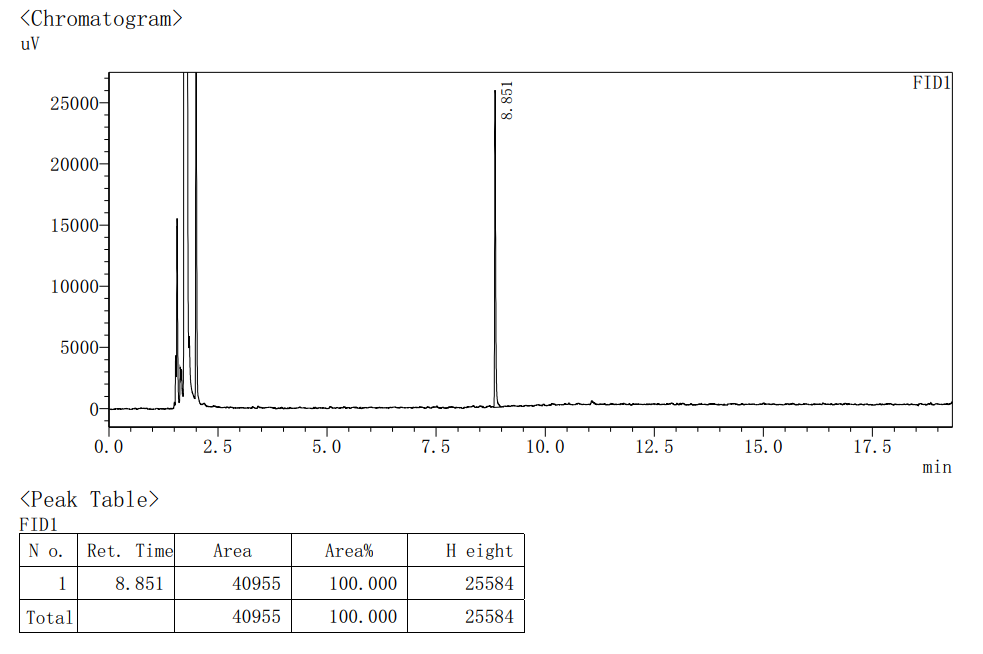
 Supplementary Figure 77. GC spectra for **3m**


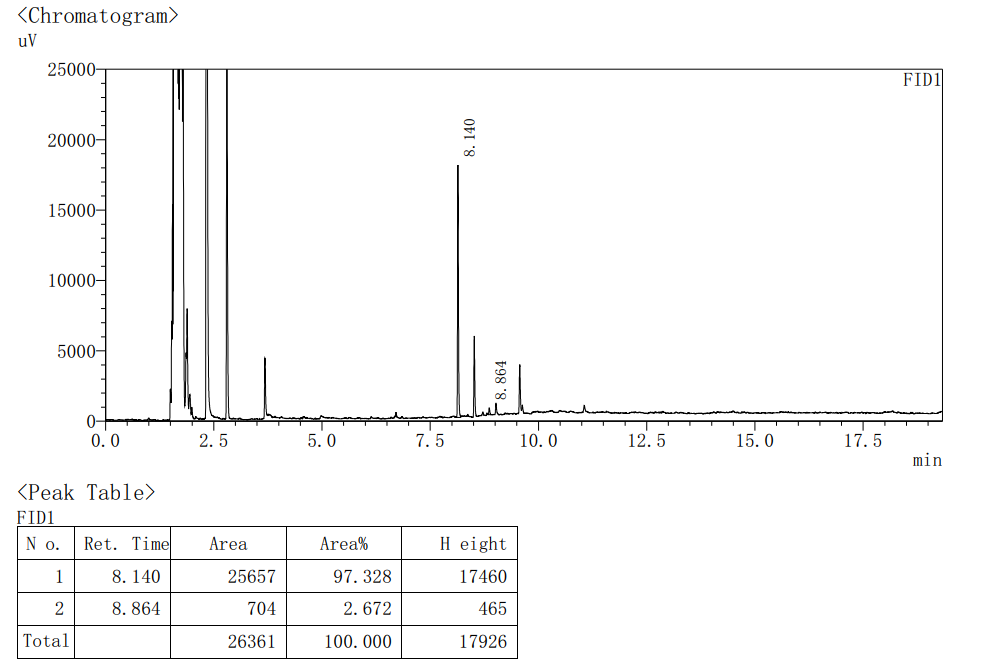
 Supplementary Figure 78. GC spectra for crude product of **2m**


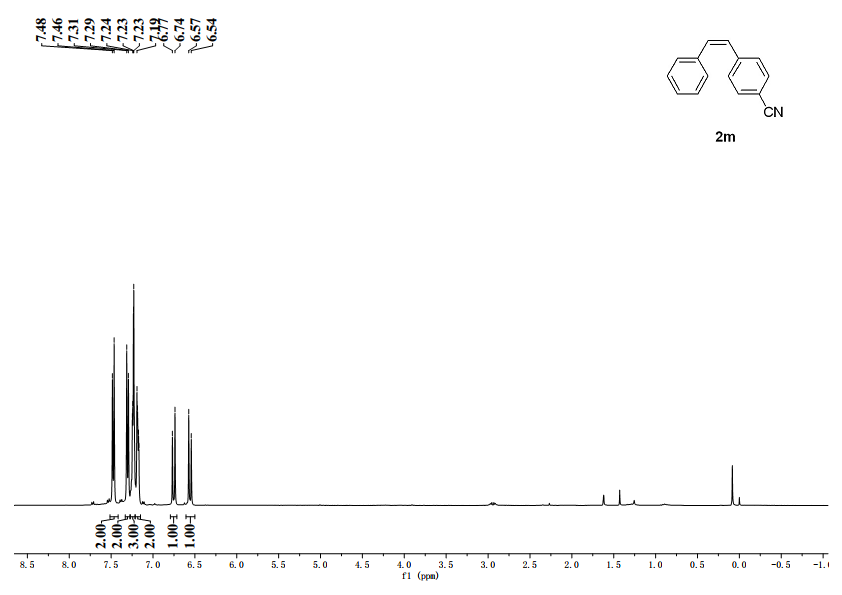
 Supplementary Figure 79. ^1^H NMR (400 MHz, CDCl_3_) spectra for compound **2m**
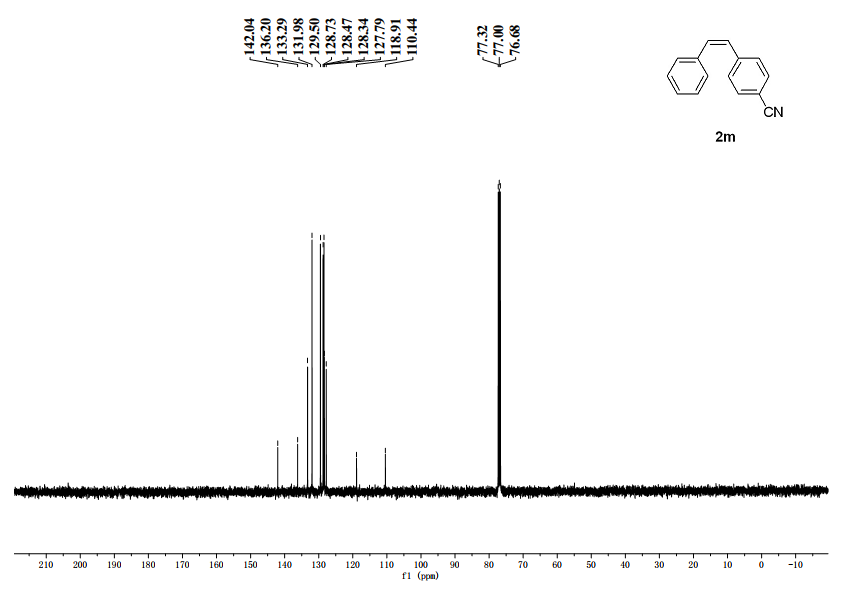
 Supplementary Figure 80. ^13^C NMR (101 MHz, CDCl_3_) spectra for compound **2m**


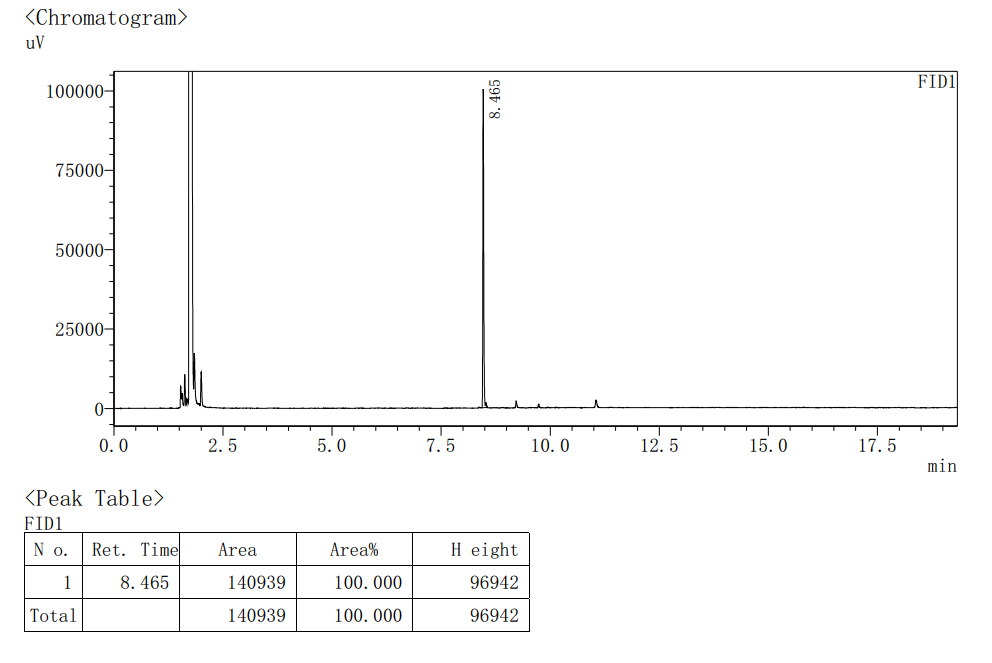
 Supplementary Figure 81. GC spectra for **2n**
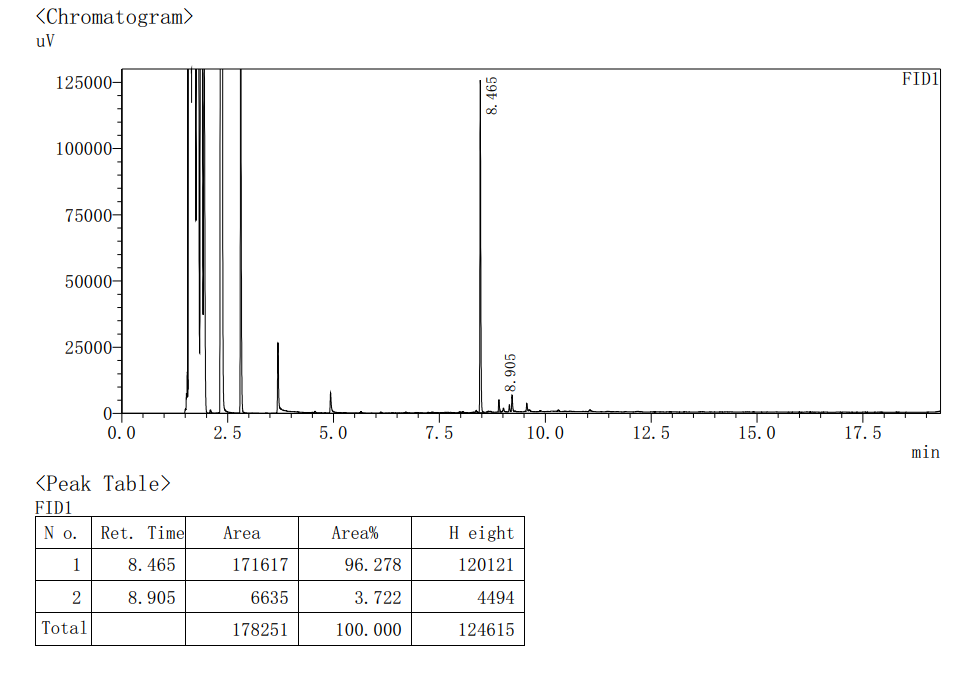


Supplementary Figure 82. GC spectra for crude product of **2n**
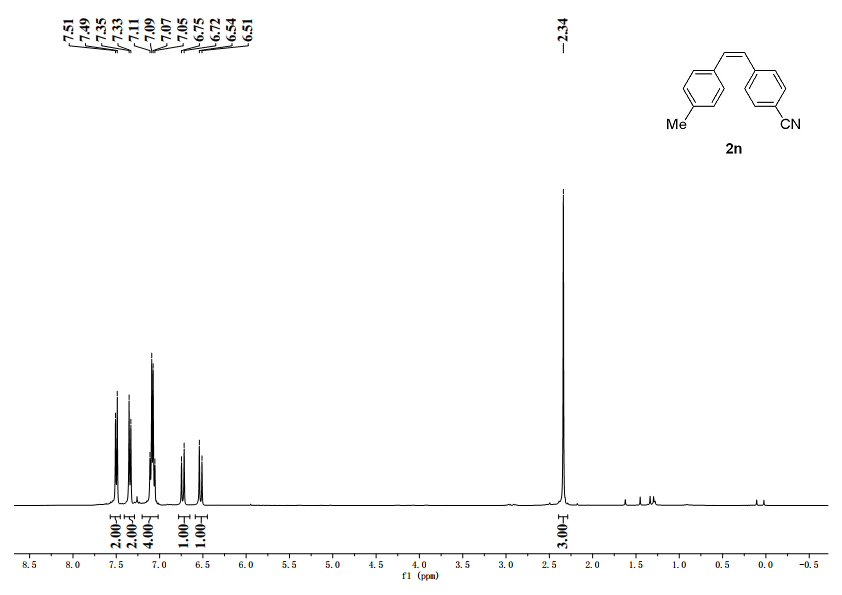
 Supplementary Figure 83. ^1^H NMR (400 MHz, CDCl_3_) spectra for compound **2n**
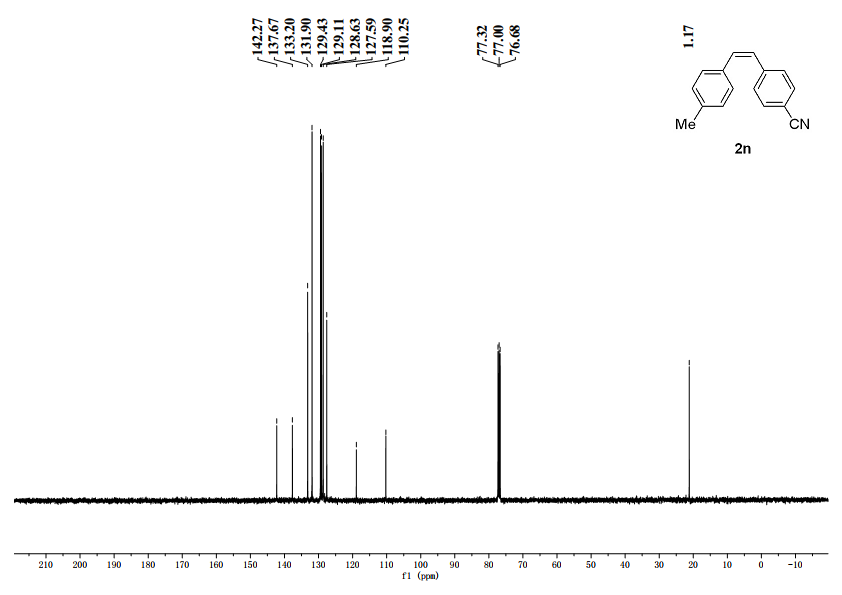
 Supplementary Figure 84. ^13^C NMR (101 MHz, CDCl_3_) spectra for compound **2n**


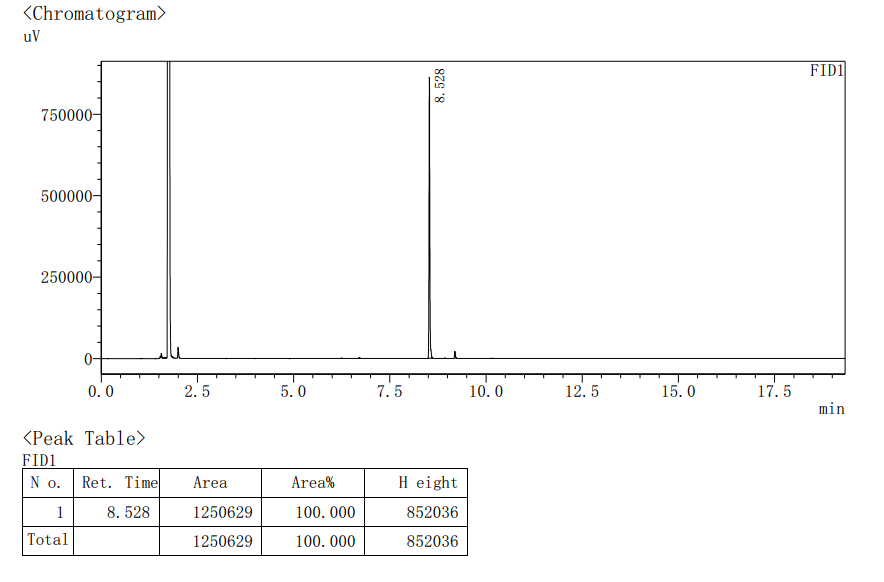
 Supplementary Figure 85. GC spectra for **2o**
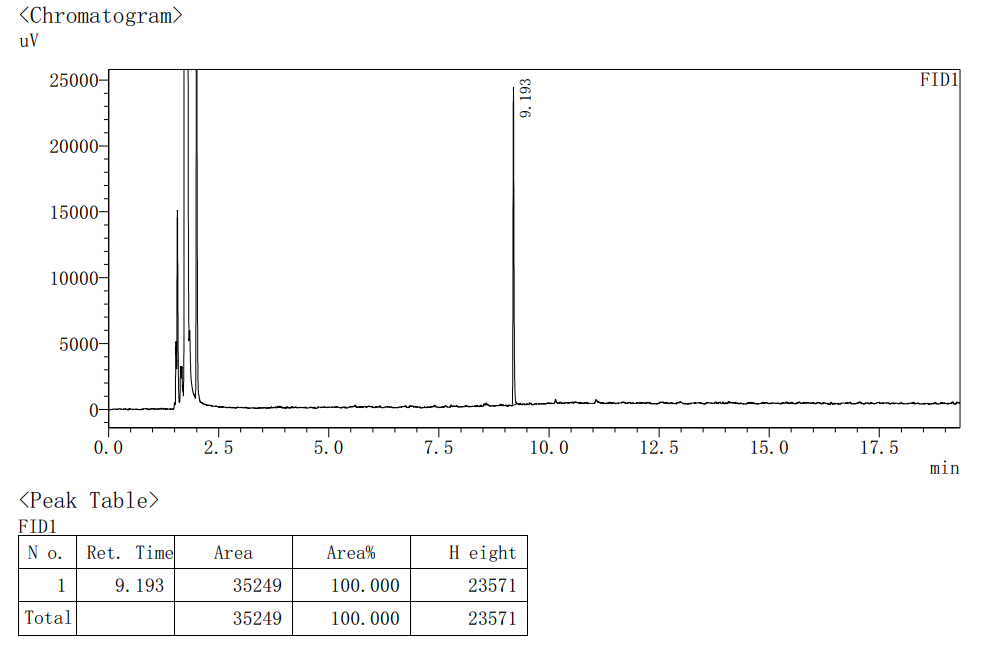
 Supplementary Figure 86. GC spectra for **3o**


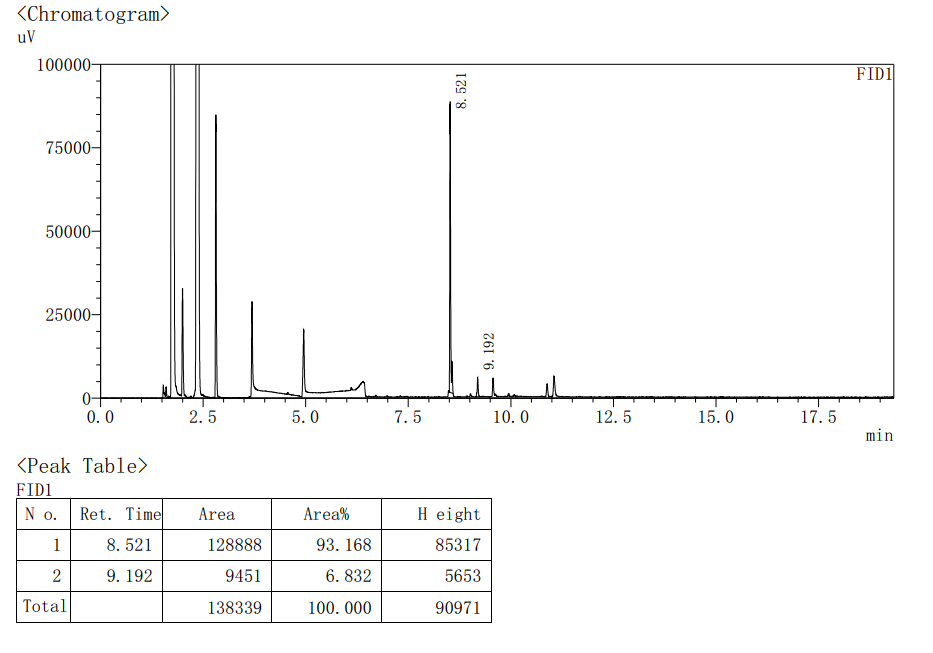
 Supplementary Figure 87. GC spectra for crude product of **2o**


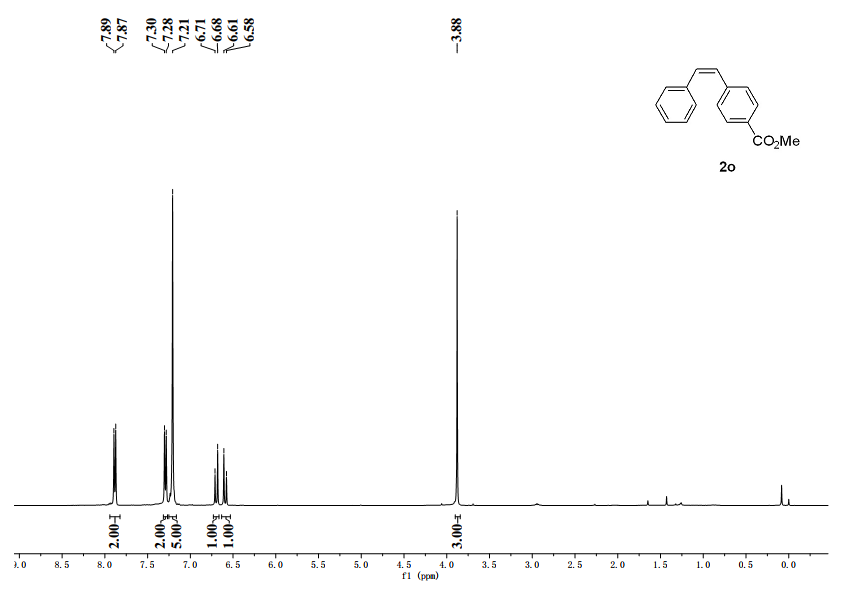
 Supplementary Figure 88. ^1^H NMR (400 MHz, CDCl_3_) spectra for compound **2o**
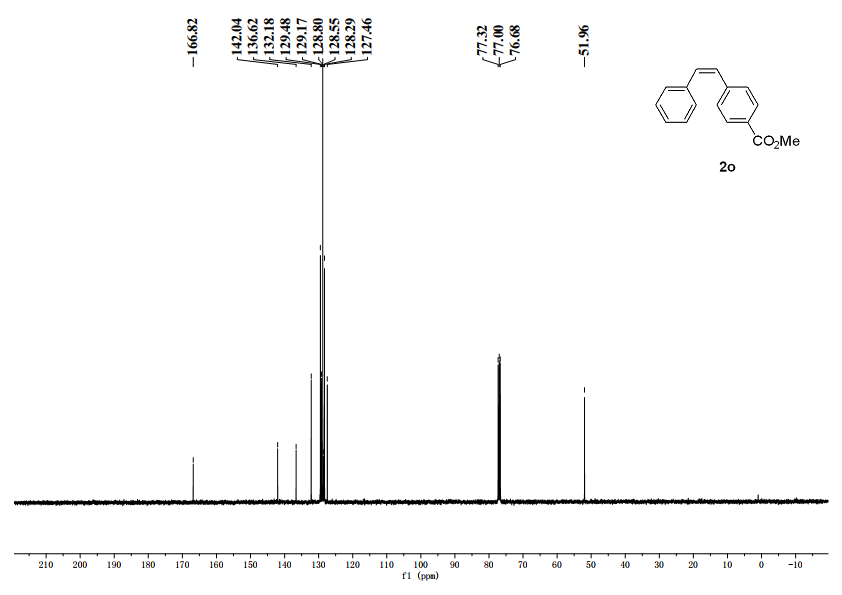
 Supplementary Figure 89. ^13^C NMR (101 MHz, CDCl_3_) spectra for compound **2o**


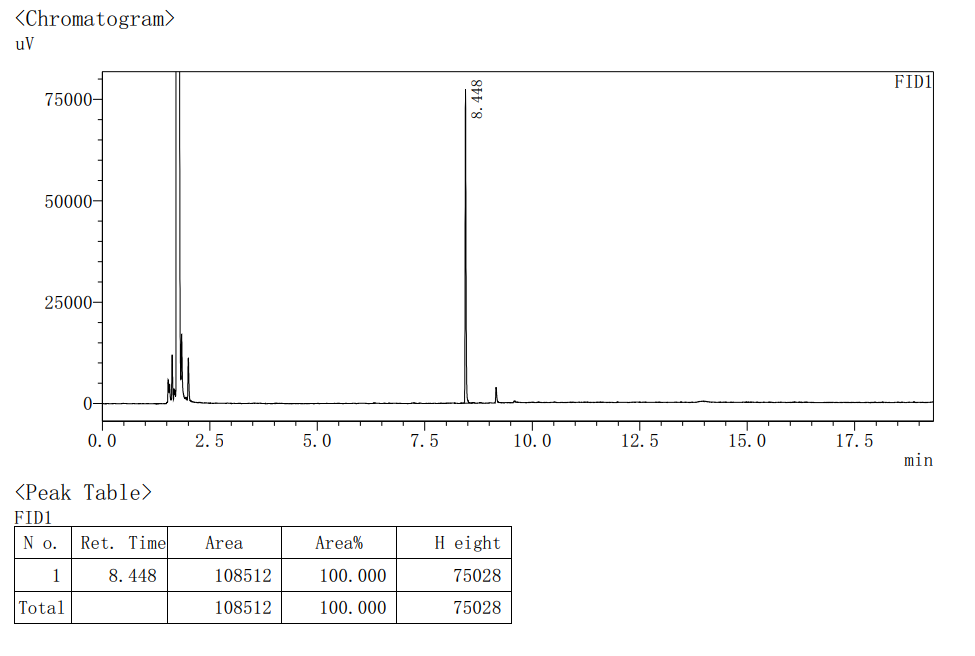


Supplementary Figure 90. GC spectra for **2p**


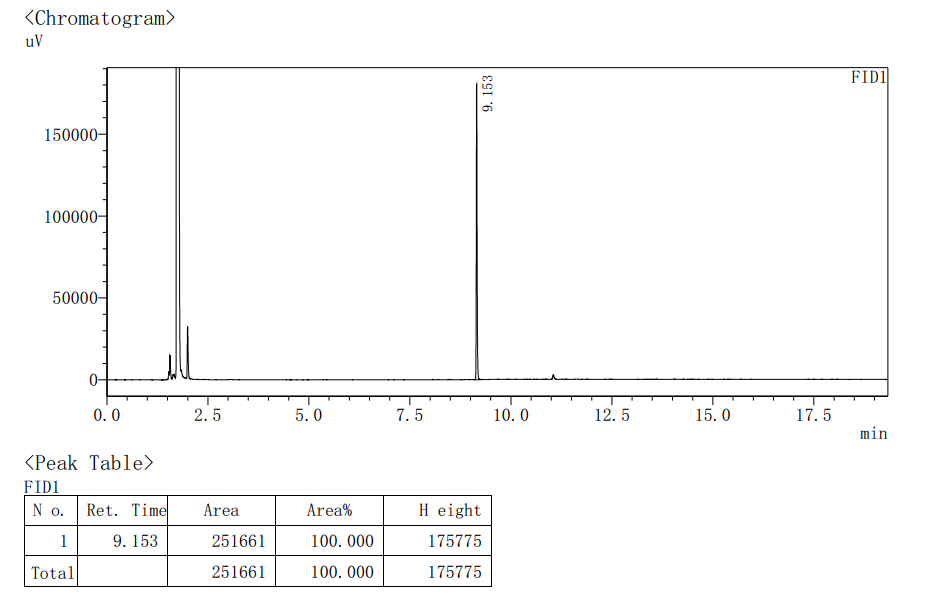


Supplementary Figure 91. GC spectra for **3p**


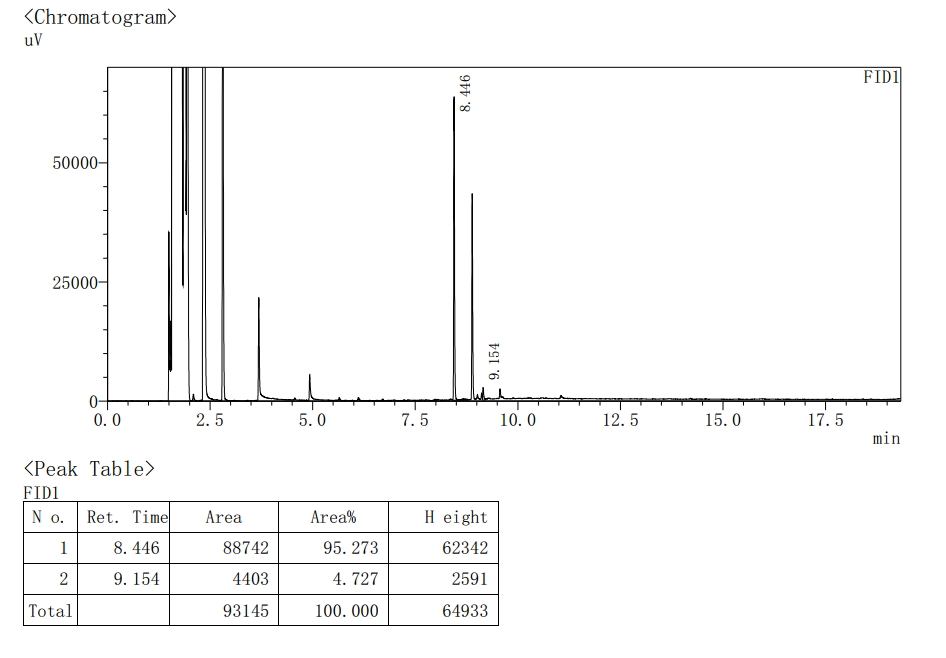
 Supplementary Figure 92. GC spectra for crude product of **2p**
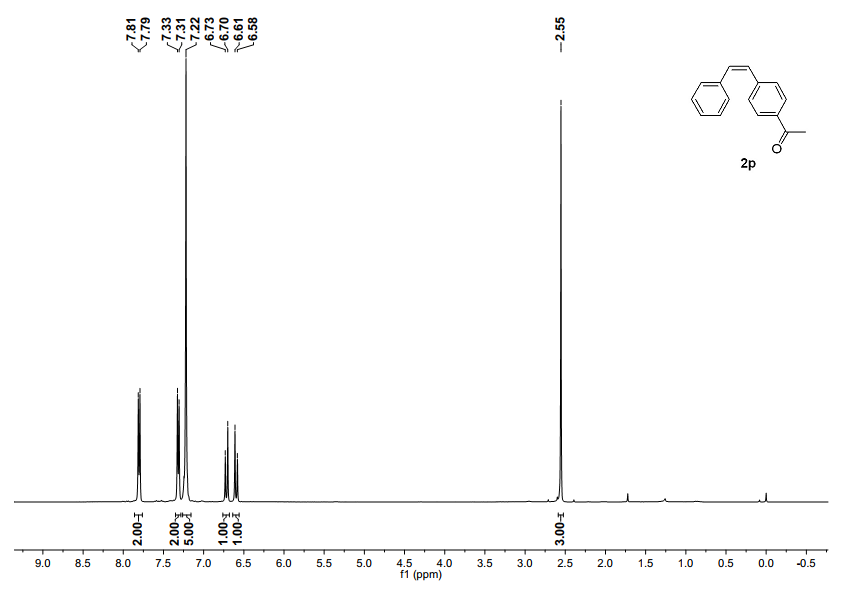
 Supplementary Figure 93. ^1^H NMR (400 MHz, CDCl_3_) spectra for compound **2p**
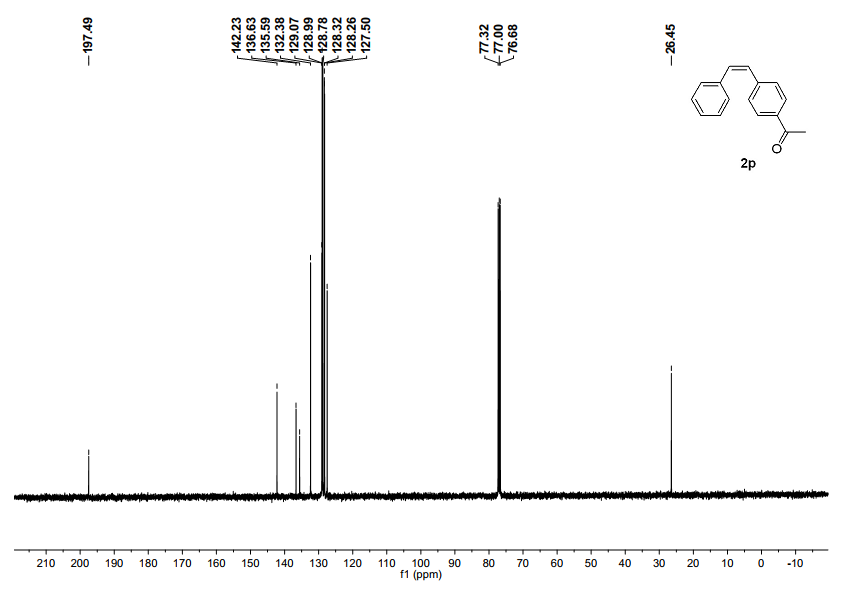
 Supplementary Figure 94. ^13^C NMR (101 MHz, CDCl_3_) spectra for compound **2p**


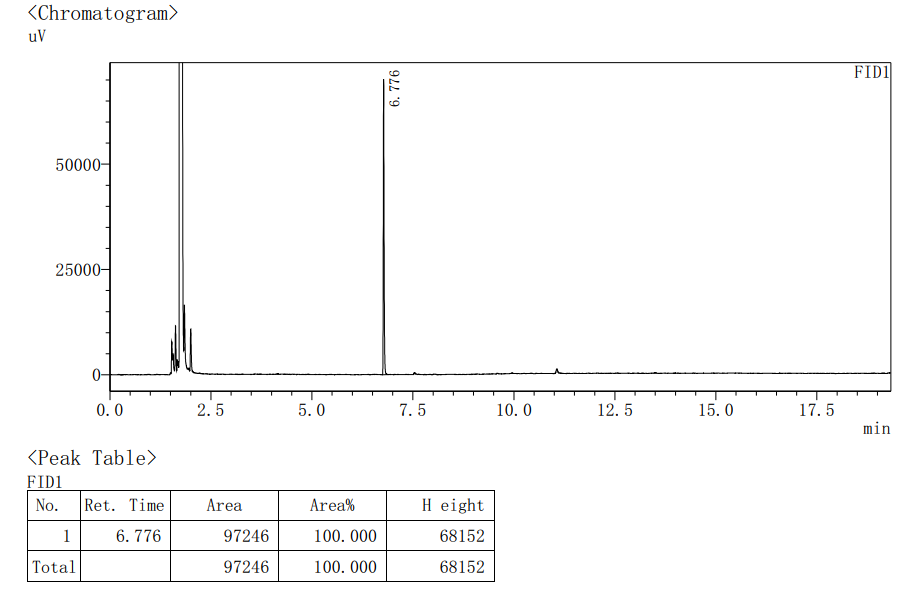
 Supplementary Figure 95. GC spectra for **2q**
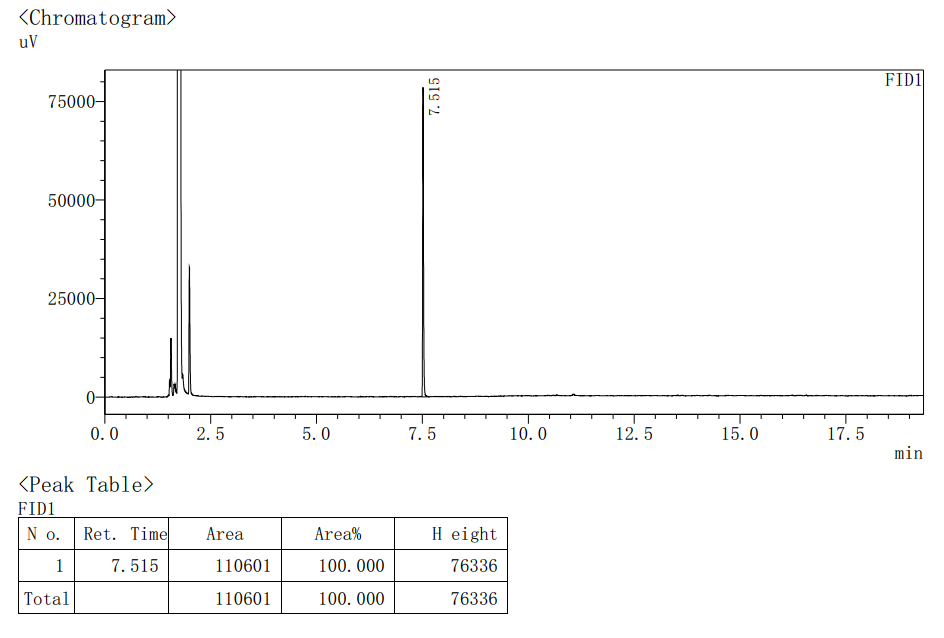
 Supplementary Figure 96. GC spectra for **3q**


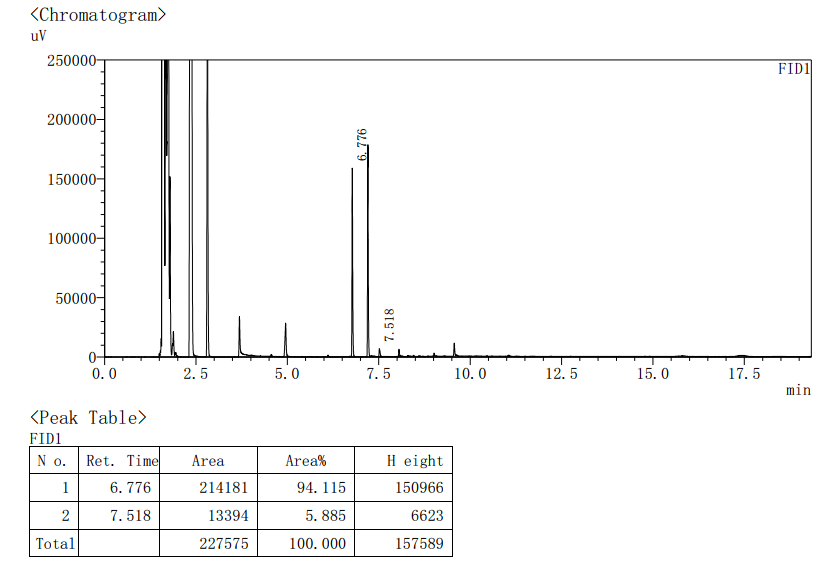
 Supplementary Figure 97. GC spectra for crude product of **2q**


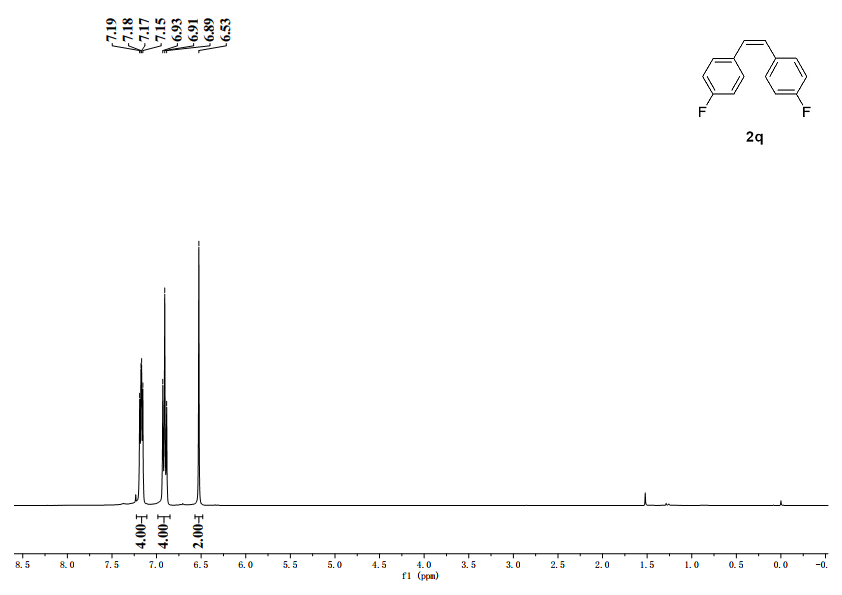
 Supplementary Figure 98. ^1^H NMR (400 MHz, CDCl_3_) spectra for compound **2q**
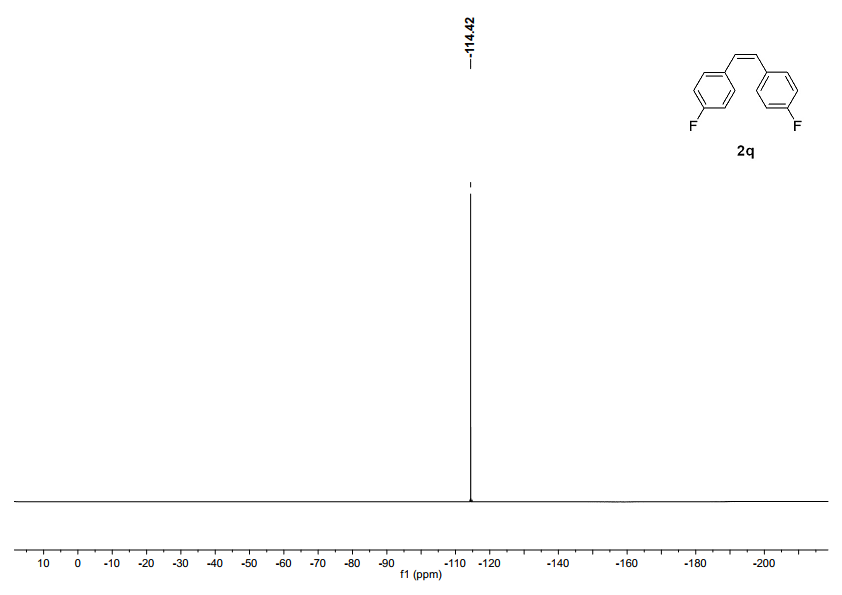
 Supplementary Figure 99. ^19^F NMR (376 MHz, CDCl_3_) spectra for compound **2q**
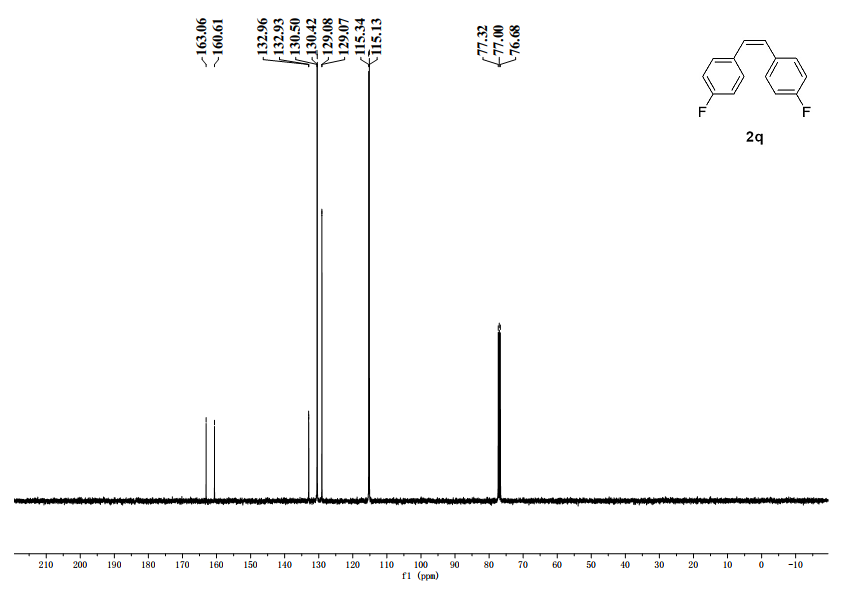
 Supplementary Figure 100. ^13^C NMR (101 MHz, CDCl_3_) spectra for compound **2q**

Supplementary Figure 101. GC spectra for **2r** Supplementary Figure 102. GC spectra for **3r**

Supplementary Figure 103. GC spectra for crude product of **2r** Supplementary Figure 104. ^1^H NMR (400 MHz, CDCl_3_) spectra for compound **2r** Supplementary Figure 105. ^19^F NMR (376 MHz, CDCl_3_) spectra for compound **2r** Supplementary Figure 106. ^13^C NMR (101 MHz, CDCl_3_) spectra for compound **2r**

Supplementary Figure 107. GC spectra for **2s**

Supplementary Figure 108. GC spectra for **3s**

Supplementary Figure 109. GC spectra for crude product of **2s** Supplementary Figure 110. ^1^H NMR (400 MHz, CDCl_3_) spectra for compound **2s** Supplementary Figure 111. ^13^C NMR (101 MHz, CDCl_3_) spectra for compound **2s**

Supplementary Figure 112. GC spectra for **2t**

Supplementary Figure 113. GC spectra for **3t**

Supplementary Figure 114. GC spectra for crude product of **2t** Supplementary Figure 115. ^1^H NMR (400 MHz, CDCl_3_) spectra for compound **2t** Supplementary Figure 116. ^13^C NMR (101 MHz, CDCl_3_) spectra for compound **2t**

Supplementary Figure 117. GC spectra for the mixture of **2u** and **3u**

Supplementary Figure 118. GC spectra for **3u** Supplementary Figure 119. GC spectra for crude product of **2u**

Supplementary Figure 120. ^1^H NMR (400 MHz, CDCl_3_) spectra for compound **2u** Supplementary Figure 121. ^13^C NMR (101 MHz, CDCl_3_) spectra for compound **2u**

Supplementary Figure 122. GC spectra for **2v** Supplementary Figure 123. GC spectra for the mixture of **2v** and **3v**

Supplementary Figure 124. GC spectra for crude product of **2v** Supplementary Figure 125. ^1^H NMR (400 MHz, CDCl_3_) spectra for compound **2v** Supplementary Figure 126. ^13^C NMR (101 MHz, CDCl_3_) spectra for compound **2v**

Supplementary Figure 127. GC spectra for **2w**

Supplementary Figure 128. GC spectra for *E*-isomer of **2w** Supplementary Figure 129. GC spectra for **2w**

Supplementary Figure 130. ^1^H NMR (400 MHz, CDCl_3_) spectra for compound **2w** Supplementary Figure 131. ^13^C NMR (101 MHz, CDCl_3_) spectra for compound **2w**

Supplementary Figure 132. GC spectra for **2x** Supplementary Figure 133. GC spectra for **3x**

Supplementary Figure 134. GC spectra for crude product of **2x** Supplementary Figure 135. ^1^H NMR (400 MHz, CDCl_3_) spectra for compound **2x** Supplementary Figure 136. ^13^C NMR (101 MHz, CDCl_3_) spectra for compound **2x**

Supplementary Figure137. GC spectra for **2y**

Supplementary Figure 138. GC spectra for **3y**

Supplementary Figure 139. GC spectra for crude product of **2y** Supplementary Figure 140. ^1^H NMR (400 MHz, CDCl_3_) spectra for compound **2y** Supplementary Figure 141. ^13^C NMR (101 MHz, CDCl_3_) spectra for compound **2y**

Supplementary Figure 142. GC spectra for **2z** Supplementary Figure 143. GC spectra for **3z**

Supplementary Figure 144. GC spectra for crude product of **2z**

Supplementary Figure 145. ^1^H NMR (400 MHz, CDCl_3_) spectra for compound **2z** Supplementary Figure 146. ^13^C NMR (101 MHz, CDCl_3_) spectra for compound **2z**

Supplementary Figure 147. GC spectra for **2bb**

Supplementary Figure 148. GC spectra for crude product of **2bb** Supplementary Figure 149. ^1^H NMR (400 MHz, CDCl_3_) spectra for compound **2bb** Supplementary Figure 150. ^13^C NMR (101 MHz, CDCl_3_) spectra for compound **2bb**

Supplementary Figure 151. GC spectra for **2cc**

Supplementary Figure 152. GC spectra for crude product of **2cc** Supplementary Figure 153. ^1^H NMR (400 MHz, CDCl_3_) spectra for compound **2cc** Supplementary Figure 154. ^13^C NMR (101 MHz, CDCl_3_) spectra for compound **2cc**

Supplementary Figure 155. GC spectra for **2dd** Supplementary Figure 156. GC spectra for crude product of **2dd**

Supplementary Figure 157. ^1^H NMR (400 MHz, CDCl_3_) spectra for compound **2dd** Supplementary Figure 158. ^13^C NMR (101 MHz, CDCl_3_) spectra for compound **2dd**

Supplementary Figure 159. GC spectra for **2ee**

Supplementary Figure 160. GC spectra for crude product of **2ee** Supplementary Figure 161. ^1^H NMR (400 MHz, CDCl_3_) spectra for compound **2ee** Supplementary Figure 162. ^13^C NMR (101 MHz, CDCl_3_) spectra for compound **2ee**

Supplementary Figure 163. GC spectra for **2ff**

Supplementary Figure 164. GC spectra for crude product of **2ff** Supplementary Figure 165. ^1^H NMR (400 MHz, CDCl_3_) spectra for compound **2ff** Supplementary Figure 166. ^13^C NMR (101 MHz, CDCl_3_) spectra for compound **2ff**

Supplementary Figure 167. GC spectra for **2gg** Supplementary Figure 168. GC spectra for crude product of **2gg**

Supplementary Figure 169. ^1^H NMR (400 MHz, CDCl_3_) spectra for compound **2gg** Supplementary Figure 170. ^13^C NMR (101 MHz, CDCl_3_) spectra for compound **2gg**

Supplementary Figure 171. GC spectra for **2ii**

Supplementary Figure 172. GC spectra for crude product of **3ii**

Supplementary Figure 173. GC spectra for crude product of **2ii** Supplementary Figure 174. ^1^H NMR (400 MHz, CDCl_3_) spectra for compound **2ii** Supplementary Figure 175. ^13^C NMR (101 MHz, CDCl_3_) spectra for compound **2ii** Supplementary Figure 176. GC spectra for **2a** Supplementary Figure 177. GC spectra for **3a** Supplementary Figure 178. GC spectra for crude product of **3a** Supplementary Figure 179. ^1^H NMR (400 MHz, CDCl_3_) spectra for compound **3a** Supplementary Figure 180. ^13^C NMR (101 MHz, CDCl_3_) spectra for compound **3a**

Supplementary Figure 181. GC spectra for **2b**

Supplementary Figure 182. GC spectra for **3b** Supplementary Figure 183. GC spectra for crude product of **3b**

Supplementary Figure 184. ^1^H NMR (400 MHz, CDCl_3_) spectra for compound **3b** Supplementary Figure 185. ^13^C NMR (101 MHz, CDCl_3_) spectra for compound **3b**

Supplementary Figure 186. GC spectra for **2c** Supplementary Figure 187. GC spectra for **3c** Supplementary Figure 188. GC spectra for crude product of **3c** Supplementary Figure 189. ^1^H NMR (400 MHz, CDCl_3_) spectra for compound **3c** Supplementary Figure 190. ^13^C NMR (101 MHz, CDCl_3_) spectra for compound **3c**

Supplementary Figure 191. GC spectra for **2d** Supplementary Figure 192. GC spectra for **3d** Supplementary Figure 193. GC spectra for crude product of **3d** Supplementary Figure 194. ^1^H NMR (400 MHz, CDCl_3_) spectra for compound **3d** Supplementary Figure 195. ^13^C NMR (101 MHz, CDCl_3_) spectra for compound **3d**

Supplementary Figure 196. GC spectra for **2e** Supplementary Figure 197. GC spectra for **3e**

Supplementary Figure 198. GC spectra for crude product of **3e**

Supplementary Figure 199. ^1^H NMR (400 MHz, CDCl_3_) spectra for compound **3e** Supplementary Figure 200. ^13^C NMR (101 MHz, CDCl_3_) spectra for compound **3e**

Supplementary Figure 201. GC spectra for **2g** Supplementary Figure 202. GC spectra for **3g**

Supplementary Figure 203. GC spectra for crude product of **3g**

Supplementary Figure 204. ^1^H NMR (400 MHz, CDCl_3_) spectra for compound **3g** Supplementary Figure 205. ^13^C NMR (101 MHz, CDCl_3_) spectra for compound **3g**

Supplementary Figure 206. GC spectra for **2i**

Supplementary Figure 207. GC spectra for **3i** Supplementary Figure 208. GC spectra for crude product of **3i**

Supplementary Figure 209. ^1^H NMR (400 MHz, CDCl_3_) spectra for compound **3i** Supplementary Figure 210. ^13^C NMR (101 MHz, CDCl_3_) spectra for compound **3i**

Supplementary Figure 211. GC spectra for **2j** Supplementary Figure 212. GC spectra for **3j** Supplementary Figure 213. GC spectra for crude product of **3j**

Supplementary Figure 214. ^1^H NMR (400 MHz, CDCl_3_) spectra for compound **3j**

Supplementary Figure 215. ^13^C NMR (101 MHz, CDCl_3_) spectra for compound **3j**

Supplementary Figure 216. GC spectra for **2k** Supplementary Figure 217. GC spectra for **3k**

Supplementary Figure 218. GC spectra for crude product of **3k**

Supplementary Figure 219. ^1^H NMR (400 MHz, CDCl_3_) spectra for compound **3k** Supplementary Figure 220. ^13^C NMR (101 MHz, CDCl_3_) spectra for compound **3k**

Supplementary Figure 221. GC spectra for **2l**

Supplementary Figure 222. GC spectra for **3l**

Supplementary Figure 223. GC spectra for crude product of **3l**

Supplementary Figure 224. ^1^H NMR (300 MHz, CDCl_3_) spectra for compound **3l** Supplementary Figure 225. ^19^F NMR (376 MHz, CDCl_3_) spectra for compound **3l** Supplementary Figure 226. ^13^C NMR (75 MHz, CDCl_3_) spectra for compound **3l**

Supplementary Figure 227. GC spectra for **2m** Supplementary Figure 228. GC spectra for **3m**

Supplementary Figure 229. GC spectra for crude product of **3m**

Supplementary Figure 230. ^1^H NMR (400 MHz, CDCl_3_) spectra for compound **3m** Supplementary Figure 231. ^13^C NMR (101 MHz, CDCl_3_) spectra for compound **3m**

Supplementary Figure 232. GC spectra for **2o** Supplementary Figure 233. GC spectra for **3o**

Supplementary Figure 234. GC spectra for crude product of **3o** Supplementary Figure 235. ^1^H NMR (400 MHz, CDCl_3_) spectra for compound **3o** Supplementary Figure 236. ^13^C NMR (101 MHz, CDCl_3_) spectra for compound **3o**

Supplementary Figure 237. GC spectra for **2p**

Supplementary Figure 238. GC spectra for **3p** Supplementary Figure 239. GC spectra for crude product of **3p**

Supplementary Figure 240. ^1^H NMR (400 MHz, CDCl_3_) spectra for compound **3p** Supplementary Figure 241. ^13^C NMR (101 MHz, CDCl_3_) spectra for compound **3p**

Supplementary Figure 242. GC spectra for **2q** Supplementary Figure 243. GC spectra for **3q**

Supplementary Figure 244. GC spectra for crude product of **3q**

Supplementary Figure 245. ^1^H NMR (300 MHz, CDCl_3_) spectra for compound **3q** Supplementary Figure 246. ^19^F NMR (376 MHz, CDCl_3_) spectra for compound **3q** Supplementary Figure 247. ^13^C NMR (75 MHz, CDCl_3_) spectra for compound **3q**

Supplementary Figure 248. GC spectra for **2r** Supplementary Figure 249. GC spectra for **3r**

Supplementary Figure 250. GC spectra for crude product of **3r** Supplementary Figure 251. ^1^H NMR (400 MHz, CDCl_3_) spectra for compound **3r** Supplementary Figure 252. ^19^F NMR (376 MHz, CDCl_3_) spectra for compound **3r** Supplementary Figure 253. ^13^C NMR (101 MHz, CDCl_3_) spectra for compound **3r**

Supplementary Figure 254. GC spectra for **2s**

Supplementary Figure 255. GC spectra for **3s**

Supplementary Figure 256. GC spectra for crude product of **3s**

Supplementary Figure 257. ^1^H NMR (300 MHz, CDCl_3_) spectra for compound **3s** Supplementary Figure 258. ^13^C NMR (75 MHz, CDCl_3_) spectra for compound **3s**

Supplementary Figure 259. GC spectra for **2t**

Supplementary Figure 260. GC spectra for **3t**

Supplementary Figure 261. GC spectra for crude product of **3t**

Supplementary Figure 262. ^1^H NMR (400 MHz, CDCl_3_) spectra for compound **3t** Supplementary Figure 263. ^13^C NMR (101 MHz, CDCl_3_) spectra for compound **3t**

Supplementary Figure 264. GC spectra for the mixture of **2u** and **3u**

Supplementary Figure 265. GC spectra for **3u**

Supplementary Figure 266. GC spectra for crude product of **3u**

Supplementary Figure 267. ^1^H NMR (400 MHz, CDCl_3_) spectra for compound **3u** Supplementary Figure 268. ^13^C NMR (101 MHz, CDCl_3_) spectra for compound **3u**

Supplementary Figure 269. GC spectra for **2v** Supplementary Figure 270. GC spectra for the mixture of **2v** and **3v**

Supplementary Figure 271. GC spectra for crude product of **3v** Supplementary Figure 272. ^1^H NMR (400 MHz, CDCl_3_) spectra for compound **3v** Supplementary Figure 273. ^13^C NMR (101 MHz, CDCl_3_) spectra for compound **3v**

Supplementary Figure 274. GC spectra for **2x** Supplementary Figure 275. GC spectra for **3x**

Supplementary Figure 276. GC spectra for crude product of **3x** Supplementary Figure 277. ^1^H NMR (400 MHz, CDCl_3_) spectra for compound **3x** Supplementary Figure 278. ^13^C NMR (101 MHz, CDCl_3_) spectra for compound **3x**

Supplementary Figure 279. GC spectra for **2y**

Supplementary Figure 280. GC spectra for **3y**

Supplementary Figure 281. GC spectra for crude product of **3y** Supplementary Figure 282. ^1^H NMR (400 MHz, CDCl_3_) spectra for compound **3y** Supplementary Figure 283. ^13^C NMR (101 MHz, CDCl_3_) spectra for compound **3y**

Supplementary Figure 284. GC spectra for **2z** Supplementary Figure 285. GC spectra for **3z**

Supplementary Figure 286. GC spectra for crude product of **3z**

Supplementary Figure 287. ^1^H NMR (400 MHz, CDCl_3_) spectra for compound **3z** Supplementary Figure 288. ^13^C NMR (101 MHz, CDCl_3_) spectra for compound **3z**

Supplementary Figure 289. GC spectra for **3aa** Supplementary Figure 290. GC spectra for crude product of **3aa**

Supplementary Figure 291. ^1^H NMR (300 MHz, CDCl_3_) spectra for compound **3aa** Supplementary Figure 292. ^13^C NMR (101 MHz, CDCl_3_) spectra for compound **3aa**

Supplementary Figure 293. GC spectra for **2b** Supplementary Figure 294. GC spectra for crude product of **3hh**

Supplementary Figure 295. ^1^H NMR (300 MHz, CDCl_3_) spectra for compound **3hh** Supplementary Figure 296. ^13^C NMR (101 MHz, CDCl_3_) spectra for compound **3hh**

Supplementary Figure 297. GC spectra for **2ii**

Supplementary Figure 298. GC spectra for **3ii**

Supplementary Figure 299. GC spectra for crude product of **3ii**

Supplementary Figure 300. ^1^H NMR (400 MHz, CDCl_3_) spectra for compound **3ii** Supplementary Figure 301. ^13^C NMR (101 MHz, CDCl_3_) spectra for compound **3ii**

Supplementary Figure 302. GC spectra for **3jj** Supplementary Figure 303. GC spectra for crude product of **3jj** Supplementary Figure 304. ^1^H NMR (400 MHz, CDCl_3_) spectra for compound **3jj** Supplementary Figure 305. ^13^C NMR (101 MHz, CDCl_3_) spectra for compound **3jj**

Supplementary Figure 306. GC spectra for **3kk**

Supplementary Figure 307. GC spectra for crude product of **3kk**

Supplementary Figure 308. ^1^H NMR (400 MHz, CDCl_3_) spectra for compound **3kk** Supplementary Figure 309. ^13^C NMR (101 MHz, CDCl_3_) spectra for compound **3kk**

Supplementary Figure 310. GC spectra for **3ll**

Supplementary Figure 311. GC spectra for crude product of **3ll**

Supplementary Figure 312. ^1^H NMR (300 MHz, CDCl_3_) spectra for compound **3ll** Supplementary Figure 313. ^13^C NMR (101 MHz, CDCl_3_) spectra for compound **3ll** Supplementary Figure 314. ^1^H NMR (400 MHz, CDCl_3_) spectra for compound **6** Supplementary Figure 315. ^13^C NMR (101 MHz, CDCl_3_) spectra for compound **6**

Supplementary Figure 316. GC spectra for **7** Supplementary Figure 317. GC spectra for *ZE*- and *EE*-isomer of **7** Supplementary Figure 318. GC spectra for crude product of **7** Supplementary Figure 319. ^1^H NMR (400 MHz, CDCl_3_) spectra for compound **7** Supplementary Figure 320. ^13^C NMR (101 MHz, CDCl_3_) spectra for compound **7**

Supplementary Figure 321. GC spectra for **8** Supplementary Figure 322. GC spectra for crude product of **8**

Supplementary Figure 323. ^1^H NMR (400 MHz, CDCl_3_) spectra for compound **8** Supplementary Figure 324. ^13^C NMR (101 MHz, CDCl_3_) spectra for compound **8**

Supplementary Figure 325. GC spectra for **9**

Supplementary Figure 326. GC spectra for crude product of **9**

Supplementary Figure 327. ^1^H NMR (400 MHz, CDCl_3_) spectra for compound **9** Supplementary Figure 328. ^13^C NMR (101 MHz, CDCl_3_) spectra for compound **9**

Supplementary Figure 329. GC spectra for **10**

Supplementary Figure 330. GC spectra for crude product of **10**

Supplementary Figure 331. ^1^H NMR (400 MHz, CDCl_3_) spectra for compound **10** Supplementary Figure 332. ^13^C NMR (101 MHz, CDCl_3_) spectra for compound **10**
